# Supplementary figures and images for: Origin and evolutionary landscape of Nr2f transcription factors across Metazoa
Source: PLoS One. 2021 Nov 22;16(11):e0254282. doi: 10.1371/journal.pone.0254282 (PMC8608329; doi:10.1371/journal.pone.0254282)

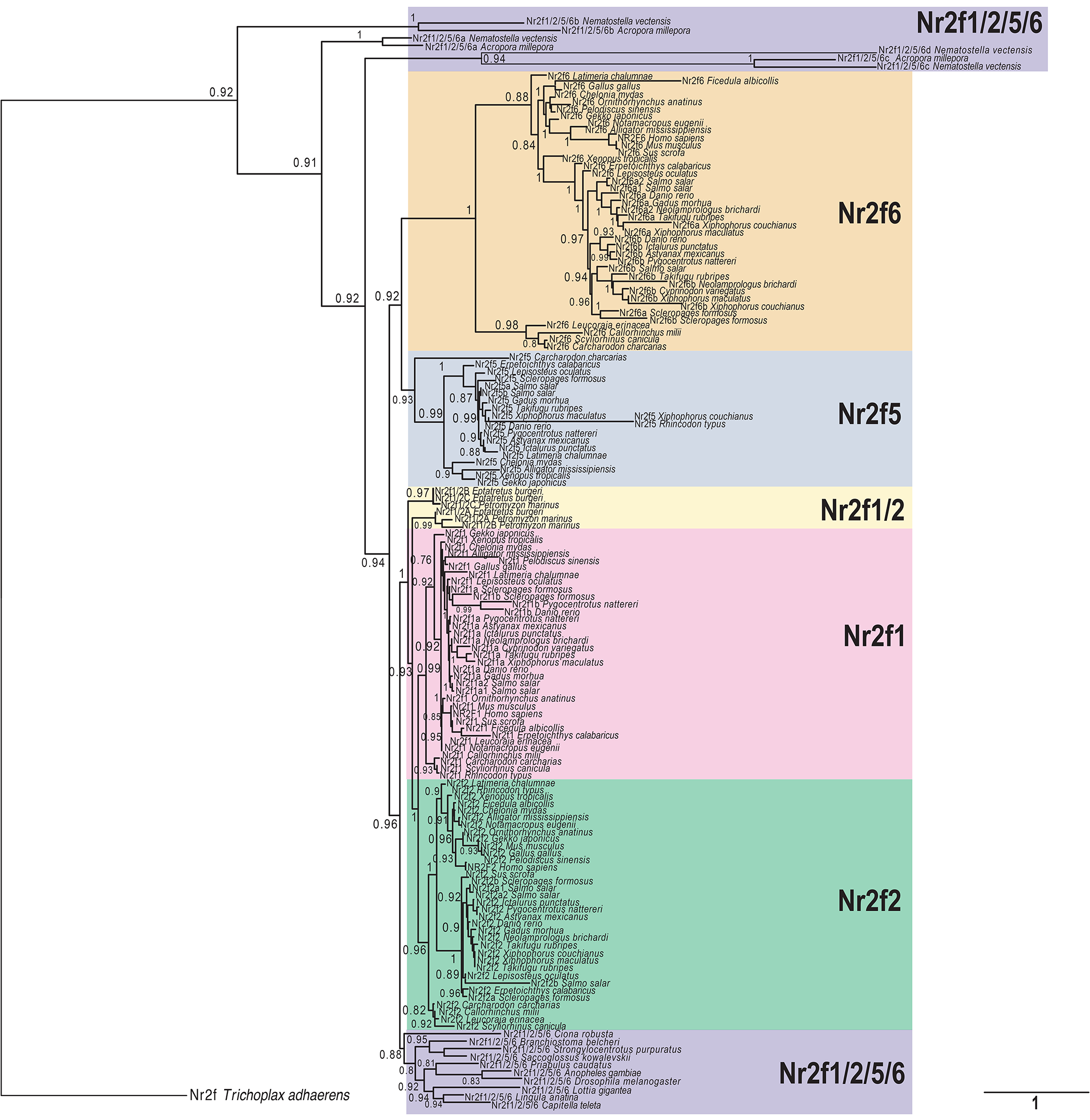

Supplement: S1 Fig — The same color code as Fig 2 is used. Values at the branches indicate replicates obtained employing the aBayes method. (TIF) [file pone.0254282.s001.tif]

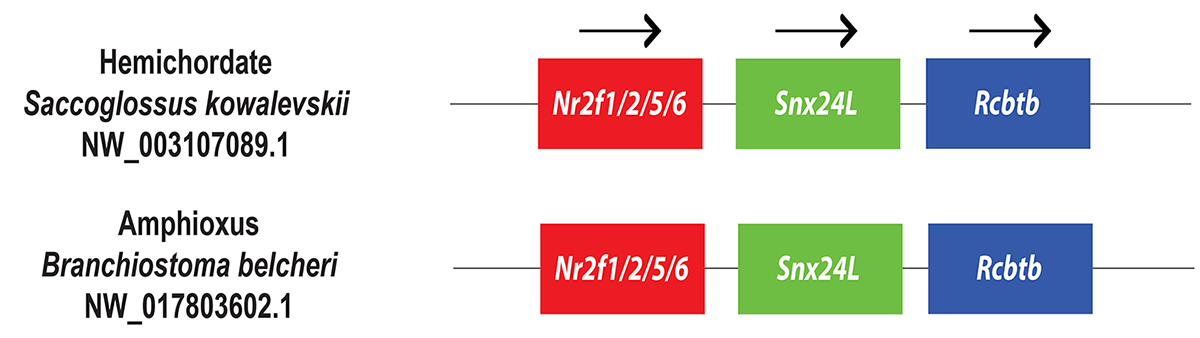

Supplement: S2 Fig — Schematic of limited conservation for Nr2f1/2/5/6 loci between the hemichordate S. kowalevskii and amphioxus B. belcheri. Black arrows indicate transcription orientation. (TIF) [file pone.0254282.s002.tif]

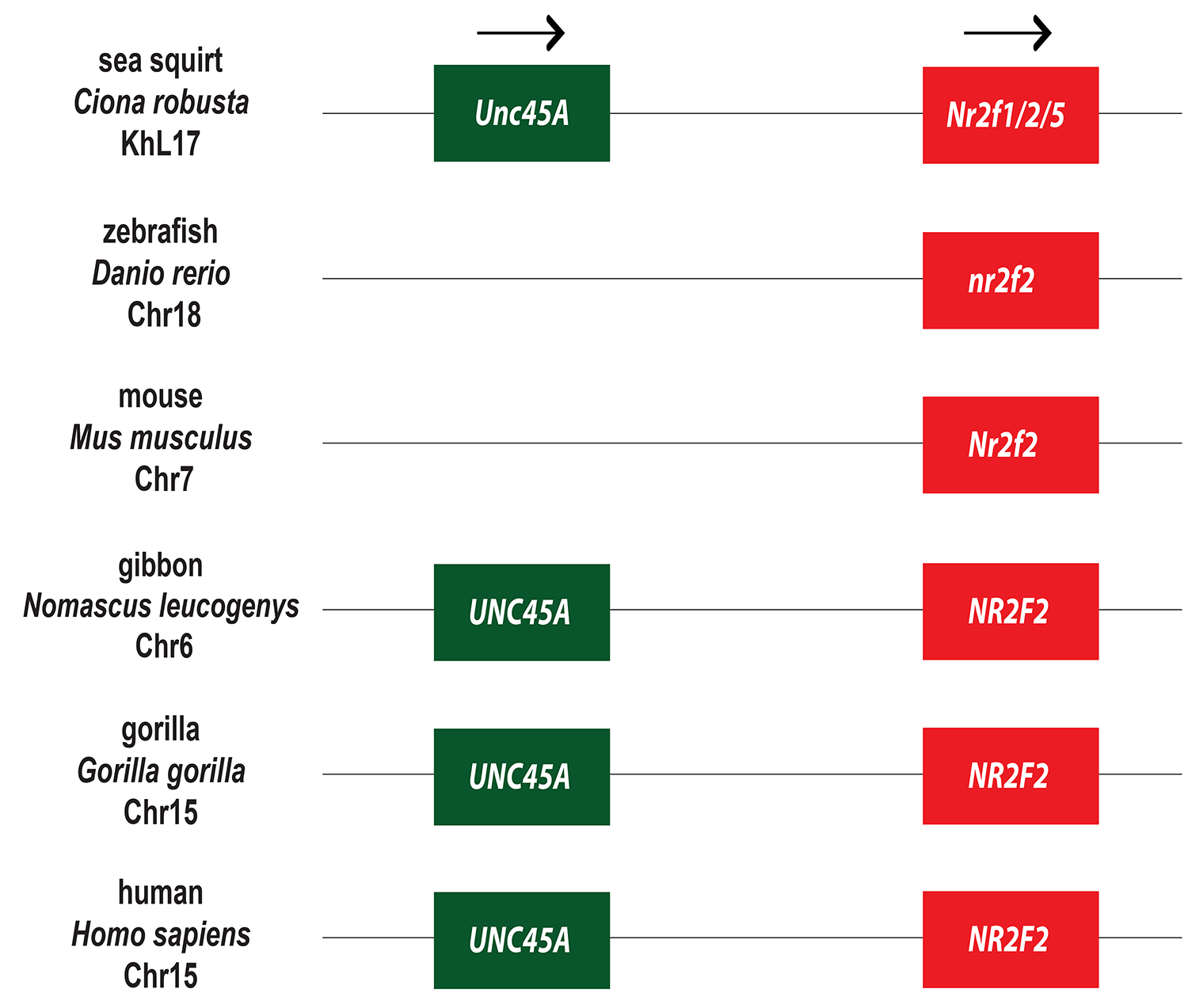

Supplement: S3 Fig — Schematic of Unc45-Nr2f duplet conservation in genomes of ascidians (Ciona) and primates. The duplet is absent in other vertebrate models, including zebrafish and mouse. (TIF) [file pone.0254282.s003.tif]

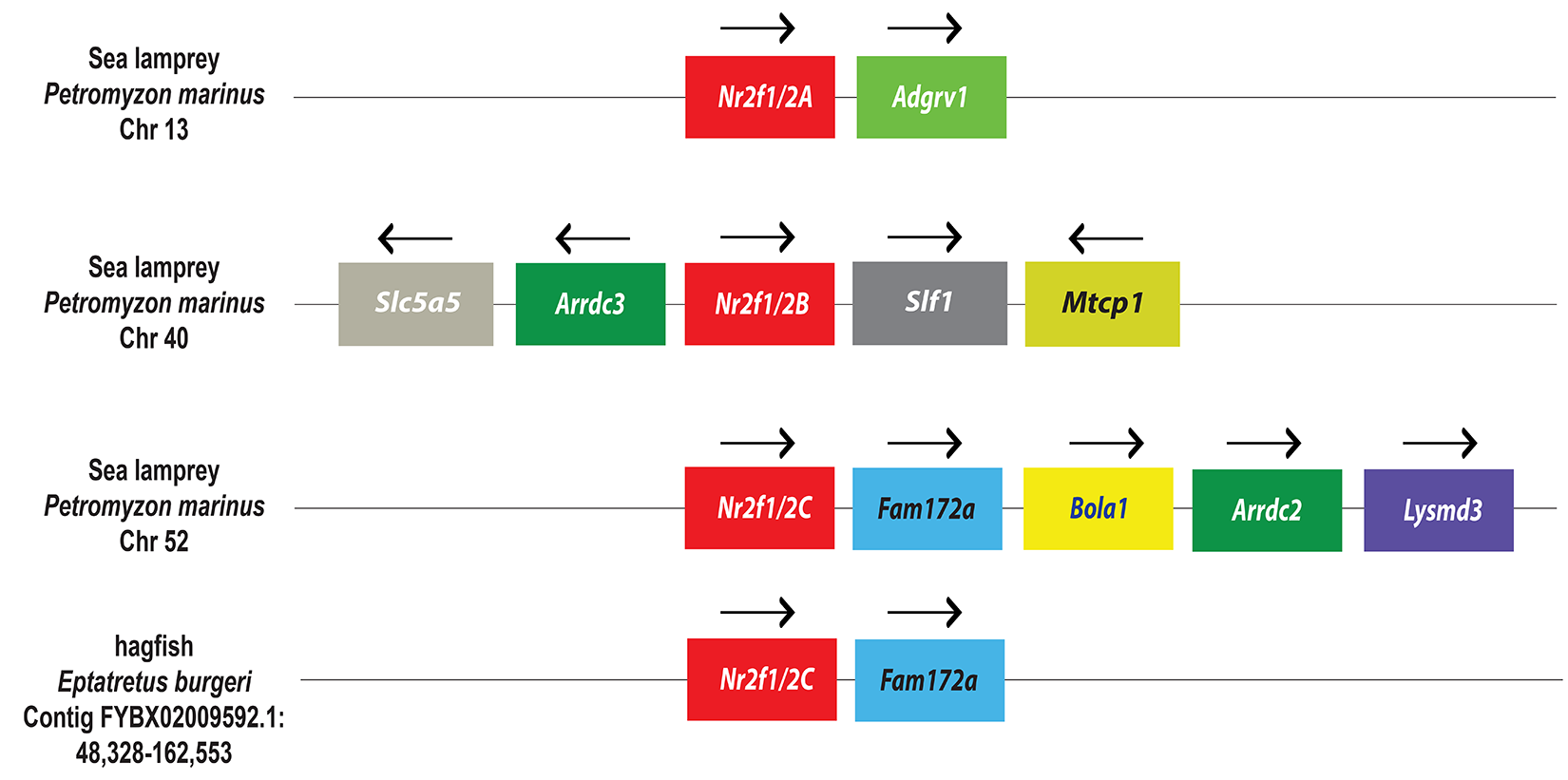

Supplement: S4 Fig — Schematic of lamprey (P. marinus) Nr2f1/2 loci with relative chromosomes and available genomic data from the hagfish (E. burgeri). Genomic data could only be obtained for the hagfish Nr2f1/2C gene. Same color code of Figs 6–8 is used. Flanking genes are in common with gnathostomes, with Arrdc2 and Arrdc3 (green) that form a conserved duplet with Nr2f1/2B and Nr2f1/2C. Nr2f1/2C is adjacent to Fam172a in both lamprey and hagfish. Arrows indicate transcription orientation. (TIF) [file pone.0254282.s004.tif]
