# Supplementary material for: Origin and evolutionary landscape of Nr2f transcription factors across Metazoa
Source: PLoS One. 2021 Nov 22;16(11):e0254282. doi: 10.1371/journal.pone.0254282 (PMC8608329; doi:10.1371/journal.pone.0254282)
Supplement: S4 File — Alignment of specific and conserved intron/exon boundaries within the Zinc finger motifs of DBD (underlined) and LBDs (red). The intron phases have been depicted using color code: Phase 0 (yellow), Phase 1 (green), Phase 2 (turquoise). (DOCX) [file pone.0254282.s008.docx]

**S4 File. Intron/exon structure of *Nr2f* genes in Metazoa**

phase 0

phase 1

phase 2

**Nr2f *Trichoplax adhaerens*  ------------------------------------------------------------**

**Nr2f1/2/5/6 *Lottia gigantea* -----------------------------------------MTPTQPTVTSWRDHTDDLV**

**Nr2f1/2/5/6 *Ciona robusta* -------------------------------------------MAMV-VTSWRESNHHDD**

**Nr2f1/2A *Petromyzon marinus*  ----------------------MSLDSLPHFYDALRSPTSPQAMAMV-VGPWRDPQGDLT**

**Nr2f1/2A *Eptatretus burgeri*  -------------------------------------------MAMV-VGPWRDPQDDLT**

**Nr2f1/2B *Petromyzon marinus*  MEAYGHTGDDMGRHLQLARLATYGAKEIQHTYQ----ASPRRAMAMV-VGPWRDPQDEMA**

**Nr2f1/2B *Eptatretus burgeri*  -------------------------------------------MAMV-VSAWRDPQDDVA**

**Nr2f1/2C *Petromyzon marinus*  -------------------------------------------MAMVVGNPWREPPNG--**

**Nr2f1/2C *Eptatretus burgeri*  -------------------------------------------MAMVVSATWRDPLDNSP**

**Nr2f1a *Danio rerio* -------------------------------------------MAMV-VSVWRDPQEDVA**

**Nr2f1b *Danio rerio* -------------------------------------------MAMV-VSAWRDPQEELA**

**Nr2f1 *Xenopus tropicalis*  -------------------------------------------MAMV-VSSWRDPQEDVA**

**Nr2f1 *Latimeria chalumnae*  -------------------------------------------MAMV-VSSWRDPQEDVA**

**NR2F1 *Homo sapiens*  -------------------------------------------MAMV-VSSWRDPQDDVA**

**Nr2f2 *Danio rerio*  ---------------------------------------------MA-MVVWRGSQDDVA**

**Nr2f2 *Latimeria chalumnae*  -------------------------------------------MAMV-VSAWRDPQDDVA**

**Nr2f2 *Xenopus tropicalis*  -------------------------------------------MAMV-VGAWRDPQDDMP**

**NR2F2 *Homo sapiens*  -------------------------------------------MAMV-VSTWRDPQDEVP**

**Nr2f5 *Danio rerio*  -------------------------------------------MAMV-VNQWQENISADP**

**Nr2f5 *Latimeria chalumnae*  -------------------------------------------MAMV-VNVWQEDIPGAS**

**Nr2f5 *Xenopus tropicalis*  -------------------------------------------MAMV-VNPWQEDIPGVP**

**Nr2f6a  *Danio rerio*  -------------------------------------------MAMV-RGGWGDPNGETN**

**Nr2f6b  *Danio rerio*  -------------------------------------------MAMV-SGGWANPNGSAN**

**Nr2f6 *Latimeria chalumnae*  -------------------------------------------MAMV-TGGWGDPNGETN**

**Nr2f6 *Xenopus tropicalis*  -------------------------------------------MAMV-SGGWGDPNGDTN**

**NR2F6 *Homo sapiens*  -------------------------------------------MAMV-TGGWGGPGGDTN**

**Nr2f *Trichoplax adhaerens* --------------------------------------MS-----TRSSEYIDSP--TAA**

**Nr2f1/2/5/6 *Lottia gigantea* GTTPTVVPPP--------LP------PPNPTVTPTQTQIPTATTPTPQQNG-GS-----P**

**Nr2f1/2/5/6 *Ciona robusta* VTVLQPANHEAVSTPLPPHPQ------PPPDLTPQTPTTPTNDVTIPDC-----STSRPT**

**Nr2f1/2A *Petromyzon marinus* SPPNVPGQAGQA--------------PLAP-AGPQTPQTPSQSGGPPTTPGQGSTQ----**

**Nr2f1/2A *Eptatretus burgeri* SGQNGPSQAVQP--------------PLAP-GGPQTPQTPSQS-GPPTTPGQGSQQ----**

**Nr2f1/2B *Petromyzon marinus* GPPS---QTGQP--------------PLAPAAGPHTPQTPGHAGPPPTTPGQGSTQ----**

**Nr2f1/2B *Eptatretus burgeri* GAQGTQPSQAPPG-QGPPTG------------APHTPQTPVQVGPPTTPAQSNQTNQPNQ**

**Nr2f1/2C *Petromyzon marinus* ------------------------------------------------------------**

**Nr2f1/2C *Eptatretus burgeri* APGAAAPIAQ---------SVPGAASVGASQGSSGALSA------PSSASSSSSASSTAS**

**Nr2f1a *Danio rerio* GGPPSGPNPAAQP-AR--------EQQQAASAAPHTPQTPSQPGPPSTPGTAG---DKGS**

**Nr2f1b *Danio rerio* AVDDQSAAGREHL------------------------------------QHRHSPKSAEE**

**Nr2f1 *Xenopus tropicalis*  GGTPSGPNPAAQS-AR--------EQQQTQSAAPHTPQTPGQPGPPSTPGTAG---DKGQ**

**Nr2f1 *Latimeria chalumnae*  GGNPGGPNPGA----R--------EQQQAPSAAPHTPQTPSQPGPPSTPGAAG---DKGQ**

**NR2F1 *Homo sapiens*  GGNPGGPNPAAQA-ARGGGGGAGEQQQQAGSGAPHTPQTPGQPGAPATPGTAG---DKGQ**

**Nr2f2 *Danio rerio*  ETHGTLSSQTQGG-LSLPTPQPGQLGLTASQVAPPTPQTPVQGPPNNNNNTQSTPTNQTT**

**Nr2f2 *Latimeria chalumnae*  GAQGTQPSQAPPG-QGPPTG------------APHTPQTPVQVGPPTTPAQSNQTNQPNQ**

**Nr2f2 *Xenopus tropicalis*  G---TQPSQAPPG-QGP-NG------------APHTPQTPGQGVPSTTPAQSNPSSQPSQ**

**NR2F2 *Homo sapiens*  G---SQASQAPPV-PGPPPG------------APHTPQTPGQGGPASTPAQTAAGGQGGP**

**Nr2f5 *Danio rerio*  GSQL----Q---------------MCSQEPGGTP---GTPSGSTPGND------------**

**Nr2f5 *Latimeria chalumnae*  GSQARSQPQ---------------MCTQEAGGTPQTPGTPAGSTPGQE------------**

**Nr2f5 *Xenopus tropicalis*  GSQVNNPPG---------------LCNQDPGGTPQTPTTPKGGVPGQD------------**

**Nr2f6a  *Danio rerio*  GLGDKGYLRG--D---------------EDDGS------PQGG---------GS--D--M**

**Nr2f6b  *Danio rerio*  GLGEKGYLRG--E---------------EEGSS------PQAG---------NS--D--V**

**Nr2f6 *Latimeria chalumnae*  GVIK-GYPRKSEE---------------EEEAS------PQGG---------GS--D--Q**

**Nr2f6 *Xenopus tropicalis*  GVGK-GYPRNSEE----------------EEAS------PQGG---------MS--D--P**

**NR2F6 *Homo sapiens*  GVDKAGGYPRAAE---------------DDSAS------PPGA---------AS--D--A**

**Nr2f *Trichoplax adhaerens* AKDETKSLSKELCCLICGDRSNGRHYGVISCEGCKGFFKRSVRRNMKYACTCSANACKIT**

**Nr2f1/2/5/6 *Lottia gigantea* NSETNNNNKQHIECVVCGDKSSGKHYGQYTCEGCKSFFKRSVRRNLNYTCRGN-KNCPID**

**Nr2f1/2/5/6 *Ciona robusta* GNTSSTSEKPQIECVVCGDKSSGKHYGQYTCEGCKSFFKRSVRRNLSYTCRGN-RNCPID**

**Nr2f1/2A *Petromyzon marinus* GDKQQQQQQPNVECVVCGDKSSGKHYGQFTCEGCKSFFKRSVRRNLTYTCRAN-RNCPID**

**Nr2f1/2A *Eptatretus burgeri* GDKQ---QQPNVECVVCGDKSSGKHYGQFTCEGCKSFFKRSVRRNLTYTCRAN-RNCPID**

**Nr2f1/2B *Petromyzon marinus* GDKQ----QSNVECVVCGDKSSGKHYGQFTCEGCKSFFKRSVRRNLTYTCRAN-RNCPID**

**Nr2f1/2B *Eptatretus burgeri* QN-QVEKQQQHIECVVCGDKSSGKHYGQFTCEGCKSFFKRSVRRNLSYTCRAN-RNCPID**

**Nr2f1/2C *Petromyzon marinus* ---TPIASGQNAECVVCGDKSSGKHYGQLTCEGCKSFFKRSVRRNLNYACRAA-RACPID**

**Nr2f1/2C *Eptatretus burgeri* SAPGDKTPLQHIECVVCGDKSSGKHYGQFTCEGCKSFFKRSVRRNLSYTCRAS-RNCPID**

**Nr2f1a *Danio rerio* QNSGQS-Q-QHIECVVCGDKSSGKHYGQFTCEGCKSFFKRSVRRNLTYTCRAN-RNCPID**

**Nr2f1b *Danio rerio* KAQIAAQNQQHVECVVCGDKSSGKHYGQFTCEGCKSFFKRSVRRNLSYTCRAN-RNCPVD**

**Nr2f1 *Xenopus tropicalis*  QGSGQS-QQQHIECVVCGDKSSGKHYGQFTCEGCKSFFKRSVRRNLTYTCRAN-RNCPID**

**Nr2f1 *Latimeria chalumnae*  NQQNSGQSQQHIECVVCGDKSSGKHYGQFTCEGCKSFFKRSVRRNLTYTCRAN-RNCPID**

**NR2F1 *Homo sapiens*  GPPGSGQSQQHIECVVCGDKSSGKHYGQFTCEGCKSFFKRSVRRNLTYTCRAN-RNCPID**

**Nr2f2 *Danio rerio*  QSQSEKQQPQHIECVVCGDKSSGKHYGQFTCEGCKSFFKRSVRRNLTYTCRAN-RNCPID**

**Nr2f2 *Latimeria chalumnae*  QN-QVEKQQQHIECVVCGDKSSGKHYGQFTCEGCKSFFKRSVRRNLSYTCRAN-RNCPID**

**Nr2f2 *Xenopus tropicalis*  NQGEKQQQQQHIECVVCGDKSSGKHYGQFTCEGCKSFFKRSVRRNLTYTCRAN-RNCPID**

**NR2F2 *Homo sapiens*  PGSDKQQQQQHIECVVCGDKSSGKHYGVFTCEGCKSFFKRSVRRNLSYTCRAN-RNCPID**

**Nr2f5 *Danio rerio*  ALSG--DKIPNVDCMVCGDKSSGKHYGQFTCEGCKSFFKRSVRRNLSYTCRGN-RDCPID**

**Nr2f5 *Latimeria chalumnae*  ALSG--DRAPAVDCMVCGDKSSGKHYGQFTCEGCKSFFKRSVRRNLSYTCRGN-RDCPID**

**Nr2f5 *Xenopus tropicalis*  PVHSGDKGVPNVDCLVCGDKSSGKHYGQFTCEGCKSFFKRSVRRNLTYTCRGN-RDCPID**

**Nr2f6a  *Danio rerio*  EAGEDD-KGCVVDCVVCGDKSSGKHYGVFTCEGCKSFFKRSVRRNLNYTCRSN-RDCQID**

**Nr2f6b  *Danio rerio*  EGGEED-KACVVDCVVCGDKSSGKHYGVFTCEGCKSFFKRSIRRNLNYTCRSN-RECQID**

**Nr2f6 *Latimeria chalumnae*  EHGEEDKPGIQVDCVVCGDKSSGKHYGAFTCEGCKSFFKRSIRRNLNYTCRSN-RDCQID**

**Nr2f6 *Xenopus tropicalis*  EQGDEERPGIQVDCVVCGDKSSGKHYGVFTCEGCKSFFKRSVRRNLSYTCRSN-RDCQID**

**NR2F6 *Homo sapiens*  EPGDEERPGLQVDCVVCGDKSSGKHYGVFTCEGCKSFFKRSIRRNLSYTCRSN-RDCQID**

**Nr2f *Trichoplax adhaerens* KANRNQCQFCRLQKCFKVGMRKE--------AVQKERHTSTIRADRN-SGK---------**

**Nr2f1/2/5/6 *Lottia gigantea* QHHRNQCQYCRLRKCLKAGMRRE--------AVQRGRIPPSQHPF---AGQ--------M**

**Nr2f1/2/5/6 *Ciona robusta* QHHRNQCQYCRLNKCVKIGMRRE--------AVQRGRMPPSQPHT---TGQ--------Y**

**Nr2f1/2A *Petromyzon marinus* QHHRNQCQYCRLKKCLKVGMRRE--------AVQRGRLPPSQHPNP----------A-QY**

**Nr2f1/2A *Eptatretus burgeri* QHHRNQCQYCRLKKCLKVGMRREDRSVFLFAAVQRGRLPPTQHPNP----------ALQY**

**Nr2f1/2B *Petromyzon marinus* QHHRNQCQYCRLKKCLKVGMRREAL--VPFPAVQRGRLAPTQHPNP----------ALQY**

**Nr2f1/2B *Eptatretus burgeri* QHHRNQCQYCRLKKCLKVGMRREVSSLF-TAAVQRGRMPPT-QPT---HGQ--------F**

**Nr2f1/2C *Petromyzon marinus* QHHRNQCQYCRLKKCLKVGMRRE--------AVQRGRMPSSMQHPATPGGAYALQPNGGD**

**Nr2f1/2C *Eptatretus burgeri* QHHRNQCQYCRLKKCLKVGMRRE--------AVQRGRLPHMQHPV---PGQ--------Y**

**Nr2f1a *Danio rerio* QHHRNQCQYCRLKKCLKVGMRRE--------AVQRGRMPPTQP-N---PGQ--------Y**

**Nr2f1b *Danio rerio* QHHRNQCQYCRLKKCLKVGMRRE--------AVQRGRMPPN-QPN---PSH--------Y**

**Nr2f1 *Xenopus tropicalis*  QHHRNQCQYCRLKKCLKVGMRRE--------AVQRGRMPPTQP-N---PGQ--------Y**

**Nr2f1 *Latimeria chalumnae*  QHHRNQCQYCRLKKCLKVGMRRE--------AVQRGRMPPTQP-N---PGQ--------Y**

**NR2F1 *Homo sapiens*  QHHRNQCQYCRLKKCLKVGMRRE--------AVQRGRMPPTQP-N---PGQ--------Y**

**Nr2f2 *Danio rerio*  QHHRNQCQYCRLKKCLKVGMRRE--------AVQRGRMPPT-QPH---HGQ--------F**

**Nr2f2 *Latimeria chalumnae*  QHHRNQCQYCRLKKCLKVGMRREVSSLF-TAAVQRGRMPPT-QPT---HGQ--------F**

**Nr2f2 *Xenopus tropicalis*  QHHRNQCQYCRLKKCLKVGMRRE--------AVQRGRMPPT-QPT---HGQ--------F**

**NR2F2 *Homo sapiens*  QHHRNQCQYCRLKKQAVWGMRRE--------AVQRGRMPPT-QPT---HGQ--------F**

**Nr2f5 *Danio rerio*  QHHRNQCQYCRLKKCLKVGMRRE--------AVQRGRMSNSQS----SPGQ--------Y**

**Nr2f5 *Latimeria chalumnae*  QHHRNQCQYCRLKKCLKVGMRRE--------AVQRGRMTHPQT----SPGQ--------Y**

**Nr2f5 *Xenopus tropicalis*  QHHRNQCQYCRLKKCLKVGMRRE--------AVQRGRMSHPQT----SPGQ--------Y**

**Nr2f6a  *Danio rerio*  QHHRNQCQYCRLKKCFRVGMRKE--------AVQRGRIPPSHSSLSPSTTP--VGGNAGG**

**Nr2f6b  *Danio rerio*  QHHRNQCQYCRLKKCFRVGMRKE--------AVQRGRIPPSHAGISPASMV-GAGGDVGG**

**Nr2f6 *Latimeria chalumnae*  QHHRNQCQYCRLKKCFRVGMRKE--------AVQRGRIPLAQSTTSPNSTP-G-------**

**Nr2f6 *Xenopus tropicalis*  QHHRNQCQYCRLKKCFRVGMRKE--------AVQRGRIPPAHSSASPTSAP-GA------**

**NR2F6 *Homo sapiens*  QHHRNQCQYCRLKKCFRVGMRKE--------AVQRGRIPHSLPGAVAASSG-SPPGSALA**

**: .. * ***: ***

**Nr2f *Trichoplax adhaerens* --TEKEMTPDSETAINSLIKNLVAAETLVLSSRSLQL---------QSGFIGFEAICQSS**

**Nr2f1/2/5/6 *Lottia gigantea* GFPNGD-PLGGHGYFSSFITMLLRAEPYPTSRYG-QCMQ--------NNIVGIDSICELA**

**Nr2f1/2/5/6 *Ciona robusta* AITNGVESNFGPGYMSGYISMLLRAEPYPTSRFALQCPVP-------NQIMGIDNICELA**

**Nr2f1/2A *Petromyzon marinus* ALVNGD-PLNGHSYLSGYISLLLRAEPYPTSRYGSQCMQP-------NNIMGIENICELA**

**Nr2f1/2A *Eptatretus burgeri* ALVNGD-PLNGHSYLSGYISLLLRAEPYPTSRYGSQCMQP-------NNIMGIENICELA**

**Nr2f1/2B *Petromyzon marinus* ALVNGD-PLNGHSYLSGYISLLLRAEPYPTSRYGSQCMQP-------NNIMGIENICELA**

**Nr2f1/2B *Eptatretus burgeri* ALTNGD-PLNCHSYLSGYISLLLRAEPYPTSRFGSQCMQP-------NNIMGIENICELA**

**Nr2f1/2C *Petromyzon marinus* PLLNGA-GGHAASYLSGYISLLLRAEPYPTSRYGSQCMQP-------NNLMGIENICELA**

**Nr2f1/2C *Eptatretus burgeri* ALANGD-PLNGHSYLSGYISLLLRAEPYPTSRYGSQCMQP-------NNIMGIENICELA**

**Nr2f1a *Danio rerio* ALTNGD-PLNGHCYLSGYISLLLRAEPYPTSRYGSQCMQP-------NNIMGIENICELA**

**Nr2f1b *Danio rerio* ALTNGD-HLNGQCYLSGYISLLLRAEPYPASRYGNQCMQS-------GNIMGIENICELA**

**Nr2f1 *Xenopus tropicalis*  ALTNGD-PLNGHCYLSGYISLLLRAEPYPTSRYGSQCMQP-------NNIMGIENICELA**

**Nr2f1 *Latimeria chalumnae*  ALTNGD-PLNGHCYLSGYISLLLRAEPYPTSRYGSQCMQP-------NNIMGIENICELA**

**NR2F1 *Homo sapiens*  ALTNGD-PLNGHCYLSGYISLLLRAEPYPTSRYGSQCMQP-------NNIMGIENICELA**

**Nr2f2 *Danio rerio*  ALTNGD-PLHCHSYLSGYISLLLRAEPYPTSRYGSQCMQP-------NNIMGIENICELA**

**Nr2f2 *Latimeria chalumnae*  ALTNGD-PLNCHSYLSGYISLLLRAEPYPTSRFGSQCMQP-------NNIMGIENICELA**

**Nr2f2 *Xenopus tropicalis*  ALTNGD-PLNCHSYLSGYISLLLRAEPYPTSRFGSQCMQP-------NNIMGIENICELA**

**NR2F2 *Homo sapiens*  ALTNGD-PLNCHSYLSGYISLLLRAEPYPTSRFGSQCMQP-------NNIMGIENICELA**

**Nr2f5 *Danio rerio*  LSNGSD-PYNGQPYLSGFISLLLRAEPYPTSRYGAQCMQS-------NNLMGIENICELA**

**Nr2f5 *Latimeria chalumnae*  TLTNSD-QYNGHSYLTGFISMLLRAEPYPMSRYGGQCMQP-------NNFMGIENICELA**

**Nr2f5 *Xenopus tropicalis*  TLNNVD-PYNGHSYLTGFISLLLRAEPYPTSRYGAQCLQP-------NNIMGIENICELA**

**Nr2f6a  *Danio rerio*  G---VSEFYNG-QPVSELISQLLRAEPYPNSRYSHQYNQQMQGGGGGGSGMGIDSICELA**

**Nr2f6b  *Danio rerio*  GPGMGADFFNG-QPVSELISQLLRAEPYPNSRYGAQCGQQLQGA--NSSMMGIDNICELA**

**Nr2f6 *Latimeria chalumnae*  -----GDYFNG-QPVSELIFQLLRAEPYPTARYGSQYTQQ-------NTVMGIDNICELA**

**Nr2f6 *Xenopus tropicalis*  -----GEYFNG-QPVSELISQLLRAEPYPASRYGSQYAQQ-------GSVMGIDNICELA**

**NR2F6 *Homo sapiens*  AVASGGDLFPG-QPVSELIAQLLRAEPYPAAAG-----RFGAGGGAAGAVLGIDNVCELA**

**.. * *: ** : . :*:: :*: :**

**Nr2f *Trichoplax adhaerens* MRILYSVVEWTVKLPYFSEMTSCTDQMTLLRSCWSELFILNAAQWSPPLNMFPYSTTSNF**

**Nr2f1/2/5/6 *Lottia gigantea* ARLLFSAVEWARNVHFFPELH-VNDQVELLRISWSELFVLNAAQSSMPCHLSPLLAAAGL**

**Nr2f1/2/5/6 *Ciona robusta* ARLLFSAVEWARNIPFFPELQ-VTDQVAMLKWVWSELFVLNAAQSHMPLHVAPLLAAAGL**

**Nr2f1/2A *Petromyzon marinus* ARLLFSAVEWARNIPFFPDLQ-ITDQVALLRLVWSELFVLNAAQCAMPLHVAPLLAAAGL**

**Nr2f1/2A *Eptatretus burgeri* ARLLFSAVEWARNIPFFPDLQ-ITDQVALLRLVWSELFVLNAAQCAMPLHVAPLLAAAGL**

**Nr2f1/2B *Petromyzon marinus* ARLLFSAVEWARNIPFFPDLQ-ITDQVALLRLVWSELFVLNAAQCAMPLHVAPLLAAAGL**

**Nr2f1/2B *Eptatretus burgeri* ARMLFSAVEWARNIPFFPDLQ-ITDQVALLRLTWSELFVLNAAQCSMPLHVAPLLAAAGL**

**Nr2f1/2C *Petromyzon marinus* ARLLFSAVEWARNIPFFPELQ-VGDQVALLRLVWSELFVLNAAQCAMPLHVAPLLAAAGL**

**Nr2f1/2C *Eptatretus burgeri* ARLLFSAVEWARNIPFFPELQ-VGDQVALLRLAWSELFVLNAAQCAMPLHVAPLLAAAGL**

**Nr2f1a *Danio rerio* ARLLFSAVEWARNIPFFPDLQ-ITDQVSLLRLTWSELFVLNAAQCSMPLHVAPLLAAAGL**

**Nr2f1b *Danio rerio* ARLLFSAVEWARNIPFFPDLQ-ITDQVSLLRLTWSELFVLNAAQSSMPLHVAPLLAAAGL**

**Nr2f1 *Xenopus tropicalis*  ARLLFSAVEWARNIPFFPDLQ-ITDQVALLRLTWSELFVLNAAQCSMPLHVAPLLAAAGL**

**Nr2f1 *Latimeria chalumnae*  ARLLFSAVEWARNIPFFPDLQ-ITDQVALLRLTWSELFVLNAAQCSMPLHVAPLLAAAGL**

**NR2F1 *Homo sapiens*  ARLLFSAVEWARNIPFFPDLQ-ITDQVSLLRLTWSELFVLNAAQCSMPLHVAPLLAAAGL**

**Nr2f2 *Danio rerio*  ARMLFSAVEWARNIPFFPDLQ-ITDQVALLRLTWSELFVLNAAQCSMPLHVAPLLAAAGL**

**Nr2f2 *Latimeria chalumnae*  ARMLFSAVEWARNIPFFPDLQ-ITDQVALLRLTWSELFVLNAAQCSMPLHVAPLLAAAGL**

**Nr2f2 *Xenopus tropicalis*  ARMLFSAVEWARNIPFFPDLQ-ITDQVALLRLTWSELFVLNAAQCSMPLHVAPLLAAAGL**

**NR2F2 *Homo sapiens*  ARMLFSAVEWARNIPFFPDLQ-ITDQVALLRLTWSELFVLNAAQCSMPLHVAPLLAAAGL**

**Nr2f5 *Danio rerio*  ARLLFSAVEWAKNIPFFPDLQ-LMDQVALLRMSWSELFVLNAAQCSMPLHVAPLLAAAGL**

**Nr2f5 *Latimeria chalumnae*  ARLLFSAIEWAKSIPFFPDLQ-LGDQVSLLRMTWSELFVLNAAQCSMPLHVAPLLAAAGL**

**Nr2f5 *Xenopus tropicalis*  ARLLFSAIEWAKNIPFFPDFQ-LSDQVSLLRMTWSELFVLNAAQCSMPLHVAPLLAAAGL**

**Nr2f6a  *Danio rerio*  ARLLFSIIEWARNIPYFPELP-VSEQVALLRLSWSELFILNAAQSALPLHMAPLLAAAGF**

**Nr2f6b  *Danio rerio*  ARLLFSTIEWARNIPYFPDLP-VSEQVALLRLSWSELFILNAAQSALPLHTAPLLAAAGF**

**Nr2f6 *Latimeria chalumnae*  ARLLFSTVEWARNIPFFPELP-VSDQISLLRLSWSELFVLNAAQSALPLHMAPLLAAAGF**

**Nr2f6 *Xenopus tropicalis*  ARLLFSTVEWSRNIPYFPELA-MADQVSLLRLSWSELFVLSAAQSALPLHMAPLLAAAGF**

**NR2F6 *Homo sapiens*  ARLLFSTVEWARHAPFFPELP-VADQVALLRLSWSELFVLNAAQAALPLHTAPLLAAAGL**

***:*:* :**: :* :: :*: :*: *****:*.*** * : * :::.:**

**Nr2f *Trichoplax adhaerens* Y-----LTHPQEVMHHICLFQEAIVKLKKRFIDTTEFSCLKALILFNPDVRGLVNPNYVE**

**Nr2f1/2/5/6 *Lottia gigantea* HASPMPADHVVAFMENIRTFQDHVEKLKNLHIDTAEYSCLKAIALFSSDSRSLSDINQIE**

**Nr2f1/2/5/6 *Ciona robusta* HTS-MSADRVMTFMDHIRIFQEQVERLKSLHVDSAEYSCLKAIVLFTADSHGLSDMTHIE**

**Nr2f1/2A *Petromyzon marinus* HASPMSADRVVAFMDHIRIFQEQVEKLKALHVDSAEYSCIKAIVLFTTDACGLSDAAHIE**

**Nr2f1/2A *Eptatretus burgeri* HASPMSADRVVAFMDHIRIFQEQVEKLKALHVDSAEYSCLKAIVLFSTDACGLSDAAHIE**

**Nr2f1/2B *Petromyzon marinus* HASPMSADRVVAFMDHIRIFQEQVEKLKALHVDSAEYSCLKAIVLFSTDACGLSDAAHIE**

**Nr2f1/2B *Eptatretus burgeri* HASPMSADRVVAFMDHIRIFQEQVEKLKALHVDSAEYSCLKAIVLFTSDACGLSDVAHVE**

**Nr2f1/2C *Petromyzon marinus* HASPMSAERVVAFMDHIRIFQEQVEKLKALHVDSAEYSCLKAIVLFTSDACGLSDTAHVE**

**Nr2f1/2C *Eptatretus burgeri* HASPMSADRVVAFMDHIRIFQEQVEKLKSLHVDSAEYSCLKAIVLFTSDACGLSDTAHVE**

**Nr2f1a *Danio rerio* HASPMSADRVVAFMDHIRIFQEQVEKLKALHVDSAEYSCLKAIVLFTSDACGLSDAAHIE**

**Nr2f1b *Danio rerio* HASPMSADRVVAFMDHIRIFQEQVEKLKALHVDSAEYSCIKAIVLFTSDACGLSDAAHIE**

**Nr2f1 *Xenopus tropicalis*  HASPMSADRVVAFMDHIRFFQEQVEKLKALQVDSAEYSCAKAIVLFTSDACGLSDIPHIE**

**Nr2f1 *Latimeria chalumnae*  HASPMSADRVVAFMDHIRIFQEQVEKLKALHVDSAEYSCLKAIVLFTSDACGLSDVAHIE**

**NR2F1 *Homo sapiens*  HASPMSADRVVAFMDHIRIFQEQVEKLKALHVDSAEYSCLKAIVLFTSDACGLSDAAHIE**

**Nr2f2 *Danio rerio*  HASPMSADRVVAFMDHIRIFQEQVEKLKALHVDSAEYSCLKAIVLFTSDACGLSDVAHVE**

**Nr2f2 *Latimeria chalumnae*  HASPMSADRVVAFMDHIRIFQEQVEKLKALHVDSAEYSCLKAIVLFTSDACGLSDVAHVE**

**Nr2f2 *Xenopus tropicalis*  HASPMSADRVVAFMDHIRIFQEQVEKLKALHVDSAEYSCLKAIVLFTSDACGLSDVAHVE**

**NR2F2 *Homo sapiens*  HASPMSADRVVAFMDHIRIFQEQVEKLKALHVDSAEYSCLKAIVLFTSDACGLSDVAHVE**

**Nr2f5 *Danio rerio*  HASPMSAERVVAFMDHIRVFQEQVEKLKALQVDTAEYSCLKSIVLFTSDAMGLSDVAHVE**

**Nr2f5 *Latimeria chalumnae*  HASPMSADRVVAFMDHIRVFQEQVEKLKALHVDSAEYSCLKAIVLFTPDAVGVSDLAHVE**

**Nr2f5 *Xenopus tropicalis*  HASPMSADRVVAFMDHIRVFQEQVEKLKALHVDSAEYSCLKAIALFTPDAVGLSDIGHVE**

**Nr2f6a  *Danio rerio*  HSSPMSAERVVSFMDQVRVFQDQVEKLTRLQVDSAEYSCLKAIALFSPDACGLTDPAHVE**

**Nr2f6b  *Danio rerio*  HSSPMPADRVVSFMDQVRVFQDQVDKLTRLQVDSVEYSCLKAIALFSPDACGLSDPAHVE**

**Nr2f6 *Latimeria chalumnae*  HTSPMSADRVVSFMDQIRIFQDQVEKLNRLQVDSAEYSCLKAIALFTPDACGLSDPAHVE**

**Nr2f6 *Xenopus tropicalis*  HASPMSADRVVSFMDQIRLFQDQVEKLNRLQVDSAEYACLKAIALFTSDACGLTDPAHVE**

**NR2F6 *Homo sapiens*  HAAPMAAERAVAFMDQVRAFQEQVDKLGRLQVDSAEYGCLKAIALFTPDACGLSDPAHVE**

**: : .*.:: **: : :* :*:.*:.* *:: **. * .: : :***

**Nr2f *Trichoplax adhaerens* YIQENIQCALKQHVKSQYPDQPSRFGYLLLRLLMLRSISSKVIEEIFFTSVLCRRSIDIF**

**Nr2f1/2/5/6 *Lottia gigantea* SLQERSQCALEEYVRSQYPNQPTRFGKLLLRLPSLRAINSHVIEQLFFVRLVGKTHIETL**

**Nr2f1/2/5/6 *Ciona robusta* SVQEKSQCALEEYVRHQYPNQPSRFGKLLLRLPSLRTVSASAIEQLFFVRLVGKTPIETL**

**Nr2f1/2A *Petromyzon marinus* SLQEKSQCALEEYVRSQYPNQPTRFGKLLLRLPSLRTVSSSVIEQLFFVRLVGKTPIETL**

**Nr2f1/2A *Eptatretus burgeri* SLQEKSQCALEEYVRSQYPNQPTRFGKLLLRLPSLRTVSSSVIEQLFFVRLVGKTPIETL**

**Nr2f1/2B *Petromyzon marinus* NLQEKSQCALEEYVRSQYPNQPTRFGKLLLRLPSLRTVSSSVIEQLFFVRLVGKTPIETL**

**Nr2f1/2B *Eptatretus burgeri* SLQEKSQCALEEYVRSQYPNQPTRFGKLLLRLPSLRTVSSSVIEQLFFVRLVGKTPIETL**

**Nr2f1/2C *Petromyzon marinus* SLQEKSQCALEEYVRTQYPSQPTRFGKLLLRLPSLRTVSSAVIEQLFFVRLVGKTPIETL**

**Nr2f1/2C *Eptatretus burgeri* GLQEKSQCALEEYVRAQYPGQPSRFGRLLLRLPSLRSVSSSVIEQLFFVRLVGKTPIETL**

**Nr2f1a *Danio rerio* SLQEKSQCALEEYVRSQYPNQPSRFGKLLLRLPSLRTVSSSVIEQLFFVRLVGKTPIETL**

**Nr2f1b *Danio rerio* GLQEKSQCALEEYVRSQYPNQPTRFGKLLLRLPALRMVSSSVIEQLFFVRLVGKTPIETL**

**Nr2f1 *Xenopus tropicalis*  SLQEKSQCALEEYVRSQYPNQPSRFGKLLLRLPSLRTVSSSVIEQLFFVRLVGKTPIETL**

**Nr2f1 *Latimeria chalumnae*  SLQEKSQCALEEYVRSQYPNQPSRFGKLLLRLPSLRTVSSSVIEQLFFVRLNLPSSLEQW**

**NR2F1 *Homo sapiens*  SLQEKSQCALEEYVRSQYPNQPSRFGKLLLRLPSLRTVSSSVIEQLFFVRLVGKTPIETL**

**Nr2f2 *Danio rerio*  SLQEKSQCALEEYVRSQYPNQPTRFGKLLLRLPSLRTVSSSVIEQLFFVRLVGKTPIETL**

**Nr2f2 *Latimeria chalumnae*  SLQEKSQCALEEYVRSQYPNQPTRFGKLLLRLPSLRTVSSSVIEQLFFVRLVGKTPIETL**

**Nr2f2 *Xenopus tropicalis*  SLQEKSQCALEEYVRSQYPNQPTRFGKLLLRLPSLRTVSSSVIEQLFFVRLVGKTPIETL**

**NR2F2 *Homo sapiens*  SLQEKSQCALEEYVRSQYPNQPTRFGKLLLRLPSLRTVSSSVIEQLFFVRLVGKTPIETL**

**Nr2f5 *Danio rerio*  SIQEKSQCALEEYVRNQYPNQPNRFGRLLLRLPSLRIVSSPVIEQLFFVRLVGKTPIETL**

**Nr2f5 *Latimeria chalumnae*  SIQEKSQCALEEYVRNQYPNQPSRFGRLLLRLPSLRIVSSPIIEQLFFVRLVGKTPIETL**

**Nr2f5 *Xenopus tropicalis*  SIQEKSQCALEEYVRNQYPNQPTRFGRLLLRLPSLRIVSAPVIEQLFFVRLVGKTPIETL**

**Nr2f6a  *Danio rerio*  SLQEKAQVALTEYERMQYPGQPQRFGRLLLRLPALRAVPASLISQLFFMRLVGKTPIETL**

**Nr2f6b  *Danio rerio*  SLQEKAQVALTEYERMQYPGQPQRFGRLLLRLPALRAVPANLISQLFFMRLVGKTPIETL**

**Nr2f6 *Latimeria chalumnae*  SLQEKAQVALTEYVRSQYPSQPQRFGRLLLRLPALRAVPASLISQLFFMRLVGKTPIETL**

**Nr2f6 *Xenopus tropicalis*  SLQEKAQVALTEYVRAQYPSQPQRFGRLLLRLPALRAVPASLISQLFFMRLVGKTPIETL**

**NR2F6 *Homo sapiens*  SLQEKAQVALTEYVRAQYPSQPQRFGRLLLRLPALRAVPASLISQLFFMRLVGKTPIETL**

**:**. * ** :: : ***.** *** ***** ** : : *.::** : ::**

**Nr2f *Trichoplax adhaerens* LCEAMESVKRA------------------------------------------**

**Nr2f1/2/5/6 *Lottia gigantea* IRDILLS--------------------GNSFSWPYMPIQ--------------**

**Nr2f1/2/5/6 *Ciona robusta* IRDMLLS--------------------GSSYGWPYHMTLQ-------------**

**Nr2f1/2A *Petromyzon marinus* IRDMLLS--------------------GSSFNWPYMPIQ--------------**

**Nr2f1/2A *Eptatretus burgeri* IRDMLLS--------------------GSSFNWPYMPIQ--------------**

**Nr2f1/2B *Petromyzon marinus* IRDMLLS--------------------GSSFNWPYMPIQ--------------**

**Nr2f1/2B *Eptatretus burgeri* IRDMLLS--------------------GSSFNWPYMSIQ--------------**

**Nr2f1/2C *Petromyzon marinus* IRDMLLS--------------------GSSFNWPYMSIQ--------------**

**Nr2f1/2C *Eptatretus burgeri* IRDMLLS--------------------GSSFSWPYMSIQ--------------**

**Nr2f1a *Danio rerio* IRDMLLS--------------------GSSFNWPYMSIQ--------------**

**Nr2f1b *Danio rerio* IRDMLLS--------------------GSSFNWPYMPIQ--------------**

**Nr2f1 *Xenopus tropicalis*  IKSKTTGYKISPVVRGAYLRETRLTAVSDPFVLIYEDVCCV------------**

**Nr2f1 *Latimeria chalumnae*  IRDMLLS--------------------GSSFNWPYMPIQCS------------**

**NR2F1 *Homo sapiens*  IRDMLLS--------------------GSSFNWPYMSIQCS------------**

**Nr2f2 *Danio rerio*  IRDMLLS--------------------GSSFNWPYMSIQ--------------**

**Nr2f2 *Latimeria chalumnae*  IRDMLLS--------------------GSSFNWPYMSIQ--------------**

**Nr2f2 *Xenopus tropicalis*  IRDMLLS--------------------GSSFNWPYMSIQ--------------**

**NR2F2 *Homo sapiens*  IRDMLLS--------------------GSSFNWPYMAIQ--------------**

**Nr2f5 *Danio rerio*  LRDMLLS--------------------GSSYNWPYMPVQRDRPISIHYNENGP**

**Nr2f5 *Latimeria chalumnae*  IRDMLLS--------------------GSSLNWPYMAMQ--------------**

**Nr2f5 *Xenopus tropicalis*  IRDMLLS--------------------GSSFNWPYMPMQ--------------**

**Nr2f6a  *Danio rerio*  IRDMQLS--------------------GSSISWPYAPGQ--------------**

**Nr2f6b  *Danio rerio*  IRDMQLS--------------------GSSISWPYVPGQ--------------**

**Nr2f6 *Latimeria chalumnae*  IRDMLLS--------------------GSTFNWPYVGGQQ-------------**

**Nr2f6 *Xenopus tropicalis*  IRDMLLS--------------------GSSFNWPYSSGQ--------------**

**NR2F6 *Homo sapiens*  IRDMLLS--------------------GSTFNWPYGSGQ--------------**

**: . .**
