# Supplementary material for: Origin and evolutionary landscape of Nr2f transcription factors across Metazoa
Source: PLoS One. 2021 Nov 22;16(11):e0254282. doi: 10.1371/journal.pone.0254282 (PMC8608329; doi:10.1371/journal.pone.0254282)
Supplement: S6 File — Sequences used in analysis with DBDs (yellow) and LBDs (green) domains in metazoan Nr2f proteins indicated. the Zinc-finger motifs within the DBDs are underlined. (DOCX) [file pone.0254282.s010.docx]

**S6 File. Nr2f domain architectures during metazoan evolution.**

>XP_002109806.1_Nr2f_Trichoplax_adhaerens

MSTRSSEYIDSPTAAAKDETKSLSKELC**CLICGDRSNGRHYGVISCEGC**KGFFKRSVRRNMKYACT**CSANACKITKANRNQCQFC**RLQKCFKVGMRKEAVQKERHTSTIRADRNSGKTEKEMTPDSETAINSLIKNLVAAETLVLSSRSLQLQSGFIGFEAICQSSMRILYSVVEWTVKLPYFSEMTSCTDQMTLLRSCWSELFILNAAQWSPPLNMFPYSTTSNFYLTHPQEVMHHICLFQEAIVKLKKRFIDTTEFSCLKALILFNPDVRGLVNPNYVEYIQENIQCALKQHVKSQYPDQPSRFGYLLLRLLMLRSISSKVIEEIFFTSVLCRRSIDIFLCEAMESVKRA

>XP_032233936.1_Nr2f1/2/5/6a_Nematostella_vectensis

MQGVNSSQGSRKFSGCSAESVSMEVAPVTTWTRDSTETPEDSEKNVQVE**CAVCGDKSSGKHYGVFTCEGC**KSFFKRSVRRNLTYT**CRASRDCPIDQHHRNQCQYC**RLKKCLKVGMRREAVQRGRIPAAQTPTQNAALPGINGDGSTNGHSYLSGFIALLLRAEPYPTTRFQQGLNMPCGIMGIENICELAARLLFSAVEWARNIPFFPDLAVTDQVALLRLVWSELFVLNAAQCPMPLQVAPLLATAGIHSNHMSPDRMVSFMDNIRIFQEQVEKLRNLHVDAAEFACLKAIVLFTSDASGLTDPQYIESLQEKTQCALEEYTRNQYPNQPTRFGKLLLRLPSLRSISSSVVEQLFFVRLVGKTPIETLLRDMLLSGTPTTWPYLPCS

>XP_001634378.2_Nr2f1/2/5/6b_Nematostella_vectensis

MSTCTDVPPTQEVTMPKHKTDERVACAVC**GDKSTGKHYGVSTCEGC**KSFFKRTVRNNTNYT**CRGQNTCAIDRNSR**SRCPSCRFQKCLSTGMKKEAVQTTKLPPFPALQFPFYGDVNTMYAQTMFPLTLFQSPFNAPLTFPMGMVPLNQNRTDVAYELAANVLFAVVDWARKLTTFNNLMDSDQITLLKMAWTDLFLLEASRSPLQLYVQQMYATINAQTKQLSMEVIVKRMEYARLFQEQAERIRNLGMDMTEHFHLKCIVLFRADGSLINQPRQVEVLQDTSQSSLEQYIRSQYPSQPTRFGKLLLMLSSLRKVESTVIEQLFFADVLRGASMGEVLKKMLTTGNQSPTTLAAALANGKGSPMS

>XP_032239605.1_Nr2f1/2/5/6c_Nematostella_vectensis

MNHLDDPLSDSNLGISVFDHGSTYSEPYEIPSIVSSCAESTGSLPSPDTVESSHPSEDLSDNDPDSPASSSSKPLYID**CAVCGDKSSGKHYGVYTCEGC**KSFFKRSIRRSLSYS**CRGVRNCPVDIQNRNQCQYC**RLKKCLKVGMRKEAVQKGRIPSTHPDVGPLSVSMVEMNGHQSFYSSYITLLLRADTIARYQQSLTLPCNINGLENTPELAARLLVSAVEWAKNIPFYSDLPLPDQAVLLRSCWSELFTLNAAQHCSPFHISPTLTSNSSGFAGNGGGYLNTRVMSAFDCQNNNMKLFEEQVEKLKNMHIDSAEFACLKAIVLFNPDSQGLSEPAQVENLQDRTQSALEDYIRTQYPNQTTRFGKLLLRLPALRLLRPVSVENLFFSRLSMGNTVDSLLNDMLLSGLGGGVVPWLPGPSPPLNCTTSNMNVITQM

>XP_001636010.1_Nr2f1/2/5/6d_Nematostella_vectensis

MSSWSQMHEVYWYDNPDKTIE**CAVCSAPSSGRHYGVFTCEGC**KCFFNRTVRYKLTYI**CEGSGSCRVDKQNRTQCQAC**RFKKCATVGMRREAIRRGRPTKYSYISRSSKSFTPQYDLISVLTQLERSIRPPVPYPSSGLQSSPVSMYHRVCSILVSTLDWSRRVPMFANLDVCEQYSVLRSRWCEMLIVSAAQYEVHIDGIPLAYEVEMNPGFCNEKQIQLKRSLRNFQESVWRLRGLEEAEYACLKTIILFSPDASEGPFVQEFESLQELVLSALDRFCRARFPEEPSRYGKVLLKLMSLKSVIAEDIETLVFSKLFPHSSVSGIIRNHLVSDVTSAPESMSPAKTSPVNQ

>XP_029186020.1_Nr2f1/2/5/6a_Acropora_millepora

MAGVPAQSWCTEKTEPLEESAEKNVQQVE**CAVCGDKSSGKHYGVFTCEGC**KSFFKRSVRRNLSYT**CRASRNCPIDQHHRNQCQYC**RLRKCMKVGMRREGTAVQRGRIPPTQVPQPSPQHSALNGNDVSNGHSFLSGFISLLLRAEPYPTTRFQQGMNMPCGIMGIENICELAARLLFSAVEWARNIPFFPDLAVTDQVALLRLVWSELFVLNAAQCPMPLQVAPLLASAGIHSNHMSPDRMVTFMDNVRIFQEQIEKYRNLHVDAAEFACLKAIVLFTSDASGLTDPQYIESLQEKTQCALEEYTRNQYPNQPTRFGKLLLRLPSLRSINSSIVEQLFFVRLVGKTPIDTLLRDMLLSGTPTSWPYLPCS

>XP_029186102.1_Nr2f1/2/5/6b_Acropora_millepora

MNCFNDDTTSTIEVNIRALTDFPQTQNPTAGFSNEENSPVLLETAFYSPEECSESEESSSASKPSIE**CVVCGDKSSGKHYGVFTCEGC**KSFFKRSIRRNLSYT**CRGFKNCPVDIQHRNHCQYC**RLKKCMKVGMRKDAVQKGRISTTQSDVTNSLQLSLGDMRNPQSFYSSYITLLLRADTIARYQQSIGIPTNFAGLENASELGARLLVSIAEWAKSIPFFSEFTLPDQSILLRSCWSELFILSAAQHCSPFHTAQPLTINALTPSSNVDELVASSSGTMLFDSAENLRLFEEQVEKLKNLQIDSAEFCCLKAIILFNPDSQGLSSYAHVEGLQERSQCALEDYIRTQYPNQPTRFGKLLLRLPSLRLIRPGTVEVLFFPPVGLGSTVESAVNEMLVISSSPQVAATSPNNWPATPVFPNCFTAGNMSEMNDMNSMTNSNMNGGAVL

>XP_029186447.1_Nr2f1/2/5/6c_Acropora_millepora

MTACTDSTITELPKLKTEDKLD**CAVCGDKSTGKHYGVSTCEGC**KSFFKRTVRNSTSYT**CRGNNNCTVDRDNRSRCPSC**RFQKCLSTGMKKEAVQTTKLPAFPGFSFPYLNDLSGFYSPGLLPFTLFQPAMSNQGSIHQGMLPQSQGTSEVVYELAAHVLFSVVDWARKLPTFNNLVESDQITLLKMAWSDLYLLESARSPIHIYMPQLYATGNMQMRQLSMENILKRMEHARLFQDQLEKIRSLGMDLTEHFHIKCVVLFRTDGSPITQPQQIEVLQDTSQSSLEQYIRTQRSNQPTRFGKLLLMLSSLRKVEPTVIEQLFFAEVLRGASMGDVLKKMLTSNSSAPTVHGSLMA

>XP_029191520.1_Nr2f1/2/5/6d_Acropora_millepora

MEI**CSVCGDHSTGRHYGANTCEGC**KLFFKRSIKKQLYYT**CRVTGCCPVNKRYRNSCQYC**RMKKCLRVGMKREAVQQTRAQDFEKGRRRTRRHQTKSDIRRETDFVSLSDFVCHLQAVEPYQTPRTSKNEARSTSEKSDAYVVDSANAAPKLAVSENTTEIAARLLFMSIHWAKDVRHFSELSHFDQVVLLQENWYKLFVINLVQWAMPFEIAPLVADVVEKTPSQHLDGFLHNLGKLNEVVCKFLHLELDRAEFCLIKALALFNPDIDQLTDAVQVQAVQNKTRNALEEYLRMHRAHAPNRLGQLLIKLTSICAVDPKMLEHVFFNKLIGGAAINGLVDDILRANYLSSHSNAKSTTTV

>AEB21388.1_Nr2f1/2/5/6c_Hydractinia_echinata TNKHTLVD**CAVCGDKSSGKHYGVYTCEGC**KSFFKRSIRRNLEYQ**CRSNKKCPVDQHHRNQCQHC**RLKKCFKVGMRREAVQSGRHKVQANTQSSASSSFSSTFLSQLIAAEPFQPVLSPISMNFMNDGSQNMCELATKLLFNAIEWAKNIPIFPTLTTGDQIALIKLGWKELFVLNLGKCQSPLRLNDVLSNSNLDGLPEYQATFYEHVKALQTQIDTLKSLQIDAAEYACLKAMILFTPDTPSLNNSAYIHTLQEKAMNALESYTKTKYPLQPARFGKLLLRLSSIRPINTVVIEQLFFTWLVGKTPIETIIQDYMFGAPNQSYQWACLKEIHLLEKGE

>XP_004210599.1_Nr2f1/2/5/6b_Hydra_vulgaris

MASVQPTDGNIFRIARNLNVDYKSSSNVLSEKNFQLSFDCIRPTMDPTLQNPMKNSLADTNIADSDSKIIY**CAVCSDRSTGKHYKVNSCEGC**KNFFRRSVRKNVKYV**CPAYGKCLVHKDQRTRCKAC**RLKKCLKVGMRKEAVQCERKPAALVITSFKDGMGLKNKTVGLDYLTSSEDSEEENLTYTQLNKTTKNEIFQCIEEKDVNNYLQAEIEKDSKVSFKRTNSPNNKVNETKQNNNIEEIEVLNQSTNKVDRSDRFSPKKSSNKVENVRLLDSYKNLGAKNEGEFPPIIFKSGQYKSFYVEPPSIPTTDVGYLYEMATRLLFVTIDWVQHLIAFRKLNKTDQLNLLVDKWYSLFILGLAQCSSMFPISTLLFLANNSHDGIKPMLSWQTFSKLKEVILNGNSAIKTSTKIYDNMKIITLFDSDTPGLNDRNAVSYTRCQIQEELEYIINDLQHNEKIENIRQQIICFLESVNNVEKNEITDTFFVPILRETTIEFVIQKLLAQKLR

>XP_002168741.3_Nr2f1/2/5/6c_Hydra_vulgaris

MSSNASVSHNNQNNNSSFDHANGYMSIIQEVKSKPI**DCGVCGDKSSGKHYGVYTCEGC**KSFFKRSIRRNLAYT**CRAFQNCSIDLNHRNQCQYCRLKKC**VKVGMRKDVQKGRLPKSPLEGSMVDQFIAMNNNVSHYNGLHQIANYSNVLSARLQQQQQNQYLSQNNLFGGDSIYEMATRLLFNAVEWARNVPFFSALPTSDQIALLKSSWSELYILSTSQHCIAFQINARALTSEQNLSEIKKRSEGANEASVKMFEELVERFKNLQTDAAEFSCLKALVLFNPDSPGLGNPSLIENLQEKAQSALEDYLRQQNATQSYHNRFGKLLLRLPALSLIRPATIEALFFPRHHGSQNIDSLVGSMLLYGLGSNNNTSFVNNGIPAVGLNGGSNGSILNMTGLAGHVTSGFINNANLAFSNANNLSSLQMPIQDNIQNLCNPNMFSATGINPVNGLNASNGYGHHVINNMMNQHSVANRNSQNNSCVQSMQQNVQMHLSPTTRHHIKIEPPLR

>XP_012567198.1_Nr2f1/2/5/6d_Hydra_vulgaris

MAVNAPLDLKSYLRYVDDSHARFSNAQEAEQFLIILNKQHPPIQYTIEIESENRTLNFLDLTIVNNTKGKYEFKVYRKNAITNIQIKPHSNHDPKILNAIFKGYVHRAYSICSDLYLEDEINFIFHVFKENGYNICQLTRIANLIRNKRSIKSNKIQSGTLNLLTLINPVTLQLENFITQSDKKNEWNSILKVKNGEKKTCVID**CGVCGDKSSGKHYGVNTCEGC**KSFFKRSVRRNLQYT**CRAKRNCSIDQHHRNQCQHCRL**KKCLKAGMRKDAVQRGRLNSQQGAAQVFEDATVNNFSFLSGFVTLLLRAEPCPIFRYSQGVSNNPQFDFIDIDNYELAARLLFNAVEWSRNIPFFPNLSLTDQIALLRLCWKELFILNVAQCPMLIDVSHLLNSQMNIYASPEHMASFLDQVRILKEQLNKLRAMHVDPAEFACLKAIVVFSSDAPGLNDPQYIETLQEKTYFALEDYIKTQYPLQPTRFGKLLLRFPSIRIISATVIEQLFFVRLVGKTPIETLIRDILISGTSYDWPTLNYSERT

>NP_001262499.1_Nr2f1/2/5/6_Drosophila_melanogaster

MCASPSTAPGFFNPRPQSGAELSAFDIGLSRSMGLGVPPHSAWHEPPASLGGHLHAASAGPGTTTGSVATGGGGTTPSSVASQQSAVIKQDLSCPSLNQAGSGHHPGIKEDLSSSLPSANGGSAGGHHSGSGSGSGSGVNPGHGSDMLPLIKGHGQDMLTSIKGQPTGCGSTTPSSQANSSHSQSSNSGSQIDSKQNIE**CVVCGDKSSGKHYGQFTCEGC**KSFFKRSVRRNLTYS**CRGSRNCPIDQHHRNQCQYC**RLKKCLKMGMRREAVQRGRVPPTQPGLAGMHGQYQIANGDPMGIAGFNGHSYLSSYISLLLRAEPYPTSRYGQCMQPNNIMGIDNICELAARLLFSAVEWAKNIPFFPELQVTDQVALLRLVWSELFVLNASQCSMPLHVAPLLAAAGLHASPMAADRVVAFMDHIRIFQEQVEKLKALHVDSAEYSCLKAIVLFTTDACGLSDVTHIESLQEKSQCALEEYCRTQYPNQPTRFGKLLLRLPSLRTVSSQVIEQLFFVRLVGKTPIETLIRDMLLSGNSFSWPYLPSMXHTMWRQLTTT

>AGAP002544-PA.3_Nr2f1/2/5/6_Anopheles_gambiae

MYGGGVLCPSPSTATGFYNPRGQGSEIGALELGFPRGMALVPPPPHGAWRDPSSLTGTHLPVSSSTASAAGDDITTLGSQGGGPGPGQTGSVVVPGMQTSSSSSSGGGAQDKKDFISSSSGGSSIGSVGQHPSVTGQGNTIEKKEFLSLQQSTTPGSQSMNSQNGQDLKNQNIE**CVVCGDKSSGKHYGQFTCEGC**KSFFKRSVRRNLTYS**CRGNRNCPIDQHHR**NQCQFCRLRKCLKMGMRREAVQRGRVPPSQPPGIPYGQYSIPNGDTVTGFNGHSYLSSYISLLLRAEPYPTSRYGQCMQTNNIMGIDNICELAARLLFSAVEWARNIPFFPDLQVTDQVALLRLVWSELFVLNASQCSMPLHVAPLLAAAGLHASPMAADRVVAFMDHIRIFQEQVEKLKALHVDSAEYSCLKAIVLFTTDACGLSDVAHIESLQEKSQCALEEYCRSQYPNQPTRFGKLLLRLPSLRTVSSQVIEQLFFVRLVGKTPIETLIRDMLLSGSSFSWPYLPSM

>XP_014681927.1_Nr2f1/2/5/6_Priapulus_caudatus

MAMTVSNAVNPGWRDPTQPTLLPSYTTTTTPATAPPVPAAPTPSQTHELDVKIDMNTTSSTGSVQSTNSDKSSQHIECTVC**GDKSSGKHYGQFTCEGC**KSFFKRSVRRNLTYT**CRGSRNCPIDQHHRNQCQYC**RLKKCLKVGMRREAVQRGRVPPTQPFPGMALTNGELLNGHTYLSSYISLLLRAEPYPTSRYGQCMQPNGIMGIDNICELAARLLFSAVEWARNIPFFPDLQITDQVALLRLVWSELFVLNASQCSMPLHVAPLLAAAGLHTSPMAADRVVAFMDHIRIFQEQVEKLKSLHVDSAEYSCLKAVVLFTTDACGLSDQAHIESLQEKSQCALEEYCRTQYPNQPTRFGKLLLRLPSLRTVSSSVIEQLFFVRLVGKTPIETLIRDMLLSGSSFNWPYMAIQ

>KZS13048_Nr2f1/2/5/6_Daphnia_magna

MGIGGGGVGGLGVGNDGGLNGSHGYLSSYISLLLRAEPYPTSRYGQCMQPTNNIMGIDNICELAARLLFSAVEWARNIPFFPDLQVTDQVALLRLVWSELFVLNASQCSMPLHVAPLLAAAGLHASPMAADRVVAFMDHIRIFQEQVEKLKALHVDSAEYSCLKAIVLFTTDACGLSDVGHIESLQEKSQCALEEYCRTQYANQPVRFGKLLLRLPSLRTVSSQVIEQLFFVRLVGKTPIETLIRDMLLSGSSFNWPYMPSM

>NP_001122483.2_Nr2f1/2/5/6_Caenorhabditis_elegans

MQDGSSGAASLGNSSPDATD**CVVCGDKSSGKHYGQFTCEGC**KSFFKRSIRRSLSYT**CRATKNCAIDVQHRNQCQYC**RLTKCIRMGMRKEVQRGRLPVTMPSLFPPNMFLRSPFPFMSVPFNPLMTAQFTKPSIKESIFEFAAQTIFATVNWARTSMSNLVKGDQLILLRHSWTPIFIFALAQSNFALNLSTHLTAVTATAASTENGSSSLGSKSEDEEKSEEKPERVFDEPQFQGFQAKIDKIRDFHLDVVESSSLRAVLLFSCDEEALEEKGKIEEIVEKLKSAVDEYCKMNKRSERYHQICECLQLLKSTRNLPISRLFFSRLLGTTPLETILSDLLITPPPPTLPFFPQLPSRN

>Bm4429_Nr2f1/2/5/6_Brugia_malayi

MKKKQWKEEHRVPPMEMSVPVVSSSWMDSSIKNPSTSNPDANAQESRSSSGASSSDSNSGVASGSGGAVHPDNSPIDKQLLLGTE**CVVCGDKSSGKHYGQFSCEGC**KSFFKRSIRRSLNYT**CRGSKNCPVDVNHRNQCQYC**RLKKCERMGMRKEAVQRGRIPPNAQHAYSSTVLFGEQLLTVNQSVGSHVSSIVTHLIHAEPYPPIACSSSSSSIGMDNIYEFGAKLLFSAVEWAKNIPFFNELSDTDQLTLLRASWAELFVINAAQFGMPVHVAPLLAASGLHSSPPLPTDQLVVFMDRIRVFQGQIERLKALQMDLAEFCSLKAVILFSVDCCGLNDVIRVETIQEKVQSALEEYCRTQKQLQVGRFGRLLLRLPSLRSISASVIEQLFFVKLVGETPIEFLLRDMLRTQNENVIKPFVWPCQLRS

>OVOC635_Nr2f1/2/5/6_Onchocerca_volvulus

MKRKQWKEEHRVPAVEMSVPVVSSSWMNSSIRNPSTSNPDNNIQDSRSSSGASSSDSGNASNSCVAGGSSGGIHAENSPTDKQLLLGTE**CVVCGDKSSGKHYGQFSCEGC**KSFFKRSIRRSLNYT**CRGSKNCPVDVNHRNQCQYC**RLKKCERMGMRKEAVQRGRIPPNTQYAYSSAVLFGERLVAVNQDVGLHFSSVLTHLIHAEPYPPTACKCFISSSSSNSIGMDNIYEFGAKLLFSVVEWAKSIPFFNELSDTDQLTLLRASWAELFVMNAAQFGMPVHVAPLLAASGLHSFPLPTNQLVVFMDRIRVFQGQIERLKALQMDPTEFCSLKAVILFSIDCCGLNDVIRVETIQEKVQSALEEYCRTQKQLQIGRFGRLLLRLPSLRSISASVIEQLFFVKLVGETPIEFLLRDMLGTQNENVIKPFIWPCQLRS

>CapteP171549_Nr2f1/2/5/6_Capitella_teleta

MALAVANQPNTVPTWGGHSAEDLAGTTCTTPQTTGPPPAVHAQPTAGVTAPTSQPPQPPPSIVTDSTSTPTSNNNNNSPGSNSDKTAQTIECIVC**GDKSSGKHYGQFTCEGC**KSFFKRSVRRNLTYS**CRGNRNCPIDQHHRNQCQYC**RFKKCLKSGMRREAVQRGRVPPTQHPGFPGQLALANTDPFSGHTYLSSFISMLLRAEPYPMSRYGQCMQPNNIMGIENICELAARLLFSAVEWGRNIPFFPDLQVADQVALLRLAWSELFVLNAAQCAMPLHVAPLLAAAGLHASPMAADRVVAFMDHIRIFQEQVEKLKALHVDSAEYSCLKAIVLFSSDACGLSDSAHIESLQEKAQCALEEYDRSQYPNQPTRFGKLLLRLPSLRSVSAQVIEQLFFVRLVGKTPIETLIRDMLLSGGSFNWPYMPIQ

>g9418.t1_Nr2f1/2/5/6_Lingula_anatina

MAMTPVSQTTPVVNTWHDPSEELNSPTQQQPPPPVPVNTPGSQTPSGENKQQHIE**CVVCGDKSSGKHYGQFTCEGC**KSFFKRSVRRNLTYT**CRGSRNCPIDQHHRNQCQYC**RLKKCLKSGMRREAVQRGRIPPTQHPGFPGQLALTNGDPLNGHTYLSSFISMLLRAEPYPTSRYGQCMQPNNIMGIENICELAARLLFSAVEWARNIPFFPDLQVTDQVALLRLCWSELFVLNAAQCNMPLHVAPLLAAAGLHASPMAADRVVAFMDHIRIFQEQVEKLKALHVDSAEYSCLKAIVLFSSDACGLSDQGHIESLQEKSQCALEEYVRSQYPNQPTRFGKLLLRLPSLRTVSAQVIEQLFFVRLVGKTPIETLIRDMLLSGGSFNWPYMAIQ

>LotgiP126514_Nr2f1/2/5/6_Lottia_gigantea

MTPTQPTVTSWRDHTDDLVGTTPTVVPPPLPPPNPTVTPTQTQIPTATTPTPQQNGGSPNSETNNNNKQHIE**CVVCGDKSSGKHYGQYTCEGC**KSFFKRSVRRNLNYT**CRGNKNCPIDQHHRNQCQYC**RLRKCLKAGMRREAVQRGRIPPSQHPFAGQMGFPNGDPLGGHGYFSSFITMLLRAEPYPTSRYGQCMQNNIVGIDSICELAARLLFSAVEWARNVHFFPELHVNDQVELLRISWSELFVLNAAQSSMPCHLSPLLAAAGLHASPMPADHVVAFMENIRTFQDHVEKLKNLHIDTAEYSCLKAIALFSSDSRSLSDINQIESLQERSQCALEEYVRSQYPNQPTRFGKLLLRLPSLRAINSHVIEQLFFVRLVGKTHIETLIRDILLSGNSFSWPYMPIQ

>XP_033749784.1_Nr2f1/2/5/6_Pecten_maximus

MAMTASQGNPVNVTSWPRDPTEDLGTTPPSAVQPPAQPPPVTPTAVTPTQPPPQTQPANVPNGTATTPNGTSSSPEAKQQHIE**CVVCGDKSSGKHYGQFTCEGC**KSFFKRSVRRNLTYT**CRGNRTCPIDQHHRNQCQYC**RLKKCLKMGMRREAVQRGRIPPTQHPFPGQMAFGSGDPLNGHTYLSSFISMLLRAEPYPTSRYGQCMQTNNIMGIENICELAARLLFSAVEWARNIPFFPDLQVTDQVALLRLSWSELFVLNAAQCSMPLHVAPLLAAAGLHASPMAADRVVAFMDHIRIFQEQVEKLKALHVDSAEYSCLKAIVLFSSDACGLSDANHVESIQEKSQCALEEYVRSQYPNQPTRFGKLLLRLPSLRTVSAQVIEQLFFVRLVGKTPIETLIRDMLLSGGSFNWPYMAIQ

>Ocbimv22029145m.p_Nr2f1/2/5/6_Octopus_bimaculoides

VCSTAEVIASRSLNKHANPSSGAKTAVQRGRVPPTQHPGFPGQMALTNGDPLNGHTYLSSFISMLIRAEPYPTSRYGQCMQPNNIMGIENICELAARLLFSAVEWARNIPFFPDLQVADQVALLRLSWSELFVLNAAQCSMPLHVAPLLAAAGLHASPMAADRVVAFMDHIRIFQEQVEKLKALHVDSAEYSCLKAIVLFSSGVVTSSTTTTIIIIIIINDSNNYNNTNKLHNMQGNRIQNIPPLSSTRVCVIA

>NP_001158369.1_Nr2f1/2/5/6_Saccoglossus_kowalevskii

MAMAVSTWRDPNEDLSADKQQHQQQPPPPHQQQQPPPPTQPQPQQQPPQQTPTTPQQTPPAPHQTITPQQTSTPQQPPQQTATPQQTTTPQQTASQQQHIE**CVVCGDKSSGKHYGQFTCEGC**KSFFKRSVRRNLTYT**CRANRNCPIDQHHRNQCQYC**RLKKCLKMGMRREAVQRGRIPPTQPVPGQYALTDGRFDGHSYLSGFISLLLRAEPYPTSRYGAQCMQSNMMGIDGICELAARLLFSAVEWARNIPFFPDLQVTDQVALLRMVWSELFVLNASQCSMPLHVAPLLAASGLHTSPMSADRVVAFMDHIRIFQEQVEKLKALHVDSAEYSCLKAIVLFTSDACGLSDAAHIENLQEKSQCALEEYVRSQYPNQPNRFGKLLLRLPSLRTVSSQVIEQLFFVRLVGKTPIETLIRDMLLSGSSFNWPYMAIQ

>XP_003724162.2_Nr2f1/2/5/6_Strongylocentrotus_purpuratus

MNCQKAPGIGMAQWTDRWSWNELFKPVPVPLAGDRFPQRHRSMSMAVNSWRNEDHLTADKHHGPPQQTGLEPSPLDPCPPDTDHYADTGPLRLPPPPPQTQPPHSAAGPPPPQVQPPQAPIGPPPPSGALQAATPQQTPATPTQTGPGSQGGGGAAQNGPQSGSDSGSNVTSNNNNNSQHIE**CVVCHDKSSGKHYGQFTCEGC**KSFFKRSVRRNLTYS**CRANRNCPIDQHHRNQCQYC**RLKKCLKMGMRREAVQRGRMPPTQPGPGQYLDGRFEGHTFLSGYISLLLRAEPYPTSRYAQCMQTNSVMGIDNICELAARLLFSAVEWARNIPFFPDLQVTDQVALLRMCWSELFVLNASQCSMPLHVAPLLAASGLHASPMSADRVVAFMDHIRIFQEQVEKLKALHVDSAEYSCIKAIVLFTSDACGLSDAAHIEALQEKSQCALEEYVRSQYPNQPNRFGKLLLRLPSLRTVSSHVIEQLFFVRLVGKTPIETLIRDMLLSGSSFSWPYMTMQ

>KH.L17.15_Nr2f1/2/5/6_Ciona_robusta

MAMVVTSWRESNHHDDVTVLQPANHEAVSTPLPPHPQPPPDLTPQTPTTPTNDVTIPDCSTSRPTGNTSSTSEKPQIE**CVVCGDKSSGKHYGQYTCEGC**KSFFKRSVRRNLSYT**CRGNRNCPIDQHHRNQCQYC**RLNKCVKIGMRREAVQRGRMPPSQPHTTGQYAITNGVESNFGPGYMSGYISMLLRAEPYPTSRFALQCPVPNQIMGIDNICELAARLLFSAVEWARNIPFFPELQVTDQVAMLKWVWSELFVLNAAQSHMPLHVAPLLAAAGLHTSMSADRVMTFMDHIRIFQEQVERLKSLHVDSAEYSCLKAIVLFTADSHGLSDMTHIESVQEKSQCALEEYVRHQYPNQPSRFGKLLLRLPSLRTVSASAIEQLFFVRLVGKTPIETLIRDMLLSGSSYGWPYHMTLQ

>phmamm.CG.MTP2014.S347.g07625.01.p_Nr2f1/2/5/6_Phallusia_mammillata

MGFGPGYMSGYISMLLRAEPYPTSRFALQCPVPNQIMGIDNICELAARLLFSAVEWARNIPFFPDLQVTDQVAMLKWVWSELFVLNAAQSHMPLHVAPLLAAAGLHTSMSADRVMSFMDHIRIFQDQVERLKALHVDSAEYSCLKAIVLFTSGLCFICPCINNVEDVQSHICGSRLTGSNSRHFDTSTSCW

>CBY09835.1_Nr2f1/2/5/6_Oikopleura_dioica

MAMVAPNVWREPTSVTSVADSVTGLEIPSLTNYKVESLESVSFEHSSSGPVLRDLDQNQNVTVSFASPTIPTSPNTPGGSSSDSSLTSVVKQEPGGNSTSTSDKSHIE**CVVCGDKSSGKHYGQFTCEGC**KSFFKRSVRRNLNYT**CRGNRSCPIDQHHRNQCQYC**RLKKCLKVGMRREAVQRGRMPPGQNLGQFALPGPDFLGSGPGYLSGFISLLLRAEPYPRYNPTSGLMGVESVCELAARLLFCAVEWARQIPFFPELQITDQVNMLKHCWPELFVLNAAQSHMPLHVAPLLAAAGFHNSADKVMNFMDQVRVFQEQVEKLKSLHIDSAEFTCLKAIVLLSPDAPGVSDPAHVESIQEKAQCALEEYTRCQYPGQPSRFGRLLLRLPSVRTVSPHVIEQLFFVRLVGKTPIETLIRDMLLSGSPTPTSFQPPPTFQTPPPVPAPVPAQVPYPIVTSSFSDLLDTNPVVTSSSFLSMPVPFQLQVPPNA

>XP_019624146.1_Nr2f1/2/5/6_Branchiostoma_belcheri

MAMAVSTWREPGEDLSSPDKQQQQPAPVPGPGPQQPPPSQPPGQTQQSTPGPQQPGPPTPNQQTPNGGSMPQQTPTAPTQPSSQQGQQQSTPTSGSQSQHIE**CVVCGDKSSGKHYGQFTCEGC**KSFFKRSVRRNLTYS**CRGNRTCPIDQHHRNQCQYC**RLKKCLKMGMRREAVQRGRIPPTQHPAGGQYALTNGVDPINGHSYLSGYISLLLRAEPYPTSRYGTQCMQPNNIMGIDNICELAARLLFSAVEWARNIPYFPELQVTDQVALLRLVWSELFVLNASQCSMPLHVAPLLAAAGLHASPMSADRVVAFMDHIRIFQEQVEKLKALHVDSAEYSCLKAIVLFTSDACGLSDAAHIDSLQEKSQCALEEYVRSQYPNQPTRFGKLLLRLPSLRTVSSSVIEQLFFVRLVGKTPIETLIRDMLLSGSSFNWPYMSIQ

>ENSEBUP00000001687.1_Nr2f1/2C_Eptatretus_burgeri

MAMVVSATWRDPLDNSPAPGAAAPIAQSVPGAASVGASQGSSGALSAPSSASSSSSASSTASSAPGDKTPLQHIE**CVVCGDKSSGKHYGQFTCEGC**KSFFKRSVRRNLSYT**CRASRNCPIDQHHRNQCQYC**RLKKCLKVGMRREAVQRGRLPHMQHPVPGQYALANGDPLNGHSYLSGYISLLLRAEPYPTSRYGSQCMQPNNIMGIENICELAARLLFSAVEWARNIPFFPELQVGDQVALLRLAWSELFVLNAAQCAMPLHVAPLLAAAGLHASPMSADRVVAFMDHIRIFQEQVEKLKSLHVDSAEYSCLKAIVLFTSDACGLSDTAHVEGLQEKSQCALEEYVRAQYPGQPSRFGRLLLRLPSLRSVSSSVIEQLFFVRLVGKTPIETLIRDMLLSGSSFSWPYMSIQ

>ENSEBUP00000018705.1_Nr2f1/2B_Eptatretus_burgeri

MAMVVSAWRDPQDDVAGAQGTQPSQAPPGQGPPTGAPHTPQTPVQVGPPTTPAQSNQTNQPNQQNQVEKQQQHIE**CVVCGDKSSGKHYGQFTCEGC**KSFFKRSVRRNLSYT**CRANRNCPIDQHHRNQCQYC**RLKKCLKVGMRREVSSLFTAAVQRGRMPPTQPTHGQFALTNGDPLNCHSYLSGYISLLLRAEPYPTSRFGSQCMQPNNIMGIENICELAARMLFSAVEWARNIPFFPDLQITDQVALLRLTWSELFVLNAAQCSMPLHVAPLLAAAGLHASPMSADRVVAFMDHIRIFQEQVEKLKALHVDSAEYSCLKAIVLFTSDACGLSDVAHVESLQEKSQCALEEYVRSQYPNQPTRFGKLLLRLPSLRTVSSSVIEQLFFVRLVGKTPIETLIRDMLLSGSSFNWPYMSIQ

>ENSEBUP00000011135.1_Nr2f1/2A_Eptatretus_burgeri

MAMVVGPWRDPQDDLTSGQNGPSQAVQPPLAPGGPQTPQTPSQSGPPTTPGQGSQQGDKQQQPNVE**CVVCGDKSSGKHYGQFTCEGC**KSFFKRSVRRNLTYT**CRANRNCPIDQHHRNQCQYC**RLKKCLKVGMRREDRSVFLFAAVQRGRLPPTQHPNPALQYALVNGDPLNGHSYLSGYISLLLRAEPYPTSRYGSQCMQPNNIMGIENICELAARLLFSAVEWARNIPFFPDLQITDQVALLRLVWSELFVLNAAQCAMPLHVAPLLAAAGLHASPMSADRVVAFMDHIRIFQEQVEKLKALHVDSAEYSCLKAIVLFSTDACGLSDAAHIESLQEKSQCALEEYVRSQYPNQPTRFGKLLLRLPSLRTVSSSVIEQLFFVRLVGKTPIETLIRDMLLSGSSFNWPYMPIQ

>XP_032809125.1_Nr2f1/2A_Petromyzon_marinus

MSLDSLPHFYDALRSPTSPQAMAMVVGPWRDPQGDLTSPPNVPGQAGQAPLAPAGPQTPQTPSQSGGPPTTPGQGSTQGDKQQQQQQPNVE**CVVCGDKSSGKHYGQFTCEGC**KSFFKRSVRRNLTYT**CRANRNCPIDQHHRNQCQYC**RLKKCLKVGMRREAVQRGRLPPSQHPNPAQYALVNGDPLNGHSYLSGYISLLLRAEPYPTSRYGSQCMQPNNIMGIENICELAARLLFSAVEWARNIPFFPDLQITDQVALLRLVWSELFVLNAAQCAMPLHVAPLLAAAGLHASPMSADRVVAFMDHIRIFQEQVEKLKALHVDSAEYSCIKAIVLFTTDACGLSDAAHIESLQEKSQCALEEYVRSQYPNQPTRFGKLLLRLPSLRTVSSSVIEQLFFVRLVGKTPIETLIRDMLLSGSSFNWPYMPIQ

>XP_032824766.1_Nr2f1/2B_Petromyzon_marinus

MEAYGHTGDDMGRHLQLARLATYGAKEIQHTYQASPRRAMAMVVGPWRDPQDEMAGPPSQTGQPPLAPAAGPHTPQTPGHAGPPPTTPGQGSTQGDKQQSNVE**CVVCGDKSSGKHYGQFTCEGC**KSFFKRSVRRNLTYT**CRANRNCPIDQHHRNQCQYC**RLKKCLKVGMRREALVPFPAVQRGRLAPTQHPNPALQYALVNGDPLNGHSYLSGYISLLLRAEPYPTSRYGSQCMQPNNIMGIENICELAARLLFSAVEWARNIPFFPDLQITDQVALLRLVWSELFVLNAAQCAMPLHVAPLLAAAGLHASPMSADRVVAFMDHIRIFQEQVEKLKALHVDSAEYSCLKAIVLFSTDACGLSDAAHIENLQEKSQCALEEYVRSQYPNQPTRFGKLLLRLPSLRTVSSSVIEQLFFVRLVGKTPIETLIRDMLLSGSSFNWPYMPIQ

>XP_032829568.1_Nr2f1/2C_Petromyzon_marinus

MAMVVGNPWREPPNGTPIASGNSTQTSSSSSSSSSAAAASSAAVSGSASLLLGQQQQQQQTSSLQTSIAVAGNVSSVDSLSSSSLSSSSSSSSSSSSSSSSTSSAHGTKSASASHHHQQQQQQHQQQQQHHHQQQQQQQQQQNAE**CVVCGDKSSGKHYGQLTCEGC**KSFFKRSVRRNLNYA**CRAARACPIDQHHRNQCQYC**RLKKCLKVGMRREAVQRGRMPSSMQHPATPGGAYALQPNGGDPLLNGAGGHAASYLSGYISLLLRAEPYPTSRYGSQCMQPNNLMGIENICELAARLLFSAVEWARNIPFFPELQVGDQVALLRLVWSELFVLNAAQCAMPLHVAPLLAAAGLHASPMSAERVVAFMDHIRIFQEQVEKLKALHVDSAEYSCLKAIVLFTSDACGLSDTAHVESLQEKSQCALEEYVRTQYPSQPTRFGKLLLRLPSLRTVSSAVIEQLFFVRLVGKTPIETLIRDMLLSGSSFNWPYMSIQ

>XP_007891254.1_Nr2f1_Callorhinchus_milii

MAMVVSTWRDPQDDVAGAQGGQSAQAQPGQQPQQAATGAPHTPQTPSQSGPPSTPLGGSSQGGQPGTEKQPSQQQQQQQIE**CVVCGDKSSGKHYGQFTCEGC**KSFFKRSVRRNLTYT**CRANRNCPIDQHHRNQCQYC**RLKKCLKVGMRREAVQRGRMPPTQPNPGQYALTNGDPLNGHCYLSGYISLLLRAEPYPTSRYGSQCMQPNNIMGIENICELAARLLFSAVEWARNIPFFPDLQITDQVALLRLTWSELFVLNAAQCSMPLHVAPLLAAAGLHASPMSADRVVAFMDHIRIFQEQVEKLKALHVDSAEYSCLKAIVLFTSDACGLSDAAHIESLQEKSQCALEEYVRSQYPNQPSRFGKLLLRLPSLRTVSSSVIEQLFFVRLVGKTPIETLIRDMLLSGSSFNWPYMSIQ

>XP_007905008.1_Nr2f2_Callorhinchus_milii

MAMVVSTWRDPQDDVAGPQGTQPPQPQQGPQPPSGAPLTPQTPGQPGPPGTPAQSNQQNQQGEKQQQHIE**CVVCGDKSSGKHYGQFTCEGC**KSFFKRSVRRNLTYT**CRANRNCPIDQHHRNQCQYC**RLKKCLKVGMRREVCSFRTAAVQRGRMPPTQPTPGQFALTNGDPLNCHSYLSGYISLLLRAEPYPTSRYGSQCMQPNNIMGIENICELAARLLFSAVEWARNIPFFPDLQITDQVALLRLTWSELFVLNAAQCSMPLHVAPLLAAAGLHASPMSADRVVAFMDHIRIFQEQVEKLKALHVDSAEYSCLKAIVLFTSDACGLSDVAHVESLQEKSQCALEEYVRSQYPNQPTRFGKLLLRLPSLRTVSSSVIEQLFFVRLVGKTPIETLIRDMLLSGSSFNWPYMSIQ

>XP_007896989.1_Nr2f6_Callorhinchus_milii

MATVADDWRDSHTDSKPRSCNPEEALKSPAPSSHAGASDPDLGGQGIQAE**CVVCGDRASGKHYGQFTCEGC**KSFFKRSIRRNLSY**CCRSNRDCHIDQHHRNQCQYC**RLRKCFKVGMKREAVQRGRIAPVQSSPGEISLTSGDHLNGSSVPSLISLLLRAEPYPSSRYNSQCNLMSIDNICELAARLLFSVVEWARNIPFFHELQIPDQVALLRLSWSELFVLNAAQSSLPLHMAPLLAAAGLHSAQMSAERVVAFMDQIRIFQDQVEKLKNLQVDSAEYSCLKAIALYTPDASGLSEPAHIESLQEKVQESLAEYVRSQYPPQPQRFGKLLLRLPALRAVPASLIGQLFFMRLVGKTPIETLIRDMLLSGGSFNWPYMSAQ

>ctg17543_Nr2f1_Scyliorhinus_canicula

MAMVVSTWRDPQEDVAGAQGGQSAQTQPGQQQQAASGAPHTPQTPGQPGPPSTPLGGSSQSVQPVGEKQQPCQQQQQHIE**CVVCGDKSSGKHYGQFTCEGC**KSFFKRSVRRNLTYT**CRANRNCPIDQHHRNQCQYC**RLKKCLKVGMRREVQRGRMPPTQPNPGQYALTNGDPLNGHCYLSGYISLLLRAEPYPTSRYGSQCMQPNNIMGIENICELAARLLFSAVEWARNIPFFPDLQITDQVALLRLTWSELFVLNAAQCSMPLHVAPLLAAAGLHASPMSADRVVAFMDHIRIFQEQVEKLKALHVDSAEYSCLKAIVLFTSDACGLSDAAHIESLQEKSQCALEEYVRSQYPNQPSRFGKLLLRLPSLRTVSSSVIEQLFFVRLVGKTPIETLIRDMLLSGSSFNWPYMSIQ

>ctg95653_Nr2f2_Scyliorhinus_canicula

AVQRGRMPPTQPTPGQFALTNGDPLNCHSYLSGYISLLLRAEPYPTSRYGSQCMQPNNIMGIENICELAARLLFSAVEWARNIPFFPDLQITDQVALLRLTWSELFVLNAAQCSMPLHVAPLLAAAGLHASPMSADRVVAFMDHIRIFQEQVEKLKALHVDSAEYSCLKAIVLFTSGRQISIRKYG

>ctg23986_Nr2f6_Scyliorhinus_canicula

**NQCQYC**RLRKCFKVGMKREAVQRGRIPPTQANPGDLSLSSGDHLNGGNISGLISLLLRAEPYPTSRFSTQCTQYNLMSIDNICELAARLLFSAVEWARNIPFFHELQISDQVALLRLSWSELFVLNAAQSSLPLHMAPLLAAAGLHSAQMSADRVVSFMDQIRIFQDQVEKLKVLQVDSAEYSCLKAIALYTPDASGLSEPAHIESLQEKVQESLAEYVRCQYPPQPQRFGKLLLRLPALRAVPASLIGQLFFMRLVGKTPIETLIRDMLLSGGSFNWPYMPTQ

>ctg66070_Nr2f1_Leucoraja_erinacea

VQRGRMPPTQPNPGQYALTNGDPLNGHCYLSGYISLLLRAEPYPTSRYGSQCMQPNNIMGIENICELAARLLFSAVEWARNIPFFPDLQITDQVALLRLTWSELFVLNAAQCSMPLHVAPLLAAAGLHASPMSADRVVAFMDHIRIFQEQVEKLKALHVDSAEYSCLKAIVLFTSDACGLSDAAHIESLQEKSQCALEEYVRSQYPNQPSRFGKLLLRLPSLRTVSSSVIEQLFFVRLVGKTPIETLIRDMLLSGSSFNWPYMSIQ

>ctg62759_Nr2f2_Leucoraja_erinacea

AVQRGRMPPTQPTPGQFALTNGDPLNCHSYLSGYISLLLRAEPYPTSRYGSQCMQPNNIMGIENICELAARLLFSAVEWARNIPFFPDLQITDQVGLLRLTWSELFVLNAAQCAMPLHVAPLLAAAGLHASPMSADRVVAFMDHIRIFQEQVEKLKALHVDSAEYSCLKAIVLFTSDACGLSDVAHIESLQEKSQCALEEYVRSQYPNQPTRFGKLLLRLPSLRTVSSSVIEQLFFVRLVGKTPIETLIRDMLLSGSSFNWPYMSIQ

>ctg24393_Nr2f6_Leucoraja_erinacea

FPELQVSDQVALLRLSWSELFVLNAAQSSLPLHMAPLLAAAGLHSAHMSADRVVSFMDQIRAFQDQVEKLKLLQVDCAEYSCLKAIALYTPDASGLSEPAHVESLQEKVQECLAEYVRCQYPPQPQRFGKLLLRLPALRAVSASLIGQLFFMRLVGKTPIETLIRDMLLSGGSFNWPYMPTQ

>ENSLOCP00000001918.1_Nr2f1_Lepisosteus_oculatus

MAMVVSVWRDTQEDVAGGTPSGPNPVAQPAREQQQAASGAPHTPQTPSQPGPPSTPGTAGDKGQSQQNSGQNQQHIE**CVVCGDKSSGKHYGQFTCEGC**KSFFKRSVRRNLTYT**CRANRNCPIDQHHRNQCQYC**RLKKCLKVGMRREAVQRGRMPPTQPNPGQYALTNGDPLNGHCYLSGYISLLLRAEPYPTSRYGSQCMQPNNIMGIENICELAARLLFSAVEWARNIPFFPDLQITDQVSLLRLTWSELFVLNAAQCSMPLHVAPLLAAAGLHASPMSADRVVAFMDHIRIFQEQVEKLKALHVDSAEYSCIKAIVLFTSGWSDACGLSDAAHIESLQEKSQCALEEYVRSQYPNQPSRFGKLLLRLPSLRTVSSSVIEQLFFVRLVGKTPIETLIRDMLLSGSSFNWPYMSIQ

>ENSLOCP00000017729.1_Nr2f2_Lepisosteus_oculatus

MAMVVWRGSQDDVAESQGTLSSQAPQGPLSLPTPQPGQLNLAAPQVAPPTPQTPVQGGPPSTTAQSTPTNQTSQNQTGEKQQPQHIE**CVVCGDKSSGKHYGQFTCEGC**KSFFKRSVRRNLSYT**CRANRNCPIDQHHRNQCQYC**RLKKCLKVGMRREAVQRGRMPPTQPHHGQFALTNGDPLHCHSYLSGYISLLLRAEPYPTSRYGSQCMQPNNIMGIENICELAARMLFSAVEWARNIPFFPDLQITDQVALLRLTWSELFVLNAAQCSMPLHVAPLLAAAGLHASPMSADRVVAFMDHIRIFQEQVEKLKALHVDSAEYSCLKAIVLFTSDACGLSDVAHVESLQEKSQCALEEYVRSQYPNQPTRFGKLLLRLPSLRTVSSSVIEQLFFVRLVGKTPIETLIRDMLLSGSSFNWPYMSIQ

>ENSLOCP00000010977.1_Nr2f5_Lepisosteus_oculatus

MAMVVNQWQESISAEPGTQLQICSQEPGGTPIQAPGTPSGSTPGTDGDKIPNVDCMVC**GDKSSGKHYGQFTCEGC**KSFFKRSVRRNLSYT**CRGNRDCPIDQHHRNQCQYC**RLKKCLKVGMRREAVQRGRMSSSQSSPGQYLTNGGDSYNSQPYMSGFISLLLRAEPYPTSRYGAQCMQSNNLMGIENICELAARLLFSAVEWAKNIPFFPDLQLIDQVSLLRMSWSELFVLNAAQCSMPLHVAPLLAAAGLHASPMSAERVVAFMDHIRVFQEQVEKLKALQVDTAEYSCLKAIVLFTSDAMGLSDVAHVESIQEKSQCALEEYVRNQYPSQPNRFGRLLLRLPSLRIVSSPVIEQLFFVRLVGKTPIETLLRDMLLSGSSYNWPYMPVQRDRGLSLHYNENGP

>ENSLOCP00000003403.1_Nr2f6_Lepisosteus_oculatus

AMAMVSGGWGDPNGDTNGLGEKGGYLRDEEDGSPQAGGSDAEAGEEDKACVVD**CVVCGDKSSGKHYGVFTCEGC**KSFFKRSIRRNLSYT**CRSNRDCQIDQHHRNQCQYC**RLKKCFRVGMRKEAVQRGRIPPSHSGISPTSTPGAGGGGGGGGGGVDFFNGQPVSELISQLLRAEPYPSSRYGAQFGQQPQQGASVMGIDNICELAARLLFSTIEWARNIPYFPELPVSEQVALLRLSWSELFILNAAQSALPLHMAPLLAAAGFHAAPMSAERVVSFMDQVRVFQDQVDKLTRLQVDSAEYSCLKAIALFSPDACGLTDPAHVESLQEKAQVALTEYERAQYPGQPQRFGRLLLRLPALRAVPASLISQLFFMRLVGKTPIETLIRDMQLSGSSISWPYVPGQ

>XP_028660587.1_Nr2f1_Erpetoichthys_calabaricus

MFSYSVQRGRMPPTQPNPGQYALTNGDPLNGHCYLSGYISLLLRAEPYPTSRYGSQCMQPNNIMGIENICELAARLLFSAVEWARNIPFFPIYRSQDQVSLLRLTWSELFVLNAXQCSMPLHVAPLLAAXGLHASPMSADRVVAFMDHIRIFQEQVEKLKALHVDSAEVQRIKAIVLFTSDACGLSDAAHIESLQEKSQCALEEYVRSQYPNQPSRFGKLLLRLPSLRTVSSSVIEQLFFVRLVGKTPIETLIRDMLLSGSSFNWPYMSIQYLKKEQLKTIQRKSDREDQNKT

>XP_028678282.1_Nr2f2_Erpetoichthys_calabaricus

MAMVVWRGTQDDVAETQGTLSSQGGPLSLPTPQVAPPTPQTPVQGGPPTTTSQSTPSSQAGQQNQTVEKQQPQHIE**CVVCGDKSSGKHYGQFTCEGC**KSFFKRSVRRNLSYT**CRANRNCPIDQHHRNQCQYC**RLKKCLKVGMRREVSLFTAAVQRGRMPPTQPHHGQFALTNGDPLNCHSYLSGYISLLLRAEPYPTSRYGSQCMQPNNIMGIENICELAARMLFSAVEWARNIPFFPDLQITDQVALLRLTWSELFVLNAAQCSMPLHVAPLLAAAGLHASPMSADRVVAFMDHIRIFQEQVEKLKALHVDSAEYSCLKAIVLFTTDACGLSDVAHVESLQEKSQCALEEYVRSQYPNQPTRFGKLLLRLPSLRTVSSSVIEQLFFVRLVGKTPIETLIRDMLLSGSSFNWPYMSIQ

>XP_028649848.1_Nr2f5_Erpetoichthys_calabaricus

MAMVVNPWQEDISTGPGSQGSSQSQICSQEPVGTSLQTPGTPSGSTPGNDAHSGDKIPNVDCMVC**GDKSSGKHYGQFTCEGC**KSFFKRSVRRNLSYT**CRGNRDCPIDQHHRNQCQYC**RLKKCLKVGMRREAVQRGRMSNSQSSPGQYLSNGNDPYNSQSYLSGFISLLLRAEPYPTSRYGSQCMQTNNLMGIENICELAARLLFSAVEWAKNIPFFPDLQLADQVALLRMTWSELFVLNAAQCSMPLHVAPLLAAAGLHASPMSADRVVAFMDHIRIFQEQVEKLKALQVDSAEYSCLKAIVLFTSDAMGLSDVSHVESIQEKSQCALEEYERNQYPSQPNRFGRLLLRLPSLRIVSSPVIEQLFFVRLVGKTPIETLLRDMLLSGSSFNWPYIPMQRDRGLTLHYNETGH

>XP_028672412.1_Nr2f6_Erpetoichthys_calabaricus

MAMVSGGWGDPNGDTNGLGDKGYLREDDDGSPQAGGSDVEPGEDDKGCVVD**CVVCGDKSSGKHYGVFTCEGC**KSFFKRSIRRNLNYT**CRSNRDCQIDQHHRNQCQYC**RLKKCFRVGMRKEAVQRGRIPPSHSGISPTSTPGGGGGGGGGEYFNGQPVSELISQLLRAEPYPSSRFGSQYSQQHQQGSSVMGIDNICELAARLLFSTIEWARNIPYFPELPVSEQVALLRLSWSELFILNAAQSALPLHMAPLLAAAGFHASPMSAERVVSFMDQVRVFQDQVEKLIRLQVDSAEYSCLKAIALFSPDACGLTDPAHVESLQEKAQVALTEYVRSQYPGQPQRFGRLLLRLPALRAVPASLISQLFFMRLVGKTPIETLIRDMQLSGSSISWPYVSGQ

>ENSDARP00000116750.2_Nr2f1a_Danio_rerio

MAMVVSVWRDPQEDVAGGPPSGPNPAAQPAREQQQAASAAPHTPQTPSQPGPPSTPGTAGDKGSQNSGQSQQHIE**CVVCGDKSSGKHYGQFTCEGC**KSFFKRSVRRNLTYT**CRANRNCPIDQHHRNQCQYC**RLKKCLKVGMRREAVQRGRMPPTQPNPGQYALTNGDPLNGHCYLSGYISLLLRAEPYPTSRYGSQCMQPNNIMGIENICELAARLLFSAVEWARNIPFFPDLQITDQVSLLRLTWSELFVLNAAQCSMPLHVAPLLAAAGLHASPMSADRVVAFMDHIRIFQEQVEKLKALHVDSAEYSCIKAIVLFTSDACGLSDAAHIESLQEKSQCALEEYVRSQYPNQPSRFGKLLLRLPSLRTVSSSVIEQLFFVRLVGKTPIETLIRDMLLSGSSFNWPYMSIQ

>ENSDARP00000010118.5_Nr2f1b_Danio_rerio

MAMVVSAWRDPQEELAAVDDQSAAGREHLQHRHSPKSAEEKAQIAAQNQQHVE**CVVCGDKSSGKHYGQFTCEGC**KSFFKRSVRRNLSYT**CRANRNCPVDQHHRNQCQYC**RLKKCLKVGMRREAVQRGRMPPNQPNPSHYALTNGDHLNGQCYLSGYISLLLRAEPYPASRYGNQCMQSGNIMGIENICELAARLLFSAVEWARNIPFFPDLQITDQVSLLRLTWSELFVLNAAQSSMPLHVAPLLAAAGLHASPMSADRVVAFMDHIRFFQEQVEKLKALQVDSAEYSCAKAIVLFTSDACGLSDIPHIEGLQEKSQCALEEYVRSQYPNQPTRFGKLLLRLPALRMVSSSVIEQLFFVRLVGKTPIETLIRDMLLSGSSFNWPYMPIQ

>ENSDARP00000107285.2_Nr2f2_Danio_rerio

MAMVVWRGSQDDVAETHGTLSSQTQGGLSLPTPQPGQLGLTASQVAPPTPQTPVQGPPNNNNNTQSTPTNQTTQSQSEKQQPQHIE**CVVCGDKSSGKHYGQFTCEGC**KSFFKRSVRRNLTYT**CRANRNCPIDQHHRNQCQYC**RLKKCLKVGMRREAVQRGRMPPTQPHHGQFALTNGDPLHCHSYLSGYISLLLRAEPYPTSRYGSQCMQPNNIMGIENICELAARMLFSAVEWARNIPFFPDLQITDQVALLRLTWSELFVLNAAQCSMPLHVAPLLAAAGLHASPMSADRVVAFMDHIRIFQEQVEKLKALHVDSAEYSCLKAIVLFTSDACGLSDVAHVESLQEKSQCALEEYVRSQYPNQPTRFGKLLLRLPSLRTVSSSVIEQLFFVRLVGKTPIETLIRDMLLSGSSFNWPYMSIQ

>ENSDARP00000040768.6_Nr2f5_Danio_rerio

MAMVVNQWQENISADPGSQLQMCSQEPGGTPGTPSGSTPGNDALSGDKIPNVDCMVC**GDKSSGKHYGQFTCEGC**KSFFKRSVRRNLSYT**CRGNRDCPIDQHHRNQCQYC**RLKKCLKVGMRREAVQRGRMSNSQSSPGQYLSNGSDPYNGQPYLSGFISLLLRAEPYPTSRYGAQCMQSNNLMGIENICELAARLLFSAVEWAKNIPFFPDLQLMDQVALLRMSWSELFVLNAAQCSMPLHVAPLLAAAGLHASPMSAERVVAFMDHIRVFQEQVEKLKALQVDTAEYSCLKSIVLFTSDAMGLSDVAHVESIQEKSQCALEEYVRNQYPNQPNRFGRLLLRLPSLRIVSSPVIEQLFFVRLVGKTPIETLLRDMLLSGSSYNWPYMPVQRDRPISIHYNENGP

>ENSDARP00000009513.7_Nr2f6a_Danio_rerio

MAMVRGGWGDPNGETNGLGDKGYLRGDEDDGSPQGGGSDMEAGEDDKGCVVD**CVVCGDKSSGKHYGVFTCEGC**KSFFKRSVRRNLNYT**CRSNRDCQIDQHHRNQCQYC**RLKKCFRVGMRKEAVQRGRIPPSHSSLSPSTTPVGGNAGGGVSEFYNGQPVSELISQLLRAEPYPNSRYSHQYNQQMQGGGGGGSGMGIDSICELAARLLFSIIEWARNIPYFPELPVSEQVALLRLSWSELFILNAAQSALPLHMAPLLAAAGFHSSPMSAERVVSFMDQVRVFQDQVEKLTRLQVDSAEYSCLKAIALFSPDACGLTDPAHVESLQEKAQVALTEYERMQYPGQPQRFGRLLLRLPALRAVPASLISQLFFMRLVGKTPIETLIRDMQLSGSSISWPYAPGQ

>ENSDARP00000002435.9_Nr2f6b_Danio_rerio

MAMVSGGWANPNGSANGLGEKGYLRGEEEGSSPQAGNSDVEGGEEDKACVVD**CVVCGDKSSGKHYGVFTCEGC**KSFFKRSIRRNLNYT**CRSNRECQIDQHHRNQCQYC**RLKKCFRVGMRKEAVQRGRIPPSHAGISPASMVGAGGDVGGGPGMGADFFNGQPVSELISQLLRAEPYPNSRYGAQCGQQLQGANSSMMGIDNICELAARLLFSTIEWARNIPYFPDLPVSEQVALLRLSWSELFILNAAQSALPLHTAPLLAAAGFHSSPMPADRVVSFMDQVRVFQDQVDKLTRLQVDSVEYSCLKAIALFSPDACGLSDPAHVESLQEKAQVALTEYERMQYPGQPQRFGRLLLRLPALRAVPANLISQLFFMRLVGKTPIETLIRDMQLSGSSISWPYVPGQ

>ENSLACP00000019810.2_Nr2f1_Latimeria_chalumnae

MAMVVSSWRDPQEDVAGGTPSGPNPAAQSAREQQQTQSAAPHTPQTPGQPGPPSTPGTAGDKGQNQQNSGQSQQHIE**CVVCGDKSSGKHYGQFTCEGC**KSFFKRSVRRNLTYT**CRANRNCPIDQHHRNQCQYC**RLKKCLKVGMRREAVQRGRMPPTQPNPGQYALTNGDPLNGHCYLSGYISLLLRAEPYPTSRYGSQCMQPNNIMGIENICELAARLLFSAVEWARNIPFFPDLQITDQVALLRLTWSELFVLNAAQCSMPLHVAPLLAAAGLHASPMSADRVVAFMDHIRIFQEQVEKLKALHVDSAEYSCLKAIVLFTSDACGLSDVAHIESLQEKSQCALEEYVRSQYPNQPSRFGKLLLRLPSLRTVSSSVIEQLFFVRLNLPSSLEQWIKSKTTGYKISPVVRGAYLRETRLTAVSDPFVLIYEDVCCV

>ENSLACP00000009967.1_Nr2f2_Latimeria_chalumnae

MAMVVSAWRDPQDDVAGAQGTQPSQAPPGQGPPTGAPHTPQTPVQVGPPTTPAQSNQTNQPNQQNQVEKQQQHIE**CVVCGDKSSGKHYGQFTCEGC**KSFFKRSVRRNLSYT**CRANRNCPIDQHHRNQCQYC**RLKKCLKVGMRREVSSLFTAAVQRGRMPPTQPTHGQFALTNGDPLNCHSYLSGYISLLLRAEPYPTSRFGSQCMQPNNIMGIENICELAARMLFSAVEWARNIPFFPDLQITDQVALLRLTWSELFVLNAAQCSMPLHVAPLLAAAGLHASPMSADRVVAFMDHIRIFQEQVEKLKALHVDSAEYSCLKAIVLFTSDACGLSDVAHVESLQEKSQCALEEYVRSQYPNQPTRFGKLLLRLPSLRTVSSSVIEQLFFVRLVGKTPIETLIRDMLLSGSSFNWPYMSIQ

>ENSLACP00000011998.2_Nr2f5_Latimeria_chalumnae

MAMVVNVWQEDIPGASGSQARSQPQMCTQEAGGTPQTPGTPAGSTPGQEALSGDRAPAVDCMV**CGDKSSGKHYGQFTCEGC**KSFFKRSVRRNLSYT**CRGNRDCPIDQHHRNQCQYC**RLKKCLKVGMRREAVQRGRMTHPQTSPGQYTLTNSDQYNGHSYLTGFISMLLRAEPYPMSRYGGQCMQPNNFMGIENICELAARLLFSAIEWAKSIPFFPDLQLGDQVSLLRMTWSELFVLNAAQCSMPLHVAPLLAAAGLHASPMSADRVVAFMDHIRVFQEQVEKLKALHVDSAEYSCLKAIVLFTPDAVGVSDLAHVESIQEKSQCALEEYVRNQYPNQPSRFGRLLLRLPSLRIVSSPIIEQLFFVRLVGKTPIETLIRDMLLSGSSLNWPYMAMQ

>ENSLACP00000012183.1_Nr2f6_Latimeria_chalumnae

MAMVTGGWGDPNGETNGVIKGYPRKSEEEEEASPQGGGSDQEHGEEDKPGIQVD**CVVCGDKSSGKHYGAFTCEGC**KSFFKRSIRRNLNYT**CRSNRDCQIDQHHRNQCQYC**RLKKCFRVGMRKEAVQRGRIPLAQSTTSPNSTPGGDYFNGQPVSELIFQLLRAEPYPTARYGSQYTQQNTVMGIDNICELAARLLFSTVEWARNIPFFPELPVSDQISLLRLSWSELFVLNAAQSALPLHMAPLLAAAGFHTSPMSADRVVSFMDQIRIFQDQVEKLNRLQVDSAEYSCLKAIALFTPDACGLSDPAHVESLQEKAQVALTEYVRSQYPSQPQRFGRLLLRLPALRAVPASLISQLFFMRLVGKTPIETLIRDMLLSGSTFNWPYVGGQQ

>XP_029115375.1_Nr2f1a_Scleropages_formosus

MAMVVSVWRDPQEDVAGGTPSGPNPAAQPARDQQQAASAAPHTPQTPSQPGPPSTPGTAGDKGQGQNSAQNQQHIE**CVVCGDKSSGKHYGQFTCEGC**KSFFKRSVRRNLTYT**CRANRNCPIDQHHRNQCQYC**RLKKCLKVGMRREAVQRGRMPPTQPNPGQYALTNGDPLNGHCYLSGYISLLLRAEPYPTSRYGSQCMQPNNIMGIENICELAARLLFSAVEWARNIPFFPDLQITDQVSLLRLTWSELFVLNAAQCSMPLHVAPLLAAAGLHASPMSADRVVAFMDHIRIFQEQVEKLKALHVDSAEYSCIKAIVLFTSDACGLSDAAHIESLQEKSQCALEEYVRSQYPNQPSRFGKLLLRLPSLRTVSSSVIEQLFFVRLVGKTPIETLIRDMLLSGSSFNWPYMSIQ

>XP_029109033.1_Nr2f1b_Scleropages_formosus

MAMVVSVWRDPPEDVAGGTPGGPGAATQPAREQQPQAASATPHTPHTPGQAGHPSTPGTAGDKQGHGGQTSGQSQHIE**CVVCGDKSSGKHYGQFTCEGC**KSFFKRSVRRNLTYT**CRANRNCPIDQHHRNQCQYC**RLKKCLKVGMRREAVQRGRMPPSQPNPGQYALSNGDPLNGHCYLSGYISLLLRAEPYPTSRYGSQCMQPNSIMGIENICELAARLLFSAVEWARNIPFFPDLQITDQVSLLRLTWSELFVLNAAQCSMPLHVAPLLAAAGLHASPMSADRVVAFMDHIRIFQEQVEKLKALHVDSAEYSCMKAVVLFTPDACGLSDVAHIESLQEKSQCALEEYVRSQYPNQPSRFGKLLLRLPSLRTVSSSVIEQLFFVRLVGKTPIETLIRDMLLSGSSFNWPYMSIQ

>XP_018594757.1_Nr2f2a_Scleropages_formosus

MAMVVWRSSQDDVAETQSTLSSQGGPLSLPTHQPGQLNLAASQAAPPTPHTPVQGGPPSTTAQSTPTNQSGQNQAVEKQQPQHIE**CVVCGDKSSGKHYGQFTCEGC**KSFFKRSVRRNLTYT**CRANRNCPIDQHHRNQCQYC**RLKKCLKVGMRREAVQRGRMPPTQPHHGQFALTNGDPLHCHSYLSGYISLLLRAEPYPTSRYGSQCMQPNNIMGIENICELAARMLFSAVEWARNIPFFPDLQITDQVALLRLTWSELFVLNAAQCSMPLHVAPLLAAAGLHASPMSADRVVAFMDHIRIFQEQVEKLKALHVDSAEYSCLKAIVLFTTDACGLSDVAHVESLQEKSQCALEEYVRSQYPNQPTRFGKLLLRLPSLRTVSSSVIEQLFFVRLVGKTPIETLIRDMLLSGSSFNWPYMSIQ

>XP_018619549.2_Nr2f2b_Scleropages_formosus

MAMAVWRGSQDDVADTHGALSSQTTPGGPLSLPAPQPGQLNLAASQVAPPTPQTPVQGGPPSATAQSTPTNHSAQSQTAEKQQPQHIE**CVVCGDKSSGKHYGQFTCEGC**KSFFKRSVRRNLTYT**CRANRNCPIDQHHR**NQCQHCRLKKCLKVGMRREAVQRGRMPPTQPHHGQFALTNGDPLHCHSYLSGYISLLLRAEPYPTSRYGSQCMQPNNIMGIENICELAARMLFSAVEWARNIPFFPDLQITDQVALLRLTWSELFVLNAAQCSMPLHVAPLLAAAGLHASPMSADRVVAFMDHIRIFQEQVEKLKALHVDSAEYSCLKAIVLFTSDACGLSDVAHVESLQEKSQCALEEYVRSQYPNQPTRFGKLLLRLPSLRTVSSSVIEQLFFVRLVGKTPIETLIRDMLLSGSSFNWPYMPIQ

>XP_018595261.1_Nr2f5_Scleropages_formosus

MAMVVNQWQENISTDPGSQLQICGQDSGGTPGTPSGSTSGNDALSGDKLPNVDCLVC**GDKSSGKHYGQFTCEGC**KSFFKRSVRRNLSYT**CRGNRDCPIDQHHRNQCQYC**RLKKCLKVGMRREAVQRGRMSNSQTSPGQYLSTNSEAYNGQPYLSGFISLLMRAEPYPTSRYGTQCMQSNNLMGIENICELAARLLFSAVEWAKNIPFFPDLQLMDQVALLRMSWSELFVLNAAQCSMPLHVAPLLAAAGLHASPMSAERVVAFMDHIRVFQEQVEKLKALQVDTAEYSCLKAIVLFTSDAIGLSDVAHVESVQEKSQCALEEYVHNQYPGQPSRFGRLLLRLPSLRVVSSPVIEQLFFVRLVGKTPIETLLRDMLLSGSSYNWPYVPVQRDRPISLHYNENGP

>XP_018586811.1_Nr2f6b_Scleropages_formosus

MAMVSGGWGNPNGDTNGLGEKGYLQGDGSPHAGGSDQEVGEDDRACSMDCAV**CGDKSSGKHYGVFTCEGC**KSFFKRSIRRSLSYT**CRSNRDCQIDQHHR**NQCQFCRLKKCFRVGMRKEAVQRGRMPSSHPSLSPTPAPGAGRGPEEALNGQPVSELISQLLRAEPYPSSRYAPQLGLPQGTSNTMGIDNICELAARLLFSTIEWARNIPFFPELPMSDQVALLRLSWSELFILNAAQSALPLHTASLLAAAGFHAGPMSAERVVSFMDQVRLFQDQVEKLTRLQVDDAEYSCLKAIALFSPDACGLTDPTQVENLQEKAQVALTEYERLQYPGQPQRFGRLLLRLPALRAVPANLISELFFVRLVGKTPIETLIRDMQLSGSSISWPYTPGQ

>XP_018601468.1_Nr2f6a_Scleropages_formosus

MLTETGSSDVEAGEDDKACSVD**CVVCGDKSSGKHYGVFTCEGC**KSFFKRSIRRNLSYT**CRSNRECQIDQHHRNQCQYC**RLKKCFRVGMRKEAVQRGRIPPSHSGISPTSMAGVGSGGPAGSGDFFNGQPVSELISQLLRAEPYPPSRYGPQYGPQQQQGPGGAMGIDSICELAARLLFSTIEWARNIPYFPELPVSEQVALLRLSWSELFILNAAQSALPLHTAPLLAAAGFHSSPMSAERVVSFMDQVRVFQDQVDKLTRLQVDSAEYSCLKAIALFSPDACGLTDPVHVESLQEKAQVALTEYERLQYPGQPQRFGRLLLRLPALRAVPSSLISQLFFMRLVGKTPIETLIRDMQLSGSSISWPYVPGQ

>XP_014027894.1_Nr2f1a1_Salmo_salar

MAMVVSVWRDPQEDVVGGPPSGPNPATQPAREQQQTASAAPHTPQTPSQPGPPSTPGTAGDKGSQNSGQSGAQHIE**CVVCGDKSSGKHYGQFTCEGC**KSFFKRSVRRNLTYT**CRANRNCPIDQHHRNQCQYC**RLKKCLKVGMRREAVQRGRMPPTQPNPGQYGLTNGDPLNGHCYLSGYISLLLRAEPYPTSRYGSQCMQPNNIMGIENICELAARLLFSAVEWARNIPFFPDLQITDQVSLLRLTWSELFVLNAAQCSMPLHVAPLLAAAGLHASPMSADRVVAFMDHIRIFQEQVEKLKALHVDSAEYSCIKAIVLFTSDACGLSDAAHIESLQEKSQCALEEYVRSQYPNQPSRFGKLLLRLPSLRTVSSSVIEQLFFVRLVGKTPIETLIRDMLLSGSSFNWPYMSIQ

>XP_014016366.1_Nr2f1a2_Salmo_salar

MAMVVSVWRDPQEDVVGGPPSGPNPATQPAREQQQTASAAPHTPQTPSQPGPPSTPGTAGDKGSQNSGPQHIE**CVVCGDKSSGKHYGQFTCEGC**KSFFKRSVRRNLTYT**CRANRNCPIDQHHRNQCQYC**RLKKCLKVGMRREAVQRGRMPPTQPNPGQYALTNGDPLNGHCYLSGYISLLLRAEPYPTSRYGSQCMQPNNIMGIENICELAARLLFSAVEWARNIPFFPDLQITDQVSLLRLTWSELFVLNAAQCSMPLHVAPLLAAAGLHASPMSADRVVAFMDHIRIFQEQVEKLKALHVDSAEYSCIKAIVLFTSDACGLSDAAHIESLQEKSQCALEEYVRSQYPNQPSRFGKLLLRLPSLRTVSSSVIEQLFFVRLVGKTPIETLIRDMLLSGSSFNWPYMSIQ

>XP_013979582.1_Nr2f2a1_Salmo_salar

MAMVVWRGSQDDVAETQGTLSSQSQGGLSLPTPQLNLSGSQVAPPTPQTPVQGPPNNTVQSTPTNQTTQSEKQQPQHIE**CVVCGDKSSGKHYGQFTCEGC**KSFFKRSVRRNLSYT**CRANRNCPIDQHHRNQCQYC**RLKKCLKVGMRREAVQRGRMPPTQPHHGQFALTNGDPLHCHSYLSGYISLLLRAEPYPTSRYGSQCMQPNNIMGIENICELAARMLFSAVEWARNIPFFPDLQITDQVALLRLTWSELFVLNAAQCSMPLHVAPLLAAAGLHASPMSADRVVAFMDHIRIFQEQVEKLKALHVDSAEYSCLKAIVLFTSDACGLSDVAHVESLQEKSQCALEEYVRSQYPNQPTRFGKLLLRLPSLRTVSSSVIEQLFFVRLVGKTPIETLIRDMLLSGSSFNWPYMSIQ

>XP_014004594.1_Nr2f2a2_Salmo_salar

MAMVVWRGSQDDVSETQGTLSSQSQGGLSLPTPQMNLSGSQVAPPTPQTTVQGPPNNTVQSTPTNQTMQSEKQQPQHIE**CVVCGDKSSGKHYGQFTCEGC**KSFFKRSVRRNLSYT**CRANRNCPIDQHHRNQCQYC**RLKKCLKVGMRREAVQRGRMPPTQPHHGQFALTNGDPLHCHSYLSGYISLLLRAEPYPTSRYGSQCMQPNNIMGIENICELAARMLFSAVEWARNIPFFPDLQITDQVALLRLTWSELFVLNAAQCSMPLHVAPLLAAAGLHASPMSADRVVAFMDHIRIFQEQVEKLKVLHVDSAEYSCLKAIVLFTSDACGLSDVAHVESLQEKSQCALEEYVRSQYPNQPTRFGKLLLRLPSLRTVSSSVIEQLFFVRLVGKTPIETLIRDMLLSGSSFNWPYMSIQ

>XP_014032261.1_Nr2f2b_Salmo_salar

MAMVTWRNGEENIARDSQGGISSPVSQVGPLSLSTDLTGHLNPIPSLDIPSTHQTPQGAHSSNTSQSITSQTSTNSVDKQQLQQIE**CVVCGDKSSGKHYGQFTCEGC**KSFFKRSVRRNLSYT**CRASRNCPIDQHHRNQCQYC**RLKKCVKVGMRREAVQRGRMLPSQPYHGQFSITNGDPLQCHSYLSGYISLLMRAEPYPTSRYSTQSMQSNNVMGMENICELAARMLFSAVEWARNIPFFPDLQITDQVALLRLTWSELFVLNAAQCSMPVHVAPLLAAAGLHAAPMSAERVVSFMDHIRIFQEQVEKLKVLHVDSAEYSCIKAIVLFTSDACGLSDVTHVDDLQEKSQCALDGYIRNQYPNQPNRFGKLLLRLPSLRTVSSSVIEQLFFIRLVGKTPIETLIRDMLLSGSSFNWPYMSMQ

>ENSSSAP00000017044.1_Nr2f5a_Salmo_salar

MAMVVNQWQENISADPGSQLQICSQEPGGTPGTPSGSTPGNDALSGDKIPNVDCMVC**GDKSSGKHYGQFTCEGC**KSFFKRSVRRNLSYT**CRGNRDCPIDQHHRNQCQYC**RLKKCLKVGMRREAVQRGRMSNSQSSPGQYLTNGSDPYNGQPYLSGFISLLLRAEPYPTSRYGSQCMQGNNLMGIENICELAARLLFSAVEWAKNIPFFPDLQLMDQVALLRMSWSELFVLNAAQCSMPLHVAPLLAAAGLHASPMSAERVVAFMDHIRVFQEQVEKLKVLQVDTAEYSCLKSIVLFTSDAMGLSDVAHVESIQEKSQCALEEYVRNQYPSQPNRFGRLLLRLPSLRIVSSPVIEQLFFVRLVGKTPIETLLRDMLLSGSSYNWPYMPVQRDRPISLHYNENGP

>ENSSSAP00000095772.1_Nr2f5b_Salmo_salar

MAMVVNQWQENISADPGSQLQICSQEPGGTPGTPSGSTPGNDALSGDKIPNVDCMVC**GDKSSGKHYGQFTCEGC**KSFFKRSVRRNLSYT**CRGNRDCPIDQHHRNQCQYC**RLKKCLKVGMRREAVQRGRMSNSQSSPGQYLTNGSDPYNGQPYLSGFISLLLRAEPYPTSRYGSQCMQGNNLMGIENICELAARLLFSAVEWAKNIPFFPDLQLMDQVALLRMSWSELFVLNAAQCSMPLHVAPLLAAAGLHASPMSAERVVAFMDHIRVFQEQVEKLKVLQVDTAEYSCLKSIVLFTSDAMGLSDVAHVESIQEKSQCALEEYVRNQYPSQPNRFGRLLLRLPSLRIVSSPVIEQLFFVRLVGKTPIETLLRDMLLSGSSYNWPYMPVQRDRPISLHYNENGP

>XP_014045477.1_Nr2f6a1_Salmo_salar

MAMVSGGWGDPNGGTNGLGEKGYLRGEEEDGSPQAGGSDMEVGDEDKACVVD**CVVCGDKSSGKHYGVFTCEGC**KSFFKRSIRRNLNYT**CRSNRECQIDQHHRNQCQYC**RLEKCFRVGMRKEAVQRGRIPPSHSSLSPTGTPVGGGSGVGVAEFYNNNGGQPVSELISQLLRAEPYPSSRYGHQYNQQGQATGAGGAVMGIDNICELAARLLFSTIEWARNIPYFPELPVSEQVALLRLSWSELFILNAAQSALPLHMAPLLAAAGFHSSPMSAERVVSFMDQVRVFQNQVDKLTRLQVDSAEYSCLKAIVLFSPDACGLTDPVHVESLQEKAQVALTEYERMQYPGQPQRFGRLLLRLPALRAVPANLISQLFFMRLVGKTPIETLIRDMQLSGSSISWPYVPGQ

>XP_013995327.1_Nr2f6a2_Salmo_salar

MAMVSGGWGDPNGGTNGLGEKGYLRGEEEDGSPQAGGSDMEAGDEDKACVVD**CVVCGDKSSGKHYGVFTCEGC**KSFFKRSIRRNLNYT**CRSNRECQIDQHHRNQCQYC**RLEKCFRVGMRKEAVQRGRIPPPHSSLSPTGTPVGGGSGVGGADFYNNNGGQPVSELISQLLRAEPYPSSRYGHQYNQQGQATGAGGAVMGIDNICELAARLLFSTIEWARNIPYFPELPVSEQVALLRLSWSELFILNAAQSALPLHMAPLLAAAGFHSSPMSAERVVSFMDQVRVFQNQVEKLSRLQVDSAEYSCLKAIVLFSPDACGLTDPAHVESLQEKAQVALTEYERMQYPGQPQRFGRLLLRLPALRAVPANLISQLFFMRLVGKTPIETLIRDMQLSGSSISWPYVPGQ

>XP_013979062.1_Nr2f6b_Salmo_salar

MAMVSGGWGNPNRETNGLGGEKAYLRGDEEGSPQAGSDVEVGDEDKACVVD**CVVCGDKSSGKHYGVFTCEGC**KSFFKRSIRRNLSYS**CRSNRECQIDQHHRNQCQYC**RLKKCFRVGMRKEAVQRGRIPPSHSGISPTSLVGGGGGTGAGPVGGEFFNGQPVSELISQLLRAEPYPSSRYGPQYGQQMQGAGGGSVMGIDNICELAARLLFSTIEWARNIPYFPELPVSEQVALLRLSWSELFILNAAQSALPLHMAPLLAAAGFHSSPMSAERVVSFMDQVRVFQDQVDKLTRLQVDSAEYSCLKAIALLSPDACGLTDPVHVESLQEKAQVALMEYERMQYPGQPQRFGRLLLRLPALRAVPANLISQLFFMRLVGKTPIETLIRDMQLSGSSISWPYVPGQ

>ENSTRUP00000048930.1_Nr2f1a_Takifugu_rubripes

MAMVVSVWRDPQEDVAGGPPSGPNPAAQPAREQHQAASAAPHTPQTPSQPGPPSTPGTAGDKGSQNSGQSQQHIE**CVVCGDKSSGKHYGQFTCEGC**KSFFKRSVRRNLTYS**CRANRNCPIDQHHRNQCQYC**RLKKCLKVGMRREAVQRGRMPPTQPNPGQYALTNGDPLNGHCYLSGYISLLLRAEPYPTSRYGSQCMQPNNIMGIENICELAARLLFSAVEWSRNIPFFPDLQITDQVSLLRLTWSELFVLNAAQCSMPLHVAPLLAAAGLHASPMSADRVVAFMDHIRIFQEQVEKLKTLHVDSAEYSCLKAIVLFTSDACGLSDAAHIESLQEKSQCALEEYVRSQYPNQPSRFGKLLLRLPSLRTVSSSVIEQLFFVRLQQTDLRDIFTERTSGAPSPPGP

>ENSTRUP00000088094.1_Nr2f2_Takifugu_rubripes

MAMVAWRGSQDDVADTQGTLSSQTQGGLSLPTPQPGQLSLTASQTSQQSEKQPQHIE**CVVCGDKSSGKHYGQFTCEGC**KSFFKRSVRRNLTYT**CRANRNCPIDQHHRNQCQYC**RLKKCLKVGMRREAVQRGRVPPTQPHHGQFALTNGDPLHCHSYLSGYISLLLRAEPYPTSRYGSQCMQPNAVMGIENICELAARMLFSAVEWARNIPFFPDLQITDQVSLLRLTWSELFVLNAAQCSMPLHVAPLLAAAGLHASPMSADRVVAFMDHIRIFQEQVEKLKALHVDSAEYSCLKAIVLFTTDACGLSDVAHVESLQEKSQCALEEYVRSQYPNQPTRFGKLLLRLPSLRTVSSSVIEQLFFVRLVGKTPIETLIRDMLLSGSSFNWPYMSIQ

>ENSTRUP00000062483.1_Nr2f5_Takifugu_rubripes

MAMVVSPWPENISADPGSQLQICGQEPGGAPGTPNGSTSGNDALSGDKIPNVDCMVC**GDRSSGKHYGQFTCEGC**KSFFKRSVRRNLTYT**CRGNRDCPIDQHHRNQCQYC**RLKKCLKVGMRREGSNRRVRERSVRQESAPPLLGLTVVMAVQRGRTSNSQSSPGQYLTNGTDPYNGQPYLSGFISLLLRAEPYPTSRYGAQCMQGNNLMGIENICELAARLLFSAVEWAKNIPFFPDLQLMDQVALLRMSWSELFVLNAAQCSMPLHVAPLLAAAGLHASPMSAERVVAFMDHIRVFQEQVEKLKALQVDTAEYSCLKSIVLFTSDAMGLSDVAHVESIQEKSQCALEEYVRNQYPSQPNRFGRLLLRLPSLRIVSSPVIEQLFFVRLVGKTPIETLLRDMLLSGSSYNWPYMPAVQRERPLSLHYNENGP

>ENSTRUP00000050418.2_Nr2f6a_Takifugu_rubripes

MAMVSGGWGDPNGGTNGLGDKSYLRGDEEDGSPQAGGSDMEAGDEDKACVVD**CVVCGDKSSGKHYGVFTCEGC**KSFFKRSVRRNLTYT**CRSNRECQIDQHHRNQCQYC**RLKKCFRVGMRKEAVQRGRVPPQPSLSPSITPIGGSSGLGGEFYNNNNGISGGGQPVSELISQLLRAEPYPGSRYGHQYSQQSGPDNSMGIDNICELAARLLFSIVEWARNIPYFPELPVSDQVALLRLSWSELFILNAAQSALPLHMAPLLAAAGFHSSPMSAERVVSFMDQVRVFQDQVDKLTRLQVDSAEYSCLKAIALFSPDACGLTDPVHVESLQEKAQVALTEYERMQYPSQPQRFGRLLLRLPALRAVPASLISQLFFMRLVGKTPIETLIRDMQLSGSSISWPYVPGQ

>ENSTRUP00000010353.2_Nr2f6b_Takifugu_rubripes

MAMVSGGWGNPSGDTNGLGNKAYLKREEEEGSPQAGSSDVDVGDEDKACVVD**CVVCGDKSSGKHYGVFSCEGC**KSFFKRSIRRNLNYS**CRSNRECQIDQHHRNQCQYC**RLKKCFRVGMRKEAVQRGRIPPSQQGISPNSLPGGVGAGVPGHMGPDFFNGQPVSELISQLLRAEPYPVSRYGAPYGQTQMQGSAGGAPVMGIDSICELAARLLFSTIEWARNIPYFPELPVSEQVALLRLSWSELFILNAAQSSLPVHMAPLLAAAGFHSSPMSAERVVSFMDQVRVFQDQVDKLNRLQVDTAEYSCLKAIALFSPDACGLTDPAHVESLQEKAQVALTEYERIQYPSQPQRFGRLLLRLPALRAVPANLISQLFFMRLVGKTPIETLIRDMQLSGSSISWPYMSGQ

>ENSXMAP00000019453.2_Nr2f1a_Xiphophorus_maculatus

MAMVVSVWRDPQEDVAGGPPSGPNPAAQPAREQQQAASAAPHTPQTPSQPGPPSTPGTAGDKGSQNSGQSQQHIE**CVVCGDKSSGKHYGQFTCEGC**KSFFKRSVRRNLTYS**CRANRNCPIDQHHRNQCQYC**RLKKCLKVGMRREAVQRGRMPPTQPNPGQYALTNGDPLNGHCYLSGYISLLLRAEPYPTSRYGSQCMQPNNIMGIENICELAARLLFSAVEWARNIPFFPDLQITDQVSLLRLTWSELFVLNAAQCSMPLHVAPLLAAAGLHASPMSADRVVAFMDHIRIFQEQVEKLKTLHVDSAEYSCLKAIVLFTSDACGLSDAAHIESLQEKSQCALEEYVRSQYPNQPSRFGKLLLRLPSLRTVSSSVIEQLFFVRLQKQQNETDLQDVFTDIFTPSGAHPSPLHPSPLHSFPRTTSVCGVDHQSKEQTERHEIKKHF

>ENSXMAP00000014130.1_Nr2f2_Xiphophorus_maculatus

MAMVAWRGSQDDVADTQGALSSQTQGGLSLPTPQPGQLNLTASQIAPPTPQTPVQGPPNNAQSTPTNQTSQQQSEKQPQHIE**CVVCGDKSSGKHYGQFTCEGC**KSFFKRSVRRNLTYT**CRANRNCPIDQHHRNQCQYC**RLKKCLKVGMRREAVQRGRVPPTQPHHGQFALTNGDPLHCHSYLSGYISLLLRAEPYPTSRYGSQCMQPNNIMGIENICELAARMLFSAVEWARNIPFFPDLQITDQVALLRLTWSELFVLNAAQCSMPLHVAPLLAAAGLHASPMSADRVVAFMDHIRIFQEQVEKLKALHVDSAEYSCLKAIVLFTTDACGLSDVAHVESLQEKSQCALEEYVRSQYPNQPTRFGKLLLRLPSLRTVSSSVIEQLFFVRLVGKTPIETLIRDMLLSGSSFNWPYMSIQ

>ENSXMAP00000013467.1_Nr2f5_Xiphophorus_maculatus

MAMVVNQWPENISADPGSQLQMCGQEPGGGPGTPNGSTPGNDALSGDKLPNVDCMVC**GDKSSGKHYGQFTCEGC**KSFFKRSVRRNLTYT**CRGNRDCPIDQHHRNQCQYC**RLKKCLKVGMRREAVQRGRTSNSQSSPGQYLTNGSDPYNSQPYLSGFISLLLRAEPYPTSRYGAQCMQGNNLMGIENICELAARLLFSAVEWAKNIPFFPDLQLMDQVALLRMSWSELFVLNAAQCSMPLHVAPLLAAAGLHASPMSAERVVAFMDHIRVFQEQVEKLKALQVDTAEYSCLKSIVLFTSDAMGLSDVAHVESIQEKSQCALEEYVRNQYPSQPNRFGRLLLRLPSLRIVSSPVIEQLFFVRLVGKTPIETLLRDMLLSGSSYNWPYMPTVQRERPLSLHYNENGP

>ENSXMAP00000015028.2_Nr2f6a_Xiphophorus_maculatus

MAMVSGGWGDPNGGTNGLGDKGYLRGEEEDGSPQAGGSDMEAGDEDKACVVD**CVVCGDKSSGKHYGVFTCEGC**KSFFKRSVRRNLSYT**CRSNRECQIDQHHRNQCQYC**RLKKCFRVGMRKEAVQRGRIPPQPSLSPSITPIGGASGIGGSEFYNNNNGGGGGGQPVSELISQLLRAEPYPNSRYGHQYNQQAGPDNAMGIDNICELAARLLFSTVEWARNIPYFPELPVSDQVALLRLSWSELFILNAAQSALPLHMAPLLAAAGFHSSPMSAERVVSFMDQVRMFQDQVEKLMRLQVDSAEYSCLKAIALFSPDACGLTDSVHVESLQEKAQVALTEYERMQYPSQPQRFGRLLLRLPALRAVPASLISQLFFMRLVGKTPIETLIRDMQLSGSSISWPYVPGQ

>ENSXMAP00000029501.1_Nr2f6b_Xiphophorus_maculatus

MAMVSGGWGNPNGDTNGLGDKAYLRREEDNDSPQAGSSDVDVGDEDKTCVVD**CVVCGDKSSGKHYGVFTCEGC**KSFFKRSIRRNLNYS**CRSNRECQIDQHHRNQCQYC**RLKKCFRVGMRKEAVQRGRIPPSHSGISPNSLSGGPGGPGHIGAEYFNGQPVSELISQLLRAEPYSNSRYGAPYTQAQIQASASGASVMGIDSICELAARLLFSTIEWARNIPYFPELPVSEQVALLRLSWSELFILNAAQSALPLHMAPLLAAAGFHSSPMSAERVVSFMDQVRVFQDQVDKLNRLQVDSAEYSCLKAIALFSPDACGLSDPAHVESLQEKAQVALTEYERLTYPSQPQRFGRLLLRLPALRAVPANLISQLFFMRLVGKTPIETLIRDMQLSGSSISWPYATGQ

>ENSPNAP00000036065.1_Nr2f1a_Pygocentrus_nattereri

MAMVVSVWRDPQDDVAGGAPSGPNPAAQPAREQQQAASAAPHTPQTPSQPGPPSTPGTAGDKGSQNSAQSQQHIE**CVVCGDKSSGKHYGQFTCEGC**KSFFKRSVRRNLTYT**CRANRNCPIDQHHRNQCQYC**RLKKCLKVGMRREAVQRGRMPPTQPNPGQYALTNGDPLNGHCYLSGYISLLLRAEPYPTSRYGSQCMQPNNIMGIENICELAARLLFSAVEWARNIPFFPDLQITDQVSLLRLTWSELFVLNAAQCSMPLHVAPLLAAAGLHASPMSADRVVAFMDHIRIFQEQVEKLKALHVDSAEYSCMKAIVLFTSDACGLSDAAHIESLQEKSQCALEEYVRSQYPNQPSRFGKLLLRLPSLRTVSSSVIEQLFFVRLVGKTPIETLIRDMLLSGSSFNWPYMSIQ

>ENSPNAP00000024402.1_Nr2f1b_Pygocentrus_nattereri

MPESGQPNPGLYALTNGDPLNGHCYLSGFISLLLRAEPYPTSRYGNQCMQAGNITGIENICELAARLLFSAVEWARNIPFFPELQITDQVSLLRLTWSELFVLNAAQSSMPLHVAPLLAAAGLHASPMAADRVVAFMDHIRFFQEQVEKLKALNVDSAEFSCAKAIVLFTTDACGLSDVPHIESVQEKSQCALEEYVRSQYPSQPARFGKLLLRLPALRMVSSSVIEQLFFVRLVGKTPIETLIRDMLLTGSSYNWPYMAIQ

>ENSPNAP00000030209.1_Nr2f2_Pygocentrus_nattereri

MAMVVWRGSQDDVAETQGALSSQAQGGLSLAAPQPGQLNLAASQVAPPTPQTPVQPGGPNANAQSTPSNPTSQSQSDKQQQQHIE**CVVCGDKSSGKHYGQFTCEGC**KSFFKRSVRRNLTYT**CRANRNCPIDQHHRNQCQYC**RLKKCLKVGMRREAVQRGRMPPTQPHHGQFALTNGDPLHCHSYLSGYISLLLRAEPYPTSRYGSQCMQPNNIMGIENICELAARMLFSAVEWARNIPFFPDLQITDQVALLRLTWSELFVLNAAQCSMPLHVAPLLAAAGLHASPMSADRVVAFMDHIRIFQEQVEKLKALHVDSAEYSCMKAIVLFTTDACGLSDVAHVESLQEKSQCALEEYVRSQYPNQPTRFGKLLLRLPSLRTVSSSVIEQLFFVRLVGKTPIETLIRDMLLSGSSFNWPYMPIQ

>ENSPNAP00000021686.1_Nr2f5_Pygocentrus_nattereri

MAMVVNQWQENISADPGSQLQLCSQEPGGGTPGTPSGSTPGNDALSADKIPNVDCMVC**GDKSSGKHYGQFTCEGC**KSFFKRSVRRNLSYT**CRGNRDCPIDQHHRNQCQYC**RLKKCLKVGMRREAVQRGRMSSSQSSPGQYLTNGNDPYNGQPYLSGFISLLLRAEPYPTSRYGAQCMQSNNLMGIENICELAARLLFSAVEWAKNIPFFPDLQLMDQVALLRMSWSELFVLNAAQCSMPLHVAPLLAAAGLHASPMSAERVVAFMDHIRVFQEQVEKLKALQVDTAEYSCLKSIVLFTSDAMGLSDVAHVESIQEKSQCALEEYVRNQYPNQPNRFGRLLLRLPSLRIVSSPVIEQLFFVRLVGKTPIETLLRDMLLSGSSYNWPYMPVQRDRPISIHYNENGP

>ENSPNAP00000017404.1_Nr2f6b_Pygocentrus_nattereri

MAMVGGGWGNPNGSTNGLGEKGYLQGEEDESSPQAGNSDAEGGEDDKICVVD**CVVC**GDKSSGKHYGVFTCEGCKSFFKRSVRRNLSYTCRSNRECQIDQHHR**NQCQYC**RLKKCFRVGMRKEAVQRGRIPPSHSGISPTSLVGGGGGGGGVGGPAMGGDFFNGQPAPELITQLLRAEPYPNSRYGAQCGQQLGGGNSAVMGIDNICELAARLLFSIIEWARNIPFFPDLPVSEQVALLRLSWSELFILNAAQSALPLHMAPLLAAAGFHASPMSAERVVSFMDQVRVFQDQVDKLTRLQVDSAEYSCLKAIALFSPDACGLTDPAHVESLQEKAQVALTEYERMQYPTQPQRFGRLLLRLPSLRAVPANLISQLFFMRLVGKTPIETLIRDMQLSGSSISWPYVPGQ

>ENSNBRP00000001354.1_Nr2f1a_Neolamprologus_brichardi

MAMVVSVWRDPQEDVAGPPSGPNPAAQPAREQQQAASAAPHTPQTPSQPGPPSTPGTAGDKGSQNSGQSQQHIE**CVVCGDKSSGKHYGQFTCEGC**KSFFKRSVRRNLTYS**CRANRNCPIDQHHRNQCQYC**RLKKCLKVGMRREAVQRGRMPPTQPNPGQYALTNGDPLNGHCYLSGYISLLLRAEPYPTSRYGSQCMQPNNIMGIENICELAARLLFSAVEWARNIPFFPDLQITDQVSLLRLTWSELFVLNAAQCSMPLHVAPLLAAAGLHASPMSADRVVAFMDHIRIFQEQVEKLKTLHVDSAEYSCLKAIVLFTSDACGLSDAAHIESLQEKSQCALEEYVRSQYPNQPSRFGKLLLRLPSLRTVSSSVIEQLFFVRLVGKTPIETLIRDMLLSGSSFNWPYMSIQ

>ENSNBRP00000027173.1_Nr2f2_Neolamprologus_brichardi

MAMVVWRGSQDDVADTQGTLSSQTQGGLSLPTPQAGQLNLTASQVAPPTPQTPVQGPPNNTQSTPTNQTSQQSEKQPQHIE**CVVCGDKSSGKHYGQFTCEGC**KSFFKRSVRRNLTYT**CRANRNCPIDQHHRNQCQYC**RLKKCLKVGMRREAVQRGRVPPTQPHHGQFALTNGDPLHCHSYLSGYISLLLRAEPYPTSRYGSQCMQPNNIMGIENICELAARMLFSAVEWARNIPFFPDLQITDQVALLRLTWSELFVLNAAQCSMPLHVAPLLAAAGLHASPMSADRVVAFMDHIRIFQEQVEKLKALHVDSAEYSCLKAIVLFTTDACGLSDVAHVESLQEKSQCALEEYVRSQYPNQPTRFGKLLLRLPSLRTVSSSVIEQLFFVRLVGKTPIETLIRDMLLSGSSFNWPYMPIQ

>ENSNBRP00000001992.1_Nr2f6a_Neolamprologus_brichardi

MAMVSGGWGDPNGGTNGLGDKGYLRGEEEDGSPQAGGSDMEAGEDDKACVVD**CVVCGDKSSGKHYGVFTCEGC**KSFFKRSVRRNLSYT**CRSNRECQIDQHHRNQCQYC**RLKKCFRVGMRKEAVQRGRIPPQPSLSPSITPIGGASGLGGGEFYNNNNGGSGGGQPVSELISQLLRAEPYPNSRYGHQYNQQAGPDNAMGIDNICELAARLLFSTVEWARNIPYFPELPVSDQVALLRLSWSELFILSAAQSALPLHMAPLLAAAGFHSSPMSAERVVSFMDQVRVFQDQVDKLTRLQVDSAEYSCLKAIALFSPDACGLTDPVHVESLQEKAQVALTEYERMQYPSQPQRFGRLLLRLPALRAVPASLISQLFFMRLIGKTPIETLIRDMQLSGNSISWPYVPGQ

>ENSNBRP00000028293.1_Nr2f6b_Neolamprologus_brichardi

MAMVSGGWGNPNGGANGLGEKAYLRRGEDEEGSPRAGSSDVDVGDDDKACVVD**CVVCGDKSSGKHYGVFTCEGC**KSFFKRSIRRNLSYS**CRRCAIHSAHGRGV**PGVTFGLRLLVNHAERGRDAGAVSTLPGNVRVTSDPVREAAVQVAFSETLTPPVSELISQLLRAEPYPPSRYGAPYGQAQMQASASGASVMGIDSICELAARLLFSTIEWARNIPYFPELPVSEQVALLRLSWSELFILNAAQSALPLHMAPLLAAAGFHSSPMSAERVVSFMDQVRIFQDQVEKLNRLQVDSAEYSCLKAIALFSPDACGLTDPAHVESLQEKAQVALTEYERLQYPNQPQRFGRLLLRLPALRAVPANLISQLFFMRLVGKTPIETLIRDMQLSGSSISWPYAPGQ

>ENSGMOP00000000785.1_Nr2f1a_Gadus_morhua

MAMVVSVWRDPQEDVAGGPPNGPNPAVQPAREQQQTASAAPHTPQTPSQPGPPSTPGAAGDKGSQNSGQSQQHIE**CVVCGDKSSGKHYGQFTCEGC**KSFFKRSVRRNLTYT**CRANRNCPIDQHHRNQCQYC**RLKKCLKVGMRREAVQRGRMPPTQPNPGQYALTNGDPLNGHCYLSGYISLLLRAEPYPTSRYGSQCMQPNNIMGIENICELAARLLFSAVEWARNIPFFPDLQITDQVSLLRLTWSELFVLNAAQCSMPLHVAPLLAAAGLHASPMSADRVVAFMDHIRIFQEQVEKLKALHVDSAEYSCIKAIVLFTSDACGLSDAAHIESLQEKSQCALEEYVRSQYPNQPSRFGKLLLRLPSLRTVSSSVIEQLFFVRLVGKTPIETLIRDMLLSGSSFNWPYMSIQ

>ENSGMOP00000016617.1_Nr2f2_Gadus_morhua

MAMVVWRGSQDDVADTQGSLSSQTQGGLSLPTPQPGQLNLSGSQVAPSSQTTVQGPPNNTQSTPTNQSSEKQPQHIE**CVVCGDKSSGKHYGQFTCEGC**KSFFKRSVRRNLTYT**CRANRNCPIDQHHRNQCQYC**RLKKCLKVGMRREAVQRGRMPPTQPHHGQFALTNGDPLHCHSYLSGYISLLLRAEPYPTSRYGSQCMQPNNIMGIENICELAARMLFSAVEWARNIPFFPDLQITDQVALLRLTWSELFVLNAAQCSMPLHVAPLLAAAGLHASPMSADRVVAFMDHIRIFQEQVEKLKALHVDSAEYSCLKAIVLFTSADACGLSDVAHVESLQEKSQCALEEYVRSQYPNQPTRFGKLLLRLPSLRTVSSSVIEQLFFVRLVGKTPIETLIRDMLLSGSSFNWPYMSIQ

>ENSGMOP00000016025.1_Nr2f5_Gadus_morhua

PGSQLQICGQEPGGTPGTPNGSTPGNEGLSGDKIPNVDCMVC**GDKSSGKHYGQFTCEGC**KSFFKRSVRRNLSYT**CRGNRDCPIDQHHRNQCQYC**RLKKCLKVGMRREXXXXXXXXXXXXXXXXXXXXXXXXXXXXXXXXXXXXXXLRAEPYPTSRYGAQCMQGNNLMGIENICELAARLLFSAVEWAKNIPFFPDLQLMDQVALLRMSWSELFVLNAAQCSMPLHVAPLLAAAGLHASPMSAERVVAFMDHIRVFQEQVEKLKALQVDAAEYSCLKSIVLF

>ENSGMOP00000011070.1_Nr2f6a_Gadus_morhua

AMAMVSGGWGDPNGGTNGFGDKSYLKGEEEDGSPQAGGSDMEAGEDDKACVVD**CVVCGDKSSGKHYGVFTCEGC**KSFFKRSIRRNLNYT**CRSNRECQIDQHHRNQCQYC**RLKKCFRVGMRKEAVQRGRIPPQPSLSPSITPLGGAGGLGGIGGEYYNNNNNNGGGGGGGGGGQPVSELISQLLRAEPYPSSRYSHQYSQQGGADNAMGIDNICELAARLLFSTIEWARNIPFFPELPVSDQVALLRLSWSELFILNAAQSALPLHMAPLLAAAGFHSSPMSAERVVSFMDQVRVFQDQVDKLTRLQVDSAEYSCLKAIAIFSPDACGLTDPAHVESLQEKAQVALTEYERMQYPNQPQRFGRLLLRLPALRAVPASLISQLFFMRLVGKTPIETLIRD

>ENSAMXP00005017046.1_Nr2f1a_Astyanax_mexicanus

MAMVVSVWRDPQDDVAGGAPSGPNPAAQPAREQQQAASAAPHTPQTPSQPGPPSTPGTAGDKGSQNSAQSQQHIE**CVVCGDKSSGKHYGQFTCEGC**KSFFKRSVRRNLTYT**CRANRNCPIDQHHRNQCQYC**RLKKCLKVGMRREAVQRGRMPPTQPNPGQYALTNGDPLNGHCYLSGYISLLLRAEPYPTSRYGSQCMQPNNIMGIENICELAARLLFSAVEWARNIPFFPDLQITDQVSLLRLTWSELFVLNAAQCSMPLHVAPLLAAAGLHASPMSADRVVAFMDHIRIFQEQVEKLKALHVDSAEYSCIKAIVLFTSDACGLSDAAHIESLQEKSQCALEEYVRSQYPNQPSRFGKLLLRLPSLRTVSSSVIEQLFFVRLVGKTPIETLIRDMLLSGSSFNWPYMSIQ

>ENSAMXP00005019213.1_Nr2f2_Astyanax_mexicanus

MAMVVWRGSQDDVAETQGALSSQAQGGLSLAAPQPGQLNLAASQVAPPTPQTPVQPGGPNANAQSTPSNPTSQSQSDKQQQQHIE**CVVCGDKSSGKHYGQFTCEGC**KSFFKRSVRRNLTYT**CRANRNCPIDQHHRNQCQYC**RLKKCLKVGMRREVSLFTAAVQRGRMPPTQPHHGQFALTNGDPLHCHSYLSGYISLLLRAEPYPTSRYGSQCMQPNNIMGIENICELAARMLFSAVEWARNIPFFPDLQITDQVALLRLTWSELFVLNAAQCSMPLHVAPLLAAAGLHASPMSADRVVAFMDHIRIFQEQVEKLKALHVDSAEYSCLKAIVLFTTDACGLSDVAHIESLQEKSQCALEEYVRSQYPNQPTRFGKLLLRLPSLRTVSSSVIEQLFFVRLVGKTPIETLIRDMLLSGSSFNWPYMSIQ

>ENSAMXP00005043400.1_Nr2f5_Astyanax_mexicanus

MAMVVNQWQENISADPGSQLQLCGQEPGGTPGTPSGSTPGNDALSADKIPNVDCMVC**GDKSSGKHYGQFTCEGC**KSFFKRSVRRNLSYT**CRGNRDCPIDQHHRNQCQYC**RLKKCLKVGMRREAVQRGRMSSSQSSPGQYLTNGNDPYNGQPYLSGFISLLLRAEPYPTSRYGAQCMQSNNLMGIENICELAARLLFSAVEWAKNIPFFPDLQLMDQVALLRMSWSELFVLNAAQCSMPLHVAPLLAAAGLHASPMSAERVVAFMDHIRVFQEQVEKLKALQVDTAEYSCLKSIVLFTSDAMGLSDVAHVESIQEKSQCALEEYVRNQYPNQPNRFGRLLLRLPSLRIVSSPVIEQLFFVRLVGKTPIETLLRDMLLSGSSYNWPYMPVQRDRPISLHYNENGP

>ENSAMXP00005048901.1_Nr2f6b_Astyanax_mexicanus

MAMVGGGWGNPNGSTNGLGDKGYLRGEEDESSPQAGNSDVEGGEDDKACVVD**CVVCGDKSSGKHYGVFTCEGC**KSFFKRSIRRNLSYT**CRSNRECQIDQHHRNQCQYC**RLKKCFRVGMRKEAVQRGRIPPSHSGISPTSMVGAGGGGVGGQGMGGEYFNGQPAPELITQLLRAEPYPNSRYGAQCGQQLGGGNSAVMGIDNICELAARLLFSTIEWARNIPYFPDLPVSEQVALLRLSWSELFILNAAQSALPLHMAPLLAAAGFHASPMSAERVVSFMDQVRVFQDQVDKLTRLQVDSAEYSCLKAIALFSPDACGLTDPAHVESLQEKAQVALTEYERMQYPTQPQRFGRLLLRLPSLRAVPANLISQLFFMRLVGKTPIETLIRDMQLSGSSISWPYVPGQ

>ENSIPUP00000026477.1_Nr2f1a_Ictalurus_punctatus

MAMVVSVWRDPQEDVAGGAASGPNPAAQPAREQQQAASAAPHTPQTPSQPGAPSTPGTAGDKSSQNSGQSQQHIE**CVVCGDKSSGKHYGQFTCEGC**KSFFKRSVRRNLTYT**CRANRNCPIDQHHRNQCQYC**RLKKCLKVGMRREAVQRGRMPPTQPNPGQYALTNGDPLNGHCYLSGYISLLLRAEPYPTSRYGSQCMQPNNIMGIENICELAARLLFSAVEWARNIPFFPDLQITDQVSLLRLTWSELFVLNAAQCSMPLHVAPLLAAAGLHASPMSADRVVAFMDHIRIFQEQVEKLKALHVDSAEYSCIKAIVLFTSDACGLSDAAHIESLQEKSQCALEEYVRSQYPNQPSRFGKLLLRLPSLRTVSSSVIEQLFFVRLVGKTPIETLIRDMLLSGSSFNWPYMSIQ

>ENSIPUP00000007211.1_Nr2f2_Ictalurus_punctatus

MAMVVWRGSQDDVAETQGALSSQAQGGLNLATPQPGQLNLTASQVAPPTPQTPVQGAPNSNAQSTPTNQTSQSQSDKQQQQHIE**CVVCGDKSSGKHYGQFTCEGC**KSFFKRSVRRNLTYT**CRANRNCPIDQHHRNQCQYC**RLKKCLKVGMRREAVQRGRMPPTQPHHGQFALTNGDPLHCHSYLSGYISLLLRAEPYPTSRYGSQCMQPNNIMGIENICELAARMLFSAVEWARNIPFFPDLQITDQVALLRLTWSELFVLNAAQCSMPLHVAPLLAAAGLHASPMSADRVVAFMDHIRIFQEQVEKLKALHVDSAEYSCLKAIVLFTTDACGLSDVAHIESLQEKSQCALEEYVRSQYPNQPTRFGKLLLRLPSLRTVSSSVIEQLFFVRLVGKTPIETLIRDMLLSGSSFNWPYMSIQ

>ENSIPUP00000009276.1_Nr2f5_Ictalurus_punctatus

MAMVVNQWQESIAADPGSQLQLCGQEPGGAPATPSASTPGNDAIGADKLASVDCMVC**GDKSSGKHYGQFTCEGC**KSFFKRSVRRNLTYS**CRGNRDCPVDQHHRNQCQYC**RLKKCLKVGMRREAVQRGRMSSSQTSPGQYLPNGNDPYNGQPYLSGFISLLLRAEPYPTSRYGAQCMQSNNLMGIENICELAARLLFSAVEWAKNIPFFPDLQLMDQVALLRMSWSELFVLNAAQCSMPLHVAPLLAAAGLHASPMSAERVVAFMDHIRVFQEQVEKLKALQVDTAEYSCLKSIVLFTSDAMGLSDVAHVESIQEKSQCALEEYVRNQYPNQPNRFGRLLLRLPSLRIVSSPVIEQLFFVRLVGKTPIETLLRDMLLSGSSYNWPYMPVQRDRPLSLHYNENGP

>ENSIPUP00000010784.1_Nr2f6_Ictalurus_punctatus

MAMVGGGWGNPNGSTNGLGEKGYLRGEEDESSPQAGNSDAEGGEDDKACVVD**CVVCGDKSSGKHYGVFTCEGC**KSFFKRSIRRNLSYT**CRSNRECQIDQHHRNQCQYC**RLKKCFRVGMRKEAVQRGRIPPSHSGISPTTMVSAGGGGPGGPGMAGDFFNGQPAPELISQLLRAEPYPSSRYGAQCGQQLPGGHGSVMGIDSICELAARLLFSTIEWARNIPFFPDLPVSEQVALLRLSWSELFILNAAQSALPLHMAPLLAAAGFHASPMSAERVVSFMDQVRVFQDQVDKLTRLQVDSAEYSCLKAIALFSPDACGLTDPAHVESLQEKAQVALTEYERMQYPTQPQRFGRLLLRLPSLRAVPANLISQLFFMRLVGKTPIETLIRDMQLSGSSISWPYVPGQ

>ENSXCOP00000003906.1_Nr2f2_Xiphophorus_couchianus

MAMVAWRGSQDDVADTQGALSSQTQGGLSLPTPQPGQLNLTASQIAPPTPQTPVQGPPNNKQPQHIE**CVVCGDKSSGKHYGQFTCEGC**KSFFKRSVRRNLTYT**CRANRNCPIDQHHRNQCQYC**RLKKCLKVGMRREAVQRGRVPPTQPHHGQFALTNGDPLHCHSYLSGYISLLLRAEPYPTSRYGSQCMQPNNIMGIENICELAARMLFSAVEWARNIPFFPDLQITDQVALLRLTWSELFVLNAAQCSMPLHVAPLLAAAGLHASPMSADRVVAFMDHIRIFQEQVEKLKALHVDSAEYSCLKAIVLFTTDACGLSDVAHVESLQEKSQCALEEYVRSQYPNQPTRFGKLLLRLPSLRTVSSSVIEQLFFVRLVGKTPIETLIRDMLLSGSSFNWPYMSIQ

>ENSXCOP00000005591.1_Nr2f5_Xiphophorus_couchianus

SPFPPALFLPSIFLFSTYHLSLSTLFHYSSLLPHFHPLTHGSSLHFHCLSPLSHLLRVTVDGVISQTVGMPEQVFSKWAFERSPHHHSSPAGGWDVCLLESLTSVCPFVSWETFVKLMQPPVFVCFWTAVQRGRTSNSQSSPGQYLTNGSDPYNSQPYLSGFISLLLRAEPYPTSRYGAQCMQGNNLMGIENICELAARLLFSAVEWAKNIPFFPDLQLMDQVALLRMSWSELFVLNAAQCSMPLHVAPLLAAAGLHASPMSAERVVAFMDHIRVFQEQVEKLKALQVDTAEYSCLKSIVLFTSDAMGLSDVAHVESIQEKSQCALEEYVRNQYPSQPNRFGRLLLRLPSLRIVSSPVIEQLFFVRLVGKTPIETLLRDMLLSVAFLLPHILTCFVLICLKYCKWRQKKQA

>ENSXCOP00000026555.1_Nr2f6a_Xiphophorus_couchianus

PKSCFLYPPSPCLLPPGPPTKLQQFNAGESHLSRASLISQLLRAEPYPNSRYGHQYNQQAGPDNAMGIDNICELAARLLFSTVEWARNIPYFPELPVSDQVALLRLSWSELFILNAAQSALPLHMAPLLAAAGFHSSPMSAERVVSFMDQVRMFQDQVEKLMRLQVDSAEYSCLKAIALFSPDACGLTDSVHVESLQEKAQVALTEYERMQYPSQPQRFGRLLLRLPALRAVPASLISQLFFMRLVGKTPIETLIRDMQLSGSSISWPYVPGQ

>ENSXCOP00000011776.1_Nr2f6b_Xiphophorus_couchianus

MAMVSGGWGNPNGDTNGLGDKAYLRREEDNDSPQAGSSDVDVGDEDKTCVVD**CVVCGDKSSGKHYGVFTCEGC**KSFFKRSIRRNLNYS**CRSNRECQIDQHHRNQCQYC**RLKKCFRVGMRKEAVQRGRIPPSHSGISPNSLSGGPGGPGHIGAEYFNGQPVSELISQLLRAEPYSNSRYGAPYTQAQIQASASGASVMGIDSICELAARLLFSTIEWARNIPYFPELPVSEQVALLRLSWSELFILNAAQSALPLHMAPLLAAAGFHSSPMSAERVVSFMDQVRVFQDQVDKLNRLQVDSAEYSCLKAIALFSPGTSELIQMKKLFYVKNIILFQHQQNNFKKCYLGSLRFCVSVFSNKLFSDFLHYGCMMSGRHELEIIKHNIIN

>ENSCVAP00000023386.1_Nr2f1a_Cyprinodon_variegatus

MAMVVSVWRDPQEDVAGGPPNGPNPAAQPAREQQQAASAAPHTPQTPSQPGPPSTPGTAGDKGSQNSGQSQQHIE**CVVCGDKSSGKHYGQFTCEGC**KSFFKRSVRRNLTYS**CRANRNCPIDQHHR**NQCQYCRLKKCLKVGMRREAVQRGRMPPTQPNPGQYALTNGDPLNGHCYLSGYISLLLRAEPYPTSRYGSQCMQPNNIMGIENICELAARLLFSAVEWARNIPFFPDLQITDQVSLLRLTWSELFVLNAAQCSMPLHVAPLLAAAGLHASPMSADRVVAFMDHIRIFQEQVEKLKTLHVDSAEYSCLKAIVLFTSDACGLSDAAHIESLQEKSQCALEEYVRSQYPNQPSRFGKLLLRLPSLRTVSSSVIEQLFFVRLGAAACCDSMGVLFCLLLLLRTQGFIEELEDGFKTHPGDQRSSTCSFSLN

>ENSCVAP00000001470.1_Nr2f2_Cyprinodon_variegatus

MAMVAWRGSQDDVADTQGALSSQTQGGLSLPTPQPGQLNLTASQIAPPTPQTPVQGPPNNAQSTPTNQTSQQQSEKQPQHIE**CVVCGDKSSGKHYGQFTCEGC**KSFFKRSVRRNLTYT**CRANRNCPIDQHHRNQCQYC**RLKKCLKVGMRREVSLFTAAVQRGRVPPTQPHHGQFALTNGDPLHCHSYLSGYISLLLRAEPYPTSRYGSQCMQPNNIMGIENICELAARMLFSAVEWARNIPFFPDLQITDQVALLRLTWSELFVLNAAQCSMPLHVAPLLAAAGLHASPMSADRVVAFMDHIRIFQEQVEKLKALHVDSAEYSCLKAIVLFTTDACGLSDVAHVESLQEKSQCALEEYVRSQYPNQPTRFGKLLLRLPSLRTVSSSVIEQLFFVRLVGKTPIETLIRDMLLSGSSFNWPYMSIQ

>ENSCVAP00000018632.1_Nr2f6b_Cyprinodon_variegatus

MAMVSGGWGNPNGDTNGLGEKAYLRREEDNDSPQAGSSDVDVGDEDKAGVVD**CVVCGDKSSGKHYGVFTCEGC**KSFFKRSIRRNLNYS**CRSNRECQIDQHHRNQCQYC**RLKKCFRVGMRKEAVQRGRLPPTHSGISPNPLSGGAGGPGPGHLGADYFNGNPVSELISQLLRAEPYTNSRYGAPYAQAQMQASAGGASVMGIDSICELAARLLFSTIEWARNIPYFPELPVSEQVALLRLSWSELFILNAAQSALPLHMAPLLAAAGFHSSPMSAERVVSFMDQVRVFQDQVDKLNRLQVDSAEYSCLKAIALFSPDACGLSDPAHVESLQEKAQVALTEYERLTYPSQPQRFGRLLLRLPALRAVPANLISQLFFMRLVGKTPIETLIRDMQLSGSSISWPYAAGQ

>ENSXETP00000006874.1_Nr2f1_Xenopus_tropicalis

MAMVVSSWRDPQEDVAGGNPGGPNPGAREQQQAPSAAPHTPQTPSQPGPPSTPGAAGDKGQQGSGQSQQQHIE**CVVCGDKSSGKHYGQFTCEGC**KSFFKRSVRRNLTYT**CRANRNCPIDQHHRNQCQYC**RLKKCLKVGMRREAVQRGRMPPTQPNPGQYALTNGDPLNGHCYLSGYISLLLRAEPYPTSRYGSQCMQPNNIMGIENICELAARLLFSAVEWARNIPFFPDLQITDQVALLRLTWSELFVLNAAQCSMPLHVAPLLAAAGLHASPMSADRVVAFMDHIRIFQEQVEKLKALHVDSAEYSCLKAIVLFTSDACGLSDAAHIESLQEKSQCALEEYVRSQYPNQPSRFGKLLLRLPSLRTVSSSVIEQLFFVRLVGKTPIETLIRDMLLSGSSFNWPYMPIQCS

>ENSXETP00000031045.1_Nr2f2_Xenopus_tropicalis

MAMVVGAWRDPQDDMPGTQPSQAPPGQGPNGAPHTPQTPGQGVPSTTPAQSNPSSQPSQNQGEKQQQQQHIE**CVVCGDKSSGKHYGQFTCEGC**KSFFKRSVRRNLTYT**CRANRNCPIDQHHRNQCQYC**RLKKCLKVGMRREAVQRGRMPPTQPTHGQFALTNGDPLNCHSYLSGYISLLLRAEPYPTSRFGSQCMQPNNIMGIENICELAARMLFSAVEWARNIPFFPDLQITDQVALLRLTWSELFVLNAAQCSMPLHVAPLLAAAGLHASPMSADRVVAFMDHIRIFQEQVEKLKALHVDSAEYSCLKAIVLFTSDACGLSDVAHVESLQEKSQCALEEYVRSQYPNQPTRFGKLLLRLPSLRTVSSSVIEQLFFVRLVGKTPIETLIRDMLLSGSSFNWPYMSIQ

>ENSXETP00000014637.1_Nr2f5_Xenopus_tropicalis

MAMVVNPWQEDIPGVPGSQVNNPPGLCNQDPGGTPQTPTTPKGGVPGQDPVHSGDKGVPNVDCLVC**GDKSSGKHYGQFTCEGC**KSFFKRSVRRNLTYT**CRGNRDCPIDQHHRNQCQYC**RLKKCLKVGMRREAVQRGRMSHPQTSPGQYTLNNVDPYNGHSYLTGFISLLLRAEPYPTSRYGAQCLQPNNIMGIENICELAARLLFSAIEWAKNIPFFPDFQLSDQVSLLRMTWSELFVLNAAQCSMPLHVAPLLAAAGLHASPMSADRVVAFMDHIRVFQEQVEKLKALHVDSAEYSCLKAIALFTPDAVGLSDIGHVESIQEKSQCALEEYVRNQYPNQPTRFGRLLLRLPSLRIVSAPVIEQLFFVRLVGKTPIETLIRDMLLSGSSFNWPYMPMQ

>ENSXETP00000041927.1_Nr2f6_Xenopus_tropicalis

MAMVSGGWGDPNGDTNGVGKGYPRNSEEEEASPQGGMSDPEQGDEERPGIQVD**CVVCGDKSSGKHYGVFTCEGC**KSFFKRSVRRNLSYT**CRSNRDCQIDQHHRNQCQYC**RLKKCFRVGMRKEAVQRGRIPPAHSSASPTSAPGAGEYFNGQPVSELISQLLRAEPYPASRYGSQYAQQGSVMGIDNICELAARLLFSTVEWSRNIPYFPELAMADQVSLLRLSWSELFVLSAAQSALPLHMAPLLAAAGFHASPMSADRVVSFMDQIRLFQDQVEKLNRLQVDSAEYACLKAIALFTSDACGLTDPAHVESLQEKAQVALTEYVRAQYPSQPQRFGRLLLRLPALRAVPASLISQLFFMRLVGKTPIETLIRDMLLSGSSFNWPYSSGQ

>ENSGALP00000033285.3_Nr2f1_Gallus_gallus

MAMVVSSWRDPQEDVAGGTPSGPNPAAQPAREQPPQQQGGSAAPHTPQTPSQPGPPSTPGTAGDKGPGQQGSGQSQQHIE**CVVCGDKSSGKHYGQFTCEGC**KSFFKRSVRRNLTYT**CRANRNCPIDQHHRNQCQYC**RLKKCLKVGMRREAVQRGRMPPTQPNPGQYALTNGDPLNGHCYLSGYISLLLRAEPYPTSRYGSQCMQPNNIMGIENICELAARLLFSAVEWARNIPFFPDLQITDQVALLRLTWSELFVLNAAQCSMPLHVAPLLAAAGLHASPMSADRVVAFMDHIRIFQEQVEKLKALHVDSAEYSCLKAIVLFTSDACGLSDAAHIESLQEKSQCALEEYVRSQYPNQPSRFGKLLLRLPSLRTVSSSVIEQLFFVRLVGKTPIETLIRDMLLSGSSFNWPYMSIQCS

>ENSGALP00000073426.1_Nr2f2_Gallus_gallus

MQAIWDLEQGKYGFAVQRGRMPPTQPTHGQFALTNGDPLNCHSYLSGYISLLLRAEPYPTSRFGSQCMQPNNIMGIENICELAARMLFSAVEWARNIPFFPDLQITDQVALLRLTWSELFVLNAAQCSMPLHVAPLLAAAGLHASPMSADRVVAFMDHIRIFQEQVEKLKALHVDSAEYSCLKAIVLFTSDACGLSDVAHVESLQEKSQCALEEYVRSQYPNQPTRFGKLLLRLPSLRTVSSSVIEQLFFVRLVGKTPIETLIRDMLLSGSSFNWPYMSIQ

>ENSGALP00000044103.2_Nr2f6_Gallus_gallus

MAMVAGGWGEPNGGGGAAEEAASPAGGGSDAEHGEEERPGAAVD**CVVCGDKSSGKHYGVFTCEGC**KSFFKRSIRRNLSYT**CRSNRDCQIDQHHRNQCQYC**RLKKCFRVGMRKEAVQRGRIPPTHSSASPTAMPSGEYFNGQPVSELISQLLRAEPYPAARYGSQYAQQGSVMGIDNICELAARLLFSTVEWARNIPFFPELPVSDQVALLRLSWSELFVLNAAQSALPLHMAPLLAAAGFHASPMSADRVVSFMDQIRIFQDQVEKLNRLQVDSAEYSCLKAIALFTPDACGLSDPAHVEGLQEKAQVALTEYVRSQYPSQPQRFGRLLLRLPALRAVPAALISQLFFMRLVGKTPIETLIRDMLLSGSTFNWPYGTGQ

>ENSFALP00000009459.1_Nr2f1_Ficedula_albicollis

MFGYSVQRGRMPPTQPNPGQYALTNGDPLNGHCYLSGYISLLLRAEPYPTSRYGSQCMQPNNIMGIENICELAARLLFSAVEWARNIPFFPDLQITDQVALLRLTWSELFVLNAAQCSMPLHVAPLLAAAGLHASPMSADRVVAFMDHIRIFQEQVEKLKALHVDSAEYSCLKAIVLFTSDACGLSDAAHIESLQEKSQCALEEYVRSQYPNQPSRFGKLLLRLPSLRTVSSSVIEQLFFVRLCS

>ENSFALP00000010808.1_Nr2f2_Ficedula_albicollis

MAMVVGAWRDPQDDVPGAQGTQPSQAPPVQGPPAGAPHTPQTPGPGGPPSTPAQTNPPSQQNQGDKQQQQQHIE**CVVCGDKSSGKHYGQFTCEGC**KSFFKRSVRRNLSYT**CRANRNCPIDQHHRNQCQYC**RLKKCLKVGMRREAVQRGRMPPTQPTHGQFALTNGDPLNCHSYLSGYISLLLRAEPYPTSRFGSQCMQPNNIMGIENICELAARMLFSAVEWARNIPFFPDLQITDQVALLRLTWSELFVLNAAQCSMPLHVAPLLAAAGLHASPMSADRVVAFMDHIRIFQEQVEKLKALHVDSAEYSCLKAIVLFTSDACGLSDVAHVESLQEKSQCALEEYVRSQYPNQPTRFGKLLLRLPSLRTVSSSVIEQLFFVRLVGKTPIETLIRDMLLSGSSFNWPYMSIQ

>ENSFALP00000009512.1_Nr2f6_Ficedula_albicollis

SIRRNLSYTCSTNQQLQFRVWDENPLKYFGFRLKIQIRMGLRRRSTVQRGRIPPSHSSSSPNPLPNAEFSNGQPVSELISQLLRAEPYPAARYGSQYAQHGSVMGIDNICELAARLLFSTVEWARNIPFFPELPVSDQVALLRLSWSELFVLNAAQSALPLHMAPLLAAAGFHASPMSADRVVAFMDQIRVFQEQVDKLNRLQVDSAEYSCLKAIALFTPDACGLSDTAHVEGHSPITPEILGGGGGGGGGGGGGGFGRLLLRLPALRAVPASLISQLFFMRLVGKTPIETLIRDMLLSGSTFNWPYGAGQ

>ENSPSIP00000011312.1_Nr2f1_Pelodiscus_sinensis

LSTIRAQPCAPQRAQSAGGGSGGQPRHQQTPSQPGPPSTPGTAGDKGQSQQGSGQSQQHIE**CVVCGDKSSGKHYGQFTCEGC**KSFFKRSVRRNLTYT**CRANRNCPIDQHHRNQCQYC**RLKKCLKVGMRREDGFPSIPPPSFPPYALFPALLTGILLDAHFLSFSLSFCXGPHPSPTPRSGSLCMQPNNIMGIENICELAARLLFSAVEWARNIPFFPDLQITDQVALLRLTWSELFVLNAAQCSMPLHVAPLLAAAGLHASPMSADRVVAFMDHIRIFQEQVEKLKALHVDSAEYSCLKAIVLFTSDACGLSDAAHIESLQEKSQCALEEYVRSQYPNQPSRFGKLLLRLPSLRTVSSSVIEQLFFVRLVGKTPIETLIRDMLLSGSSFNWPYMSIQCS

>ENSPSIP00000019330.1_Nr2f2_Pelodiscus_sinensis

MQAIWDLEQGKYGFAVQRGRMPPTQPTHGQFALTNGDPLNCHSYLSGYISLLLRAEPYPTSRFGSQCMQPNNIMGIENICELAARMLFSAVEWARNIPFFPDLQITDQVALLRLTWSELFVLNAAQCSMPLHVAPLLAAAGLHASPMSADRVVAFMDHIRIFQEQVEKLKALHVDSAEYSCLKAIVLFTSDACGLSDVAHVESLQEKSQCALEEYVRSQYPNQPTRFGKLLLRLPSLRTVSSSVIEQLFFVRLVF

>ENSPSIP00000015473.1_Nr2f6_Pelodiscus_sinensis

MAMVAGGWGDPNGDTNGVDKGYPRTSEEDSVSPQGGASDPEPGDEDKPGIQVD**CTVCGDKSSGKHYGVFTCEGC**KSFFKRSIRRNLSYT**CRSNRDCQIDQHHRNQCQYC**RLKKCFRVGMRKEAVQRGRIPPSHASTSPTAMPSGEYFNGQPVSELISQLLRAEPYPAARYGSQYAQQGSVMGIDNICELAARLLFSTVEWARNIPFFPELPVSDQVALLRLSWSELFVLNAAQSALPLHMAPLLAAAGFHAAPMSADRVVSFMDQIRIFQDQVEKLNRLQVDSAEYSCLKAIALFTPDACGLSDPAHVESLQEKAQVALTEYVRAQYPSQPQRFGRLLLRLPALRAVPASLISQLFFMRLVGKTPIETLIRDMLLSGSTFNWPYGSGQ

>XP_015282619.1_Nr2f1_Gekko_japonicus

MAMVVSSWRDAQEDVAGGGPAGAAQPGRDPQQGASAAPHTPQTPSQPGAPSTPGDKGQQQQQQQQGGGQSGQQQQQQHIE**CVVCGDKSSGKHYGQFTCEGC**KSFFKRSVRRNLTYT**CRANRNCPIDQHHRNQCQYC**RLKKCLKVGMRREAVSRGRMPPSQPNPGQYALTNGDPLNGHCYLSGYISLLLRAEPYPTSRYGSQCMQPNNIMGIENICELAARLLFSAVEWARNIPFFPDLQITDQVALLRLTWSELFVLNAAQCSMPLHVAPLLAAAGLHASPMSADRVVAFMDHIRIFQEQVEKLKALHVDSAEYSCLKAIVLFTSDACGLSDAAHIESLQEKSQCALEEYVRSQYPNQPSRFGKLLLRLPSLRTVSSSVIEQLFFVRLVGKTPIETLIRDMLLSGSSFNWPYMSIQCS

>XP_015273868.1_Nr2f2_Gekko_japonicus

MAMVVGAWRDPQDDVAGLQGTQPSQAPPAPGPPTGAPHPPQTPGQGGPPTTPAQPNAGSQQSQGDKATQQQQHIE**CVVCGDKSSGKHYGQFTCEGC**KSFFKRSVRRNLSYT**CRANRNCPIDQHHRNQCQYC**RLKKCLKVGMRREAVQRGRMPPTQPTHGQFALTNGDPLNCHSYLSGYISLLLRAEPYPTSRFGSQCMQPNNIMGIENICELAARMLFSAVEWARNIPFFPDLQITDQVALLRLTWSELFVLNAAQCSMPLHVAPLLAAAGLHASPMSADRVVAFMDHIRIFQEQVEKLKALHVDSAEYSCLKAIVLFTSDACGLSDVAHVESLQEKSQCALEEYVRSQYPNQPTRFGKLLLRLPSLRTVSSSVIEQLFFVRLVGKTPIETLIRDMLLSGSSFNWPYMAIQ

>XP_015270008.1_Nr2f5_Gekko_japonicus

MDCLV**CGDKASGKHYGQFTCEGC**KSFFKRSVRRNLSYT**CRASRQCPVDQHHRNQC**QHCRLTKCLKVGMRREAVQRGRLAHAQPGPGQYPLANGDPYGCPSYLTGFISLLLRAEPYPASRYGPQCLQAGNIMGIESICEMAARLLFSAIEWAKNIPFFPDFQLADQVCLLRMTWSELFVLNAAQCAMPLHVAPLLAAAGLHASPMSAERVVAFMDHIRVFQEQVEKLKALHVDAAEYACLKALALFTPDAVGLTDWCHVESVQEKSQCALEEYVRNQYPNQPSRFGRLLLRMPSLRIVSAPVIEQLFFVRLVGKTPIETLIRDMLLSGSSFSWPYIPMQ

>XP_015262308.1_Nr2f6_Gekko_japonicus

MAMVTGGWGDPNGDANGVEKGYPRNSEDDSASPQGGTSDQEPGDEDKAGLQVD**CVVCGDKSSGKHYGVFTCEGC**KSFFKRSIRRNLSYT**CRSNRDCQIDQHHRNQCQYC**RLKKCFRVGMRKEAVQRGRIPPTHSSASPNAMPSGEYYNGQPVSELISQLLRAEPYPTARFSSQYAQQGSVMGIDNICELAARLLFSTVEWARNIPFFPDLPVSDQVALLRLSWSELFVLNAAQSALPLHMAPLLAAAGFHASPMSADRVVSFMDQIRIFQDQVEKLNRLQVDSAEYSCLKAIALFTPDACGLSDPAHVESLQEKAQVALTEYVRSQYPSQPQRFGRLLLRLPALRAVPASLISQLFFMRLVGKTPIETLIRDMLLSGSTFNWPYGTGQ

>ENSP00000495420.1_NR2F1_Homo_sapiens

MAMVVSSWRDPQDDVAGGNPGGPNPAAQAARGGGGGAGEQQQQAGSGAPHTPQTPGQPGAPATPGTAGDKGQGPPGSGQSQQHIE**CVVCGDKSSGKHYGQFTCEGC**KSFFKRSVRRNLTYT**CRANRNCPIDQHHRNQCQYC**RLKKCLKVGMRREAVQRGRMPPTQPNPGQYALTNGDPLNGHCYLSGYISLLLRAEPYPTSRYGSQCMQPNNIMGIENICELAARLLFSAVEWARNIPFFPDLQITDQVSLLRLTWSELFVLNAAQCSMPLHVAPLLAAAGLHASPMSADRVVAFMDHIRIFQEQVEKLKALHVDSAEYSCLKAIVLFTSDACGLSDAAHIESLQEKSQCALEEYVRSQYPNQPSRFGKLLLRLPSLRTVSSSVIEQLFFVRLVGKTPIETLIRDMLLSGSSFNWPYMSIQCS

>ENSP00000377721.3_NR2F2_Homo_sapiens

MAMVVSTWRDPQDEVPGSQGSQASQAPPVPGPPPGAPHTPQTPGQGGPASTPAQTAAGGQGGPGGPGSDKQQQQQHIE**CVVCGDKSSGKHYGQFTCEGC**KSFFKRSVRRNLSYT**CRANRNCPIDQHHRNQCQYC**RLKKCLKVGMRREAVQRGRMPPTQPTHGQFALTNGDPLNCHSYLSGYISLLLRAEPYPTSRFGSQCMQPNNIMGIENICELAARMLFSAVEWARNIPFFPDLQITDQVALLRLTWSELFVLNAAQCSMPLHVAPLLAAAGLHASPMSADRVVAFMDHIRIFQEQVEKLKALHVDSAEYSCLKAIVLFTSDACGLSDVAHVESLQEKSQCALEEYVRSQYPNQPTRFGKLLLRLPSLRTVSSSVIEQLFFVRLVGKTPIETLIRDMLLSGSSFNWPYMAIQ

>ENSP00000291442.2_NR2F6_Homo_sapiens

MAMVTGGWGGPGGDTNGVDKAGGYPRAAEDDSASPPGAASDAEPGDEERPGLQVD**CVVCGDKSSGKHYGVFTCEGC**KSFFKRSIRRNLSYT**CRSNRDCQIDQHHRNQCQYC**RLKKCFRVGMRKEAVQRGRIPHSLPGAVAASSGSPPGSALAAVASGGDLFPGQPVSELIAQLLRAEPYPAAAGRFGAGGGAAGAVLGIDNVCELAARLLFSTVEWARHAPFFPELPVADQVALLRLSWSELFVLNAAQAALPLHTAPLLAAAGLHAAPMAAERAVAFMDQVRAFQEQVDKLGRLQVDSAEYGCLKAIALFTPDACGLSDPAHVESLQEKAQVALTEYVRAQYPSQPQRFGRLLLRLPALRAVPASLISQLFFMRLVGKTPIETLIRDMLLSGSTFNWPYGSGQ

>ENSOANP00000038951.1_Nr2f1_Ornithorhynchus_anatinus

MAMVVSSWRDPQEDVAGGNPGGPNPAAQPARAEQQGQQQQQQGQQGQQGQQQQAGSAAPHTPQTPSQPGPPSTPGTAGDKGQPGSGQSQQHIE**CVVCGDKSSGKHYGQFTCEGC**KSFFKRSVRRNLTYT**CRANRNCPIDQHHRNQCQYC**RLKKCLKVGMRREAVQRGRMPPTQPNPGQYALTNGDPLNGHCYLSGYISLLLRAEPYPTSRYGSQCMQPNNIMGIENICELAARLLFSAVEWARNIPFFPDLQITDQVALLRLTWSELFVLNAAQCSMPLHVAPLLAAAGLHASPMSADRVVAFMDHIRIFQEQVEKLKALHVDSAEYSCLKAIVLFTSDACGLSDAAHIESLQEKSQCALEEYVRSQYPNQPSRFGKLLLRLPSLRTVSSSVIEQLFFVRLVGKTPIETLIRDMLLSGSSFNWPYMSIQCS

>ENSOANP00000014984.2_Nr2f2_Ornithorhynchus_anatinus

MAMVVGAWRDPQDDVPGNQGTQPSQAPPVQGPPAGAPHTPQTPGQGGPPSTPAQTNQPSQQNQAGDKQQQQQHIE**CVVCGDKSSGKHYGQFTCEGC**KSFFKRSVRRNLSYT**CRANRNCPIDQHHRNQCQYC**RLKKCLKVGMRREAVQRGRMPPTQPTHGQFALTNGDPLNCHSYLSGYISLLLRAEPYPTSRFGSQCMQPNNIMGIENICELAARMLFSAVEWARNIPFFPDLQITDQVALLRLTWSELFVLNAAQCSMPLHVAPLLAAAGLHASPMSADRVVAFMDHIRIFQEQVEKLKALHVDSAEYSCLKAIVLFTSDACGLSDVAHVESLQEKSQCALEEYVRSQYPNQPTRFGKLLLRLPSLRTVSSSVIEQLFFVRLISDVMENQNGQNEDLEEE

>ENSOANP00000016523_Nr2f6_Ornithorhynchus_anatinus

MFVTHLR**SNRDCQIDQHHRNQCQYC**RLKKCFRVGMRKEAAVQRGRIPHSHSSTSPTALPSGEYFNGQPVSELISQLLRAEPYPAARYGSQYAQQGSVMGIDNICELAARLLFSTVEWARNIPFFPELPVSDQVSLLRLSWSELFVLNAAQSALPLHMAPLLAAAGFHASPMSADRVVSFMDQIRVFQDQVEKLSRLQVDSAEYSCLKAIALFTPDACGLSDPAHVESLQEKAQVALTEYVRAQYPSQPQRFGRLLLRLPALRAVPASLISQLFFMRLVGKTPIETLIRDMLLSGSTFNWPYTTGQ

>ENSMUSP00000089036.6_Nr2f1_Mus_musculus

MAMVVSSWRDPQDDVAGGNPGGPNPAAQAARGGGGGEQQQAGSGAPHTPQTPGQPGAPATPGTAGDKGQGPPGSGQSQQHIE**CVVCGDKSSGKHYGQFTCEGC**KSFFKRSVRRNLTYT**CRANRNCPIDQHHRNQCQYC**RLKKCLKVGMRREAVQRGRMPPTQPNPGQYALTNGDPLNGHCYLSGYISLLLRAEPYPTSRYGSQCMQPNNIMGIENICELAARLLFSAVEWARNIPFFPDLQITDQVSLLRLTWSELFVLNAAQCSMPLHVAPLLAAAGLHASPMSADRVVAFMDHIRIFQEQVEKLKALHVDSAEYSCLKAIVLFTSDACGLSDAAHIESLQEKSQCALEEYVRSQYPNQPSRFGKLLLRLPSLRTVSSSVIEQLFFVRLVGKTPIETLIRDMLLSGSSFNWPYMSIQCS

>ENSMUSP00000086993.5_Nr2f2_Mus_musculus

MQAVWDLEQGKYGFAVQRGRMPPTQPTHGQFALTNGDPLNCHSYLSGYISLLLRAEPYPTSRFGSQCMQPNNIMGIENICELAARMLFSAVEWARNIPFFPDLQITDQVALLRLTWSELFVLNAAQCSMPLHVAPLLAAAGLHASPMSADRVVAFMDHIRIFQEQVEKLKALHVDSAEYSCLKAIVLFTSDACGLSDVAHVESLQEKSQCALEEYVRSQYPNQPTRFGKLLLRLPSLRTVSSSVIEQLFFVRLVGKTPIETLIRDMLLSGSSFNWPYMAIQ

>ENSMUSP00000002466.8_Nr2f6_Mus_musculus

MAMVTGGWGDPGGDTNGVDKAGGSYPRATEDDSASPPGATSDAEPGDEERPGLQVD**CVVCGDKSSGKHYGVFTCEGC**KSFFKRSIRRNLSYT**CRSNRDCQIDQHHRNQCQYC**RLKKCFRVGMRKEAVQRGRIPHALPGPAACSPPGATGVEPFTGPPVSELIAQLLRAEPYPAAGRFGGGGAVLGIDNVCELAARLLFSTVEWARHAPFFPELPAADQVALLRLSWSELFVLNAAQAALPLHTAPLLAAAGLHAAPMAAERAVAFMDQVRAFQEQVDKLGRLQVDAAEYGCLKAIALFTPDACGLSDPAHVESLQEKAQVALTEYVRAQYPSQPQRFGRLLLRLPALRAVPASLISQLFFMRLVGKTPIETLIRDMLLSGSTFNWPYGSG

>ENSSSCP00000015055.3_Nr2f1_Sus_scrofa

MAMVVSSWRDPQDDVAGGNPGGPNPAAQAARGGGGAGEQQQQAGSGAPHTPQTPGQPGAPATPGTAGDKGQGPPGSGQSQQHIE**CVVCGDKSSGKHYGQFTCEGC**KSFFKRSVRRNLTYT**CRANRNCPIDQHHRNQCQYC**RLKKCLKVGMRREAVQRGRMPPTQPNPGQYALTNGDPLNGHCYLSGYISLLLRAEPYPTSRYGSQCMQPNNIMGIENICELAARLLFSAVEWARNIPFFPDLQITDQVSLLRLTWSELFVLNAAQCSMPLHVAPLLAAAGLHASPMSADRVVAFMDHIRIFQEQVEKLKALHVDSAEYSCLKAIVLFTSDACGLSDAAHIESLQEKSQCALEEYVRSQYPNQPSRFGKLLLRLPSLRTVSSSVIEQLFFVRLCS

>ENSSSCP00000002449.2_Nr2f2_Sus_scrofa

MAMVVSTWRDPQDEVPGSQGSQASQAPPVPGPPPGAPHTPQTPGQGGPASTPAQTAAGGQGGPGGPGGDKQQQQQHIE**CVVCGDKSSGKHYGQFTCEGC**KSFFKRSVRRNLSYT**CRANRNCPIDQHHRNQCQYC**RLKKCLKVGMRREAVQRGRMPPTQPTHGQFALTNGDPLNCHSYLSGYISLLLRAEPYPTSRFGSQCMQPNNIMGIENICELAARMLFSAVEWARNIPFFPDLQITDQVALLRLTWSELFVLNAAQCSMPLHVAPLLAAAGLHASPMSADRVVAFMDHIRIFQEQVEKLKALHVDSAEYSCLKAIVLFTSDACGLSDVAHVESLQEKSQCALEEYVRSQYPNQPTRFGKLLLRLPSLRTVSSSVIEQLFFVRLVGKTPIETLIRDMLLSGSSFNWPYMAIQ

>ENSSSCP00000014750.3_Nr2f6_Sus_scrofa

MAMVTGGWGGPGGGGGGGDTNGVDKAGGYPRAAEEDSASPPGAASDAEPGDEERPGLQVD**CVVCGDKSSGKHYGVFTCEGC**KSFFKRSIRRNLSYT**CRSNRDCQIDQHHRNQCQYC**RLKKCFRVGMRKEAVQRGRIPHSLPGAVAASSGSPPGSALAAAGGDLFPGQPVSELIAQLLRAEPYPAAAGRFGAGAAGAFGAGGGAAGAVLGIDNVCELAARLLFSTVEWARHAPFFPELPVADQVALLRLSWSELFVLNAAQAALPLHTAPLLAAAGLHAAPMAAERAVAFMDQVRAFQEQVDKLGRLQVDSAEYGCLKAIALFTPDACGLSDPAHVESLQEKAQVALTEYVRAQYPSQPQRFGRLLLRLPALRAVPASLISQLFFMRLVGKTPIETLIRDMLLSGSTFNWPYGSGQ

>ENSMEUP00000002917.1_Nr2f1_Notamacropus_eugenii

VQRGRMPPTQPNPGQYALTNGDPLNGHCYLSGYISLLLRAEPYPTSRYGSQCMQPNNIMGIENICELAARLLFSAVEWARNIPFFPDLQITDQVALLRLTWSELFVLNAAQCSMPLHVAPLLAAAGLHASPMSADRVVAFMDHIRIFQEQVEKLKALHVDSAEYSCLKAIVLFTSDACGLSDAAHIESLQEKSQCALEEYVRSQYPNQPSRFGKLLLRLPSLRTVSSSVIEQLFFVRLVGKTPIETLIRDMLLSGSSFNWPYMSIQCS

>ENSMEUP00000000478.1_Nr2f2_Notamacropus_eugenii

MAMVVGAWRDPQDDVPGNQGTQPSQAPPVQGPPAGAPHTPQTPGQGGPPSTPVQTNQPSQQSQAGDKQQQQQHIE**CVVCGDKSSGKHYGQFTCEGC**KSFFKRSVRRNLSYT**CRANRNCPIDQHHRNQCQYC**RLKKCLKVGMRREAVQRGRMPPTQPTHGQFALTNGDPLNCHSYLSGYISLLLRAEPYPTSRFGSQCMQPNNIMGIENICELAARMLFSAVEWARNIPFFPDLQITDQVALLRLTWSELFVLNAAQCSMPLHVAPLLAAAGLHASPMSADRVVAFMDHIRIFQEQVEKLKALHVDSAEYSCLKAIVLFTS

>ENSMEUP00000012741.1_Nr2f6_Notamacropus_eugenii

**SNRDCQIDQHHRNQCQYC**RLKKCFRVGMRKEAVQRGRIPHTHSGGSPTALEVGVEYFNGQPVSELISQLLRAEPYPAARYGSVCPAAAVLGIDNICELAARLLFSTVEWARNIPFFPELPVADQVALLRLSWSELFVLNAAQSALPLHMAPLLAAAGFHAAPMAADRVVSFMDQIRVFQEQVDKLNRLQVDSAEYSCLKAIALFTPDACGLSDPAHVESLQEKAQVALTEYVRAQYPSQPQRFGRLLLRLPALRAVPAALISQLFFMRLVGKTPIETLIRDMLLSGSTFNWPYAAGQ

>XP_037739369.1_Nr2f5_Chelonia_mydas

MAMVVNPWQEEIPGGPPGAQPPPGLPCAPEPGVAPPPPGPPKGAVAAGPPEAGEKGGAPPSVD**CMVCGDKSSGKHYGQFTCEGC**KSFFKRSVRRNLSYT**CRSNRECPIDQHHRNQCQHC**RLKKCLKVGMRREAVQRGRMAHTQTSPGQYLLSNGDPYNGHSYLTGFISLLLRAEPYPTSRYGAQCLQSNIMGIENICELAARLLFSAIEWAKNIPFFPDFQLSDQVSLLRMTWSELFVLNAAQCSMPLHVAPLLAAAGLHASPMSADRVVAFMDHIRVFQEQVEKLKALHVDSAEYSCLKAIALFTPDAVGLSDLGPVESIQEKSQCALEEYVRNQYPNQPSRFGRLLLRMPSLRIVSAPVIEQLFFVRLVGKTPIETLIRDMLLSGSSFNWPYIPMQ

>XP_037759164.1_Nr2f1_Chelonia_mydas

MAMVVSSWRDPQEDVAGGNPSGPNPAAQPARDQQQQQQAASAAPHTPQTPSQPGPPSTPGTAGDKGQSQQGSGQSQQHIE**CVVCGDKSSGKHYGQFTCEGC**KSFFKRSVRRNLTYT**CRANRNCPIDQHHRNQCQYC**RLKKCLKVGMRREAVQRGRMPPTQPNPGQYALTNGDPLNGHCYLSGYISLLLRAEPYPTSRYGSQCMQPNNIMGIENICELAARLLFSAVEWARNIPFFPDLQITDQVALLRLTWSELFVLNAAQCSMPLHVAPLLAAAGLHASPMSADRVVAFMDHIRIFQEQVEKLKALHVDSAEYSCLKAIVLFTSDACGLSDAAHIESLQEKSQCALEEYVRSQYPNQPSRFGKLLLRLPSLRTVSSSVIEQLFFVRLVGKTPIETLIRDMLLSGSSFNWPYMSIQCS

>XP_007060431.2_Nr2f2_Chelonia_mydas

MAMVVGAWRDPQDDVPGAQGTQPAQAPPVQGPPAGAPHTPQTPGQGGPPSTPAQTNQPSQQSQGGDKQQQQQHIE**CVVCGDKSSGKHYGQFTCEGC**KSFFKRSVRRNLSYT**CRANRNCPIDQHHRNQCQYC**RLKKCLKVGMRREAVQRGRMPPTQPTHGQFALTNGDPLNCHSYLSGYISLLLRAEPYPTSRFGSQCMQPNNIMGIENICELAARMLFSAVEWARNIPFFPDLQITDQVALLRLTWSELFVLNAAQCSMPLHVAPLLAAAGLHASPMSADRVVAFMDHIRIFQEQVEKLKALHVDSAEYSCLKAIVLFTSDACGLSDVAHVESLQEKSQCALEEYVRSQYPNQPTRFGKLLLRLPSLRTVSSSVIEQLFFVRLVGKTPIETLIRDMLLSGSSFNWPYMSIQ

>XP_007072117.1_Nr2f6_Chelonia_mydas

MAMVAGGWGDPNGDTNGVDKGYPRTSEEDSVSPQGGVSDQEHGDEDKPGIQV**DCTVCGDKSSGKHYGVFTCEGC**KSFFKRSIRRNLSYT**CRSNRDCQIDQHHRNQCQYC**RLKKCFRVGMRKEAVQRGRIPPTHSSTSPSAMPSGEYYNGQPVSELISQLLRAEPYPTARYGSQYAQQGSVMGIDNICELAARLLFSTVEWARNIPFFPELPVSDQVALLRLSWSELFVLNAAQSALPLHMAPLLAAAGFHASPMSAERVVSFMDQIRIFQDQVEKLNRLQVDSAEYSCLKAIALFTPDACGLSDPAHVESLQEKAQVALTEYVRAQYPSQPQRFGRLLLRLPALRAVPASLISQLFFMRLVGKTPIETLIRDMLLSGSTFNWPYGTGQ

>XP_041037625.1_Nr2f5_Carcharodon_carcharias

MIRNSRRDSMQEASFTPRPHHSAEELKDTSPGDLLPSPLGQDLPQLEKLQQQPTVE**CMVCGDKSSGKHYGQFTCEGC**KSFFKRCVRRNLSYS**CRGSRNCPIDQHHRNECQHC**RFQKCLKVGMRREVSSLFTAPVMRGRLSHTQSNPGQYSLSNGDGFTNPSYLSSFISLLLRAEPYSMSRYGSPCMQPSSVMGVESICELAARLLFSAVEWARNIPFFSDLQISDQVGLLRLTWSELFVLNAAQCSMPLHVAPLLAAAGLHASPMSGERVVAFMDHIRVFQEQVEKLKLLHVDAAEFSCLKAIVLFTSDACGLSDPSQVESLQEKCQCALEDYSHNQYPNQPNRFGKLLLRLPSLRVVSSPIIEQLFFVRLVGKTPIETLIRDMLLSGSSFSWPYLPVQ

>XP_041030925.1_Nr2f2_Carcharodon_carcharias

MAMVVSTWRDPQDDVAGAQGTQPPPPQQGPQPPSGAPLTPQTPGQPGAPGTPAQSNQQNQQQGEKQQQHIE**CVVCGDKSSGKHYGQFTCEGC**KSFFKRSVRRNLTYT**CRANRNCPIDQHHRNQCQYC**RLKKCLKVGMRREVCSFRTAAVQRGRMPPTQPTPGQFALTNGDPLNCHSYLSGYISLLLRAEPYPTSRYGSQCMQPNNIMGIENICELAARLLFSAVEWARNIPFFPDLQITDQVALLRLTWSELFVLNAAQCSMPLHVAPLLAAAGLHASPMSADRVVAFMDHIRIFQEQVEKLKALHVDSAEYSCLKAIVLFTSDACGLSDVAHIESLQEKSQCALEEYVRSQYPNQPTRFGKLLLRLPSLRTVSSSVIEQLFFVRLVGKTPIETLIRDMLLSGSSFNWPYMSIQ

>XP_041042847.1_Nr2f1_Carcharodon_carcharias

MAMVVSTWRDPQDDVAGAQGGQSAQTQPGQQQQQQQQQAASGAPHTPQTPGQPGPPSTPLGGSSQSVQPGGEKQQPCQQQQQQHIE**CVVCGDKSSGKHYGQFTCEGC**KSFFKRSVRRNLTYT**CRANRNCPIDQHHRNQCQYC**RLKKCLKVGMRREAVQRGRMPPTQPNPGQYALTNGDPLNGHCYLSGYISLLLRAEPYPTSRYGSQCMQPNNIMGIENICELAARLLFSAVEWARNIPFFPDLQITDQVALLRLTWSELFVLNAAQCSMPLHVAPLLAAAGLHASPMSADRVVAFMDHIRIFQEQVEKLKALHVDSAEYSCLKAIVLFTSDACGLSDAAHIESLQEKSQCALEEYVRSQYPNQPSRFGKLLLRLPSLRTVSSSVIEQLFFVRLVGKTPIETLIRDMLLSGSSFNWPYMSIQ

>XP_041061550.1_Nr2f6_Carcharodon_carcharias

MAMAASDWGDPRDDSKPYPHNGGEESLESPAPSSSQTGGSEPELGEPDRQGLQVD**CVVCGDRASGKHYGQFTCEGC**KSFFKRSIRRNLSYT**CRSNRDCQIDQHHRNQCQYC**RLRKCFKVGMKREAVQRGRIPPAQANPGELSLSSGDHLNGGNISGLISLLLRAEPYPTSRFSTQCTQYNLMSIDNICELAARLLFSAVEWARNIPFFHELQISDQVALLRLSWSELFVLNAAQSSLPLHMAPLLAAAGLHSTQMSADRVVSFMDQIRIFQDQVEKLKVLQVDSAEYSCLKAIALYTPDASGLSEPAHIESLQEKVQESLAEYVRCQYPPQPQRFGKLLLRLPALRAVPASLIGQLFFMRLVGKTPIETLIRDMLLSGGSFNWPYMPTQ

>XP_020392814.1_Nr2f1_Rhincodon_typus

MAMVVSSWRDPQDDVAGAQGGQSAQTQPSQQQPQQQQAASAAPHTPQTPGQPGPPSTPLGGSSHSVQPGGEKQQPCQQQQQHIE**CVVCGDKSSGKHYGQFTCEGC**KSFFKRSVRRNLTYT**CRANRNCPIDQHHRNQCQYC**RLKKCLKVGMRREAVQRGRMPPTQPNPGQYALTNGDPLNGHCYLSGYISLLLRAEPYPTSRYGSQCMQPNNIMGIENICELAARLLFSAVEWARNIPFFPDLQITDQVALLRLTWSELFVLNAAQCSMPLHVAPLLAAAGLHASPMSADRVVAFMDHIRIFQEQVEKLKALHVDSAEYSCLKAIVLFTSDACGLSDAAHIESLQEKSQCALEEYVRSQYPNQPSRFGKLLLRLPSLRTVSSSVIEQLFFVRLVGKTPIETLIRDMLLSGSSFNWPYMSIQ

>XP_020382960.1_Nr2f2_Rhincodon_typus

MAMVVSTWRDPQDDVAGAQGTQPPPPQQGPQPPSGAPLTPQTPGQPGAPGTPAQPNQQSQQQGEKQQQHIE**CVVCGDKSSGKHYGQFTCEGC**KSFFKRSVRRNLTYT**CRANRNCPIDQHHRNQCQYC**RLKKCLKVGMRREAVQRGRMPPTQPTPGQFALTNGDPLNCHSYLSGYISLLLRAEPYPTSRYGSQCMQPNNIMGIENICELAARLLFSAVEWARNIPFFPDLQITDQVALLRLTWSELFVLNAAQCSMPLHVAPLLAAAGLHASPMSADRVVAFMDHIRIFQEQVEKLKALHVDSAEYSCLKAIVLFTSDACGLSDVAHIESLQEKSQCALEEYVRSQYPNQPTRFGKLLLRLPSLRTVSSSVIEQLFFVRLVGKTPIETLIRDMLLSGSSFNWPYMSIQ

>XP_020380563.1_Nr2f5_Rhincodon_typus

MIRNVWRDPVQEAPLTPRPHLNAEEQKTASPEDVLPCSAGQQDMPQLEKLQQQPGVE**CMVCGDKSSGKHYGQFTCEGC**KSFFKRSIRRNLSYS**CRGSRNCPVDQHHRNQCQHC**RFKKCLKVGMRREAVQRGRLAHTQSNHGQYSLNNGDGFTSPSYLSGFISLLQRAEPYAMSRYGSQCMQPSSMMGIENICEFAARLLFSAVEWARNIPFFPDLQISDQVGLLRLTWSELFVLNAAQCSMPLHVAPLLAAAGLHASPMSAERVVAFMDHIRIFQEQVEKLKLLHMDSAEYSCLKAIVLFTSDACGLSDPTHVENLQEKCQCALEDYTRNQYPNQPNRFGKLLLRLPSLRVVSSPVIEQLFFVRLVGKTPIETLIRDMLLSGASFTWPYIPIQ

>XP_006264114.1_Nr2f1_Alligator_mississippiensis

MAMVVSSWRDPQEDVAGGNPSGPNPAAQPAREQQQQQAASAAPHTPQTPSQPGPPSTPGTAGDKGQGQQGSGQSQQHIE**CVVCGDKSSGKHYGQFTCEGC**KSFFKRSVRRNLTYT**CRANRNCPIDQHHRNQCQYC**RLKKCLKVGMRREAVQRGRMPPTQPNPGQYALTNGDPLNGHCYLSGYISLLLRAEPYPTSRYGSQCMQPNNIMGIENICELAARLLFSAVEWARNIPFFPDLQITDQVALLRLTWSELFVLNAAQCSMPLHVAPLLAAAGLHASPMSADRVVAFMDHIRIFQEQVEKLKALHVDSAEYSCLKAIVLFTSDACGLSDAAHIESLQEKSQCALEEYVRSQYPNQPSRFGKLLLRLPSLRTVSSSVIEQLFFVRLVGKTPIETLIRDMLLSGSSFNWPYMSIQCS

>XP_006270791.1_Nr2f2_Alligator_mississippiensis

MAMVVGTWRDPQDDVPGAQGTQPSQAPPVQGPPAGAPHTPQTPVQGGPPPSTPVQTNQPSQQSTGEKQQQQQHIE**CVVCGDKSSGKHYGQFTCEGC**KSFFKRSVRRNLSYT**CRANRNCPIDQHHRNQCQYC**RLKKCLKVGMRREAVQRGRMPPTQPTHGQFALTNGDPLNCHSYLSGYISLLLRAEPYPTSRFGSQCMQPNNIMGIENICELAARMLFSAVEWARNIPFFPDLQITDQVALLRLTWSELFVLNAAQCSMPLHVAPLLAAAGLHASPMSADRVVAFMDHIRIFQEQVEKLKALHVDSAEYSCLKAIVLFTSDACGLSDVAHVESLQEKSQCALEEYVRSQYPNQPTRFGKLLLRLPSLRTVSSSVIEQLFFVRLVGKTPIETLIRDMLLSGSSFNWPYMSIQ

>XP_019342006.1_Nr2f5_Alligator_mississippiensis

MAHTQNSPGQYPLNNGDPYNGYSYLTGFISLLLRAEPYPTSRYGAQCLQPNNIMGIENICELAARLLFSAIEWAKNIPFFPDFQLTDQVSLLRMTWSELFVLNAAQCSMPLHVAPLLAAAGLHASPMPADRVVAFMDHIRVFQEQVEKLKALHVDSAEYSCLKAIALFTPDAMGLADLGHVESVQEKSQCALEEYVRTQYPSQPSRFGRLLLRLPSLRIVSAPVIEQLFFVRLVGKTPIETLIRDMLLSGSSFNWPYIPMQ

>XP_014459987.1_Nr2f6_Alligator_mississippiensis

MAMVAGGWGEPGGDTNGVDKGYPRGSEEESVSPPGGASDPEHGDEDKPGIQVD**CVVCGDKSSGKHYGVFTCEGC**KSFFKRSIRRNLSYT**CRSNRDCQIDQHHRNQCQYC**RLKKCFRVGMRKEAVQRGRIPPTHSNTSPTAMPSGEYFNGQPVSELISQLLRAEPYPAARYGSQYAQQGSIMGIDNICELAARLLFSTVEWARNIPFFPELPVSDQVALLRLSWSELFVLNAAQSALPLHMAPLLAAAGFHASPMSADRVVSFMDQIRIFQDQVEKLNRLQVDSAEYSCLKAIALFTPDACGLSDPVHVESLQEKAQVALTEYVRAQYPSQPQRFGRLLLRLPALRAVPASLISQLFFMRLVGKTPIETLIRDMLLSGSTFNWPYGAGQ

>ENSONIP00000029659.1_Nr2f5_Oreochromis_niloticus

MAMVVNQWPENISADPGSQLQICGQEPGGAPGTPNGSTPGNDALSGDKIPNVDC**MVCGDKSSGKHYGQFTCEGC**KSFFKRSVRRNLTYT**CRGNRDCPIDQHHRNQCQYC**RLKKCLKVGMRREAVQRGRTSNSQSSPGQYLTNGTDPYNGQPYLSGFISLLLRAEPYPTSRYGAQCMQGNNLMGIENICELAARLLFSAVEWAKNIPFFPDLQLMDQVALLRMSWSELFVLNAAQCSMPLHVAPLLAAAGLHASPMSAERVVAFMDHIRVFQEQVEKLKALQVDTAEYSCLKSIVLFTSDAMGLSDVAHVESIQEKSQCALEEYVRNQYPSQPNRFGRLLLRLPSLRIVSSPVIEQLFFVRLVGKTPIETLLRDMLLSGSSYNWPYMPTVQRERPISLHYNENGP

>ENSMZEP00005021820.1_Nr2f5_Maylandia_zebra

MAMVVNQWPENISADPGSQLQICGQEPGGAPGTPNGSTPGNDALSGDKIPNVD**CMVCGDKSSGKHYGQFTCEGC**KSFFKRSVRRNLTYT**CRGNRDCPIDQHHRNQCQYC**RLKKCLKVGMRREAVQRGRTSNSQSSPGQYLTNGTDPYNGQPYLSGFISLLLRAEPYPTSRYGAQCMQGNNLMGIENICELAARLLFSAVEWAKNIPFFPDLQLMDQVALLRMSWSELFVLNAAQCSMPLHVAPLLAAAGLHASPMSAERVVAFMDHIRVFQEQVEKLKALQVDTAEYSCLKSIVLFTSDAMGLSDVAHVESIQEKSQCALEEYVRNQYPSQPNRFGRLLLRLPSLRIVSSPVIEQLFFVRLVGKTPIETLLRDMLLSGSSYNWPYMPTVQRERPISLHYNENGP

>XP_041708728.1_Nr2f5a_Coregonus_clupeaformis

MAMVVNQWQENISADPGSQLQICSQEPGGTPGTPSGSTPGNDALSGDKIPNVD**CMVCGDKSSGKHYGQFTCEGC**KSFFKRSVRRNLSYT**CRGNRDCPIDQHHRNQCQYC**RLKKCLKVGMRREAVQRGRMSNSQSSPGQYLTNGSDPYNGQPYLSGFISLLLRAEPYPTSRYGSQCMQGNNLMGIENICELAARLLFSAVEWAKNIPFFPDLQLMDQVALLRMSWSELFVLNAAQCSMPLHVAPLLAAAGLHASPMSAERVVAFMDHIRVFQEQVEKLKVLQVDTAEYSCLKSIVLFTSDAMGLSDVAHVESIQEKSQCALEEYVRNQYPSQPNRFGRLLLRLPSLRIVSSPVIEQLFFVRLVGKTPIETLLRDMLLSGSSYNWPYMPVQRDRPISLHYNENGP

>XP_041730074.1_Nr2f5b_Coregonus_clupeaformis

MAMVVNQWQENISADPGSQLQICSQEPGGTPGTPSGSTPGNDALSGDKIPNVD**CMVCGDKSSGKHYGQFTCEGC**KSFFKRSVRRNLSYT**CRGNRDCPIDQHHRNQCQYC**RLKKCLKVGMRREAVQRGRMSNSQSSPGQYLTNGSDPYNGQPYLSGFISLLLRAEPYPTSRYGSQCMQGNNLMGIENICELAARLLFSAVEWAKNIPFFPDLQLMDQVALLRMSWSELFVLNAAQCSMPLHVAPLLAAAGLHASPMSAERVVAFMDHIRVFQEQVEKLKVLQVDTAEYSCLKSIVLFTSDAMGLSDVAHVESIQEKSQCALEEYVRNQYPSQPNRFFGRLLLRLPSLRIVSSPVIEQLFFVRLVGKTPIETLLRDMLLSGSSYNWPYMPVQRDRPISLHYNENGP

>XP_029535578.1_Nr2f5a_Oncorhynchus_nerka

MAMVVNQWQENISADPGSQLQICSQEPGGTPGTPSGSTPGNDALSGDKIPNVD**CMVCGDKSSGKHYGQFTCEGC**KSFFKRSVRRNLSYT**CRGNRDCPIDQHHRNQCQYC**RLKKCLKVGMRREAVQRGRMSNSQSSPGQYLTNGSDPYNGQPYLSGFISLLLRAEPYPTSRYGSQCMQGNNLMGIENICELAARLLFSAVEWAKNIPFFPDLQLMDQVALLRMSWSELFVLNAAQCSMPLHVAPLLAAAGLHASPMSAERVVAFMDHIRVFQEQVEKLKVLQVDTAEYSCLKSIVLFTSDIPPYIPVPSPRIKERPSYHRHHLHPQSVAVCAQGARQREVVPVKDACGGVGVACEGSVTQTALAFSATDASHRQPLLSQPLMPHTDPKMADS

>XP_029481357.1_Nr2f5b_Oncorhynchus_nerka

MAMVVNQWQENISADPGSQLQICSQEPGGTPGTPSGSTPGNDALSGDKIPNVD**CMVCGDKSSGKHYGQFTCEGC**KSFFKRSVRRNLSYT**CRGNRDCPIDQHHRNQCQYC**RLKKCLKVGMRREAVQRGRMSNSQSSPGQYLTNGSDPYNGQPYLSGFISLLLRAEPYPTSRYGSQCMQGNNLMGIENICELAARLLFSAVEWAKNIPFFPDLQLMDQVALLRMSWSELFVLNAAQCSMPLHVAPLLAAAGLHASPMSAERVVAFMDHIRVFQEQVEKLKVLQVDTAEYSCLKSIVLFTSDAMGLSDVAHVESIQEKSQCALEEYVRNQYPSQPNRFGRLLLRLPSLRIVSSPVIEQLFFVRLVGKTPIETLLRDMLLSGSSYNWPYMPVQRDRPISLHYNENGP

>XP_021415215.2_Nr2f5a_Oncorhynchus_mykiss

MAMVVNQWQENISADPGSQLQICSQEPGGTPGTPSGSTPGNDALSGDKIPNVD**CMVCGDKSSGKHYGQFTCEGC**KSFFKRSVRRNLSYT**CRGNRDCPIDQHHRNQCQYC**RLKKCLKVGMRREAVQRGRMSNSQSSPGQYLTNGSDPYNGQPYLSGFISLLLRAEPYPTSRYGSQCMQGNNLMGIENICELAARLLFSAVEWAKNIPFFPDLQLMDQVALLRMSWSELFVLNAAQCSMPLHVAPLLAAAGLHASPMSAERVVAFMDHIRVFQEQVEKLKVLQVDTAEYSCLKSIVLFTSDAMGLSDVAHVESIQEKSQCALEEYVRNQYPSQPNRFGRLLLRLPSLRIVSSPVIEQLFFVRLVGKTPIETLLRDMLLSGSSYNWPYMPVQRDRPISLHYNENGP

>XP_036802179.1_Nr2f5b_Oncorhynchus_mykiss

MAMVVNQWQENISADPGSQLQICSQEPGGTPGTPSGSTPGNDALSGDKIPNVD**CMVCGDKSSGKHYGQFTCEGC**KSFFKRSVRRNLSYT**CRGNRDCPIDQHHRNQCQYC**RLKKCLKVGMRREAVQRGRMSNSQSSPGQYLTNGSDPYNGQPYLSGFISLLLRAEPYPTSRYGSQCMQGNNLMGIENICELAARLLFSAVEWAKNIPFFPDLQLMDQVALLRMSWSELFVLNAAQCSMPLHVAPLLAAAGLHASPMSAERVVAFMDHIRVFQEQVEKLKVLQVDTAEYSCLKSIVLFTSDIPPYIPVPSPRIKERPSYHCHHLHPQSVAVCAQGARQREVVPVKDACGGVGVACEGSVTQTALAFSATDASHRP
