# Supplementary material for: Origin and evolutionary landscape of Nr2f transcription factors across Metazoa
Source: PLoS One. 2021 Nov 22;16(11):e0254282. doi: 10.1371/journal.pone.0254282 (PMC8608329; doi:10.1371/journal.pone.0254282)
Supplement: S9 File — (DOCX) [file pone.0254282.s013.docx]

**S9 File. Selected Nr2f transcripts and translations used for analysis with the positions and phases of intron/exon boundaries indicated.**

phase 0

phase 1

phase 2

**>*T. adhaerens* Nr2f**
ATGTCAACAAGATCAAGTGAATACATCGATTCTCCAACCGCGGCTGCGAAAGATGAAACTAAATCTTTAAGCAAGGAACTTTGTTGTCTAATTTGCGGGGATAGAAGCAATGGTCGACATTACGGTGTTATCAGTTGTGAGGGCTGCAAAGGATTTTTTAAACGTAGTGTGAGACGTAACATGAAGTATGCGTGTACATGTTCAGCGAATGCCTGCAAAATCACAAAGGCAAACCGAAATCAGTGCCAGTTTTGTCGACTGCAGAAATGCTTCAAGGTGGGAATGAGAAAAGAAGCCGTTCAAAAAGAAAGGCACACGTCTACCATTCGCGCCGATCGTAATTCTGGCAAAACCGAGAAAGAAATGACACCAGATTCAGAAACAGCAATAAATAGCCTTATTAAGAACCTCGTCGCAGCTGAGACATTAGTACTTTCTAGTAGAAGTCTGCAACTGCAATCAGGTTTTATCGGATTTGAGGCTATCTGCCAATCTTCAATGCGAATTCTCTATTCTGTTGTGGAATGGACGGTTAAACTACCCTACTTTTCAGAGATGACTAGTTGTACCGATCAAATGACACTGCTTAGATCTTGCTGGTCTGAATTATTCATTTTGAATGCTGCCCAATGGTCTCCACCATTGAACATGTTTCCATACTCCACAACCAGTAACTTTTACCTAACTCACCCTCAAGAAGTCATGCATCATATATGCCTTTTTCAAGAAGCTATTGTGAAACTTAAGAAAAGATTTATTGACACAACGGAATTTTCTTGCTTAAAGGCATTGATATTATTTAACCCAGATGTTCGAGGATTAGTCAATCCAAATTATGTCGAATATATTCAAGAAAATATACAATGTGCTCTTAAACAGCATGTTAAAAGCCAATATCCAGATCAACCATCCCGCTTTGGTTATCTACTTTTAAGATTATTAATGTTACGCTCTATTAGCTCTAAAGTTATCGAAGAAATATTTTTTACGTCGGTGCTTTGTCGCCGTTCTATAGATATTTTCCTCTGCGAAGCTATGGAATCAGTGAAGAGAGCTTAG

atgtcaacaagatcaagtgaatacatcgattctccaaccgcggctgcgaaagatgaaact

M S T R S S E Y I D S P T A A A K D E T

aaatctttaagcaaggaactttgttgtctaatttgcggggatagaagcaatggtcgacat

K S L S K E L C C L I C G D R S N G R H

tacggtgttatcagttgtgagggctgcaaaggattttttaaacgtagtgtgagacgtaac

Y G V I S C E G C K G F F K R S V R R N

atgaagtatgcgtgtacatgttcagcgaatgcctgcaaaatcacaaaggcaaaccgaaat

M K Y A C T C S A N A C K I T K A N R N

cag**tgcc**agttttgtcgactgcagaaatgcttcaaggtgggaatgagaaaagaag**c**cgtt

Q **C** Q F C R L Q K C F K V G M R K E **A** V

caaaaagaaaggcacacgtctaccattcgcgccgatcgtaattctggcaaaaccgagaaa

Q K E R H T S T I R A D R N S G K T E K

gaaatgacaccagattcagaaacagcaataaatagccttattaagaacctcgtcgcagct

E M T P D S E T A I N S L I K N L V A A

gagacattagtactttctagtagaagtctgcaactgcaatcaggttttatcggatttgag

E T L V L S S R S L Q L Q S G F I G F E

gctatctgccaatcttcaatgcgaattctctattctgttgtggaatggacggttaaacta

A I C Q S S M R I L Y S V V E W T V K L

ccctacttttcagagatgactagttgtaccgatcaaatgacactgcttagatcttgctgg

P Y F S E M T S C T D Q M T L L R S C W

tctgaattattcattttgaatgctgcccaatggtctccaccattgaacatgtttccatac

S E L F I L N A A Q W S P P L N M F P Y

tccacaaccagtaacttttacctaactcaccctcaagaagtcatgcatcatatatgcctt

S T T S N F Y L T H P Q E V M H H I C L

tttcaagaagctattgtgaaacttaagaaaagatttattgacacaacggaattttcttgc

F Q E A I V K L K K R F I D T T E F S C

ttaaaggcattgatattatttaaccca**gat**gttcgaggattagtcaatccaaattatgtc

L K A L I L F N P **D** V R G L V N P N Y V

gaatatattcaagaaaatatacaatgtgctcttaaacagcatgttaaaagccaatatcca

E Y I Q E N I Q C A L K Q H V K S Q Y P

gatcaaccatcccgctttggttatctacttttaagattattaatgttacgctctattagc

D Q P S R F G Y L L L R L L M L R S I S

tctaaagttatcgaagaaatattttttacgtcggtgctttgtcgccgttctatagatatt

S K V I E E I F F T S V L C R R S I D I

ttcctctgcgaagctatggaatcagtgaagagagcttag

F L C E A M E S V K R A -

***>N. vectensis Nr2f1/2/5/6c***

ATGAATGGGAGTGTGTTCTGGTCTCCGGATGGAGGCGTTGACAACGACCCCGACTCGCCAGCCTCATCCTCATCTAAACCCCTTTATATTGACTGCGCGGTTTGCGGCGACAAATCTTCGGGGAAACATTATGGTGTGTACACATGCGAGGGCTGCAAAAGCTTCTTCAAGAGAAGTATAAGACGGAGTTTGAGCTACTCGTGTCGGGGCGTTCGTAATTGCCCAGTGGATATCCAGAACAGGAATCAGTGTCAGTACTGTCGGCTGAAGAAATGCCTCAAAGTTGGGATGAGAAAGGAAGCGGTACAGAAAGGGCGAATCCCATCCACCCATCCAGACGTAGGACCTTTATCTGTATCCATGGTTGAGATGAATGGTCACCAGTCCTTCTATTCCAGCTACATTACCCTCCTCTTGCGAGCAGATACCATTGCACGCTACCAACAATCACTCACACTGCCGTGTAATATAAATGGTTTAGAAAACACCCCCGAGCTTGCAGCAAGGCTTCTCGTGTCCGCGGTGGAATGGGCCAAGAACATTCCCTTCTACTCAGATCTCCCTCTGCCAGACCAGGCCGTGTTACTGCGCTCGTGTTGGAGTGAGCTTTTTACCCTGAACGCTGCCCAGCACTGCTCGCCATTTCACATTTCCCCGACGCTGACCAGCAACTCATCTGGGTTTGCAGGTAACGGAGGCGGTTATCTGAACACGCGTGTTATGTCCGCATTTGACTGCCAGAACAATAATATGAAGCTCTTCGAGGAGCAGGTTGAGAAGCTAAAGAACATGCACATAGACTCGGCCGAGTTTGCTTGTCTAAAGGCCATTGTCCTTTTTAATCCAGATAGCCAGGGCCTGTCTGAACCAGCACAGGTAGAAAACCTACAGGACCGCACCCAGAGTGCCCTTGAGGATTACATTCGAACCCAGTACCCCAATCAGACCACGCGGTTCGGCAAGCTCCTTCTCAGGCTCCCGGCTCTAAGACTCCTTCGCCCGGTGTCAGTTGAGAACCTGTTTTTCTCTCGACTCAGCATGGGGAATACTGTGGATAGCTTGCTTAACGACATGCTGTTGTCGGGTCTAGGGGGAGGGGTGGTACCCTGGTTACCAGGGCCCAGTCCCCCTCTGAATTGTACAACGTCAAATATGAATGTCATTACACAAATGTAA

atgaatgggagtgtgttctggtctccggatggaggcgtt**gac**aacgaccccgactcgcca

M N G S V F W S P D G G V **D** N D P D S P

gcctcatcctcatctaaacccctttatattgactgcgcggtttgcggcgacaaatcttcg

A S S S S K P L Y I D C A V C G D K S S

gggaaacattatggtgtgtacacatgcgagggctgcaaaagcttcttcaagagaagtata

G K H Y G V Y T C E G C K S F F K R S I

agacggagtttgagctactcgtgtcggggcgttcgtaattgcccagtggatatccagaac

R R S L S Y S C R G V R N C P V D I Q N

aggaatcagtgtcagtactgtcggctgaagaaatgcctcaaagttgggatgagaaaggaa

R N Q C Q Y C R L K K C L K V G M R K E

g**c**ggtacagaaagggcgaatcccatccacccatccagacgtaggacctttatctgtatcc

**A** V Q K G R I P S T H P D V G P L S V S

atggttgagatgaatggtcaccagtccttctattccagctacattaccctcctcttgcga

M V E M N G H Q S F Y S S Y I T L L L R

gcagataccattgcacgctaccaacaatcactcacactgccgtgtaatataaat**ggt**tta

A D T I A R Y Q Q S L T L P C N I N **G** L

gaaaacacccccgagcttgcagcaaggcttctcgtgtccgcggtggaatgggccaagaac

E N T P E L A A R L L V S A V E W A K N

attcccttctactcagatctccctctgccagaccaggccgtgttactgcgctcgtgttgg

I P F Y S D L P L P D Q A V L L R S C W

agtgagctttttaccctgaacgctgcccagcactgctcgccatttcacatttccccgacg

S E L F T L N A A Q H C S P F H I S P T

ctgaccagcaactcatctgggtttgcaggtaacggaggcggttatctgaacacgcgtgtt

L T S N S S G F A G N G G G Y L N T R V

atgtccgcatttgactgccagaacaataatatgaagctcttcgaggagcaggttgagaag

M S A F D C Q N N N M K L F E E Q V E K

ctaaagaacatgcacatagactcggccgagtttgcttgtctaaaggccattgtccttttt

L K N M H I D S A E F A C L K A I V L F

aatcca**gat**agccagggcctgtctgaaccagcacaggtagaaaacctacaggaccgcacc

N P **D** S Q G L S E P A Q V E N L Q D R T

cagagtgcccttgaggattacattcgaacccagtaccccaatcagaccacgcggttcggc

Q S A L E D Y I R T Q Y P N Q T T R F G

aagctccttctcaggctcccggctctaagactccttcgcccggtgtcagttgagaacctg

K L L L R L P A L R L L R P V S V E N L

tttttctctcgactcagcatggggaatactgtggatagcttgcttaacgacatgctgttg

F F S R L S M G N T V D S L L N D M L L

tcgggtctagggggaggggtggtaccctggttaccagggcccagtccccctctgaattgt

S G L G G G V V P W L P G P S P P L N C

acaacgtcaaatatgaatgtcattacacaaatgtaa

T T S N M N V I T Q M -

***>N. vectensis Nr2f1/2/5/6b***

ATGCCAAAGCACAAGACGGATGAGAGAGTCGCGTGCGCAGTTTGCGGAGATAAATCAACAGGGAAACACTATGGGGTCAGCACGTGTGAAGGATGTAAAAGCTTCTTTAAGAGAACAGTACGAAACAACACAAACTACACTTGCCGGGGTCAAAATACGTGCGCCATTGATAGAAATAGCAGAAGTCGTTGCCCCTCCTGCCGATTCCAGAAGTGTCTAAGCACTGGAATGAAAAAAGAAGCGGTCCAAACCACCAAGCTTCCTCCATTTCCCGCGCTCCAATTCCCGTTCTACGGTGACGTCAACACTATGTACGCCCAGACCATGTTTCCGCTGACCCTGTTCCAGTCCCCCTTCAACGCGCCGCTCACTTTCCCCATGGGGATGGTACCACTCAACCAAAACCGCACAGACGTCGCCTACGAACTCGCAGCCAACGTCCTCTTCGCAGTCGTCGACTGGGCTCGCAAATTGACCACGTTCAACAACCTGATGGACTCAGACCAGATAACGCTCCTAAAGATGGCCTGGACCGACCTGTTTCTTCTTGAAGCCTCTCGCAGTCCTCTGCAGCTCTATGTCCAGCAGATGTACGCGACAATCAATGCGCAGACGAAGCAGTTATCGATGGAGGTGATCGTGAAGAGGATGGAGTATGCGCGGTTATTTCAAGAACAGGCGGAACGGATAAGGAATCTTGGGATGGACATGACGGAGCATTTTCACCTTAAGTGCATTGTGCTATTCAGAGCAGATGGCTCTCTCATCAACCAGCCACGCCAAGTCGAGGTTCTACAAGACACTTCCCAGAGTTCTTTGGAGCAGTACATTCGTTCCCAGTACCCCAGTCAGCCCACGCGATTCGGCAAACTTCTCCTCATGTTGTCATCGTTGCGAAAGGTTGAGTCCACGGTCATCGAGCAGCTGTTTTTCGCAGATGTCTTGCGCGGCGCTTCCATGGGAGAAGTCTTGAAAAAAATGCTCACCACAGGGAACCAGTCACCTACAACGTTGGCTGCCGCTCTTGCAAATGGCAAAGGCAGCCCAATGTCATAG

atgccaaagcacaagacggatgagagagtcgcgtgcgcagtttgcggagataaatcaaca

M P K H K T D E R V A C A V C G D K S T

gggaaacactatggggtcagcacgtgtgaaggatgtaaaagcttctttaagagaacagta

G K H Y G V S T C E G C K S F F K R T V

cgaaacaacacaaactacacttgccggggtcaaaatacgtgcgccattgatagaaatagc

R N N T N Y T C R G Q N T C A I D R N S

agaagtcgttgcccctcctgccgattccagaagtgtctaagcactggaatgaaaaaagaa

R S R C P S C R F Q K C L S T G M K K E

g**c**ggtccaaaccaccaagcttcctccatttcccgcgctccaattcccgttctacggtgac

**A**  V Q T T K L P P F P A L Q F P F Y G D

gtcaacactatgtacgcccagaccatgtttccgctgaccctgttccagtcccccttcaac

V N T M Y A Q T M F P L T L F Q S P F N

gcgccgctcactttccccatggggatggtaccactcaaccaaaaccgcacagacgtcgcc

A P L T F P M G M V P L N Q N R T D V A

tacgaactcgcagccaacgtcctcttcgcagtcgtcgactgggctcgcaaattgaccacg

Y E L A A N V L F A V V D W A R K L T T

ttcaacaacctgatggactcagaccagataacgctcctaaagatggcctggaccgacctg

F N N L M D S D Q I T L L K M A W T D L

tttcttcttgaagcctctcgcagtcctctgcagctctatgtccagcagatgtacgcgaca

F L L E A S R S P L Q L Y V Q Q M Y A T

atcaatgcgcagacgaagcagttatcgatggaggtgatcgtgaagaggatggagtatgcg

I N A Q T K Q L S M E V I V K R M E Y A

cggttatttcaagaacaggcggaacggataaggaatcttgggatggacatgacggagcat

R L F Q E Q A E R I R N L G M D M T E H

tttcaccttaagtgcattgtgctattcagagcagatggctctctcatcaaccagccacgc

F H L K C I V L F R A **D** G S L I N Q P R

caagtcgaggttctacaagacacttcccagagttctttggagcagtacattcgttcccag

Q V E V L Q D T S Q S S L E Q Y I R S Q

taccccagtcagcccacgcgattcggcaaacttctcctcatgttgtcatcgttgcgaaag

Y P S Q P T R F G K L L L M L S S L R K

gttgagtccacggtcatcgagcagctgtttttcgcagatgtcttgcgcggcgcttccatg

V E S T V I E Q L F F A D V L R G A S M

ggagaagtcttgaaaaaaatgctcaccacagggaaccagtcacctacaacgttggctgcc

G E V L K K M L T T G N Q S P T T L A A

gctcttgcaaatggcaaaggcagcccaatgtcatag

A L A N G K G S P M S -

***>N. vectensis Nr2f1/2/5/6a***

ATGGAAGTAGCACCAGTTACCACATGGACTAGAGACAGCACAGAGACGCCAGAAGACTCGGAGAAAAATGTCCAAGTTGAGTGTGCGGTGTGCGGGGACAAGTCTTCCGGCAAGCACTACGGTGTCTTCACGTGCGAGGGCTGCAAGTCTTTCTTCAAGCGCAGCGTCCGGCGAAACCTTACTTATACGTGTAGAGCTTCCAGGGACTGCCCTATAGACCAGCACCACAGAAACCAGTGCCAGTACTGTCGTTTAAAGAAGTGTCTCAAAGTGGGCATGCGGCGAGAAGCGGTCCAGCGAGGTCGTATCCCAGCGGCCCAAACACCGACACAGAACGCAGCTCTCCCGGGAATCAACGGCGATGGAAGTACAAACGGACACTCGTATCTGTCGGGCTTCATCGCGCTCTTGCTCCGCGCAGAACCTTACCCGACGACGCGTTTCCAGCAGGGGCTTAACATGCCTTGTGGTATTATGGGCATCGAGAATATCTGCGAACTGGCCGCTCGGTTGTTGTTTAGCGCAGTGGAGTGGGCTCGTAATATCCCCTTCTTCCCTGACCTAGCAGTGACAGACCAAGTCGCTCTCCTTCGACTCGTGTGGAGTGAGCTGTTCGTACTGAACGCCGCACAGTGCCCGATGCCTCTCCAAGTAGCTCCTCTATTGGCCACCGCTGGGATTCACTCCAACCACATGTCGCCCGACCGAATGGTCTCGTTTATGGATAACATTCGCATCTTTCAGGAGCAAGTCGAAAAGCTGCGAAACCTGCACGTGGATGCTGCAGAGTTTGCGTGTCTGAAAGCAATCGTCCTGTTTACCTCAGACGCTTCCGGCCTCACTGATCCACAGTATATCGAAAGCCTTCAGGAGAAGACGCAATGCGCGCTTGAAGAGTACACGCGTAACCAGTACCCAAACCAACCGACCAGATTCGGCAAGTTACTACTCCGCCTGCCTTCCTTGAGAAGTATAAGTTCTTCCGTTGTGGAACAACTGTTCTTTGTTCGGTTGGTTGGGAAAACACCAATCGAGACCTTGCTAAGGGACATGCTTCTATCGGGAACTCCGACCACTTGGCCGTATCTGCCTTGCTCATAA

atggaagtagcaccagttaccacatggactagagacagcacagagacgccagaagactcg

M E V A P V T T W T R D S T E T P E D S

gagaaaaatgtccaagttgagtgtgcggtgtgcggggacaagtcttccggcaagcactac

E K N V Q V E C A V C G D K S S G K H Y

ggtgtcttcacgtgcgagggctgcaagtctttcttcaagcgcagcgtccggcgaaacctt

G V F T C E G C K S F F K R S V R R N L

acttatacgtgtagagcttccagggactgccctatagaccagcaccacagaaaccagtgc

T Y T C R A S R D C P I D Q H H R N Q C

cagtactgtcgtttaaagaagtgtctcaaagtgggcatgcggcgagaa**gcg**gtccagcga

Q Y C R L K K C L K V G M R R E  **A** V Q R

ggtcgtatcccagcggcccaaacaccgacacagaacgcagctctcccgggaatcaacggc

G R I P A A Q T P T Q N A A L P G I N G

gatggaagtacaaacggacactcgtatctgtcgggcttcatcgcgctcttgctccgcgca

D G S T N G H S Y L S G F I A L L L R A

gaaccttacccgacgacgcgtttccagcaggggcttaacatgccttgtggtattatgggc

E P Y P T T R F Q Q G L N M P C G I M G

atcgagaatatctgcgaactggccgctcggttgttgtttagcgcagtggagtgggctcgt

I E N I C E L A A R L L F S A V E W A R

aatatccccttcttccctgacctagcagtgacagaccaagtcgctctccttcgactcgtg

N I P F F P D L A V T D Q V A L L R L V

tggagtgagctgttcgtactgaacgccgcacagtgcccgatgcctctccaagtagctcct

W S E L F V L N A A Q C P M P L Q V A P

ctattggccaccgctgggattcactccaaccacatgtcgcccgaccgaatggtctcgttt

L L A T A G I H S N H M S P D R M V S F

atggataacattcgcatctttcaggagcaagtcgaaaagctgcgaaacctgcacgtggat

M D N I R I F Q E Q V E K L R N L H V D

gctgcagagtttgcgtgtctgaaagcaatcgtcctgtttacctcag**a**cgcttccggcctc

A A E F A C L K A I V L F T S **D** A S G L

actgatccacagtatatcgaaagccttcaggagaagacgcaatgcgcgcttgaagagtac

T D P Q Y I E S L Q E K T Q C A L E E Y

acgcgtaaccagtacccaaaccaaccgaccagattcggcaagttactactccgcctgcct

T R N Q Y P N Q P T R F G K L L L R L P

tccttgagaagtataagttcttccgttgtggaacaactgttctttgttcggttggttggg

S L R S I S S S V V E Q L F F V R L V G

aaaacaccaatcgagaccttgctaagggacatgcttctatcgggaactccgaccacttgg

K T P I E T L L R D M L L S G T P T T W

ccgtatctgccttgctcataa

P Y L P C S -

***>N. vectensis Nr2f1/2/5/6d***

ATGTCTTCGTGGTCGCAAATGCACGAAGTTTATTGGTACGACAACCCAGACAAGACCATCGAGTGTGCCGTGTGCTCTGCACCGTCTTCCGGGCGGCACTACGGGGTCTTCACGTGTGAAGGGTGCAAGTGTTTTTTTAACAGGACGGTGCGCTACAAACTTACGTATATATGTGAGGGCTCGGGGAGTTGCCGTGTTGATAAGCAGAATCGCACTCAGTGCCAAGCCTGTAGATTTAAGAAATGCGCCACTGTTGGAATGCGGAGAGAAGCCATCCGACGCGGCCGACCCACTAAGTACTCTTACATCTCAAGATCTTCCAAATCCTTTACACCACAGTACGACCTTATCTCCGTGCTTACACAACTCGAGCGATCCATACGGCCGCCAGTCCCGTACCCATCAAGCGGCCTACAGAGTAGCCCTGTTAGCATGTACCACCGCGTCTGTTCGATCCTCGTGTCCACCTTAGACTGGTCTCGCAGGGTACCTATGTTTGCCAACTTAGATGTGTGTGAGCAGTACTCGGTACTGCGTTCTCGTTGGTGTGAGATGTTGATCGTTAGCGCGGCGCAATACGAGGTACACATTGATGGCATACCGCTTGCTTATGAGGTGGAGATGAACCCTGGATTTTGCAATGAGAAGCAGATTCAGTTGAAGCGGAGCCTGAGGAATTTTCAAGAGAGTGTTTGGCGCCTACGCGGGCTTGAAGAGGCAGAGTACGCATGCCTCAAGACCATAATACTCTTTTCTCCAGACGCCTCGGAAGGACCGTTTGTACAGGAGTTCGAGTCCTTACAAGAACTAGTCCTTAGTGCGCTTGACCGATTTTGTCGCGCGCGCTTCCCCGAGGAGCCGTCGCGATACGGAAAAGTTCTCCTGAAACTCATGTCACTCAAGAGCGTCATAGCGGAAGATATCGAGACTCTTGTCTTCTCAAAGCTTTTTCCGCACTCATCTGTAAGTGGGATTATCAGAAACCACTTGGTTAGTGACGTTACTTCAGCACCCGAATCAATGTCGCCTGCCAAGACGTCTCCGGTCAACCAATAG

atgtcttcgtggtcgcaaatgcacgaagtttattggtacgacaacccagacaagaccatc

M S S W S Q M H E V Y W Y D N P D K T I

gagtgtgccgtgtgctctgcaccgtcttccgggcggcactacggggtcttcacgtgtgaa

E C A V C S A P S S G R H Y G V F T C E

gggtgcaagtgtttttttaacaggacggtgcgctacaaacttacgtatatatgtgagggc

G C K C F F N R T V R Y K L T Y I C E G

tcggggagttgccgtgttgataagcagaatcgcactcagtgccaagcctgtagatttaag

S G S C R V D K Q N R T Q C Q A C R F K

aaatgcgccactgttggaatgcggagagaa**gcc**atccgacgcggccgacccactaagtac

K C A T V G M R R E A I R R G R P T K Y

tcttacatctcaagatcttccaaatcctttacaccacagtacgaccttatctccgtgctt

S Y I S R S S K S F T P Q Y D L I S V L

acacaactcgagcgatccatacggccgccagtcccgtacccatcaagcggcctacagagt

T Q L E R S I R P P V P Y P S S G L Q S

agccctgttagcatgtaccaccgcgtctgttcgatcctcgtgtccaccttagactggtct

S P V S M Y H R V C S I L V S T L D W S

cgcagggtacctatgtttgccaacttagatgtgtgtgagcagtactcggtactgcgttct

R R V P M F A N L D V C E Q Y S V L R S

cgttggtgtgagatgttgatcgttagcgcggcgcaatacgaggtacacattgatggcata

R W C E M L I V S A A Q Y E V H I D G I

ccgcttgcttatgaggtggagatgaaccctggattttgcaatgagaagcagattcagttg

P L A Y E V E M N P G F C N E K Q I Q L

aagcggagcctgaggaattttcaagagagtgtttggcgcctacgcgggcttgaagaggca

K R S L R N F Q E S V W R L R G L E E A

gagtacgcatgcctcaagaccataatactcttttctcca**gac**gcctcggaaggaccgttt

E Y A C L K T I I L F S P **D** A S E G P F

gtacaggagttcgagtccttacaagaactagtccttagtgcgcttgaccgattttgtcgc

V Q E F E S L Q E L V L S A L D R F C R

gcgcgcttccccgaggagccgtcgcgatacggaaaagttctcctgaaactcatgtcactc

A R F P E E P S R Y G K V L L K L M S L

aagagcgtcatagcggaagatatcgagactcttgtcttctcaaagctttttccgcactca

K S V I A E D I E T L V F S K L F P H S

tctgtaagtgggattatcagaaaccacttggttagtgacgttacttcagcacccgaatca

S V S G I I R N H L V S D V T S A P E S

atgtcgcctgccaagacgtctccggtcaaccaatag

M S P A K T S P V N Q -

***>B. belcheri Nr2f1/2/5/6***

ATGGCGATGGCAGTCAGTACATGGCGAGAACCGGGTGAAGACCTCTCGAGCCCGGACAAGCAGCAGCAGCAGCCGGCACCCGTCCCGGGACCAGGGCCGCAGCAGCCGCCGCCGTCGCAGCCCCCAGGGCAGACGCAGCAGTCGACCCCGGGTCCGCAGCAGCCAGGCCCGCCGACCCCAAACCAGCAGACGCCGAACGGCGGGACGATGCCGCAACAGACGCCGACGGCCCCGACGCAACCGGCCTCGCAGCAGGGCCAACAACAGTCCACCCCAACCACGGGCAGCCAGAGTCAGCACATCGAGTGCGTCGTGTGCGGGGACAAGAGTTCCGGAAAGCACTATGGCCAGTTCACATGCGAGGGTTGCAAGAGTTTCTTCAAGAGAAGCGTCCGTCGGAACCTGACCTACTCCTGCCGGGGCAACCGCACCTGCCCCATCGACCAACACCACCGGAACCAGTGTCAGTACTGCCGGCTCAAGAAGTGTCTGAAGATGGGCATGAGACGAGAAGGTGTGGCGGTTCAACGGGGCCGGATTCCCCCGACCCAGCACCCAGCCGGCGGCCAGTACGCCCTGACCAACGGAGTAGACCCGATCAACGGCCACTCGTACTTGTCCGGGTACATCTCTCTCCTGCTACGGGCTGAACCCTACCCGACCTCGCGCTATGGCACACAGTGTATGCAGCCCAACAACATTATGGGCATTGACAACATCTGCGAGCTCGCGGCTCGCCTCCTCTTCAGTGCCGTGGAATGGGCCAGGAATATTCCCTACTTCCCCGAGTTACAAGTCACCGACCAAGTAGCGCTGTTGCGCCTGGTGTGGAGTGAGCTATTCGTGCTGAACGCCTCACAGTGCTCCATGCCCCTTCACGTCGCCCCATTGCTTGCCGCCGCTGGCCTGCATGCCAGCCCCATGTCAGCCGACCGAGTCGTGGCGTTCATGGATCACATACGCATATTCCAGGAGCAAGTAGAGAAACTGAAAGCATTGCACGTGGATTCTGCAGAGTACAGCTGTCTCAAGGCGATCGTGCTCTTCACCTCAGGTAAGTCGAGTGGATGA

atggcgatggcagtcagtacatggcgagaaccgggtgaagacctctcgagcccggacaag

M A M A V S T W R E P G E D L S S P D K

cagcagcagcagccggcacccgtcccgggaccagggccgcagcagccgccgccgtcgcag

Q Q Q Q P A P V P G P G P Q Q P P P S Q

cccccagggcagacgcagcagtcgaccccgggtccgcagcagccaggcccgccgacccca

P P G Q T Q Q S T P G P Q Q P G P P T P

aaccagcagacgccgaacggcgggacgatgccgcaacagacgccgacggccccgacgcaa

N Q Q T P N G G T M P Q Q T P T A P T Q

ccggcctcgcagcagggccaacaacagtccaccccaaccacgggcagccagagtcagcac

P A S Q Q G Q Q Q S T P T T G S Q S Q H

atcgagtgcgtcgtgtgcggggacaagagttccggaaagcactatggccagttcacatgc

I E C V V C G D K S S G K H Y G Q F T C

gagggttgcaagagtttcttcaagagaagcgtccgtcggaacctgacctactcctgccgg

E G C K S F F K R S V R R N L T Y S C R

ggcaaccgcacctgccccatcgaccaacaccaccggaaccagtgtcagtactgccggctc

G N R T C P I D Q H H R N Q C Q Y C R L

aagaagtgtctgaagatgggcatgagacgagaaggtgtg**gcg**gttcaacggggccggatt

K K C L K M G M R R E G V **A**  V Q R G R I

cccccgacccagcacccagccggcggccagtacgccctgaccaacggagtagacccgatc

P P T Q H P A G G Q Y A L T N G V D P I

aacggccactcgtacttgtccgggtacatctctctcctgctacgggctgaaccctacccg

N G H S Y L S G Y I S L L L R A E P Y P

acctcgcgctatggcacacagtgtatgcagcccaacaacattatgggcattgacaacatc

T S R Y G T Q C M Q P N N I M G I D N I

tgcgagctcgcggctcgcctcctcttcagtgccgtggaatgggccaggaatattccctac

C E L A A R L L F S A V E W A R N I P Y

ttccccgagttacaagtcaccgaccaagtagcgctgttgcgcctggtgtggagtgagcta

F P E L Q V T D Q V A L L R L V W S E L

ttcgtgctgaacgcctcacagtgctccatgccccttcacgtcgccccattgcttgccgcc

F V L N A S Q C S M P L H V A P L L A A

gctggcctgcatgccagccccatgtcagccgaccgagtcgtggcgttcatggatcacata

A G L H A S P M S A D R V V A F M D H I

cgcatattccaggagcaagtagagaaactgaaagcattgcacgtggattctgcagagtac

R I F Q E Q V E K L K A L H V D S A E Y

agctgtctcaaggcgatcgtgctcttcacctcaggtaagtcgagtggatga

S C L K A I V L F T S G K S S G -

***>L. gigantea Nr2f1/2/5/6***
ATGACTCCTACACAGCCCACCGTTACGTCTTGGAGAGATCATACTGACGATTTAGTGGGTACGACACCAACAGTGGTTCCTCCACCGTTACCCCCACCCAATCCCACTGTCACACCAACACAGACACAGATTCCAACAGCTACCACACCGACCCCACAGCAGAATGGTGGTTCACCCAACTCGGAAACAAATAACAATAACAAACAACATATAGAATGTGTCGTATGTGGTGATAAAAGTTCTGGAAAACATTACGGACAATATACGTGTGAAGGGTGTAAAAGTTTCTTTAAAAGATCTGTGCGAAGGAATTTGAATTACACATGTCGTGGAAACAAGAACTGTCCCATAGATCAACATCACAGAAATCAGTGCCAGTATTGTCGTCTGAGAAAATGTTTGAAGGCAGGGATGAGACGGGAAGCTGTTCAAAGAGGTCGAATCCCACCAAGCCAGCATCCATTTGCCGGACAGATGGGCTTCCCTAACGGTGATCCTCTGGGCGGACACGGTTATTTTTCCAGCTTTATTACAATGTTATTGAGGGCAGAACCATACCCTACATCAAGATATGGACAATGCATGCAGAATAATATCGTCGGAATTGACAGTATCTGCGAATTGGCCGCCAGATTATTATTTAGCGCTGTCGAATGGGCAAGAAATGTTCACTTCTTTCCGGAATTACACGTAAACGATCAAGTTGAATTACTCCGAATCAGTTGGAGCGAACTTTTTGTTTTAAATGCTGCCCAGAGTTCAATGCCTTGTCATCTGTCACCACTTTTAGCCGCGGCTGGACTCCATGCTTCACCAATGCCTGCAGATCATGTAGTAGCCTTCATGGAGAATATCCGAACCTTTCAAGACCATGTGGAGAAATTGAAAAACCTTCATATCGACACAGCTGAATACAGTTGTTTAAAGGCCATCGCACTATTTAGTTCAGATTCTCGAAGTTTATCTGATATTAACCAAATAGAGAGTTTACAAGAGAGATCTCAATGTGCTCTGGAAGAGTATGTTAGAAGTCAATATCCCAATCAACCTACACGATTTGGTAAACTGCTGTTGCGACTACCTTCATTACGAGCAATCAATTCACATGTTATAGAACAACTGTTTTTTGTCCGACTGGTTGGAAAAACCCATATAGAAACATTAATCCGAGACATATTATTGAGTGGTAATTCATTCTCGTGGCCATATATGCCAATACAATGA

atgactcctacacagcccaccgttacgtcttggagagatcatactgacgatttagtgggt

M T P T Q P T V T S W R D H T D D L V G

acgacaccaacagtggttcctccaccgttacccccacccaatcccactgtcacaccaaca

T T P T V V P P P L P P P N P T V T P T

cagacacagattccaacagctaccacaccgaccccacagcagaatggtggttcacccaac

Q T Q I P T A T T P T P Q Q N G G S P N

tcggaaacaaataacaataacaaacaacatatagaatgtgtcgtatgtggtgataaaagt

S E T N N N N K Q H I E C V V C G D K S

tctggaaaacattacggacaatatacgtgtgaagggtgtaaaagtttctttaaaagatct

S G K H Y G Q Y T C E G C K S F F K R S

gtgcgaaggaatttgaattacacatgtcgtggaaacaagaactgtcccatagatcaacat

V R R N L N Y T C R G N K N C P I D Q H

cacagaaatcagtgccagtattgtcgtctgagaaaatgtttgaaggcagggatgagacgg

H R N Q C Q Y C R L R K C L K A G M R R

gaagctgttcaaagaggtcgaatcccaccaagccagcatccatttgccggacagatgggc

E A V Q R G R I P P S Q H P F A G Q M G

ttccctaacggtgatcctctgggcggacacggttatttttccagctttattacaatgtta

F P N G D P L G G H G Y F S S F I T M L

ttgagggcagaaccataccctacatcaagatatggacaatgcatgcagaataatatcgtc

L R A E P Y P T S R Y G Q C M Q N N I V

ggaattgacagtatctgcgaattggccgccagattattatttagcgctgtcgaatgggca

G I D S I C E L A A R L L F S A V E W A

agaaatgttcacttctttccggaattacacgtaaacgatcaagttgaattactccgaatc

R N V H F F P E L H V N D Q V E L L R I

agttggagcgaactttttgttttaaatgctgcccagagttcaatgccttgtcatctgtca

S W S E L F V L N A A Q S S M P C H L S

ccacttttagccgcggctggactccatgcttcaccaatgcctgcagatcatgtagtagcc

P L L A A A G L H A S P M P A D H V V A

ttcatggagaatatccgaacctttcaagaccatgtggagaaattgaaaaaccttcatatc

F M E N I R T F Q D H V E K L K N L H I

gacacagctgaatacagttgtttaaaggccatcgcactatttagttcag**a**ttctcgaagt

D T A E Y S C L K A I A L F S S **D** S R S

ttatctgatattaaccaaatagagagtttacaagagagatctcaatgtgctctggaagag

L S D I N Q I E S L Q E R S Q C A L E E

tatgttagaagtcaatatcccaatcaacctacacgatttggtaaactgctgttgcgacta

Y V R S Q Y P N Q P T R F G K L L L R L

ccttcattacgagcaatcaattcacatgttatagaacaactgttttttgtccgactggtt

P S L R A I N S H V I E Q L F F V R L V

ggaaaaacccatatagaaacattaatccgagacatattattgagtggtaattcattctcg

G K T H I E T L I R D I L L S G N S F S

tggccatatatgccaatacaatga

W P Y M P I Q -

***>S. purpuratus Nr2f1/2/5/6***

ATGTCGATGGCAGTCAATTCATGGAGGAATGAGGACCACCTCACAGCTGACAAGCACCATGGACCGCCGCAGCAGACAGGTCTCGAACCTTCACCTCTTGATCCCTGCCCACCAGACACGGATCATTATGCGGACACCGGACCTCTTCGGTTGCCGCCACCTCCACCACAGACACAGCCCCCTCATTCAGCGGCTGGTCCTCCACCACCACAGGTACAACCTCCACAAGCTCCCATTGGACCCCCACCACCATCAGGGGCATTGCAGGCTGCCACGCCGCAGCAAACACCAGCCACACCGACACAGACAGGACCAGGGAGCCAGGGTGGTGGGGGAGCCGCACAGAACGGCCCGCAGTCGGGCTCGGACTCGGGCAGCAACGTCACTTCCAATAACAATAACAACTCACAGCATATCGAGTGTGTAGTATGTCATGATAAGAGTAGTGGAAAGCATTATGGTCAGTTTACTTGCGAAGGTTGCAAGAGTTTCTTCAAAAGGAGCGTCCGGCGTAACCTCACCTACTCGTGTCGTGCTAATCGGAATTGTCCTATTGATCAACACCACCGGAATCAATGCCAGTACTGCAGACTTAAAAAGTGCCTCAAGATGGGCATGAGAAGGGAAGCTGTTCAGAGGGGTCGTATGCCACCGACTCAACCGGGCCCTGGTCAATACCTGGATGGTCGCTTCGAAGGACACACATTCCTCTCGGGTTACATCTCGCTACTGCTTCGAGCTGAGCCCTACCCGACGTCGCGTTACGCCCAGTGCATGCAGACCAACTCAGTAATGGGCATCGACAACATCTGCGAGCTCGCAGCCCGGCTCCTCTTCAGCGCCGTCGAGTGGGCTAGGAACATCCCTTTCTTCCCCGACCTCCAAGTCACTGACCAGGTGGCCTTGCTCAGGATGTGCTGGAGCGAGCTCTTCGTCCTCAACGCGTCGCAGTGTTCGATGCCCCTCCACGTCGCCCCGCTCCTCGCTGCGTCGGGACTCCACGCCAGCCCGATGTCGGCCGACCGCGTGGTCGCTTTCATGGACCATATCCGTATTTTCCAGGAACAAGTTGAAAAACTGAAAGCGTTACACGTGGACTCGGCAGAGTATAGCTGTATCAAGGCAATTGTACTCTTCACGTCAGTGACAATGTCCTGCCTTAATCTCTACCTGTATTTTGTTCATCTCCTCATCTCCGGCCGGGAATGGTTGGCAATCGAAGTTGAATTAAATCTGATAAATGACCCCTATGCGCGAAATGTCAGCCGGATAATTCGCACAGGTAGCAAATAA

atgtcgatggcagtcaattcatggaggaatgaggaccacctcacagctgacaagcaccat

M S M A V N S W R N E D H L T A D K H H

ggaccgccgcagcagacaggtctcgaaccttcacctcttgatccctgcccaccagacacg

G P P Q Q T G L E P S P L D P C P P D T

gatcattatgcggacaccggacctcttcggttgccgccacctccaccacagacacagccc

D H Y A D T G P L R L P P P P P Q T Q P

cctcattcagcggctggtcctccaccaccacaggtacaacctccacaagctcccattgga

P H S A A G P P P P Q V Q P P Q A P I G

cccccaccaccatcaggggcattgcaggctgccacgccgcagcaaacaccagccacaccg

P P P P S G A L Q A A T P Q Q T P A T P

acacagacaggaccagggagccagggtggtgggggagccgcacagaacggcccgcagtcg

T Q T G P G S Q G G G G A A Q N G P Q S

ggctcggactcgggcagcaacgtcacttccaataacaataacaactcacagcatatcgag

G S D S G S N V T S N N N N N S Q H I E

tgtgtagtatgtcatgataagagtagtggaaagcattatggtcagtttacttgcgaaggt

C V V C H D K S S G K H Y G Q F T C E G

tgcaagagtttcttcaaaaggagcgtccggcgtaacctcacctactcgtgtcgtgctaat

C K S F F K R S V R R N L T Y S C R A N

cggaattgtcctattgatcaacaccaccggaatcaatgccagtactgcagacttaaaaag

R N C P I D Q H H R N Q C Q Y C R L K K

tgcctcaagatgggcatgagaagggaa**gct**gttcagaggggtcgtatgccaccgactcaa

C L K M G M R R E A V Q R G R M P P T Q

ccgggccctggtcaatacctggatggtcgcttcgaaggacacacattcctctcgggttac

P G P G Q Y L D G R F E G H T F L S G Y

atctcgctactgcttcgagctgagccctacccgacgtcgcgttacgcccagtgcatgcag

I S L L L R A E P Y P T S R Y A Q C M Q

accaactcagtaatgggcatcgacaacatctgcgagctcgcagcccggctcctcttcagc

T N S V M G I D N I C E L A A R L L F S

gccgtcgagtgggctaggaacatccctttcttccccgacctccaagtcactgaccaggtg

A V E W A R N I P F F P D L Q V T D Q V

gccttgctcaggatgtgctggagcgagctcttcgtcctcaacgcgtcgcagtgttcgatg

A L L R M C W S E L F V L N A S Q C S M

cccctccacgtcgccccgctcctcgctgcgtcgggactccacgccagcccgatgtcggcc

P L H V A P L L A A S G L H A S P M S A

gaccgcgtggtcgctttcatggaccatatccgtattttccaggaacaagttgaaaaactg

D R V V A F M D H I R I F Q E Q V E K L

aaagcgttacacgtggactcggcagagtatagctgtatcaaggcaattgtactcttcacg

K A L H V D S A E Y S C I K A I V L F T

tca**gtg**acaatgtcctgccttaatctctacctgtattttgttcatctcctcatctccggc

S **V** T M S C L N L Y L Y F V H L L I S G

cgggaatggttggcaatcgaagttgaattaaatctgataaatgacccctatgcgcgaaat

R E W L A I E V E L N L I N D P Y A R N

gtcagccggataattcgcacaggtagcaaataa

V S R I I R T G S K -

***>C. robusta Nr2f1/2/5/6***

ATGGCGATGGTTGTGACATCATGGAGGGAATCAAATCATCATGATGATGTCACAGTGCTACAGCCAGCCAATCACGAGGCAGTATCGACCCCGCTACCCCCCCACCCCCAACCGCCCCCTGATCTAACCCCCCAAACCCCAACAACACCGACTAATGACGTCACAATACCTGATTGTTCAACGTCACGACCGACCGGAAATACGTCATCAACCAGCGAGAAGCCGCAGATTGAATGTGTGGTGTGTGGGGACAAAAGCAGTGGCAAACATTATGGCCAGTACACTTGCGAAGGGTGTAAAAGCTTCTTCAAACGAAGCGTACGAAGGAACCTTTCGTATACATGCAGAGGAAATAGAAATTGTCCAATAGATCAACATCATAGAAATCAATGCCAATATTGCCGACTGAATAAGTGTGTGAAGATCGGGATGCGAAGGGAAGCTGTACAAAGGGGACGCATGCCGCCCAGTCAACCCCATACTACAGGCCAATATGCGATAACCAACGGTGTTGAAAGCAACTTCGGTCCAGGGTATATGTCAGGCTATATATCCATGTTGTTAAGAGCCGAGCCTTACCCAACGTCGAGATTTGCTTTGCAATGTCCCGTCCCAAACCAAATTATGGGCATCGACAATATTTGTGAACTAGCCGCTAGATTGTTGTTTTCCGCCGTGGAATGGGCGCGAAATATCCCGTTCTTCCCAGAGTTGCAGGTCACCGATCAAGTAGCGATGTTAAAATGGGTCTGGAGCGAACTATTTGTGTTGAATGCAGCACAAAGTCACATGCCTCTACACGTGGCACCTTTACTAGCAGCGGCTGGTTTGCACACTTCTATGTCAGCTGACCGGGTGATGACGTTCATGGATCATATTCGCATCTTCCAAGAACAGGTTGAACGATTAAAGTCGCTTCATGTTGATTCGGCCGAATATTCTTGTCTGAAAGCAATTGTGCTTTTTACTGCAGATTCCCATGGATTGAGTGATATGACACACATAGAGAGCGTGCAAGAAAAGTCGCAATGCGCTTTAGAAGAATATGTTCGTCACCAATATCCAAACCAACCATCAAGGTTTGGAAAACTATTACTTCGGTTGCCATCGTTACGAACAGTTAGCGCGAGCGCGATTGAACAGTTATTCTTTGTTCGCCTTGTGGGGAAAACCCCCATTGAGACACTAATTCGAGATATGTTGCTGTCGGGGAGCTCATACGGTTGGCCGTATCACATGACATTGCAATGA

atggcgatggttgtgacatcatggagggaatcaaatcatcatgatgatgtcacagtgcta

M A M V V T S W R E S N H H D D V T V L

cagccagccaatcacgaggcagtatcgaccccgctacccccccacccccaaccgccccct

Q P A N H E A V S T P L P P H P Q P P P

gatctaaccccccaaaccccaacaacaccgactaatgacgtcacaatacctgattgttca

D L T P Q T P T T P T N D V T I P D C S

acgtcacgaccgaccggaaatacgtcatcaaccagcgagaagccgcagattgaatgtgtg

T S R P T G N T S S T S E K P Q I E C V

**gtg**tgtggggacaaaagcagtggcaaacattatggccagtacacttgcgaagggtgtaaa

**V** C G D K S S G K H Y G Q Y T C E G C K

agcttcttcaaacgaagcgtacgaaggaacctttcgtatacatgcagaggaaatagaaat

S F F K R S V R R N L S Y T C R G N R N

tgtccaatagatcaacatcatagaaatcaatgccaatattgccgactgaataagtgtgtg

C P I D Q H H R N Q C Q Y C R L N K C V

aagatcgggatgcgaagggaa**gct**gtacaaaggggacgcatgccgcccagtcaaccccat

K I G M R R E **A**  V Q R G R M P P S Q P H

actacaggccaatatgcgataaccaacggtgttgaaagcaacttcggtccagggtatatg

T T G Q Y A I T N G V E S N F G P G Y M

tcaggctatatatccatgttgttaagagccgagccttacccaacgtcgagatttgctttg

S G Y I S M L L R A E P Y P T S R F A L

caatgtcccgtcccaaaccaaattatgggcatcgacaatatttgtgaactagccgctaga

Q C P V P N Q I M G I D N I C E L A A R

ttgttgttttccgccgtggaatgggcgcgaaatatcccgttcttcccagagttgcaggtc

L L F S A V E W A R N I P F F P E L Q V

accgatcaagtagcgatgttaaaatgggtctggagcgaactatttgtgttgaatgcagca

T D Q V A M L K W V W S E L F V L N A A

caaagtcacatgcctctacacgtggcacctttactagcagcggctggtttgcacacttct

Q S H M P L H V A P L L A A A G L H T S

atgtcagctgaccgggtgatgacgttcatggatcatattcgcatcttccaagaacaggtt

M S A D R V M T F M D H I R I F Q E Q V

gaacgattaaagtcgcttcatgttgattcggccgaatattcttgtctgaaagcaattgtg

E R L K S L H V D S A E Y S C L K A I V

ctttttactgca**gat**tcccatggattgagtgatatgacacacatagagagcgtgcaagaa

L F T A **D** S H G L S D M T H I E S V Q E

aagtcgcaatgcgctttagaagaatatgttcgtcaccaatatccaaaccaaccatcaagg

K S Q C A L E E Y V R H Q Y P N Q P S R

tttggaaaactattacttcggttgccatcgttacgaacagttagcgcgagcgcgattgaa

F G K L L L R L P S L R T V S A S A I E

cagttattctttgttcgccttgtggggaaaacccccattgagacactaattcgagatatg

Q L F F V R L V G K T P I E T L I R D M

ttgctgtcggggagctcatacggttggccgtatcacatgacattgcaatga

L L S G S S Y G W P Y H M T L Q -

***>E. burgeri Nr2f1/2A***

ATGGCAATGGTAGTCGGGCCATGGCGAGATCCACAGGACGATCTGACGAGCGGTCAGAACGGACCGAGCCAGGCGGTACAGCCGCCCTTGGCTCCGGGAGGACCGCAGACGCCACAGACGCCAAGCCAGTCGGGTCCGCCGACAACGCCAGGGCAGGGCAGCCAACAGGGCGACAAGCAACAACAACCGAACGTGGAGTGCGTGGTGTGCGGGGACAAGTCGAGCGGGAAGCACTACGGACAGTTTACCTGTGAGGGATGTAAAAGTTTCTTCAAGCGGAGCGTCCGCAGGAACCTCACGTACACGTGCCGCGCAAATCGGAACTGCCCCATCGACCAGCATCACCGCAACCAGTGCCAGTACTGTCGCCTCAAGAAATGCCTTAAGGTCGGGATGAGGCGTGAAGATCGCTCCGTCTTTCTCTTCGCAGCCGTTCAGCGAGGCCGCTTACCGCCCACACAGCACCCGAACCCAGCCTTGCAGTACGCGCTGGTTAATGGTGACCCCTTGAACGGTCACTCGTACCTCTCGGGCTACATCTCGCTGCTGCTCCGTGCTGAGCCCTACCCGACGTCCCGCTATGGAAGCCAGTGCATGCAGCCCAACAACATCATGGGCATCGAAAACATCTGCGAACTGGCCGCGCGTCTCCTGTTTAGCGCCGTGGAGTGGGCACGGAACATCCCCTTCTTCCCGGACCTTCAGATCACCGACCAGGTGGCGCTTCTCCGCCTCGTTTGGAGCGAGCTGTTCGTGCTGAATGCGGCGCAGTGCGCCATGCCGCTGCACGTTGCCCCCTTGCTGGCAGCCGCCGGCTTGCACGCCTCGCCGATGTCGGCGGACCGTGTGGTTGCCTTCATGGACCACATCCGCATCTTCCAGGAGCAGGTCGAGAAGCTCAAAGCGCTGCATGTGGACTCTGCTGAATACAGCTGTCTTAAAGCCATCGTGCTCTTCTCGACAGATGCCTGCGGCTTGTCGGACGCAGCACACATAGAAAGCCTGCAAGAGAAATCTCAGTGCGCGCTCGAAGAGTACGTGAGAAGTCAGTACCCGAACCAGCCAACGCGCTTCGGCAAGCTCTTGCTGCGACTACCGTCGCTACGCACTGTGTCCTCCTCTGTTATTGAGCAGCTGTTCTTCGTAAGGTTGGTAGGTAAAACCCCCATAGAAACCCTCATCCGTGACATGCTTCTGTCAGGGAGCAGTTTCAACTGGCCATACATGCCTATACAATAG

atggcaatggtagtcgggccatggcgagatccacaggacgatctgacgagcggtcagaac
 M  A  M  V  V  G  P  W  R  D  P  Q  D  D  L  T  S  G  Q  N 
ggaccgagccaggcggtacagccgcccttggctccgggaggaccgcagacgccacagacg
 G  P  S  Q  A  V  Q  P  P  L  A  P  G  G  P  Q  T  P  Q  T 
ccaagccagtcgggtccgccgacaacgccagggcagggcagccaacagggcgacaagcaa
 P  S  Q  S  G  P  P  T  T  P  G  Q  G  S  Q  Q  G  D  K  Q 
caacaaccgaacgtggagtgcgtggtgtgcggggacaagtcgagcgggaagcactacgga
 Q  Q  P  N  V  E  C  V  V  C  G  D  K  S  S  G  K  H  Y  G 
cagtttacctgtgagggatgtaaaagtttcttcaagcggagcgtccgcaggaacctcacg
 Q  F  T  C  E  G  C  K  S  F  F  K  R  S  V  R  R  N  L  T 
tacacgtgccgcgcaaatcggaactgccccatcgaccagcatcaccgcaaccagtgccag
 Y  T  C  R  A  N  R  N  C  P  I  D  Q  H  H  R  N  Q  C  Q 
tactgtcgcctcaagaaatgccttaaggtcgggatgaggcgtgaa**gat**cgctccgtcttt
 Y  C  R  L  K  K  C  L  K  V  G  M  R  R  E  **D**  R  S  V  F 
ctcttcgcagccgttcagcgaggccgcttaccgcccacacagcacccgaacccagccttg
 L  F  A  A  V  Q  R  G  R  L  P  P  T  Q  H  P  N  P  A  L 
cagtacgcgctggttaatggtgaccccttgaacggtcactcgtacctctcgggctacatc
 Q  Y  A  L  V  N  G  D  P  L  N  G  H  S  Y  L  S  G  Y  I 
tcgctgctgctccgtgctgagccctacccgacgtcccgctatggaagccagtgcatgcag
 S  L  L  L  R  A  E  P  Y  P  T  S  R  Y  G  S  Q  C  M  Q 
cccaacaacatcatgggcatcgaaaacatctgcgaactggccgcgcgtctcctgtttagc
 P  N  N  I  M  G  I  E  N  I  C  E  L  A  A  R  L  L  F  S 
gccgtggagtgggcacggaacatccccttcttcccggaccttcagatcaccgaccaggtg
 A  V  E  W  A  R  N  I  P  F  F  P  D  L  Q  I  T  D  Q  V 
gcgcttctccgcctcgtttggagcgagctgttcgtgctgaatgcggcgcagtgcgccatg
 A  L  L  R  L  V  W  S  E  L  F  V  L  N  A  A  Q  C  A  M 
ccgctgcacgttgcccccttgctggcagccgccggcttgcacgcctcgccgatgtcggcg
 P  L  H  V  A  P  L  L  A  A  A  G  L  H  A  S  P  M  S  A 
gaccgtgtggttgccttcatggaccacatccgcatcttccaggagcaggtcgagaagctc
 D  R  V  V  A  F  M  D  H  I  R  I  F  Q  E  Q  V  E  K  L 
aaagcgctgcatgtggactctgctgaatacagctgtcttaaagccatcgtgctcttctcg
 K  A  L  H  V  D  S  A  E  Y  S  C  L  K  A  I  V  L  F  S 
aca**gat**gcctgcggcttgtcggacgcagcacacatagaaagcctgcaagagaaatctcag
 T  **D**  A  C  G  L  S  D  A  A  H  I  E  S  L  Q  E  K  S  Q 
tgcgcgctcgaagagtacgtgagaagtcagtacccgaaccagccaacgcgcttcggcaag
 C  A  L  E  E  Y  V  R  S  Q  Y  P  N  Q  P  T  R  F  G  K 
ctcttgctgcgactaccgtcgctacgcactgtgtcctcctctgttattgagcagctgttc
 L  L  L  R  L  P  S  L  R  T  V  S  S  S  V  I  E  Q  L  F 
ttcgtaaggttggtaggtaaaacccccatagaaaccctcatccgtgacatgcttctgtca
 F  V  R  L  V  G  K  T  P  I  E  T  L  I  R  D  M  L  L  S 
gggagcagtttcaactggccatacatgcctatacaatag
 G  S  S  F  N  W  P  Y  M  P  I  Q  -

***>E. burgeri Nr2f1/2B***

ATGGTAGTCGGGCCGTGGCGCGACCCGCAGGGCGAGCTCGGTGGCGGGCCCAACGTTTCAGCAGCCGCCGGCCAGGGGCCATTGGCTCCAGGTGGACCGCAAACGCCACAGACTCCAAGCCAGTCGGGCCCACCGACAACGCCTGGCCAAGGAAGCCAACAGGGTGACAAACAACAAGCTAATGTGGAGTGCGTGGTGTGTGGGGACAAGTCAAGTGGGAAGCACTACGGACAGTTCACCTGTGAGGGATGTAAAAGTTTCTTCAAGCGGAGCGTCCGCAGGAACCTCACATACACGTGCCGTGCCAATCGGAACTGCCCTATCGACCAGCACCATCGCAACCAGTGCCAGTACTGTCGCCTCAAGAAATGCCTTAAGGTCGGGATGAGGCGTGAAGCAGTACAAAGAGGCCGAATGCCGCCAACACAACATCCGAATACGGCTCAGTACGCGCTCGTCAATGGGGACCCGTTGAACGGTCACTCATATCTCTCGGGCTACATATCACTGCTGCTCCGCGCTGAGCCCTATCCAACATCACGCTATGGCAGTCAGTGCATGCAGCCCAACAACTTGATGGGCATCGAGAACATCTGCGAGCTCGCGGCCCGTCTGCTCTTCAGCGCCGTCGAGTGGGCGAGGAATATTCCGTTCTTCCCGGACCTGCAGATCACCGACCAGGTGGCGCTTCTGCGCCTGGTTTGGAGTGAGCTGTTTGTGCTGAATGCGGCGCAGTGTGCCATGCCACTGCATGTGGCGCCTTTGCTGGCAGCCGCGGGCTTGCACGCCTCGCCCATGTCTGCAGACCGTGTGGTTGCCTTCATGGACCACATTCGCATCTTCCAAGAGCAGGTGGAAAAGCTCAAGGTTTTGCACGTGGACTCTGCTGAGTACAGCTGCTTAAAAGCCATTGTGCTCTTCACCACAGATGCCTGCGGCTTATCGGACGCTGCGCACATAGACAGCCTGCAGGAGAAATCGCAGTGTGCACTGGAGGAATACGTGCGGAGTCAATACCCGAACCAGCCCACTCGCTTTGGAAAGCTCTTGCTTCGCTTACCGTCACTACGCACCGTGTCTTCGTCAGTTATCGAACAGCTCTTCTTCGTCCGCTTGGTTGGTAAAACTCCCATTGAAACCCTCATCAGAGACATGCTTCTTTCGGGGAGCAGTTTCAACTGGCCGTACATGTCCATTCAGTAG

atggcaatggtagtcgggccgtggcgcgacccgcagggcgagctcggtggcgggcccaac
 M  A  M  V  V  G  P  W  R  D  P  Q  G  E  L  G  G  G  P  N 
gtttcagcagccgccggccaggggccattggctccaggtggaccgcaaacgccacagact
 V  S  A  A  A  G  Q  G  P  L  A  P  G  G  P  Q  T  P  Q  T 
ccaagccagtcgggcccaccgacaacgcctggccaaggaagccaacagggtgacaaacaa
 P  S  Q  S  G  P  P  T  T  P  G  Q  G  S  Q  Q  G  D  K  Q 
caagctaatgtggagtgcgtggtgtgtggggacaagtcaagtgggaagcactacggacag
 Q  A  N  V  E  C  V  V  C  G  D  K  S  S  G  K  H  Y  G  Q 
ttcacctgtgagggatgtaaaagtttcttcaagcggagcgtccgcaggaacctcacatac
 F  T  C  E  G  C  K  S  F  F  K  R  S  V  R  R  N  L  T  Y 
acgtgccgtgccaatcggaactgccctatcgaccagcaccatcgcaaccagtgccagtac
 T  C  R  A  N  R  N  C  P  I  D  Q  H  H  R  N  Q  C  Q  Y 
tgtcgcctcaagaaatgccttaaggtcgggatgaggcgtgaa**gca**gtacaaagaggccga
 C  R  L  K  K  C  L  K  V  G  M  R  R  E  **A**  V  Q  R  G  R 
atgccgccaacacaacatccgaatacggctcagtacgcgctcgtcaatggggacccgttg
 M  P  P  T  Q  H  P  N  T  A  Q  Y  A  L  V  N  G  D  P  L 
aacggtcactcatatctctcgggctacatatcactgctgctccgcgctgagccctatcca
 N  G  H  S  Y  L  S  G  Y  I  S  L  L  L  R  A  E  P  Y  P 
acatcacgctatggcagtcagtgcatgcagcccaacaacttgatgggcatcgagaacatc
 T  S  R  Y  G  S  Q  C  M  Q  P  N  N  L  M  G  I  E  N  I 
tgcgagctcgcggcccgtctgctcttcagcgccgtcgagtgggcgaggaatattccgttc
 C  E  L  A  A  R  L  L  F  S  A  V  E  W  A  R  N  I  P  F 
ttcccggacctgcagatcaccgaccaggtggcgcttctgcgcctggtttggagtgagctg
 F  P  D  L  Q  I  T  D  Q  V  A  L  L  R  L  V  W  S  E  L 
tttgtgctgaatgcggcgcagtgtgccatgccactgcatgtggcgcctttgctggcagcc
 F  V  L  N  A  A  Q  C  A  M  P  L  H  V  A  P  L  L  A  A 
gcgggcttgcacgcctcgcccatgtctgcagaccgtgtggttgccttcatggaccacatt
 A  G  L  H  A  S  P  M  S  A  D  R  V  V  A  F  M  D  H  I 
cgcatcttccaagagcaggtggaaaagctcaaggttttgcacgtggactctgctgagtac
 R  I  F  Q  E  Q  V  E  K  L  K  V  L  H  V  D  S  A  E  Y 
agctgcttaaaagccattgtgctcttcaccaca**gat**gcctgcggcttatcggacgctgcg
 S  C  L  K  A  I  V  L  F  T  T  **D**  A  C  G  L  S  D  A  A 
cacatagacagcctgcaggagaaatcgcagtgtgcactggaggaatacgtgcggagtcaa
 H  I  D  S  L  Q  E  K  S  Q  C  A  L  E  E  Y  V  R  S  Q 
tacccgaaccagcccactcgctttggaaagctcttgcttcgcttaccgtcactacgcacc
 Y  P  N  Q  P  T  R  F  G  K  L  L  L  R  L  P  S  L  R  T 
gtgtcttcgtcagttatcgaacagctcttcttcgtccgcttggttggtaaaactcccatt
 V  S  S  S  V  I  E  Q  L  F  F  V  R  L  V  G  K  T  P  I 
gaaaccctcatcagagacatgcttctttcggggagcagtttcaactggccgtacatgtcc
 E  T  L  I  R  D  M  L  L  S  G  S  S  F  N  W  P  Y  M  S 
attcagtag
 I  Q  -

***>E. burgeri Nr2f1/2C***

ATGGCAATGGTGGTGAGCGCGACATGGAGAGACCCACTCGACAATAGTCCAGCGCCTGGAGCAGCGGCCCCTATTGCCCAATCCGTGCCCGGTGCGGCAAGCGTCGGTGCCAGCCAAGGGAGCTCGGGAGCGCTGTCGGCACCATCGTCGGCTTCTTCATCCTCATCGGCGTCGTCGACCGCTTCGTCAGCACCGGGCGACAAAACGCCACTACAGCACATCGAATGTGTCGTGTGCGGCGACAAGTCGAGCGGAAAACACTACGGACAGTTTACGTGCGAGGGCTGCAAGAGCTTCTTCAAGCGCTCAGTGCGTCGGAACCTGAGCTACACGTGTCGGGCCAGCCGCAACTGTCCCATCGACCAGCACCACCGGAACCAGTGTCAGTACTGCCGTCTCAAGAAGTGTCTCAAAGTGGGGATGCGGAGAGAAGCTGTGCAGAGAGGCAGACTGCCCCACATGCAGCACCCTGTACCTGGTCAGTACGCACTTGCCAATGGCGACCCCCTCAATGGCCACTCGTACCTCTCGGGCTACATATCACTACTGCTGCGGGCAGAACCATACCCGACATCCCGCTACGGGAGTCAGTGCATGCAGCCCAACAACATCATGGGCATAGAGAACATCTGCGAGCTTGCAGCACGTCTTCTCTTCAGCGCCGTGGAGTGGGCGAGGAACATTCCATTCTTCCCGGAGCTCCAGGTTGGAGATCAGGTGGCCTTGCTACGGCTGGCTTGGAGTGAACTCTTCGTCCTGAATGCAGCGCAATGTGCCATGCCGCTGCATGTGGCACCACTTTTGGCTGCCGCAGGACTCCATGCGTCGCCCATGTCAGCTGACCGCGTGGTCGCTTTTATGGACCACATCCGCATCTTCCAAGAACAAGTGGAAAAGCTGAAATCACTTCACGTGGACTCAGCAGAATATAGCTGTCTTAAAGCCATCGTCCTGTTCACATCAGATGCCTGTGGTCTTTCAGACACGGCACATGTTGAGGGACTGCAGGAGAAGTCTCAATGTGCTCTCGAAGAATATGTTCGGGCACAATATCCAGGTCAGCCAAGCCGCTTTGGGCGCTTACTCCTCCGGCTTCCTTCCCTGCGATCCGTCTCATCGTCGGTCATTGAACAATTATTCTTCGTTCGGCTTGTCGGTAAGACGCCGATTGAGACTCTGATCAGAGATATGCTTTTGTCTGGCAGCTCTTTCAGCTGGCCTTACATGTCGATACAGTGA

atggcaatggtggtgagcgcgacatggagagacccactcgacaatagtccagcgcctgga

M A M V V S A T W R D P L D N S P A P G

gcagcggcccctattgcccaatccgtgcccggtgcggcaagcgtcggtgccagccaaggg

A A A P I A Q S V P G A A S V G A S Q G

agctcgggagcgctgtcggcaccatcgtcggcttcttcatcctcatcggcgtcgtcgacc

S S G A L S A P S S A S S S S S A S S T

gcttcgtcagcaccgggcgacaaaacgccactacagcacatcgaatgtgtcgtgtgcggc

A S S A P G D K T P L Q H I E C V V C G

gacaagtcgagcggaaaacactacggacagtttacgtgcgagggctgcaagagcttcttc

D K S S G K H Y G Q F T C E G C K S F F

aagcgctcagtgcgtcggaacctgagctacacgtgtcgggccagccgcaactgtcccatc

K R S V R R N L S Y T C R A S R N C P I

gaccagcaccaccggaaccagtgtcagtactgccgtctcaagaagtgtctcaaagtgggg

D Q H H R N Q C Q Y C R L K K C L K V G

atgcggagagaa**gct**gtgcagagaggcagactgccccacatgcagcaccctgtacctggt

M R R E **A** V Q R G R L P H M Q H P V P G

cagtacgcacttgccaatggcgaccccctcaatggccactcgtacctctcgggctacata

Q Y A L A N G D P L N G H S Y L S G Y I

tcactactgctgcgggcagaaccatacccgacatcccgctacgggagtcagtgcatgcag

S L L L R A E P Y P T S R Y G S Q C M Q

cccaacaacatcatgggcatagagaacatctgcgagcttgcagcacgtcttctcttcagc

P N N I M G I E N I C E L A A R L L F S

gccgtggagtgggcgaggaacattccattcttcccggagctccaggttggagatcaggtg

A V E W A R N I P F F P E L Q V G D Q V

gccttgctacggctggcttggagtgaactcttcgtcctgaatgcagcgcaatgtgccatg

A L L R L A W S E L F V L N A A Q C A M

ccgctgcatgtggcaccacttttggctgccgcaggactccatgcgtcgcccatgtcagct

P L H V A P L L A A A G L H A S P M S A

gaccgcgtggtcgcttttatggaccacatccgcatcttccaagaacaagtggaaaagctg

D R V V A F M D H I R I F Q E Q V E K L

aaatcacttcacgtggactcagcagaatatagctgtcttaaagccatcgtcctgttcaca

K S L H V D S A E Y S C L K A I V L F T

tca**gat**gcctgtggtctttcagacacggcacatgttgagggactgcaggagaagtctcaa

S **D** A C G L S D T A H V E G L Q E K S Q

tgtgctctcgaagaatatgttcgggcacaatatccaggtcagccaagccgctttgggcgc

C A L E E Y V R A Q Y P G Q P S R F G R

ttactcctccggcttccttccctgcgatccgtctcatcgtcggtcattgaacaattattc

L L L R L P S L R S V S S S V I E Q L F

ttcgttcggcttgtcggtaagacgccgattgagactctgatcagagatatgcttttgtct

F V R L V G K T P I E T L I R D M L L S

ggcagctctttcagctggccttacatgtcgatacagtga

G S S F S W P Y M S I Q -

***>P. marinus Nr2f1/2A***

ATGGCAATGGTAGTCGGGCCGTGGCGAGACCCACAGGGCGACCTGACCAGCCCCCCGAACGTGCCCGGCCAGGCGGGCCAAGCCCCGCTGGCTCCCGCGGGGCCGCAGACGCCGCAGACGCCGAGTCAGTCGGGCGGGCCGCCCACGACTCCGGGCCAGGGCAGCACGCAGGGTGACAAGCAGCAGCAGCAGCAGCAGCCGAACGTGGAGTGCGTCGTGTGCGGAGACAAATCGAGCGGCAAGCACTACGGACAGTTCACGTGCGAGGGATGTAAAAGTTTCTTCAAGCGCAGCGTTCGCCGGAACCTGACCTACACGTGCCGCGCCAATCGGAATTGTCCCATCGACCAGCACCACCGCAATCAGTGTCAGTACTGCCGCCTCAAGAAATGCCTCAAAGTCGGGATGCGACGCGAAGATCGTGCGGGTCTCGGGTCTGCAATGCAACGGTCTTCTCTGTCTCCCATTGCAGCCGTGCAGAGGGGAAGACTGCCTCCCTCGCAGCACCCGAATCCGGCACAGTACGCCCTGGTTAACGGCGACCCTCTGAACGGCCACTCGTACCTCTCGGGCTACATCTCGCTGCTGCTGCGCGCCGAACCTTACCCCACGTCGCGCTACGGCAGCCAGTGCATGCAGCCCAACAACATCATGGGCATTGAGAACATCTGCGAGCTGGCTGCGCGTCTCCTCTTCAGCGCCGTGGAGTGGGCGAGGAACATCCCGTTCTTCCCCGACCTGCAGATCACGGACCAGGTGGCGCTGCTACGACTCGTGTGGAGCGAGCTGTTCGTGCTGAACGCGGCTCAGTGCGCCATGCCGCTCCACGTGGCGCCCCTGCTCGCCGCCGCGGGCCTCCACGCGTCGCCCATGTCCGCCGATCGCGTGGTGGCCTTCATGGACCACATCCGCATCTTCCAGGAGCAGGTGGAGAAGCTCAAGGCGCTGCACGTTGACTCGGCCGAGTACAGCTGCATCAAGGCGATCGTGCTGTTCACCACGGATGCGTGTGGCTTGTCGGACGCTGCTCACATAGAAAGTCTGCAAGAGAAATCGCAATGTGCGTTGGAAGAGTACGTGAGAAGTCAGTACCCGAACCAGCCGACCCGGTTCGGGAAATTATTGCTGCGCCTGCCGTCGCTTCGCACCGTGTCCTCTTCGGTAATCGAACAGCTCTTCTTCGTACGTTTGGTAGGTAAAACCCCGATCGAGACTCTCATCCGGGACATGTTACTGTCTGGAAGCAGTTTCAACTGGCCCTACATGCCTATCCAATAG

atggca

M A

atggtagtcgggccgtggcgagacccacagggcgacctgaccagccccccgaacgtgccc

M V V G P W R D P Q G D L T S P P N V P

ggccaggcgggccaagccccgctggctcccgcggggccgcagacgccgcagacgccgagt

G Q A G Q A P L A P A G P Q T P Q T P S

cagtcgggcgggccgcccacgactccgggccagggcagcacgcagggtgacaagcagcag

Q S G G P P T T P G Q G S T Q G D K Q Q

cagcagcagcagccgaacgtggagtgcgtcgtgtgcggagacaaatcgagcggcaagcac

Q Q Q Q P N V E C V V C G D K S S G K H

tacggacagttcacgtgcgagggatgtaaaagtttcttcaagcgcagcgttcgccggaac

Y G Q F T C E G C K S F F K R S V R R N

ctgacctacacgtgccgcgccaatcggaattgtcccatcgaccagcaccaccgcaatcag

L T Y T C R A N R N C P I D Q H H R N Q

tgtcagtactgccgcctcaagaaatgcctcaaagtcgggatgcgacgcgaa**gat**cgtgcg

C Q Y C R L K K C L K V G M R R E **D** R A

ggtctcgggtctgcaatgcaacggtcttctctgtctcccattgcagccgtgcagagggga

G L G S A M Q R S S L S P I A A V Q R G

agactgcctccctcgcagcacccgaatccggcacagtacgccctggttaacggcgaccct

R L P P S Q H P N P A Q Y A L V N G D P

ctgaacggccactcgtacctctcgggctacatctcgctgctgctgcgcgccgaaccttac

L N G H S Y L S G Y I S L L L R A E P Y

cccacgtcgcgctacggcagccagtgcatgcagcccaacaacatcatgggcattgagaac

P T S R Y G S Q C M Q P N N I M G I E N

atctgcgagctggctgcgcgtctcctcttcagcgccgtggagtgggcgaggaacatcccg

I C E L A A R L L F S A V E W A R N I P

ttcttccccgacctgcagatcacggaccaggtggcgctgctacgactcgtgtggagcgag

F F P D L Q I T D Q V A L L R L V W S E

ctgttcgtgctgaacgcggctcagtgcgccatgccgctccacgtggcgcccctgctcgcc

L F V L N A A Q C A M P L H V A P L L A

gccgcgggcctccacgcgtcgcccatgtccgccgatcgcgtggtggccttcatggaccac

A A G L H A S P M S A D R V V A F M D H

atccgcatcttccaggagcaggtggagaagctcaaggcgctgcacgttgactcggccgag

I R I F Q E Q V E K L K A L H V D S A E

tacagctgcatcaaggcgatcgtgctgttcaccacg**gat**gcgtgtggcttgtcggacgct

Y S C I K A I V L F T T **D** A C G L S D A

gctcacatagaaagtctgcaagagaaatcgcaatgtgcgttggaagagtacgtgagaagt

A H I E S L Q E K S Q C A L E E Y V R S

cagtacccgaaccagccgacccggttcgggaaattattgctgcgcctgccgtcgcttcgc

Q Y P N Q P T R F G K L L L R L P S L R

accgtgtcctcttcggtaatcgaacagctcttcttcgtacgtttggtaggtaaaaccccg

T V S S S V I E Q L F F V R L V G K T P

atcgagactctcatccgggacatgttactgtctggaagcagtttcaactggccctacatg

I E T L I R D M L L S G S S F N W P Y M

cctatccaatag

P I Q -

***>P. marinus Nr2f1/2B***

ATGGCAATGGTTGTCGGGCCGTGGCGAGACCCGCAGGACGAGATGGCGGGGCCGCCGAGCCAGACCGGGCAGCCGCCCCTCGCTCCGGCGGCCGGGCCGCACACCCCGCAGACGCCCGGACACGCAGGGCCGCCCCCGACGACTCCCGGACAAGGCAGCACGCAGGGGGACAAGCAGCAGTCCAATGTGGAGTGCGTGGTCTGCGGCGACAAATCTAGCGGCAAGCACTACGGACAGTTCACGTGCGAAGGATGTAAAAGTTTTTTCAAACGGAGTGTCCGCCGGAACCTGACCTACACGTGCCGCGCCAACAGGAACTGCCCCATCGACCAGCACCACCGCAACCAGTGTCAGTACTGCCGCCTCAAGAAGTGCCTCAAGGTCGGCATGCGGCGTGAAGCTCTTGTCCCCTTTCCAGCCGTGCAGCGGGGTCGACTGGCGCCCACTCAGCACCCCAACCCGGCGCTGCAGTACGCCCTGGTGAACGGCGACCCTCTGAACGGCCACTCGTACCTCTCGGGCTACATCTCGCTGCTGCTGCGCGCCGAGCCTTACCCCACGTCACGCTACGGCAGCCAGTGCATGCAGCCCAACAACATCATGGGCATTGAGAACATCTGCGAGCTGGCGGCGCGTCTCCTCTTCAGCGCCGTGGAGTGGGCGAGGAACATCCCGTTCTTCCCCGACCTGCAGATCACGGACCAGGTGGCGCTGCTACGGCTCGTGTGGAGCGAGCTGTTCGTGCTGAACGCGGCACAGTGCGCCATGCCGCTCCACGTGGCGCCCCTGCTCGCCGCCGCGGGCCTCCACGCGTCGCCCATGTCCGCCGATCGCGTGGTGGCCTTCATGGACCACATCCGCATCTTCCAGGAGCAGGTGGAGAAGCTCAAGGCGCTGCACGTTGACTCGGCCGAGTACAGCTGCCTCAAGGCCATCGTGCTCTTCTCCACCGACGCGTGTGGCCTCTCGGACGCGGCGCACATCGAGAATCTGCAAGAGAAGTCTCAGTGCGCCCTGGAAGAGTACGTGCGGAGTCAGTACCCGAACCAGCCGACGCGCTTCGGAAAGCTGCTGCTTCGTCTGCCCTCGCTGCGCACGGTGTCCTCGTCTGTCATTGAGCAGCTGTTCTTCGTGCGCCTGGTAGGTAAAACTCCCATAGAGACGCTCATCCGTGACATGCTGCTGTCTGGTAGCAGCTTCAACTGGCCTTACATGCCGATACAATAG

atggcaatggttgtcgggccgtggcgagacccgcag

M A M V V G P W R D P Q

gacgagatggcggggccgccgagccagaccgggcagccgcccctcgctccggcggccggg

D E M A G P P S Q T G Q P P L A P A A G

ccgcacaccccgcagacgcccggacacgcagggccgcccccgacgactcccggacaaggc

P H T P Q T P G H A G P P P T T P G Q G

agcacgcagggggacaagcagcagtccaatgtggagtgcgtggtctgcggcgacaaatct

S T Q G D K Q Q S N V E C V V C G D K S

agcggcaagcactacggacagttcacgtgcgaaggatgtaaaagttttttcaaacggagt

S G K H Y G Q F T C E G C K S F F K R S

gtccgccggaacctgacctacacgtgccgcgccaacaggaactgccccatcgaccagcac

V R R N L T Y T C R A N R N C P I D Q H

caccgcaaccagtgtcagtactgccgcctcaagaagtgcctcaaggtcggcatgcggcgt

H R N Q C Q Y C R L K K C L K V G M R R

gaa**gct**cttgtcccctttccagccgtgcagcggggtcgactggcgcccactcagcacccc

E **A** L V P F P A V Q R G R L A P T Q H P

aacccggcgctgcagtacgccctggtgaacggcgaccctctgaacggccactcgtacctc

N P A L Q Y A L V N G D P L N G H S Y L

tcgggctacatctcgctgctgctgcgcgccgagccttaccccacgtcacgctacggcagc

S G Y I S L L L R A E P Y P T S R Y G S

cagtgcatgcagcccaacaacatcatgggcattgagaacatctgcgagctggcggcgcgt

Q C M Q P N N I M G I E N I C E L A A R

ctcctcttcagcgccgtggagtgggcgaggaacatcccgttcttccccgacctgcagatc

L L F S A V E W A R N I P F F P D L Q I

acggaccaggtggcgctgctacggctcgtgtggagcgagctgttcgtgctgaacgcggca

T D Q V A L L R L V W S E L F V L N A A

cagtgcgccatgccgctccacgtggcgcccctgctcgccgccgcgggcctccacgcgtcg

Q C A M P L H V A P L L A A A G L H A S

cccatgtccgccgatcgcgtggtggccttcatggaccacatccgcatcttccaggagcag

P M S A D R V V A F M D H I R I F Q E Q

gtggagaagctcaaggcgctgcacgttgactcggccgagtacagctgcctcaaggccatc

V E K L K A L H V D S A E Y S C L K A I

gtgctcttctccacc**gac**gcgtgtggcctctcggacgcggcgcacatcgagaatctgcaa

V L F S T **D** A C G L S D A A H I E N L Q

gagaagtctcagtgcgccctggaagagtacgtgcggagtcagtacccgaaccagccgacg

E K S Q C A L E E Y V R S Q Y P N Q P T

cgcttcggaaagctgctgcttcgtctgccctcgctgcgcacggtgtcctcgtctgtcatt

R F G K L L L R L P S L R T V S S S V I

gagcagctgttcttcgtgcgcctggtaggtaaaactcccatagagacgctcatccgtgac

E Q L F F V R L V G K T P I E T L I R D

atgctgctgtctggtagcagcttcaactggccttacatgccgatacaatag

M L L S G S S F N W P Y M P I Q -

***>P. marinus Nr2f1/2C***

ATGGCAATGGTGGTCGGAAATCCATGGCGAGAACCTCCAAACGGAACTCCTATCGCGAGCGGTAACAGCACACAGACCTCGTCGTCATCATCATCCTCTTCATCAGCAGCAGCAGCATCATCAGCAGCCGTGTCTGGAAGTGCTTCGCTATTACTGGGGCAGCAGCAGCAGCAGCAGCAAACCTCAAGTCTGCAGACATCAATCGCGGTTGCGGGAAACGTGAGCAGCGTCGATTCACTATCCTCTTCCTCGTTATCCTCCTCATCATCATCATCATCATCCTCATCCTCCTCGTCGTCCTCGACGTCGTCAGCGCACGGGACAAAGTCGGCGTCCGCCTCTCATCATCATCAACAACAACAGCAGCAGCATCAGCAACAACAACAACATCATCATCAGCAGCAGCAGCAACAGCAGCAGCAGCAGAACGCGGAGTGCGTGGTGTGCGGCGACAAGTCGAGCGGCAAGCACTACGGGCAGCTGACGTGCGAAGGCTGCAAGAGCTTCTTCAAGCGGAGCGTTCGACGCAACCTCAACTACGCGTGCCGCGCGGCGCGCGCCTGTCCCATCGACCAGCACCACCGCAATCAGTGTCAGTACTGCCGCCTCAAGAAGTGCCTCAAAGTGGGCATGCGGCGAGAAGCCGTGCAGCGGGGACGCATGCCTTCGTCCATGCAGCACCCTGCGACTCCGGGGGGCGCGTACGCGCTGCAGCCCAACGGCGGGGACCCCCTGCTGAACGGCGCTGGGGGCCACGCCGCCTCGTACCTGTCGGGCTACATCTCGTTGCTGCTGCGCGCAGAGCCCTATCCGACGTCGCGCTACGGCAGCCAGTGCATGCAGCCCAACAACCTGATGGGCATCGAGAACATCTGCGAGCTCGCCGCGCGCCTCCTTTTCAGCGCCGTCGAGTGGGCCCGGAACATCCCCTTCTTCCCCGAACTACAGGTGGGGGACCAGGTGGCGCTGCTGCGGCTCGTGTGGAGCGAGCTGTTCGTGCTGAACGCCGCGCAGTGCGCCATGCCGCTGCACGTGGCGCCGCTGCTGGCCGCCGCTGGGCTCCACGCGTCGCCCATGTCCGCCGAGCGCGTGGTGGCCTTCATGGACCACATCCGCATCTTCCAGGAGCAGGTGGAGAAGCTCAAGGCGCTGCACGTCGACTCGGCCGAGTACAGCTGCCTCAAGGCCATCGTGCTCTTCACGTCCGACGCGTGCGGCCTCTCGGACACGGCGCACGTGGAGTCTCTGCAGGAGAAGTCCCAGTGCGCACTTGAGGAGTACGTGCGCACGCAGTACCCCAGCCAGCCCACGCGCTTCGGAAAGCTGCTGCTGCGCCTGCCGTCACTGCGCACCGTCTCGTCCGCCGTCATCGAGCAGCTCTTCTTCGTGCGGCTCGTCGGCAAGACGCCCATCGAGACGCTCATTCGCGACATGCTGCTGTCGGGCAGCAGCTTCAACTGGCCCTACATGTCCATACAGTGACGGGCGTCGCGGTGACGGCGGCGGCGGCGACGACGCGGGGCTTCGAATTTCGTTTCCCACCCGCCCGGAGCGAGCAGTACGATGGATTTGGAGTGCCGGGTGCAGGGAATTCTTGCCGGGTCGCCGCGTGGATCGGAGCGGTTGCCGCGTCCTTGGAGTAGCCGCGGCCACCGCGGGAGAGGAGGATGCGGCGTTTGCCCTGGAGCGGGAGTCGCGTTTCAAAGCTGGATGGACCCCAGCTGA

atggcaatg

M A M

gtggtcggaaatccatggcgagaacctccaaacggaactcctatcgcgagcggtaacagc

V V G N P W R E P P N G T P I A S G N S

acacagacctcgtcgtcatcatcatcctcttcatcagcagcagcagcatcatcagcagcc

T Q T S S S S S S S S S A A A A S S A A

gtgtctggaagtgcttcgctattactggggcagcagcagcagcagcagcaaacctcaagt

V S G S A S L L L G Q Q Q Q Q Q Q T S S

ctgcagacatcaatcgcggttgcgggaaacgtgagcagcgtcgattcactatcctcttcc

L Q T S I A V A G N V S S V D S L S S S

tcgttatcctcctcatcatcatcatcatcatcctcatcctcctcgtcgtcctcgacgtcg

S L S S S S S S S S S S S S S S S S T S

tcagcgcacgggacaaagtcggcgtccgcctctcatcatcatcaacaacaacagcagcag

S A H G T K S A S A S H H H Q Q Q Q Q Q

catcagcaacaacaacaacatcatcatcagcagcagcagcaacagcagcagcagcagaac

H Q Q Q Q Q H H H Q Q Q Q Q Q Q Q Q Q N

gcggagtgcgtggtgtgcggcgacaagtcgagcggcaagcactacgggcagctgacgtgc

A E C V V C G D K S S G K H Y G Q L T C

gaaggctgcaagagcttcttcaagcggagcgttcgacgcaacctcaactacgcgtgccgc

E G C K S F F K R S V R R N L N Y A C R

gcggcgcgcgcctgtcccatcgaccagcaccaccgcaatcagtgtcagtactgccgcctc

A A R A C P I D Q H H R N Q C Q Y C R L

aagaagtgcctcaaagtgggcatgcggcgagaa**gcc**gtgcagcggggacgcatgccttcg

K K C L K V G M R R E **A**  V Q R G R M P S

tccatgcagcaccctgcgactccggggggcgcgtacgcgctgcagcccaacggcggggac

S M Q H P A T P G G A Y A L Q P N G G D

cccctgctgaacggcgctgggggccacgccgcctcgtacctgtcgggctacatctcgttg

P L L N G A G G H A A S Y L S G Y I S L

ctgctgcgcgcagagccctatccgacgtcgcgctacggcagccagtgcatgcagcccaac

L L R A E P Y P T S R Y G S Q C M Q P N

aacctgatgggcatcgagaacatctgcgagctcgccgcgcgcctccttttcagcgccgtc

N L M G I E N I C E L A A R L L F S A V

gagtgggcccggaacatccccttcttccccgaactacag**gtg**ggggaccaggtggcgctg

E W A R N I P F F P E L Q **V**  G D Q V A L

ctgcggctcgtgtggagcgagctgttcgtgctgaacgccgcgcagtgcgccatgccgctg

L R L V W S E L F V L N A A Q C A M P L

cacgtggcgccgctgctggccgccgctgggctccacgcgtcgcccatgtccgccgagcgc

H V A P L L A A A G L H A S P M S A E R

gtggtggccttcatggaccacatccgcatcttccaggagcaggtggagaagctcaaggcg

V V A F M D H I R I F Q E Q V E K L K A

ctgcacgtcgactcggccgagtacagctgcctcaaggccatcgtgctcttcacgtcc**gac**

L H V D S A E Y S C L K A I V L F T S **D**

gcgtgcggcctctcggacacggcgcacgtggagtctctgcaggagaagtcccagtgcgca

A C G L S D T A H V E S L Q E K S Q C A

cttgaggagtacgtgcgcacgcagtaccccagccagcccacgcgcttcggaaagctgctg

L E E Y V R T Q Y P S Q P T R F G K L L

ctgcgcctgccgtcactgcgcaccgtctcgtccgccgtcatcgagcagctcttcttcgtg

L R L P S L R T V S S A V I E Q L F F V

cggctcgtcggcaagacgcccatcgagacgctcattcgcgacatgctgctgtcgggcagc

R L V G K T P I E T L I R D M L L S G S

agcttcaactggccctacatgtccatacagtga

S F N W P Y M S I Q -

***>D. rerio nr2f1a***

ATGGCAATGGTAGTTAGCGTCTGGCGAGATCCGCAGGAAGACGTGGCTGGAGGACCTCCGAGCGGCCCCAATCCAGCAGCTCAGCCGGCGAGGGAGCAACAGCAGGCGGCGTCAGCGGCACCACACACTCCGCAGACCCCCAGTCAGCCAGGACCTCCGTCCACACCAGGGACGGCTGGCGACAAGGGCAGCCAGAATTCTGGACAGAGTCAACAGCATATAGAATGTGTTGTTTGCGGGGACAAATCAAGCGGCAAGCACTATGGTCAATTCACCTGCGAAGGATGCAAAAGTTTCTTCAAGAGGAGTGTCCGAAGGAACTTAACATATACATGTCGTGCCAACAGGAACTGTCCTATTGACCAACACCATCGTAATCAGTGCCAATATTGTCGGCTGAAGAAGTGTTTAAAAGTGGGCATGCGGCGGGAAGCGGTTCAGCGAGGAAGAATGCCTCCAACCCAACCGAACCCGGGCCAGTATGCGCTAACGAATGGGGATCCTCTGAACGGTCATTGCTATCTCTCCGGATACATCTCGTTACTACTTCGGGCCGAGCCTTATCCTACGTCTCGCTATGGCAGCCAGTGCATGCAGCCCAATAATATTATGGGGATCGAGAACATCTGTGAGCTTGCAGCTCGTCTGCTCTTCAGTGCAGTGGAATGGGCAAGGAACATCCCTTTCTTTCCCGATCTGCAGATCACCGACCAGGTGTCATTGCTCAGACTGACATGGAGCGAGTTATTTGTGCTAAACGCGGCTCAGTGTTCCATGCCCCTACATGTGGCCCCACTGCTCGCCGCTGCCGGCCTGCACGCCTCGCCCATGTCGGCGGATCGCGTCGTGGCCTTCATGGATCACATTCGTATCTTCCAGGAGCAGGTCGAGAAGCTCAAAGCACTGCACGTCGATTCTGCTGAATACAGCTGCATTAAGGCAATAGTGCTCTTCACGTCAGACGCTTGCGGCCTGTCAGATGCTGCACACATCGAGAGTCTGCAGGAGAAGTCTCAGTGCGCCCTGGAGGAGTACGTGAGGAGCCAGTACCCGAACCAGCCCAGTCGCTTTGGCAAGCTTTTACTGCGACTGCCTTCTCTCCGCACTGTCTCTTCGTCAGTAATTGAACAGCTGTTCTTCGTTCGCTTGGTAGGTAAAACTCCCATTGAAACCCTCATCAGGGATATGTTATTATCCGGGAGCAGCTTCAACTGGCCCTACATGTCCATTCAATGA

atggcaatggtagttagcgtctggcgagatccgcaggaagacgtggctggaggacctccg

M A M V V S V W R D P Q E D V A G G P P

agcggccccaatccagcagctcagccggcgagggagcaacagcaggcggcgtcagcggca

S G P N P A A Q P A R E Q Q Q A A S A A

ccacacactccgcagacccccagtcagccaggacctccgtccacaccagggacggctggc

P H T P Q T P S Q P G P P S T P G T A G

gacaagggcagccagaattctggacagagtcaacagcatatagaatgtgttgtttgcggg

D K G S Q N S G Q S Q Q H I E C V V C G

gacaaatcaagcggcaagcactatggtcaattcacctgcgaaggatgcaaaagtttcttc

D K S S G K H Y G Q F T C E G C K S F F

aagaggagtgtccgaaggaacttaacatatacatgtcgtgccaacaggaactgtcctatt

K R S V R R N L T Y T C R A N R N C P I

gaccaacaccatcgtaatcagtgccaatattgtcggctgaagaagtgtttaaaagtgggc

D Q H H R N Q C Q Y C R L K K C L K V G

atgcggcgggaa**gcg**gttcagcgaggaagaatgcctccaacccaaccgaacccgggccag

M R R E **A** V Q R G R M P P T Q P N P G Q

tatgcgctaacgaatggggatcctctgaacggtcattgctatctctccggatacatctcg

Y A L T N G D P L N G H C Y L S G Y I S

ttactacttcgggccgagccttatcctacgtctcgctatggcagccagtgcatgcagccc

L L L R A E P Y P T S R Y G S Q C M Q P

aataatattatggggatcgagaacatctgtgagcttgcagctcgtctgctcttcagtgca

N N I M G I E N I C E L A A R L L F S A

gtggaatgggcaaggaacatccctttctttcccgatctgcagatcaccgaccaggtgtca

V E W A R N I P F F P D L Q I T D Q V S

ttgctcagactgacatggagcgagttatttgtgctaaacgcggctcagtgttccatgccc

L L R L T W S E L F V L N A A Q C S M P

ctacatgtggccccactgctcgccgctgccggcctgcacgcctcgcccatgtcggcggat

L H V A P L L A A A G L H A S P M S A D

cgcgtcgtggccttcatggatcacattcgtatcttccaggagcaggtcgagaagctcaaa

R V V A F M D H I R I F Q E Q V E K L K

gcactgcacgtcgattctgctgaatacagctgcattaaggcaatagtgctcttcacgtca

A L H V D S A E Y S C I K A I V L F T S

**gac**gcttgcggcctgtcagatgctgcacacatcgagagtctgcaggagaagtctcagtgc

**D** A C G L S D A A H I E S L Q E K S Q C

gccctggaggagtacgtgaggagccagtacccgaaccagcccagtcgctttggcaagctt

A L E E Y V R S Q Y P N Q P S R F G K L

ttactgcgactgccttctctccgcactgtctcttcgtcagtaattgaacagctgttcttc

L L R L P S L R T V S S S V I E Q L F F

gttcgcttggtaggtaaaactcccattgaaaccctcatcagggatatgttattatccggg

V R L V G K T P I E T L I R D M L L S G

agcagcttcaactggccctacatgtccattcaatga

S S F N W P Y M S I Q -

***>D. rerio nr2f1b***

ATGGCCATGGTGGTGAGCGCGTGGAGAGATCCGCAGGAGGAGCTGGCAGCGGTGGATGATCAGAGCGCGGCCGGGAGAGAGCACCTCCAGCACCGGCACTCGCCGAAGTCCGCGGAGGAAAAAGCGCAGATCGCGGCGCAAAACCAGCAGCACGTCGAGTGTGTGGTGTGCGGGGATAAATCCAGCGGGAAGCACTACGGACAGTTCACCTGCGAGGGATGCAAAAGTTTCTTCAAGCGAAGTGTCCGGCGGAATTTATCGTACACGTGCCGCGCCAACAGAAACTGCCCGGTGGATCAGCACCACCGCAACCAGTGCCAGTACTGCCGCCTGAAGAAGTGCCTGAAAGTGGGCATGAGACGCGAAGCGGTTCAGCGAGGCAGAATGCCACCGAACCAGCCGAACCCGAGCCATTACGCGCTGACCAACGGAGATCACCTGAACGGCCAGTGCTATTTATCCGGATACATATCGCTTCTCCTCCGGGCCGAGCCCTATCCAGCCTCGAGATACGGCAACCAGTGCATGCAGTCCGGCAACATCATGGGCATAGAGAACATCTGCGAGCTGGCGGCCCGTCTGCTCTTCAGCGCGGTGGAGTGGGCCAGGAACATCCCGTTCTTCCCCGATCTGCAAATCACGGATCAGGTGTCTCTGCTCAGACTCACCTGGAGCGAGTTGTTCGTGCTGAACGCCGCGCAGAGCTCTATGCCGCTGCACGTGGCCCC[T](http://uswest.ensembl.org/Danio_rerio/ZMenu/TextSequence?db=core;factorytype=Location;g=ENSDARG00000017168;r=10:43797130-43808197;t=ENSDART00000027242;v=rs510526281;vf=7860685)CTGCTGGCCGCCGCAGGCCTGCACGCGTCGCCGATGTCCGCGGACCGAGTCGTGGCCTTCATGGATCATATTCGCTTCTTCCAGGAGCAGGTGGAGAAACTGAAGGCCCTGCAGGTGGACTCCGCCGAATACAGCTGTGCCAAAGCCATAGTGCTGTTCACCTCAGATGCATGCGGCCTTTCAGACATCCCGCACATTGAAGGTCTCCAGGAGAAATCTCAATGTGCCCTGGAGGAGTATGTGCGGAGTCAGTACCCGAACCAGCCCACCCGCTTCGGCAAACTGCTGCTCCGGCTCCCGGCGCTCCGCATGGTCTCCTCCTCCGTCATTGAACAGCTTTTCTTCGTTCGGCTGGTGGGAAAAACACCCATTGAGACGCTCATCAGGGATATGCTGCTATCTGGGAGCAGTTTCAACTGGCCCTACATGCCTATTCAATAA

atggccatggtggtgagcgcgtggagagatccgcaggaggagctggcagcggtggatgat

M A M V V S A W R D P Q E E L A A V D D

cagagcgcggccgggagagagcacctccagcaccggcactcgccgaagtccgcggaggaa

Q S A A G R E H L Q H R H S P K S A E E

aaagcgcagatcgcggcgcaaaaccagcagcacgtcgagtgtgtggtgtgcggggataaa

K A Q I A A Q N Q Q H V E C V V C G D K

tccagcgggaagcactacggacagttcacctgcgagggatgcaaaagtttcttcaagcga

S S G K H Y G Q F T C E G C K S F F K R

agtgtccggcggaatttatcgtacacgtgccgcgccaacagaaactgcccggtggatcag

S V R R N L S Y T C R A N R N C P V D Q

caccaccgcaaccagtgccagtactgccgcctgaagaagtgcctgaaagtgggcatgaga

H H R N Q C Q Y C R L K K C L K V G M R

cgcgaa**gcg**gttcagcgaggcagaatgccaccgaaccagccgaacccgagccattacgcg

R E **A** V Q R G R M P P N Q P N P S H Y A

ctgaccaacggagatcacctgaacggccagtgctatttatccggatacatatcgcttctc

L T N G D H L N G Q C Y L S G Y I S L L

ctccgggccgagccctatccagcctcgagatacggcaaccagtgcatgcagtccggcaac

L R A E P Y P A S R Y G N Q C M Q S G N

atcatgggcatagagaacatctgcgagctggcggcccgtctgctcttcagcgcggtggag

I M G I E N I C E L A A R L L F S A V E

tgggccaggaacatcccgttcttccccgatctgcaaatcacggatcaggtgtctctgctc

W A R N I P F F P D L Q I T D Q V S L L

agactcacctggagcgagttgttcgtgctgaacgccgcgcagagctctatgccgctgcac

R L T W S E L F V L N A A Q S S M P L H

gtggcccctctgctggccgccgcaggcctgcacgcgtcgccgatgtccgcggaccgagtc

V A P L L A A A G L H A S P M S A D R V

gtggccttcatggatcatattcgcttcttccaggagcaggtggagaaactgaaggccctg

V A F M D H I R F F Q E Q V E K L K A L

caggtggactccgccgaatacagctgtgccaaagccatagtgctgttcacctca**gat**gca

Q V D S A E Y S C A K A I V L F T S **D** A

tgcggcctttcagacatcccgcacattgaaggtctccaggagaaatctcaatgtgccctg

C G L S D I P H I E G L Q E K S Q C A L

gaggagtatgtgcggagtcagtacccgaaccagcccacccgcttcggcaaactgctgctc

E E Y V R S Q Y P N Q P T R F G K L L L

cggctcccggcgctccgcatggtctcctcctccgtcattgaacagcttttcttcgttcgg

R L P A L R M V S S S V I E Q L F F V R

ctggtgggaaaaacacccattgagacgctcatcagggatatgctgctatctgggagcagt

L V G K T P I E T L I R D M L L S G S S

ttcaactggccctacatgcctattcaataa

F N W P Y M P I Q -

***>D. rerio nr2f2***

ATGGCAATGGTAGTGTGGAGAGGCTCCCAGGACGATGTGGCCGAGACCCATGGCACCCTCTCATCGCAAACCCAAGGAGGACTATCCCTTCCGACCC

CTCAACCAGGCCAGCTGGGTCTGACGGCCTCTCAGGTTGCCCCTCCGACCCCTCAGACACCCGTTCAAGGACCCCCGAACAACAATAACAACACACAGTCAACCCCGACGAACCAGACGACGCAGAGCCAGTCGGAAAAGCAGCAGCCACAGCATATAGAGTGCGTGGTTTGCGGGGACAAATCCAGCGGTAAACACTATGGCCAGTTCACTTGCGAGGGGTGCAAAAGCTTTTTCAAACGGAGCGTACGAAGGAACCTCACTTACACATGCCGTGCCAACAGGAATTGTCCCATTGACCAGCACCATCGCAATCAGTGTCAGTACTGCCGCCTCAAAAAATGCCTCAAAGTTGGCATGAGACGGGAAGCCGTGCAAAGGGGACGAATGCCACCCACACAGCCACACCATGGTCAGTTCGCCTTGACAAATGGGGACCCACTGCACTGCCATTCCTACTTATCCGGATATATCTCTCTACTACTACGAGCGGAGCCCTACCCAACTTCTCGGTATGGCAGTCAATGCATGCAGCCCAACAACATCATGGGCATCGAGAACATTTGTGAACTGGCAGCCAGGATGCTGTTTAGTGCGGTAGAGTGGGCCAGGAATATTCCCTTCTTCCCAGACCTCCAAATCACCGACCAGGTTGCCCTTTTGAGGTTGACCTGGAGTGAGTTGTTTGTGCTTAACGCTGCTCAATGCTCCATGCCTCTCCATGTGGCTCCACTTCTGGCGGCGGCTGGGCTCCATGCCTCCCCCATGTCTGCGGACAGAGTGGTCGCCTTTATGGACCACATTAGGATCTTCCAAGAACAAGTAGAAAAGCTCAAAGCTTTGCACGTTGACTCTGCTGAATACAGTTGTTTAAAGGCCATCGTTTTATTCACTTCAGATGCGTGTGGCCTGTCAGATGTGGCCCATGTGGAAAGTTTGCAAGAAAAGTCCCAGTGCGCTTTGGAGGAGTACGTTCGGAGCCAGTATCCCAACCAGCCAACTCGATTTGGGAAGTTATTACTGCGCTTGCCCTCTCTCCGTACAGTCTCGTCTTCGGTCATAGAGCAATTATTTTTCGTCCGATTGGTAGGTAAAACCCCAATTGAAACCCTCATCAGGGATATGTTGCTGTCGGGGAGCAGTTTTAACTGGCCTTATATGTCGATTCAGTAG

atggcaatggtagtgtggagaggctcccaggacgatgtggccgagacccatggcaccctc

M A M V V W R G S Q D D V A E T H G T L

tcatcgcaaacccaaggaggactatcccttccgacccctcaaccaggccagctgggtctg

S S Q T Q G G L S L P T P Q P G Q L G L

acggcctctcaggttgcccctccgacccctcagacacccgttcaaggacccccgaacaac

T A S Q V A P P T P Q T P V Q G P P N N

aataacaacacacagtcaaccccgacgaaccagacgacgcagagccagtcggaaaagcag

N N N T Q S T P T N Q T T Q S Q S E K Q

cagccacagcatatagagtgcgtggtttgcggggacaaatccagcggtaaacactatggc

Q P Q H I E C V V C G D K S S G K H Y G

cagttcacttgcgaggggtgcaaaagctttttcaaacggagcgtacgaaggaacctcact

Q F T C E G C K S F F K R S V R R N L T

tacacatgccgtgccaacaggaattgtcccattgaccagcaccatcgcaatcagtgtcag

Y T C R A N R N C P I D Q H H R N Q C Q

tactgccgcctcaaaaaatgcctcaaagttggcatgagacgggaa**gcc**gtgcaaagggga

Y C R L K K C L K V G M R R E  **A** V Q R G

cgaatgccacccacacagccacaccatggtcagttcgccttgacaaatggggacccactg

R M P P T Q P H H G Q F A L T N G D P L

cactgccattcctacttatccggatatatctctctactactacgagcggagccctaccca

H C H S Y L S G Y I S L L L R A E P Y P

acttctcggtatggcagtcaatgcatgcagcccaacaacatcatgggcatcgagaacatt

T S R Y G S Q C M Q P N N I M G I E N I

tgtgaactggcagccaggatgctgtttagtgcggtagagtgggccaggaatattcccttc

C E L A A R M L F S A V E W A R N I P F

ttcccagacctccaaatcaccgaccaggttgcccttttgaggttgacctggagtgagttg

F P D L Q I T D Q V A L L R L T W S E L

tttgtgcttaacgctgctcaatgctccatgcctctccatgtggctccacttctggcggcg

F V L N A A Q C S M P L H V A P L L A A

gctgggctccatgcctcccccatgtctgcggacagagtggtcgcctttatggaccacatt

A G L H A S P M S A D R V V A F M D H I

aggatcttccaagaacaagtagaaaagctcaaagctttgcacgttgactctgctgaatac

R I F Q E Q V E K L K A L H V D S A E Y

agttgtttaaaggccatcgttttattcacttcag**a**tgcgtgtggcctgtcagatgtggcc

S C L K A I V L F T S **D** A C G L S D V A

catgtggaaagtttgcaagaaaagtcccagtgcgctttggaggagtacgttcggagccag

H V E S L Q E K S Q C A L E E Y V R S Q

tatcccaaccagccaactcgatttgggaagttattactgcgcttgccctctctccgtaca

Y P N Q P T R F G K L L L R L P S L R T

gtctcgtcttcggtcatagagcaattatttttcgtccgattggtaggtaaaaccccaatt

V S S S V I E Q L F F V R L V G K T P I

gaaaccctcatcagggatatgttgctgtcggggagcagttttaactggccttatatgtcg

E T L I R D M L L S G S S F N W P Y M S

attcagtag

I Q -

***>D. rerio nr2f5***

ATGGCAATGGTAGTAAATCAGTGGCAAGAAAACATTTCGGCTGATCCCGGCTCCCAGCTGCAAATGTGCAGTCAGGAGCCTGGCGGGACACCGGGAACTCCCTCTGGGTCAACCCCGGGCAACGATGCGCTCTCCGGAGACAAAATTCCCAATGTGGACTGCATGGTGTGCGGGGACAAATCCAGCGGTAAACACTACGGCCAGTTTACCTGCGAGGGCTGCAAGAGCTTCTTCAAGCGCTCGGTGAGACGGAACCTGAGTTACACCTGCCGGGGAAACCGGGACTGTCCCATTGACCAGCACCATCGGAACCAGTGCCAATACTGCCGGCTCAAGAAGTGCTTGAAAGTCGGAATGAGAAGAGAGGCTGTCCAGAGGGGTCGAATGTCCAACTCTCAGTCAAGTCCAGGACAGTACCTGAGCAATGGAAGTGACCCGTATAATGGTCAGCCGTACCTGTCCGGCTTCATCTCTCTGCTCTTACGGGCTGAACCGTACCCCACGTCCCGCTATGGAGCCCAATGTATGCAGTCGAATAACCTGATGGGCATCGAGAACATATGCGAACTGGCTGCACGTCTGCTGTTCAGCGCTGTGGAGTGGGCCAAAAATATACCTTTCTTTCCAGACCTGCAGCTTATGGATCAGGTGGCGCTGTTACGCATGTCGTGGAGTGAGCTGTTTGTTCTAAATGCTGCTCAGTGCTCAATGCCTCTGCATGTAGCGCCCCTGCTGGCCGCCGCTGGACTACACGCGTCCCCCATGTCTGCCGAGCGTGTGGTGGCCTTCATGGACCATATACGAGTCTTTCAGGAGCAGGTGGAGAAACTGAAAGCCCTTCAGGTGGACACGGCCGAGTACTCCTGCCTGAAGTCTATCGTACTCTTCACCTCTGATGCCATGGGTCTGTCTGATGTGGCCCATGTGGAGAGCATCCAGGAGAAGTCCCAGTGTGCTCTCGAGGAGTATGTGCGGAACCAGTATCCCAATCAACCCAACCGATTTGGCCGCCTGTTGTTACGGCTTCCCTCCCTGCGCATCGTTTCCTCTCCTGTTATCGAACAGCTGTTCTTTGTGCGGCTGGTAGGCAAAACGCCCATCGA[G](http://uswest.ensembl.org/Danio_rerio/ZMenu/TextSequence?db=core;factorytype=Location;g=ENSDARG00000033172;r=16:46294337-46327301;t=ENSDART00000040769;v=rs515424879;vf=3262978)ACCCTGTTACGTGACATGCTGCTTTCCGGCTCCAGCTACAACTGGCCTTACATGCCTGTCCAGAGGGACCG[A](http://uswest.ensembl.org/Danio_rerio/ZMenu/TextSequence?db=core;factorytype=Location;g=ENSDARG00000033172;r=16:46294337-46327301;t=ENSDART00000040769;v=rs503999613;vf=3262979)CCCATCTCGATCCATTACAATGAGAACGGGCCCTGA

agtaaatcagtggcaagaaaacatttcggctgatcccggctcccagctgcaaatgtgcagt

V N Q W Q E N I S A D P G S Q L Q M C S

caggagcctggcgggacaccgggaactccctctgggtcaaccccgggcaacgatgcgctc

Q E P G G T P G T P S G S T P G N D A L

tccggagacaaaattcccaatgtggactgcatggtgtgcggggacaaatccagcggtaaa

S G D K I P N V D C M V C G D K S S G K

cactacggccagtttacctgcgagggctgcaagagcttcttcaagcgctcggtgagacgg

H Y G Q F T C E G C K S F F K R S V R R

aacctgagttacacctgccggggaaaccgggactgtcccattgaccagcaccatcggaac

N L S Y T C R G N R D C P I D Q H H R N

cagtgccaatactgccggctcaagaagtgcttgaaagtcggaatgagaagagag**gct**gtc

Q C Q Y C R L K K C L K V G M R R E **A** V

cagaggggtcgaatgtccaactctcagtcaagtccaggacagtacctgagcaatggaagt

Q R G R M S N S Q S S P G Q Y L S N G S

gacccgtataatggtcagccgtacctgtccggcttcatctctctgctcttacgggctgaa

D P Y N G Q P Y L S G F I S L L L R A E

ccgtaccccacgtcccgctatggagcccaatgtatgcagtcgaataacctgatgggcatc

P Y P T S R Y G A Q C M Q S N N L M G I

gagaacatatgcgaactggctgcacgtctgctgttcagcgctgtggagtgggccaaaaat

E N I C E L A A R L L F S A V E W A K N

atacctttctttccagacctgcagcttatggatcag**gtg**gcgctgttacgcatgtcgtgg

I P F F P D L Q L M D Q **V**  A L L R M S W

agtgagctgtttgttctaaatgctgctcagtgctcaatgcctctgcatgtagcgcccctg

S E L F V L N A A Q C S M P L H V A P L

ctggccgccgctggactacacgcgtcccccatgtctgccgagcgtgtggtggccttcatg

L A A A G L H A S P M S A E R V V A F M

gaccatatacgagtctttcaggagcaggtggagaaactgaaagcccttcaggtggacacg

D H I R V F Q E Q V E K L K A L Q V D T

gccgagtactcctgcctgaagtctatcgtactcttcacctctg**a**tgccatgggtctgtct

A E Y S C L K S I V L F T S **D** A M G L S

gatgtggcccatgtggagagcatccaggagaagtcccagtgtgctctcgaggagtatgtg

D V A H V E S I Q E K S Q C A L E E Y V

cggaaccagtatcccaatcaacccaaccgatttggccgcctgttgttacggcttccctcc

R N Q Y P N Q P N R F G R L L L R L P S

ctgcgcatcgtttcctctcctgttatcgaacagctgttctttgtgcggctggtaggcaaa

L R I V S S P V I E Q L F F V R L V G K

acgcccatcgagaccctgttacgtgacatgctgctttccggctccagctacaactggcct

T P I E T L L R D M L L S G S S Y N W P

tacatgcctgtccagagggaccgacccatctcgatccattacaatgagaacgggccctga

Y M P V Q R D R P I S I H Y N E N G P -

***>D. rerio nr2f6a***

ATGGCCATGGTGAGAGGGGGGTGGGGAGATCCCAACGGGGAAACAAATGGACTTGGTGACAAGGGTTACCTGAGGGGAGATGAAGATGACGGGTCACCGCAAGGAGGCGGCAGCGACATGGAGGCCGGTGAGGATGATAAGGGATGTGTGGTGGACTGTGTTGTCTGCGGCGACAAGTCCAGCGGGAAACACTATGGTGTGTTCACCTGTGAGGGCTGCAAGAGCTTCTTTAAACGAAGTGTCAGACGAAACCTTAACTACACCTGCAGATCAAACCGAGACTGCCAAATTGACCAGCATCATCGCAACCAGTGTCAATACTGCCGTTTAAAGAAGTGTTTCCGAGTGGGAATGCGTAAAGAAGCAGTTCAGCGTGGACGGATCCCACCTTCACATTCGAGCCTGAGCCCATCTACAACTCCAGTAGGCGGTAATGCCGGCGGTGGTGTGAGCGAGTTCTACAATGGGCAGCCGGTGTCCGAGCTCATCTCCCAGCTCCTGCGGGCAGAGCCTTACCCTAACAGCCGCTACAGCCACCAGTACAACCAGCAGATGCAAGGCGGTGGTGGCGGTGGATCTGGCATGGGCATCGACAGCATCTGTGAGCTCGCTGCCAGACTCCTGTTTAGCATCATCGAATGGGCCCGAAACATTCCGTACTTCCCCGAATTGCCAGTATCGGAGCAGGTGGCCTTGCTGCGGCTCAGCTGGAGCGAACTGTTTATTCTGAACGCAGCTCAATCTGCCCTGCCACTGCACATGGCCCCGCTGCTGGCCGCCGCAGGCTTCCACTCCTCACCCATGTCCGCGGAAAGGGTGGTGTCCTTCATGGACCAGGTGCGCGTCTTCCAGGACCAGGTGGAGAAGCTGACGCGTCTGCAGGTGGATTCGGCAGAGTACAGCTGTCTCAAGGCCATAGCGCTCTTCTCGCCAGATGCGTGTGGGCTGACAGATCCCGCTCATGTAGAAAGCCTGCAGGAGAAAGCTCAGGTGGCTCTTACCGAGTACGAGCGCATGCAGTACCCCGGGCAGCCCCAGCGTTTCGGACGGCTCCTGCTGCGACTGCCCGCTCTGAGGGCCGTGCCCGCCAGCCTCATCTCTCAGCTCTTCTTCATGCGGCTCGTGGGCAAAACGCCCATCGAGACACTCATCCGTGACATGCAGCTGTCTGGCAGCTCCATCAGTTGGCCGTATGCTCCTGGACAATAA

atggccatggtgagaggggggtggggagatcccaacggggaaacaaatggacttggtgac

M A M V R G G W G D P N G E T N G L G D

aagggttacctgaggggagatgaagatgacgggtcaccgcaaggaggcggcagcgacatg

K G Y L R G D E D D G S P Q G G G S D M

gaggccggtgaggatgataagggatgtgtggtggactgtgttgtctgcggcgacaagtcc

E A G E D D K G C V V D C V V C G D K S

agcgggaaacactatggtgtgttcacctgtgagggctgcaagagcttctttaaacgaagt

S G K H Y G V F T C E G C K S F F K R S

gtcagacgaaaccttaactacacctgc**aga**tcaaaccgagactgccaaattgaccagcat

V R R N L N Y T C **R** S N R D C Q I D Q H

catcgcaaccagtgtcaatactgccgtttaaagaagtgtttccgagtgggaatgcgtaaa

H R N Q C Q Y C R L K K C F R V G M R K

gaa**gca**gttcagcgtggacggatcccaccttcacattcgagcctgagcccatctacaact

E **A** V Q R G R I P P S H S S L S P S T T

ccagtaggcggtaatgccggcggtggtgtgagcgagttctacaatgggcagccggtgtcc

P V G G N A G G G V S E F Y N G Q P V S

gagctcatctcccagctcctgcgggcagagccttaccctaacagccgctacagccaccag

E L I S Q L L R A E P Y P N S R Y S H Q

tacaaccagcagatgcaaggcggtggtggcggtggatctggcatgggcatcgacagcatc

Y N Q Q M Q G G G G G G S G M G I D S I

tgtgagctcgctgccagactcctgtttagcatcatcgaatgggcccgaaacattccgtac

C E L A A R L L F S I I E W A R N I P Y

ttccccgaattgccagtatcggagcaggtggccttgctgcggctcagctggagcgaactg

F P E L P V S E Q V A L L R L S W S E L

tttattctgaacgcagctcaatctgccctgccactgcacatggccccgctgctggccgcc

F I L N A A Q S A L P L H M A P L L A A

gcaggcttccactcctcacccatgtccgcggaaagggtggtgtccttcatggaccaggtg

A G F H S S P M S A E R V V S F M D Q V

cgcgtcttccaggaccaggtggagaagctgacgcgtctgcaggtggattcggcagagtac

R V F Q D Q V E K L T R L Q V D S A E Y

agctgtctcaaggccatagcgctcttctcgccag**a**tgcgtgtgggctgacagatcccgct

S C L K A I A L F S P **D** A C G L T D P A

catgtagaaagcctgcaggagaaagctcaggtggctcttaccgagtacgagcgcatgcag

H V E S L Q E K A Q V A L T E Y E R M Q

taccccgggcagccccagcgtttcggacggctcctgctgcgactgcccgctctgagggcc

Y P G Q P Q R F G R L L L R L P A L R A

gtgcccgccagcctcatctctcagctcttcttcatgcggctcgtgggcaaaacgcccatc

V P A S L I S Q L F F M R L V G K T P I

gagacactcatccgtgacatgcagctgtctggcagctccatcagttggccgtatgctcct

E T L I R D M Q L S G S S I S W P Y A P

ggacaataa

G Q -

***>D. rerio nr2f6b***

ATGGCCATGGTGAGCGGGGGATGGGCCAACCCCAATGGCAGTGCTAATGGACTCGGTGAGAAAGGTTACCTGCGGGGGGAGGAGGAGGGAAGCTCGCC

CCAGGCAGGGAACAGCGATGTGGAAGGTGGCGAGGAGGACAAGGCCTGCGTGGTGGACTGTGTTGTGTGTGGAGACAAATCCAGCGGGAAGCACTATGGTGTTTTCACCTGTGAAGGGTGCAAGAGTTTCTTCAAAAGGAGCATCAGACGGAACCTCAACTACACCTGCAGATCAAACAGAGAATGTCAGATTGATCAGCATCACCGTAACCAGTGTCAGTACTGCCGTCTGAAGAAGTGCTTCCGGGTCGGTATGAGGAAAGAAGCTGTTCAGCGTGGCCGAATTCCTCCATCCCACGCAGGCATCAGCCCAGCCTCCATGGTAGGAGCAGGTGGTGATGTTGGAGGAGGTCCGGGCATGGGTGCTGATTTTTTTAATGGTCAGCCAGTGTCTGAACTCATCTCTCAACTACTGAGAGCAGAGCCGTACCCCAACAGCCGTTATGGAGCCCAGTGTGGCCAGCAGCTCCAAGGCGCCAACAGCTCCATGATGGGCATTGACAATATATGTGAGCTTGCAGCCCGGCTGCTGTTCAGCACTATTGAGTGGGCCAGGAATATTCCCTATTTTCCTGACCTGCCAGTGTCAGAGCAGGTGGCCCTTCTGAGGCTCAGCTGGAGTGAACTGTTTATCCTGAATGCAGCTCAGTCGGCGCTGCCCCTGCACACGGCCCCTCTGCTGGCAGCTGCAGGGTTTCACTCCTCCCCTATGCCCGCTGATCGCGTCGTGTCCTTCATGGACCAGGTGCGGGTCTTTCAGGACCAGGTGGACAAGCTGACACGACTGCAGGTGGATTCTGTTGAATACAGCTGTCTGAAAGCCATCGCTCTGTTCTCACCAGATGCATGCGGGCTGTCGGATCCAGCGCACGTCGAGAGCTTGCAGGAGAAAGCACAGGTGGCCCTGACCGAATACGAGCGGATGCAGTATCCAGGCCAGCCCCAGCGGTTCGGCCGGCTCCTGCTGCGACTTCCCGCTCTGAGAGCCGTTCCCGCCAACCTCATCTCTCAACTCTTCTTCATGCGGCTGGTTGGCAAGACGCCAATCGAAACTCTAATCCGGGACATGCAGCTCTCTGGAAGCTCCATCAGCTGGCCGTATGTGCCTGGACAGTAG

atggccatggtgagcgggggatgggccaaccccaatggcagtgctaatggactcggtgag

M A M V S G G W A N P N G S A N G L G E

aaaggttacctgcggggggaggaggagggaagctcgccccaggcagggaacagcgatgtg

K G Y L R G E E E G S S P Q A G N S D V

gaaggtggcgaggaggacaaggcctgcgtggtggactgtgttgtgtgtggagacaaatcc

E G G E E D K A C V V D C V V C G D K S

agcgggaagcactatggtgttttcacctgtgaagggtgcaagagtttcttcaaaaggagc

S G K H Y G V F T C E G C K S F F K R S

atcagacggaacctcaactacacctgc**aga**tcaaacagagaatgtcagattgatcagcat

I R R N L N Y T C R S N R E C Q I D Q H

caccgtaaccagtgtcagtactgccgtctgaagaagtgcttccgggtcggtatgaggaaa

H R N Q C Q Y C R L K K C F R V G M R K

gaa**gct**gttcagcgtggccgaattcctccatcccacgcaggcatcagcccagcctccatg

E A V Q R G R I P P S H A G I S P A S M

gtaggagcaggtggtgatgttggaggaggtccgggcatgggtgctgatttttttaatggt

V G A G G D V G G G P G M G A D F F N G

cagccagtgtctgaactcatctctcaactactgagagcagagccgtaccccaacagccgt

Q P V S E L I S Q L L R A E P Y P N S R

tatggagcccagtgtggccagcagctccaaggcgccaacagctccatgatgggcattgac

Y G A Q C G Q Q L Q G A N S S M M G I D

aatatatgtgagcttgcagcccggctgctgttcagcactattgagtgggccaggaatatt

N I C E L A A R L L F S T I E W A R N I

ccctattttcctgacctgccagtgtcagagcaggtggcccttctgaggctcagctggagt

P Y F P D L P V S E Q V A L L R L S W S

gaactgtttatcctgaatgcagctcagtcggcgctgcccctgcacacggcccctctgctg

E L F I L N A A Q S A L P L H T A P L L

gcagctgcagggtttcactcctcccctatgcccgctgatcgcgtcgtgtccttcatggac

A A A G F H S S P M P A D R V V S F M D

caggtgcgggtctttcaggaccaggtggacaagctgacacgactgcaggtggattctgtt

Q V R V F Q D Q V D K L T R L Q V D S V

gaatacagctgtctgaaagccatcgctctgttctcacca**gat**gcatgcgggctgtcggat

E Y S C L K A I A L F S P  **D** A C G L S D

ccagcgcacgtcgagagcttgcaggagaaagcacaggtggccctgaccgaatacgagcgg

P A H V E S L Q E K A Q V A L T E Y E R

atgcagtatccaggccagccccagcggttcggccggctcctgctgcgacttcccgctctg

M Q Y P G Q P Q R F G R L L L R L P A L

agagccgttcccgccaacctcatctctcaactcttcttcatgcggctggttggcaagacg

R A V P A N L I S Q L F F M R L V G K T

ccaatcgaaactctaatccgggacatgcagctctctggaagctccatcagctggccgtat

P I E T L I R D M Q L S G S S I S W P Y

Gtgcctggacagtag

V P G Q -

***>H. sapiens NR2F1***

ATGGCAATGGTAGTTAGCAGCTGGCGAGATCCGCAGGACGACGTGGCCGGGGGCAACCCCGGCGGCCCCAACCCCGCAGCGCAGGCGGCCCGCGGCGGCGGCGGCGGCGCCGGCGAGCAGCAGCAGCAGGCGGGCTCGGGCGCGCCGCACACGCCGCAGACCCCGGGCCAGCCCGGAGCGCCCGCCACCCCCGGCACGGCGGGGGACAAGGGCCAGGGCCCGCCCGGTTCGGGCCAGAGCCAGCAGCACATCGAGTGCGTGGTGTGCGGGGACAAGTCGAGCGGCAAGCACTACGGCCAATTCACCTGCGAGGGCTGCAAAAGTTTCTTCAAGAGGAGCGTCCGCAGGAACTTAACTTACACATGCCGTGCCAACAGGAACTGTCCCATCGACCAGCACCACCGCAACCAGTGCCAATACTGCCGCCTCAAGAAGTGCCTCAAAGTGGGCATGAGGCGGGAAGCGGTTCAGCGAGGAAGAATGCCTCCAACCCAGCCCAATCCAGGCCAGTACGCACTCACCAACGGGGACCCCCTCAACGGCCACTGCTACCTGTCCGGCTACATCTCGCTGCTGCTGCGCGCCGAGCCCTACCCCACGTCGCGCTACGGCAGCCAGTGCATGCAGCCCAACAACATTATGGGCATCGAGAACATCTGCGAGCTGGCCGCGCGCCTGCTCTTCAGCGCCGTCGAGTGGGCCCGCAACATCCCCTTCTTCCCGGATCTGCAGATCACCGACCAGGTGTCCCTGCTACGCCTCACCTGGAGCGAGCTGTTCGTGCTCAACGCGGCCCAGTGCTCTATGCCGCTGCACGTGGCGCCGTTGCTGGCCGCCGCCGGCCTGCATGCCTCGCCCATGTCTGCCGACCGCGTCGTGGCCTTCATGGACCACATCCGCATCTTCCAGGAGCAGGTGGAGAAGCTCAAGGCGCTACACGTCGACTCAGCCGAGTACAGCTGCCTCAAAGCCATCGTGCTGTTCACGTCAGACGCCTGTGGCCTGTCGGATGCGGCCCACATCGAGAGCCTGCAGGAGAAGTCGCAGTGCGCACTGGAGGAGTACGTGAGGAGCCAGTACCCCAACCAGCCCAGCCGTTTTGGCAAACTGCTGCTGCGACTGCCCTCGCTGCGCACCGTGTCCTCCTCCGTCATCGAGCAGCTCTTCTTCGTCCGTTTGGTAGGTAAAACCCCCATCGAAACTCTCATCCGCGATATGTTACTGTCTGGGAGCAGCTTCAACTGGCCTTACATGTCCATCCAGTGCTCCTAG

atggcaatggtagttagcagctggcgagatccgcaggacgacgtggccgggggcaacccc

M A M V V S S W R D P Q D D V A G G N P

ggcggccccaaccccgcagcgcaggcggcccgcggcggcggcggcggcgccggcgagcag

G G P N P A A Q A A R G G G G G A G E Q

cagcagcaggcgggctcgggcgcgccgcacacgccgcagaccccgggccagcccggagcg

Q Q Q A G S G A P H T P Q T P G Q P G A

cccgccacccccggcacggcgggggacaagggccagggcccgcccggttcgggccagagc

P A T P G T A G D K G Q G P P G S G Q S

cagcagcacatcgagtgcgtggtgtgcggggacaagtcgagcggcaagcactacggccaa

Q Q H I E C V V C G D K S S G K H Y G Q

ttcacctgcgagggctgcaaaagtttcttcaagaggagcgtccgcaggaacttaacttac

F T C E G C K S F F K R S V R R N L T Y

acatgccgtgccaacaggaactgtcccatcgaccagcaccaccgcaaccagtgccaatac

T C R A N R N C P I D Q H H R N Q C Q Y

tgccgcctcaagaagtgcctcaaagtgggcatgaggcgggaa**gcg**gttcagcgaggaaga

C R L K K C L K V G M R R E **A** V Q R G R

atgcctccaacccagcccaatccaggccagtacgcactcaccaacggggaccccctcaac

M P P T Q P N P G Q Y A L T N G D P L N

ggccactgctacctgtccggctacatctcgctgctgctgcgcgccgagccctaccccacg

G H C Y L S G Y I S L L L R A E P Y P T

tcgcgctacggcagccagtgcatgcagcccaacaacattatgggcatcgagaacatctgc

S R Y G S Q C M Q P N N I M G I E N I C

gagctggccgcgcgcctgctcttcagcgccgtcgagtgggcccgcaacatccccttcttc

E L A A R L L F S A V E W A R N I P F F

ccggatctgcagatcaccgaccaggtgtccctgctacgcctcacctggagcgagctgttc

P D L Q I T D Q V S L L R L T W S E L F

gtgctcaacgcggcccagtgctctatgccgctgcacgtggcgccgttgctggccgccgcc

V L N A A Q C S M P L H V A P L L A A A

ggcctgcatgcctcgcccatgtctgccgaccgcgtcgtggccttcatggaccacatccgc

G L H A S P M S A D R V V A F M D H I R

atcttccaggagcaggtggagaagctcaaggcgctacacgtcgactcagccgagtacagc

I F Q E Q V E K L K A L H V D S A E Y S

tgcctcaaagccatcgtgctgttcacgtcag**a**cgcctgtggcctgtcggatgcggcccac

C L K A I V L F T S **D** A C G L S D A A H

atcgagagcctgcaggagaagtcgcagtgcgcactggaggagtacgtgaggagccagtac

I E S L Q E K S Q C A L E E Y V R S Q Y

cccaaccagcccagccgttttggcaaactgctgctgcgactgccctcgctgcgcaccgtg

P N Q P S R F G K L L L R L P S L R T V

tcctcctccgtcatcgagcagctcttcttcgtccgtttggtaggtaaaacccccatcgaa

S S S V I E Q L F F V R L V G K T P I E

actctcatccgcgatatgttactgtctgggagcagcttcaactggccttacatgtccatc

T L I R D M L L S G S S F N W P Y M S I

cagtgctcctag

Q C S -

***>H. sapiens NR2F2***

ATGGCAATGGTAGTCAGCACGTGGCGCGACCCCCAGGACGAGGTGCCCGGCTCACAGGGCAGCCAGGCCTCGCAGGCGCCGCCCGTGCCCGGCCCGCCGCCCGGCGCCCCGCACACGCCACAGACGCCCGGCCAAGGGGGCCCAGCCAGCACGCCAGCCCAGACGGCGGCCGGTGGCCAGGGCGGCCCTGGCGGCCCGGGTAGCGACAAGCAGCAGCAGCAGCAACACATCGAGTGCGTGGTGTGCGGAGACAAGTCGAGCGGCAAGCACTACGGCCAGTTCACGTGCGAGGGCTGCAAGAGCTTCTTCAAGCGCAGCGTGCGGAGGAACCTGAGCTACACGTGCCGCGCCAACCGGAACTGTCCCATCGACCAGCACCATCGCAACCAGTGCCAGTACTGCCGCCTCAAAAAGTGCCTCAAAGTGGGCATGAGACGGGAAGCGGTGCAGAGGGGCAGGATGCCGCCGACCCAGCCGACCCACGGGCAGTTCGCGCTGACCAACGGGGATCCCCTCAACTGCCACTCGTACCTGTCCGGATATATTTCCCTGCTGTTGCGCGCGGAGCCCTATCCCACGTCGCGCTTCGGCAGCCAATGCATGCAGCCCAACAACATCATGGGTATCGAGAACATTTGCGAACTGGCCGCGAGGATGCTCTTCAGCGCCGTCGAGTGGGCCCGGAACATCCCCTTCTTCCCCGACCTGCAGATCACGGACCAGGTGGCCCTGCTTCGCCTCACCTGGAGCGAGCTGTTTGTGTTGAATGCGGCGCAGTGCTCCATGCCCCTCCACGTCGCCCCGCTCCTGGCCGCCGCCGGCCTGCATGCTTCGCCCATGTCCGCCGACCGGGTGGTCGCCTTTATGGACCACATACGGATCTTCCAAGAGCAAGTGGAGAAGCTCAAGGCGCTGCACGTTGACTCAGCCGAGTACAGCTGCCTCAAGGCCATAGTCCTGTTCACCTCAGATGCCTGTGGTCTCTCTGATGTAGCCCATGTGGAAAGCTTGCAGGAAAAGTCTCAGTGTGCTTTGGAAGAATACGTTAGGAGCCAGTACCCCAACCAGCCGACGAGATTCGGAAAGCTTTTGCTTCGCCTCCCTTCCCTCCGCACCGTCTCCTCCTCAGTCATAGAGCAATTGTTTTTCGTCCGTTTGGTAGGTAAAACCCCCATCGAAACCCTCATCCGGGATATGTTACTGTCCGGCAGCAGTTTTAACTGGCCGTATATGGCAATTCAATAA

atggcaatggtagtcagcacgtggcgcgacccccaggacgaggtgcccggctcacagggc

M A M V V S T W R D P Q D E V P G S Q G

agccaggcctcgcaggcgccgcccgtgcccggcccgccgcccggcgccccgcacacgcca

S Q A S Q A P P V P G P P P G A P H T P

cagacgcccggccaagggggcccagccagcacgccagcccagacggcggccggtggccag

Q T P G Q G G P A S T P A Q T A A G G Q

ggcggccctggcggcccgggtagcgacaagcagcagcagcagcaacacatcgagtgcgtg

G G P G G P G S D K Q Q Q Q Q H I E C V

gtgtgcggagacaagtcgagcggcaagcactacggccagttcacgtgcgagggctgcaag

V C G D K S S G K H Y G Q F T C E G C K

agcttcttcaagcgcagcgtgcggaggaacctgagctacacgtgccgcgccaaccggaac

S F F K R S V R R N L S Y T C R A N R N

tgtcccatcgaccagcaccatcgcaaccagtgccagtactgccgcctcaaaaagtgcctc

C P I D Q H H R N Q C Q Y C R L K K C L

aaagtgggcatgagacgggaa**gcg**gtgcagaggggcaggatgccgccgacccagccgacc

K V G M R R E **A** V Q R G R M P P T Q P T

cacgggcagttcgcgctgaccaacggggatcccctcaactgccactcgtacctgtccgga

H G Q F A L T N G D P L N C H S Y L S G

tatatttccctgctgttgcgcgcggagccctatcccacgtcgcgcttcggcagccaatgc

Y I S L L L R A E P Y P T S R F G S Q C

atgcagcccaacaacatcatgggtatcgagaacatttgcgaactggccgcgaggatgctc

M Q P N N I M G I E N I C E L A A R M L

ttcagcgccgtcgagtgggcccggaacatccccttcttccccgacctgcagatcacggac

F S A V E W A R N I P F F P D L Q I T D

caggtggccctgcttcgcctcacctggagcgagctgtttgtgttgaatgcggcgcagtgc

Q V A L L R L T W S E L F V L N A A Q C

tccatgcccctccacgtcgccccgctcctggccgccgccggcctgcatgcttcgcccatg

S M P L H V A P L L A A A G L H A S P M

tccgccgaccgggtggtcgcctttatggaccacatacggatcttccaagagcaagtggag

S A D R V V A F M D H I R I F Q E Q V E

aagctcaaggcgctgcacgttgactcagccgagtacagctgcctcaaggccatagtcctg

K L K A L H V D S A E Y S C L K A I V L

ttcacctcag**a**tgcctgtggtctctctgatgtagcccatgtggaaagcttgcaggaaaag

F T S  **D** A C G L S D V A H V E S L Q E K

tctcagtgtgctttggaagaatacgttaggagccagtaccccaaccagccgacgagattc

S Q C A L E E Y V R S Q Y P N Q P T R F

ggaaagcttttgcttcgcctcccttccctccgcaccgtctcctcctcagtcatagagcaa

G K L L L R L P S L R T V S S S V I E Q

ttgtttttcgtccgtttggtaggtaaaacccccatcgaaaccctcatccgggatatgtta

L F F V R L V G K T P I E T L I R D M L

ctgtccggcagcagttttaactggccgtatatggcaattcaataa

L S G S S F N W P Y M A I Q -

***>H. sapiens NR2F6***

ATGGCCATGGTGACCGGCGGCTGGGGCGGCCCCGGCGGCGACACGAACGGCGTGGACAAGGCGGGCGGCTACCCGCGCGCGGCCGAGGACGACTCGGCCTCGCCCCCCGGTGCCGCCAGCGACGCCGAGCCGGGCGACGAGGAGCGGCCGGGGCTGCAGGTGGACTGCGTGGTGTGCGGGGACAAGTCGAGCGGCAAGCATTACGGTGTCTTCACCTGCGAGGGCTGCAAGAGCTTTTTCAAGCGAAGCATCCGCCGCAACCTCAGCTACACCTGCCGGTCCAACCGTGACTGCCAGATCGACCAGCACCACCGGAACCAGTGCCAGTACTGCCGTCTCAAGAAGTGCTTCCGGGTGGGCATGAGGAAGGAGGCGGTGCAGCGCGGCCGCATCCCGCACTCGCTGCCTGGTGCCGTGGCCGCCTCCTCGGGCAGCCCCCCGGGCTCGGCGCTGGCGGCAGTGGCGAGCGGCGGAGACCTCTTCCCGGGGCAGCCGGTGTCCGAACTGATCGCGCAGCTGCTGCGCGCTGAGCCCTACCCTGCGGCGGCCGGACGCTTCGGCGCAGGGGGCGGCGCGGCGGGCGCGGTGCTGGGCATCGACAACGTGTGCGAGCTGGCGGCGCGGCTGCTCTTCAGCACCGTGGAGTGGGCGCGCCACGCGCCCTTCTTCCCCGAGCTGCCGGTGGCCGACCAGGTGGCGCTGCTGCGCCTGAGCTGGAGCGAGCTCTTCGTGCTGAACGCGGCGCAGGCGGCGCTGCCCCTGCACACGGCGCCGCTACTGGCCGCCGCCGGCCTCCACGCCGCGCCTATGGCCGCCGAGCGCGCCGTGGCTTTCATGGACCAGGTGCGCGCCTTCCAGGAGCAGGTGGACAAGCTGGGCCGCCTGCAGGTCGACTCGGCCGAGTATGGCTGCCTCAAGGCCATCGCGCTCTTCACGCCCGACGCCTGTGGCCTCTCAGACCCGGCCCACGTTGAGAGCCTGCAGGAGAAGGCGCAGGTGGCCCTCACCGAGTATGTGCGGGCGCAGTACCCGTCCCAGCCCCAGCGCTTCGGGCGCCTGCTGCTGCGGCTCCCCGCCCTGCGCGCGGTCCCTGCCTCCCTCATCTCCCAGCTGTTCTTCATGCGCCTGGTGGGGAAGACGCCCATTGAGACACTGATCAGAGACATGCTGCTGTCGGGGAGTACCTTCAACTGGCCCTACGGCTCGGGCCAGTGA

atggccatggtgaccggcggctggggcggccccggcggcgacacgaacggcgtggacaag

M A M V T G G W G G P G G D T N G V D K

gcgggcggctacccgcgcgcggccgaggacgactcggcctcgccccccggtgccgccagc

A G G Y P R A A E D D S A S P P G A A S

gacgccgagccgggcgacgaggagcggccggggctgcaggtggactgcgtggtgtgcggg

D A E P G D E E R P G L Q V D C V V C G

gacaagtcgagcggcaagcattacggtgtcttcacctgcgagggctgcaagagctttttc

D K S S G K H Y G V F T C E G C K S F F

aagcgaagcatccgccgcaacctcagctacacctgccggtccaaccgtgactgccagatc

K R S I R R N L S Y T C R S N R D C Q I

gaccagcaccaccggaaccagtgccagtactgccgtctcaagaagtgcttccgggtgggc

D Q H H R N Q C Q Y C R L K K C F R V G

atgaggaaggaggcggtgcagcgcggccgcatcccgcactcgctgcctggtgccgtggcc

M R K E A V Q R G R I P H S L P G A V A

gcctcctcgggcagccccccgggctcggcgctggcggcagtggcgagcggcggagacctc

A S S G S P P G S A L A A V A S G G D L

ttcccggggcagccggtgtccgaactgatcgcgcagctgctgcgcgctgagccctaccct

F P G Q P V S E L I A Q L L R A E P Y P

gcggcggccggacgcttcggcgcagggggcggcgcggcgggcgcggtgctgggcatcgac

A A A G R F G A G G G A A G A V L G I D

aacgtgtgcgagctggcggcgcggctgctcttcagcaccgtggagtgggcgcgccacgcg

N V C E L A A R L L F S T V E W A R H A

cccttcttccccgagctgccggtggccgaccaggtggcgctgctgcgcctgagctggagc

P F F P E L P V A D Q V A L L R L S W S

gagctcttcgtgctgaacgcggcgcaggcggcgctgcccctgcacacggcgccgctactg

E L F V L N A A Q A A L P L H T A P L L

gccgccgccggcctccacgccgcgcctatggccgccgagcgcgccgtggctttcatggac

A A A G L H A A P M A A E R A V A F M D

caggtgcgcgccttccaggagcaggtggacaagctgggccgcctgcaggtcgactcggcc

Q V R A F Q E Q V D K L G R L Q V D S A

gagtatggctgcctcaaggccatcgcgctcttcacgcccgacgcctgtggcctctcagac

E Y G C L K A I A L F T P D A C G L S D

ccggcccacgttgagagcctgcaggagaaggcgcaggtggccctcaccgagtatgtgcgg

P A H V E S L Q E K A Q V A L T E Y V R

gcgcagtacccgtcccagccccagcgcttcgggcgcctgctgctgcggctccccgccctg

A Q Y P S Q P Q R F G R L L L R L P A L

cgcgcggtccctgcctccctcatctcccagctgttcttcatgcgcctggtggggaagacg

R A V P A S L I S Q L F F M R L V G K T

cccattgagacactgatcagagacatgctgctgtcggggagtaccttcaactggccctac

P I E T L I R D M L L S G S T F N W P Y

ggctcgggccagtga

G S G Q -

***>L. chalumnae Nr2f1***

ATGGCAATGGTAGTTAGTAGCTGGCGAGATCCGCAGGAAGACGTGGCCGGGGGAACTCCAAGCGGCCCGAACCCAGCAGCCCAGTCGGCGAGAGAGCAGCAGCAGACGCAGTCTGCAGCCCCACACACCCCGCAGACTCCCGGCCAGCCAGGACCCCCCTCTACCCCAGGAACTGCTGGCGACAAGGGGCAGAACCAGCAGAATTCAGGCCAGAGCCAACAGCACATCGAGTGTGTGGTTTGCGGGGACAAATCCAGTGGCAAGCACTACGGCCAGTTCACCTGCGAGGGCTGCAAAAGTTTTTTCAAGAGGAGCGTCCGTAGGAACTTAACTTACACATGTCGTGCCAACAGGAACTGTCCCATAGACCAGCACCACCGCAACCAGTGCCAGTACTGTCGCCTCAAGAAGTGTCTCAAAGTGGGCATGAGGCGGGAAGCGGTTCAGCGAGGAAGAATGCCTCCAACCCAACCGAACCCAGGCCAGTATGCTTTGACGAATGGGGACCCCCTGAACGGCCATTGCTATCTCTCTGGATACATCTCGTTACTTCTTCGGGCTGAACCCTACCCGACGTCCCGATATGGAAGCCAGTGCATGCAACCCAACAACATCATGGGCATCGAAAACATCTGCGAGCTGGCGGCCAGATTACTCTTCAGCGCCGTGGAATGGGCCAGGAACATCCCTTTCTTCCCCGACCTGCAGATCACAGACCAGGTGGCTCTCCTGCGGCTGACTTGGAGTGAGTTGTTTGTGCTCAATGCCGCTCAGTGTTCCATGCCCCTCCATGTGGCCCCTCTGCTAGCTGCGGCTGGCCTCCACGCCTCGCCCATGTCTGCGGACCGGGTGGTGGCCTTCATGGATCACATCCGCATCTTCCAGGAGCAGGTGGAGAAGCTCAAGGCCCTGCACGTTGACTCGGCAGAGTATAGCTGCCTCAAAGCCATCGTCCTTTTCACATCAGATGCCTGTGGCCTGTCAGATGTCGCCCATATCGAAAGCCTGCAGGAGAAGTCTCAATGTGCCCTGGAGGAGTACGTTAGGAGTCAGTACCCCAACCAGCCCAGCCGCTTCGGCAAGCTCTTACTGCGGCTGCCTTCTCTGCGCACTGTCTCCTCCTCGGTAATCGAACAGCTCTTCTTCGTCCGCTTGAATCTTCCATCAAGCTTAGAGCAATGGATCAAATCTAAAACTACAGGGTATAAAATATCCCCAGTTGTGAGGGGGGCTTATTTAAGGGAGACCAGACTGACTGCTGTAAGCGATCCCTTTGTGTTGATTTACGAAGACGTTTGCTGTGTATAA

atggcaatggtagttagtagctggcgagatccgcaggaagacgtggccgggggaactcca

M A M V V S S W R D P Q E D V A G G T P

agcggcccgaacccagcagcccagtcggcgagagagcagcagcagacgcagtctgcagcc

S G P N P A A Q S A R E Q Q Q T Q S A A

ccacacaccccgcagactcccggccagccaggacccccctctaccccaggaactgctggc

P H T P Q T P G Q P G P P S T P G T A G

gacaaggggcagaaccagcagaattcaggccagagccaacagcacatcgagtgtgtggtt

D K G Q N Q Q N S G Q S Q Q H I E C V V

tgcggggacaaatccagtggcaagcactacggccagttcacctgcgagggctgcaaaagt

C G D K S S G K H Y G Q F T C E G C K S

tttttcaagaggagcgtccgtaggaacttaacttacacatgtcgtgccaacaggaactgt

F F K R S V R R N L T Y T C R A N R N C

cccatagaccagcaccaccgcaaccagtgccagtactgtcgcctcaagaagtgtctcaaa

P I D Q H H R N Q C Q Y C R L K K C L K

gtgggcatgaggcgggaag**c**ggttcagcgaggaagaatgcctccaacccaaccgaaccca

V G M R R E **A** V Q R G R M P P T Q P N P

ggccagtatgctttgacgaatggggaccccctgaacggccattgctatctctctggatac

G Q Y A L T N G D P L N G H C Y L S G Y

atctcgttacttcttcgggctgaaccctacccgacgtcccgatatggaagccagtgcatg

I S L L L R A E P Y P T S R Y G S Q C M

caacccaacaacatcatgggcatcgaaaacatctgcgagctggcggccagattactcttc

Q P N N I M G I E N I C E L A A R L L F

agcgccgtggaatgggccaggaacatccctttcttccccgacctgcagatcacagaccag

S A V E W A R N I P F F P D L Q I T D Q

gtggctctcctgcggctgacttggagtgagttgtttgtgctcaatgccgctcagtgttcc

V A L L R L T W S E L F V L N A A Q C S

atgcccctccatgtggcccctctgctagctgcggctggcctccacgcctcgcccatgtct

M P L H V A P L L A A A G L H A S P M S

gcggaccgggtggtggccttcatggatcacatccgcatcttccaggagcaggtggagaag

A D R V V A F M D H I R I F Q E Q V E K

ctcaaggccctgcacgttgactcggcagagtatagctgcctcaaagccatcgtccttttc

L K A L H V D S A E Y S C L K A I V L F

acatcag**a**tgcctgtggcctgtcagatgtcgcccatatcgaaagcctgcaggagaagtct

T S **D** A C G L S D V A H I E S L Q E K S

caatgtgccctggaggagtacgttaggagtcagtaccccaaccagcccagccgcttcggc

Q C A L E E Y V R S Q Y P N Q P S R F G

aagctcttactgcggctgccttctctgcgcactgtctcctcctcggtaatcgaacagctc

K L L L R L P S L R T V S S S V I E Q L

ttcttcgtccgcttg**aat**cttccatcaagcttagagcaatggatcaaatctaaaactaca

F F V R L **N** L P S S L E Q W I K S K T T

gggtataaaatatccccagttgtgaggggggcttatttaagggagaccagactgactgct

G Y K I S P V V R G A Y L R E T R L T A

gtaagcgatccctttgtgttgatttacgaagacgtttgctgtgtataa

V S D P F V L I Y E D V C C V -

***>L. chalumnae Nr2f2***

ATGGCAATGGTAGTTAGTGCGTGGCGAGACCCCCAGGACGACGTGGCCGGAGCTCAGGGAACCCAGCCTTCCCAAGCCCCTCCGGGGCAAGGACCACCAACTGGGGCCCCTCATACCCCTCAGACCCCTGTGCAAGTGGGGCCTCCTACTACTCCAGCCCAATCCAACCAGACAAACCAGCCCAACCAGCAGAATCAAGTGGAAAAACAGCAACAACATATTGAGTGTGTGGTTTGTGGGGACAAGTCTAGTGGCAAACACTATGGCCAATTCACCTGTGAGGGTTGCAAGAGCTTCTTCAAGAGAAGTGTTAGAAGGAACTTGAGTTACACATGTCGTGCCAACAGGAACTGTCCCATAGACCAGCACCACCGCAATCAGTGTCAGTACTGTCGCCTCAAAAAATGTCTCAAAGTTGGCATGAGACGGGAAGTTTCTTCTCTATTTACTGCAGCCGTCCAGAGGGGCAGAATGCCACCCACACAGCCAACTCACGGTCAGTTCGCCTTGACAAATGGGGACCCTCTCAACTGCCATTCCTATCTATCCGGATATATCTCCCTTCTTCTCAGAGCTGAGCCCTACCCAACCTCCCGCTTTGGTAGCCAGTGCATGCAACCGAACAACATCATGGGCATTGAAAACATTTGTGAACTAGCAGCTAGGATGCTCTTCAGTGCGGTGGAGTGGGCAAGGAATATCCCCTTCTTTCCAGACCTCCAGATCACAGACCAGGTGGCCCTTCTCAGACTGACCTGGAGCGAATTATTTGTGCTTAACGCTGCCCAGTGCTCCATGCCTCTCCATGTGGCACCGCTCCTGGCTGCTGCTGGCCTCCATGCCTCTCCTATGTCTGCAGACAGAGTGGTTGCATTCATGGATCACATAAGGATCTTCCAAGAACAAGTGGAGAAGTTGAAGGCACTGCATGTCGACTCTGCTGAATACAGCTGTTTGAAGGCCATAGTTCTTTTCACCTCAGATGCCTGTGGTCTCTCTGATGTAGCCCACGTGGAAAGCTTACAGGAGAAGTCTCAATGTGCTTTGGAAGAGTATGTCAGGAGCCAGTATCCCAATCAGCCAACTCGCTTTGGGAAGCTTTTACTTCGCCTCCCCTCCCTTCGCACTGTCTCCTCTTCTGTTATAGAGCAATTGTTTTTCGTCCGTTTGGTAGGTAAAACCCCAATTGAAACCCTCATCAGGGACATGTTACTGTCTGGGAGCAGTTTTAACTGGCCTTATATGTCCATTCAATAA

atggcaatggtagttagtgcgtggcgagacccccaggacgacgtggccggagctcaggga

M A M V V S A W R D P Q D D V A G A Q G

acccagccttcccaagcccctccggggcaaggaccaccaactggggcccctcatacccct

T Q P S Q A P P G Q G P P T G A P H T P

cagacccctgtgcaagtggggcctcctactactccagcccaatccaaccagacaaaccag

Q T P V Q V G P P T T P A Q S N Q T N Q

cccaaccagcagaatcaagtggaaaaacagcaacaacatattgagtgtgtggtttgtggg

P N Q Q N Q V E K Q Q Q H I E C V V C G

gacaagtctagtggcaaacactatggccaattcacctgtgagggttgcaagagcttcttc

D K S S G K H Y G Q F T C E G C K S F F

aagagaagtgttagaaggaacttgagttacacatgtcgtgccaacaggaactgtcccata

K R S V R R N L S Y T C R A N R N C P I

gaccagcaccaccgcaatcagtgtcagtactgtcgcctcaaaaaatgtctcaaagttggc

D Q H H R N Q C Q Y C R L K K C L K V G

atgagacgggaag**t**ttcttctctatttactgcagccgtccagaggggcagaatgccaccc

M R R E **V** S S L F T A A V Q R G R M P P

acacagccaactcacggtcagttcgccttgacaaatggggaccctctcaactgccattcc

T Q P T H G Q F A L T N G D P L N C H S

tatctatccggatatatctcccttcttctcagagctgagccctacccaacctcccgcttt

Y L S G Y I S L L L R A E P Y P T S R F

ggtagccagtgcatgcaaccgaacaacatcatgggcattgaaaacatttgtgaactagca

G S Q C M Q P N N I M G I E N I C E L A

gctaggatgctcttcagtgcggtggagtgggcaaggaatatccccttctttccagacctc

A R M L F S A V E W A R N I P F F P D L

cagatcacagaccaggtggcccttctcagactgacctggagcgaattatttgtgcttaac

Q I T D Q V A L L R L T W S E L F V L N

gctgcccagtgctccatgcctctccatgtggcaccgctcctggctgctgctggcctccat

A A Q C S M P L H V A P L L A A A G L H

gcctctcctatgtctgcagacagagtggttgcattcatggatcacataaggatcttccaa

A S P M S A D R V V A F M D H I R I F Q

gaacaagtggagaagttgaaggcactgcatgtcgactctgctgaatacagctgtttgaag

E Q V E K L K A L H V D S A E Y S C L K

gccatagttcttttcacctcag**a**tgcctgtggtctctctgatgtagcccacgtggaaagc

A I V L F T S **D** A C G L S D V A H V E S

ttacaggagaagtctcaatgtgctttggaagagtatgtcaggagccagtatcccaatcag

L Q E K S Q C A L E E Y V R S Q Y P N Q

ccaactcgctttgggaagcttttacttcgcctcccctcccttcgcactgtctcctcttct

P T R F G K L L L R L P S L R T V S S S

gttatagagcaattgtttttcgtccgtttggtaggtaaaaccccaattgaaaccctcatc

V I E Q L F F V R L V G K T P I E T L I

agggacatgttactgtctgggagcagttttaactggccttatatgtccattcaataa

R D M L L S G S S F N W P Y M S I Q -

***>L. chalumnae Nr2f5***

atggcaatggtagtaaatgtatggcaagaggacatccccggggcatcggggtcccaagcgaggagccagccgcagatgtgcactcaggaagcgggcgggaccccgcagacccccggcacgccggcgggctccacgccggggcaggaggcgctgtccggggacagggcgcccgccgtggactgcatggtgtgcggcgacaagtccagcggcaaacactacggccagttcacctgcgagggctgcaagagcttcttcaagcgctcggtgcgcaggaacctgagctacacctgccggggcaaccgggactgtccgatcgaccagcaccaccgtaaccagtgccagtactgccgcctgaaaaagtgcctcaaggtcggcatgaggagggaagctgtgcagcgtggacggatgacccatccccagaccagtccaggccaatacacactgaccaacagcgaccagtacaatggccattcctacctgacaggcttcatatccatgctgcttcgagcagagccctaccccatgtctcgctacggtgggcagtgcatgcaacccaacaacttcatgggcatcgaaaacatctgtgagctggctgcccgcctccttttcagcgccatcgagtgggccaagagcatccctttcttccctgacctgcagctgggcgaccaggtctccctgctccgcatgacgtggagcgagctctttgtcctcaatgccgcccagtgctccatgccgctgcacgtggccccgctattggcggctgccgggctccatgcctcccccatgtcagcagaccgtgtggtggcgttcatggatcacatccgggtcttccaagagcaagtggaaaagctcaaggcattacatgtcgattctgcagagtattcttgcctgaaagccatagtccttttcacaccagatgccgtgggagtgtccgacttggcccacgtggagagcatccaggagaaatcacaatgtgccctggaagagtacgtgcggaaccagtaccccaaccagcctagccgcttcgggaggctgctcctacgccttccctccctccgcatcgtctcctcccccatcatcgagcaactcttctttgtccgcctggtgggcaagacccccatcgagacgctcatcagggacatgctgctgtcagggtccagcctcaactggccatacatggccatgcagtga

atggcaatggtagtaaatgtatggcaagaggacatccccggggcatcggggtcccaagcg

M A M V V N V W Q E D I P G A S G S Q A

aggagccagccgcagatgtgcactcaggaagcgggcgggaccccgcagacccccggcacg

R S Q P Q M C T Q E A G G T P Q T P G T

ccggcgggctccacgccggggcaggaggcgctgtccggggacagggcgcccgccgtggac

P A G S T P G Q E A L S G D R A P A V D

tgcatggtgtgcggcgacaagtccagcggcaaacactacggccagttcacctgcgagggc

C M V C G D K S S G K H Y G Q F T C E G

tgcaagagcttcttcaagcgctcggtgcgcaggaacctgagctacacctgccggggcaac

C K S F F K R S V R R N L S Y T C R G N

cgggactgtccgatcgaccagcaccaccgtaaccagtgccagtactgccgcctgaaaaag

R D C P I D Q H H R N Q C Q Y C R L K K

tgcctcaaggtcggcatgaggagggaagctgtgcagcgtggacggatgacccatccccag

C L K V G M R R E A V Q R G R M T H P Q

accagtccaggccaatacacactgaccaacagcgaccagtacaatggccattcctacctg

T S P G Q Y T L T N S D Q Y N G H S Y L

acaggcttcatatccatgctgcttcgagcagagccctaccccatgtctcgctacggtggg

T G F I S M L L R A E P Y P M S R Y G G

cagtgcatgcaacccaacaacttcatgggcatcgaaaacatctgtgagctggctgcccgc

Q C M Q P N N F M G I E N I C E L A A R

ctccttttcagcgccatcgagtgggccaagagcatccctttcttccctgacctgcagctg

L L F S A I E W A K S I P F F P D L Q L

ggcgaccaggtctccctgctccgcatgacgtggagcgagctctttgtcctcaatgccgcc

G D Q V S L L R M T W S E L F V L N A A

cagtgctccatgccgctgcacgtggccccgctattggcggctgccgggctccatgcctcc

Q C S M P L H V A P L L A A A G L H A S

cccatgtcagcagaccgtgtggtggcgttcatggatcacatccgggtcttccaagagcaa

P M S A D R V V A F M D H I R V F Q E Q

gtggaaaagctcaaggcattacatgtcgattctgcagagtattcttgcctgaaagccata

V E K L K A L H V D S A E Y S C L K A I

gtccttttcacaccagatgccgtgggagtgtccgacttggcccacgtggagagcatccag

V L F T P D A V G V S D L A H V E S I Q

gagaaatcacaatgtgccctggaagagtacgtgcggaaccagtaccccaaccagcctagc

E K S Q C A L E E Y V R N Q Y P N Q P S

cgcttcgggaggctgctcctacgccttccctccctccgcatcgtctcctcccccatcatc

R F G R L L L R L P S L R I V S S P I I

gagcaactcttctttgtccgcctggtgggcaagacccccatcgagacgctcatcagggac

E Q L F F V R L V G K T P I E T L I R D

atgctgctgtcagggtccagcctcaactggccatacatggccatgcagtga

M L L S G S S L N W P Y M A M Q -

***>L. chalumnae Nr2f6***

ATGGCCATGGTGACTGGAGGTTGGGGAGACCCTAATGGAGAGACCAATGGGGTTATAAAGGGCTACCCCAGAAAGTCAGAGGAGGAGGAGGAGGCATCTCCACAGGGAGGGGGCAGTGACCAAGAGCATGGCGAGGAAGATAAACCTGGGATCCAGGTGGACTGTGTGGTCTGTGGGGACAAATCTAGTGGGAAGCACTATGGAGCTTTTACTTGTGAAGGATGCAAGAGCTTTTTCAAAAGAAGCATAAGGAGAAATCTGAACTACACATGCAGGTCCAACCGGGACTGCCAGATTGATCAGCACCATCGGAACCAGTGCCAATACTGCCGCCTGAAAAAATGCTTCCGAGTGGGCATGAGGAAAGAAGCTGTTCAACGTGGTAGGATTCCCTTAGCCCAGTCAACCACCAGTCCTAACTCAACGCCTGGAGGGGATTATTTCAACGGGCAACCTGTTTCTGAACTTATCTTCCAGCTTCTACGAGCAGAACCTTACCCCACCGCCCGCTATGGGTCCCAGTACACCCAACAAAACACTGTTATGGGTATTGACAACATCTGCGAGCTGGCTGCCAGACTCCTTTTCAGCACTGTTGAGTGGGCTAGGAATATTCCATTTTTCCCAGAGTTGCCAGTTTCAGACCAGATATCCTTGCTGCGGCTAAGCTGGAGTGAGTTGTTTGTCTTGAATGCAGCGCAGTCGGCTCTTCCTCTACACATGGCCCCACTGCTGGCAGCGGCTGGGTTTCACACTTCTCCGATGTCTGCGGACCGAGTTGTGTCATTCATGGATCAGATTAGGATCTTTCAGGATCAGGTGGAGAAACTAAACCGGCTGCAGGTGGATTCCGCTGAATACAGCTGCTTGAAGGCTATAGCTCTCTTCACACCAGATGCCTGTGGTCTCTCGGATCCTGCCCATGTGGAGAGTTTGCAGGAAAAGGCTCAGGTGGCCCTAACAGAGTACGTCCGCTCTCAGTATCCTTCCCAACCTCAGCGATTTGGCAGGCTGCTGCTGCGGCTGCCAGCACTTCGGGCAGTGCCTGCATCACTTATCTCACAGTTGTTCTTCATGAGACTGGTGGGGAAGACTCCGATTGAAACTCTAATACGGGATATGTTACTATCAGGCAGTACATTCAATTGGCCCTATGTAGGAGGTCAACAATAA

atggccatggtgactggaggttggggagaccctaatggagagaccaatggggttataaag

M A M V T G G W G D P N G E T N G V I K

ggctaccccagaaagtcagaggaggaggaggaggcatctccacagggagggggcagtgac

G Y P R K S E E E E E A S P Q G G G S D

caagagcatggcgaggaagataaacctgggatccaggtggactgtgtggtctgtggggac

Q E H G E E D K P G I Q V D C V V C G D

aaatctagtgggaagcactatggagcttttacttgtgaaggatgcaagagctttttcaaa

K S S G K H Y G A F T C E G C K S F F K

agaagcataaggagaaatctgaactacacatgcag**g**tccaaccgggactgccagattgat

R S I R R N L N Y T C **R**  S N R D C Q I D

cagcaccatcggaaccagtgccaatactgccgcctgaaaaaatgcttccgagtgggcatg

Q H H R N Q C Q Y C R L K K C F R V G M

aggaaagaag**c**tgttcaacgtggtaggattcccttagcccagtcaaccaccagtcctaac

R K E **A** V Q R G R I P L A Q S T T S P N

tcaacgcctggaggggattatttcaacgggcaacctgtttctgaacttatcttccagctt

S T P G G D Y F N G Q P V S E L I F Q L

ctacgagcagaaccttaccccaccgcccgctatgggtcccagtacacccaacaaaacact

L R A E P Y P T A R Y G S Q Y T Q Q N T

gttatgggtattgacaacatctgcgagctggctgccagactccttttcagcactgttgag

V M G I D N I C E L A A R L L F S T V E

tgggctaggaatattccatttttcccagagttgccagtttcagaccagatatccttgctg

W A R N I P F F P E L P V S D Q I S L L

cggctaagctggagtgagttgtttgtcttgaatgcagcgcagtcggctcttcctctacac

R L S W S E L F V L N A A Q S A L P L H

atggccccactgctggcagcggctgggtttcacacttctccgatgtctgcggaccgagtt

M A P L L A A A G F H T S P M S A D R V

gtgtcattcatggatcagattaggatctttcaggatcaggtggagaaactaaaccggctg

V S F M D Q I R I F Q D Q V E K L N R L

caggtggattccgctgaatacagctgcttgaaggctatagctctcttcacaccag**a**tgcc

Q V D S A E Y S C L K A I A L F T P **D** A

tgtggtctctcggatcctgcccatgtggagagtttgcaggaaaaggctcaggtggcccta

C G L S D P A H V E S L Q E K A Q V A L

acagagtacgtccgctctcagtatccttcccaacctcagcgatttggcaggctgctgctg

T E Y V R S Q Y P S Q P Q R F G R L L L

cggctgccagcacttcgggcagtgcctgcatcacttatctcacagttgttcttcatgaga

R L P A L R A V P A S L I S Q L F F M R

ctggtggggaagactccgattgaaactctaatacgggatatgttactatcaggcagtaca

L V G K T P I E T L I R D M L L S G S T

ttcaattggccctatgtaggaggtcaacaataa

F N W P Y V G G Q Q -

***>X. tropicalis Nr2f1***

ATGGCAATGGTAGTTAGCAGCTGGAGAGATCCGCAGGAGGACGTGGCCGGGGGAAATCCGGGAGGCCCCAACCCGGGGGCCAGGGAGCAACAGCAAGCGCCCTCCGCTGCCCCCCACACGCCTCAGACCCCCAGCCAGCCGGGACCCCCATCCACCCCCGGTGCAGCCGGGGACAAGGGCCAGCAGGGCTCCGGGCAGAGTCAGCAGCAGCACATCGAGTGCGTCGTGTGTGGGGACAAGTCGAGCGGCAAGCACTACGGCCAGTTCACCTGCGAGGGCTGCAAAAGTTTCTTCAAGAGGAGCGTCCGCAGGAACTTAACGTACACATGTCGCGCCAACCGCAACTGCCCCATAGACCAGCACCACCGCAACCAGTGCCAGTACTGCCGCCTGAAGAAGTGCCTCAAAGTGGGCATGAGGAGGGAAGTACAGCGAGGTAGGATGCCTCCGACCCAGCCGAACCCAGGGCAGTACGCTCTCACCAACGGGGACCCCTTGAATGGCCACTGCTACCTGTCCGGATACATCTCGCTGCTGCTGCGGGCCGAACCGTACCCCACGTCCCGCTATGGCAGCCAGTGCATGCAGCCCAACAACATTATGGGCATTGAGAACATCTGCGAGCTGGCGGCCCGGTTACTGTTCAGCGCCGTGGAGTGGGCCAGGAACATCCCTTTCTTCCCGGACCTGCAGATTACTGACCAGGTGGCCCTGCTGCGGCTGACCTGGAGTGAGCTGTTTGTGCTGAATGCGGCTCAGTGCTCCATGCCCCTCCATGTGGCCCCTCTCCTAGCCGCTGCTGGCCTCCATGCTTCCCCCATGTCCGCCGACAGAGTGGTGGCCTTTATGGACCACATCCGGATCTTCCAAGAGCAGGTGGAGAAGCTTAAGGCCTTGCATGTAGACTCTGCCGAGTATAGCTGCCTCAAAGCCATCGTCCTCTTCACATCAGATGCCTGCGGCCTGTCAGACGCTGCGCACATTGAGAGCCTGCAGGAAAAGTCGCAGTGCGCCCTGGAGGAGTATGTTAGGAGCCAATACCCCAACCAGCCCAGCAGGTTCGGCAAACTACTTCTAAGGCTCCCATCCTTGCGTACAGTCTCTTCGTCTGTCATCGAGCAACTCTTCTTCGTTCGTTTGGTAGGTAAAACCCCAATAGAGACTCTCATTAGAGACATGTTATTATCTGGAAGTAGTTTCAATTGGCCTTACATGCCCATCCAGTGCTCCTAG

atggcaatggtagttagcagctggagagatccgcaggaggacgtggccgggggaaatccg

M A M V V S S W R D P Q E D V A G G N P

ggaggccccaacccgggggccagggagcaacagcaagcgccctccgctgccccccacacg

G G P N P G A R E Q Q Q A P S A A P H T

cctcagacccccagccagccgggacccccatccacccccggtgcagccggggacaagggc

P Q T P S Q P G P P S T P G A A G D K G

cagcagggctccgggcagagtcagcagcagcacatcgagtgcgtcgtgtgtggggacaag

Q Q G S G Q S Q Q Q H I E C V V C G D K

tcgagcggcaagcactacggccagttcacctgcgagggctgcaaaagtttcttcaagagg

S S G K H Y G Q F T C E G C K S F F K R

agcgtccgcaggaacttaacgtacacatgtcgcgccaaccgcaactgccccatagaccag

S V R R N L T Y T C R A N R N C P I D Q

caccaccgcaaccagtgccagtactgccgcctgaagaagtgcctcaaagtgggcatgagg

H H R N Q C Q Y C R L K K C L K V G M R

agggaagtacagcgaggtaggatgcctccgacccagccgaacccagggcagtacgctctc

R E V Q R G R M P P T Q P N P G Q Y A L

accaacggggaccccttgaatggccactgctacctgtccggatacatctcgctgctgctg

T N G D P L N G H C Y L S G Y I S L L L

cgggccgaaccgtaccccacgtcccgctatggcagccagtgcatgcagcccaacaacatt

R A E P Y P T S R Y G S Q C M Q P N N I

atgggcattgagaacatctgcgagctggcggcccggttactgttcagcgccgtggagtgg

M G I E N I C E L A A R L L F S A V E W

gccaggaacatccctttcttcccggacctgcagattactgaccaggtggccctgctgcgg

A R N I P F F P D L Q I T D Q V A L L R

ctgacctggagtgagctgtttgtgctgaatgcggctcagtgctccatgcccctccatgtg

L T W S E L F V L N A A Q C S M P L H V

gcccctctcctagccgctgctggcctccatgcttcccccatgtccgccgacagagtggtg

A P L L A A A G L H A S P M S A D R V V

gcctttatggaccacatccggatcttccaagagcaggtggagaagcttaaggccttgcat

A F M D H I R I F Q E Q V E K L K A L H

gtagactctgccgagtatagctgcctcaaagccatcgtcctcttcacatcagatgcctgc

V D S A E Y S C L K A I V L F T S D A C

ggcctgtcagacgctgcgcacattgagagcctgcaggaaaagtcgcagtgcgccctggag

G L S D A A H I E S L Q E K S Q C A L E

gagtatgttaggagccaataccccaaccagcccagcaggttcggcaaactacttctaagg

E Y V R S Q Y P N Q P S R F G K L L L R

ctcccatccttgcgtacagtctcttcgtctgtcatcgagcaactcttcttcgttcgtttg

L P S L R T V S S S V I E Q L F F V R L

gtaggtaaaaccccaatagagactctcattagagacatgttattatctggaagtagtttc

V G K T P I E T L I R D M L L S G S S F

aattggccttacatgcccatccagtgctcctag

N W P Y M P I Q C S -

***>X. tropicalis Nr2f2***

ATGGCAATGGTAGTGGGTGCGTGGCGAGACCCTCAGGACGATATGCCAGGAACTCAACCTTCACAAGCACCTCCAGGGCAAGGGCCAAACGGGGCCCCGCACACCCCGCAGACCCCGGGCCAAGGGGTCCCCTCTACTACCCCAGCCCAGTCCAACCCATCCAGCCAACCGAGCCAGAACCAAGGGGAGAAGCAGCAGCAGCAGCAGCACATCGAGTGCGTGGTGTGCGGGGACAAGTCCAGCGGGAAACATTACGGACAGTTCACATGCGAGGGCTGCAAGAGCTTCTTCAAGCGAAGTGTAAGGCGAAATCTCACTTACACGTGTCGTGCCAACAGGAACTGTCCCATAGACCAACACCACCGCAATCAGTGTCAGTACTGTCGCCTCAAAAAATGCCTCAAAGTTGGCATGAGACGGGAAGCCGTACAGAGGGGCAGAATGCCACCCACACAGCCTACACACGGGCAGTTCGCCTTGACAAATGGAGACCCTCTCAACTGCCATTCCTACCTATCCGGATATATATCGTTACTCCTGAGGGCAGAACCCTACCCGACCTCTAGATTTGGCAGCCAGTGCATGCAACCCAACAATATCATGGGCATCGAGAACATTTGTGAGCTGGCAGCCAGGATGCTGTTCAGTGCTGTGGAGTGGGCAAGGAATATCCCCTTCTTCCCAGACCTGCAGATCACAGACCAGGTGGCATTGCTCAGGCTGACCTGGAGTGAGTTGTTTGTCCTCAACGCTGCCCAGTGTTCCATGCCCCTCCATGTGGCCCCTTTACTGGCCGCTGCTGGCCTGCACGCTTCCCCCATGTCTGCGGACAGAGTGGTGGCATTTATGGATCACATACGAATCTTCCAAGAACAGGTCGAAAAACTGAAGGCTTTGCACGTTGACTCTGCAGAATACAGCTGCCTCAAGGCCATAGTACTCTTCACTTCAgATGCCTGTGGTCTGTCTGATGTGGCACATGTGGAAAGTTTGCAAGAAAAGTCCCAATGTGCTTTGGAAGAATATGTTAGAAGCCAGTACCCAAACCAACCAACAAGATTTGGGAAATTGTTACTTCGACTGCCCTCCCTCCGCACTGTCTCCTCCTCTGTCATAGAGCAGTTGTTTTTCGTCCGGTTGGTAGGTAAAACCCCTATAGAGACCCTAATTAGGGATATGTTACTCTCTGGAAGCAGTTTCAATTGGCCCTATATGTCCATACAATAA

atggcaatggtagtgggtgcgtggcgagaccctcaggacgatatgccaggaactcaacct

M A M V V G A W R D P Q D D M P G T Q P

tcacaagcacctccagggcaagggccaaacggggccccgcacaccccgcagaccccgggc

S Q A P P G Q G P N G A P H T P Q T P G

caaggggtcccctctactaccccagcccagtccaacccatccagccaaccgagccagaac

Q G V P S T T P A Q S N P S S Q P S Q N

caaggggagaagcagcagcagcagcagcacatcgagtgcgtggtgtgcggggacaagtcc

Q G E K Q Q Q Q Q H I E C V V C G D K S

agcgggaaacattacggacagttcacatgcgagggctgcaagagcttcttcaagcgaagt

S G K H Y G Q F T C E G C K S F F K R S

gtaaggcgaaatctcacttacacgtgtcgtgccaacaggaactgtcccatagaccaacac

V R R N L T Y T C R A N R N C P I D Q H

caccgcaatcagtgtcagtactgtcgcctcaaaaaatgcctcaaagttggcatgagacgg

H R N Q C Q Y C R L K K C L K V G M R R

gaag**c**cgtacagaggggcagaatgccacccacacagcctacacacgggcagttcgccttg

E **A** V Q R G R M P P T Q P T H G Q F A L

acaaatggagaccctctcaactgccattcctacctatccggatatatatcgttactcctg

T N G D P L N C H S Y L S G Y I S L L L

agggcagaaccctacccgacctctagatttggcagccagtgcatgcaacccaacaatatc

R A E P Y P T S R F G S Q C M Q P N N I

atgggcatcgagaacatttgtgagctggcagccaggatgctgttcagtgctgtggagtgg

M G I E N I C E L A A R M L F S A V E W

gcaaggaatatccccttcttcccagacctgcagatcacagaccaggtggcattgctcagg

A R N I P F F P D L Q I T D Q V A L L R

ctgacctggagtgagttgtttgtcctcaacgctgcccagtgttccatgcccctccatgtg

L T W S E L F V L N A A Q C S M P L H V

gcccctttactggccgctgctggcctgcacgcttcccccatgtctgcggacagagtggtg

A P L L A A A G L H A S P M S A D R V V

gcatttatggatcacatacgaatcttccaagaacaggtcgaaaaactgaaggctttgcac

A F M D H I R I F Q E Q V E K L K A L H

gttgactctgcagaatacagctgcctcaaggccatagtactcttcacttcag**a**tgcctgt

V D S A E Y S C L K A I V L F T S **D** A C

ggtctgtctgatgtggcacatgtggaaagtttgcaagaaaagtcccaatgtgctttggaa

G L S D V A H V E S L Q E K S Q C A L E

gaatatgttagaagccagtacccaaaccaaccaacaagatttgggaaattgttacttcga

E Y V R S Q Y P N Q P T R F G K L L L R

ctgccctccctccgcactgtctcctcctctgtcatagagcagttgtttttcgtccggttg

L P S L R T V S S S V I E Q L F F V R L

gtaggtaaaacccctatagagaccctaattagggatatgttactctctggaagcagtttc

V G K T P I E T L I R D M L L S G S S F

aattggccctatatgtccatacaataa

N W P Y M S I Q -

***>X. tropicalis Nr2f5***

ATGGCCATGGTGGTTAACCCTTGGCAGGAGGACATTCCTGGTGTGCCAGGGTCTCAGGTGAACAACCCACCAGGGCTCTGCAATCAAGATCCAGGGGGTACCCCTCAAACGCCCACCACCCCGAAAGGAGGTGTCCCTGGTCAGGATCCTGTTCATTCTGGGGATAAAGGCGTACCGAATGTGGACTGCTTGGTGTGTGGGGACAAGTCAAGTGGGAAACACTATGGACAGTTCACCTGTGAAGGGTGCAAGAGTTTCTTTAAGAGGTCAGTGAGGAGGAACCTGACCTACACGTGTAGGGGCAACAGAGACTGTCCTATAGATCAGCATCACCGTAATCAGTGCCAGTACTGCCGCCTGAAGAAGTGTCTCAAAGTTGGCATGAGAAGGGAAGCAGTCCAGCGCGGTCGGATGTCTCACCCACAAACCAGCCCAGGCCAGTATACTCTGAACAACGTTGACCCTTACAATGGGCACTCGTACCTAACGGGATTCATCTCCTTGCTTCTCCGAGCTGAGCCATACCCGACCTCACGGTATGGAGCCCAGTGCCTACAGCCGAACAACATCATGGGCATCGAAAACATCTGCGAGTTGGCGGCCCGCCTACTCTTCAGCGCTATTGAGTGGGCCAAGAACATTCCTTTCTTTCCTGACTTCCAGCTCTCTGACCAAGTGTCCCTCCTCCGTATGACGTGGAGTGAGCTGTTTGTTCTTAATGCAGCACAGTGCTCCATGCCCCTCCATGTGGCGCCCCTGTTGGCAGCCGCTGGCCTCCATGCATCGCCAATGTCTGCCGACCGTGTGGTGGCCTTTATGGACCATATTCGAGTCTTTCAGGAGCAGGTGGAAAAGCTGAAGGCACTTCACGTTGACTCTGCAGAATACTCCTGTTTGAAAGCCATAGCCTTGTTTACACCTGATGCAGTGGGACTATCAGACATTGGCCACGTGGAAAGCATTCAAGAGAAATCCCAGTGTGCCCTGGAAGAGTACGTCCGAAACCAGTACCCAAACCAGCCAACGCGGTTTGGGAGGCTTTTGCTCCGCCTTCCTTCTTTGCGCATCGTCTCCGCTCCTGTCATAGAGCAACTTTTCTTTGTGCGCTTGGTTGGCAAGACCCCCATTGAGACTTTAATCCGAGATATGCTCCTGTCTGGGTCCAGCTTTAATTGGCCCTATATGCCTATGCAGTGA

atggccatggtggttaacccttggcaggaggacattcctggtgtgccagggtctcaggtg

M A M V V N P W Q E D I P G V P G S Q V

aacaacccaccagggctctgcaatcaagatccagggggtacccctcaaacgcccaccacc

N N P P G L C N Q D P G G T P Q T P T T

ccgaaaggaggtgtccctggtcaggatcctgttcattctggggataaaggcgtaccgaat

P K G G V P G Q D P V H S G D K G V P N

gtggactgcttggtgtgtggggacaagtcaagtgggaaacactatggacagttcacctgt

V D C L V C G D K S S G K H Y G Q F T C

gaagggtgcaagagtttctttaagaggtcagtgaggaggaacctgacctacacgtgtagg

E G C K S F F K R S V R R N L T Y T C R

ggcaacagagactgtcctatagatcagcatcaccgtaatcagtgccagtactgccgcctg

G N R D C P I D Q H H R N Q C Q Y C R L

aagaagtgtctcaaagttggcatgagaagggaa**gca**gtccagcgcggtcggatgtctcac

K K C L K V G M R R E **A** V Q R G R M S H

ccacaaaccagcccaggccagtatactctgaacaacgttgacccttacaatgggcactcg

P Q T S P G Q Y T L N N V D P Y N G H S

tacctaacgggattcatctccttgcttctccgagctgagccatacccgacctcacggtat

Y L T G F I S L L L R A E P Y P T S R Y

ggagcccagtgcctacagccgaacaacatcatgggcatcgaaaacatctgcgagttggcg

G A Q C L Q P N N I M G I E N I C E L A

gcccgcctactcttcagcgctattgagtgggccaagaacattcctttctttcctgacttc

A R L L F S A I E W A K N I P F F P D F

cagctctctgaccaagtgtccctcctccgtatgacgtggagtgagctgtttgttcttaat

Q L S D Q V S L L R M T W S E L F V L N

gcagcacagtgctccatgcccctccatgtggcgcccctgttggcagccgctggcctccat

A A Q C S M P L H V A P L L A A A G L H

gcatcgccaatgtctgccgaccgtgtggtggcctttatggaccatattcgagtctttcag

A S P M S A D R V V A F M D H I R V F Q

gagcaggtggaaaagctgaaggcacttcacgttgactctgcagaatactcctgtttgaaa

E Q V E K L K A L H V D S A E Y S C L K

gccatagccttgtttacacctg**a**tgcagtgggactatcagacattggccacgtggaaagc

A I A L F T P  **D** A V G L S D I G H V E S

attcaagagaaatcccagtgtgccctggaagagtacgtccgaaaccagtacccaaaccag

I Q E K S Q C A L E E Y V R N Q Y P N Q

ccaacgcggtttgggaggcttttgctccgccttccttctttgcgcatcgtctccgctcct

P T R F G R L L L R L P S L R I V S A P

gtcatagagcaacttttctttgtgcgcttggttggcaagacccccattgagactttaatc

V I E Q L F F V R L V G K T P I E T L I

cgagatatgctcctgtctgggtccagctttaattggccctatatgcctatgcagtga

R D M L L S G S S F N W P Y M P M Q -

***>X. tropicalis Nr2f6***

ATGGCCATGGTGTCTGGGGGCTGGGGAGACCCCAACGGAGACACTAATGGTGTGGGGAAGGGATACCCCAGGAATTCTGAGGAAGAAGAGGCCTCGCCTCAGGGAGGAATGAGTGACCCCGAGCAGGGTGATGAAGAACGCCCAGGGATCCAAGTGGACTGTGTGGTGTGTGGAGATAAATCTAGCGGAAAGCATTATGGGGTTTTCACCTGTGAGGGATGCAAAAGTTTCTTTAAAAGGAGTGTCCGCAGGAATCTCAGTTACACTTGTAGGTCAAACCGAGATTGTCAGATTGATCAGCATCATCGAAACCAGTGTCAGTACTGCCGACTGAAGAAGTGCTTCCGAGTTGGCATGCGGAAAGAAGCTGTCCAGCGAGGTAGAATCCCACCTGCCCACTCTAGTGCCAGTCCCACTTCTGCTCCGGGAGCAGGCGAGTACTTCAATGGACAGCCCGTCTCTGAACTGATCTCCCAGCTCCTGAGAGCTGAGCCATATCCAGCTTCCCGCTATGGGTCCCAGTACGCTCAACAGGGTAGTGTGATGGGCATTGATAATATCTGTGAGTTGGCGGCACGGCTTCTCTTTAGCACTGTTGAATGGTCAAGGAACATCCCCTATTTCCCAGAACTTGCTATGGCTGATCAGGTCTCATTGCTTCGACTGAGCTGGAGTGAACTCTTTGTACTTAGTGCTGCTCAGTCTGCCCTTCCACTCCACATGGCTCCCCTTTTGGCTGCTGCTGGATTCCATGCTTCTCCAATGTCTGCTGACCGAGTCGTCTCATTTATGGATCAGATTCGTCTCTTCCAGGACCAGGTTGAGAAGCTTAACCGCCTGCAGGTGGACTCGGCTGAATATGCTTGTTTAAAAGCTATTGCACTCTTCACTTCAGATGCCTGTGGCCTCACAGATCCAGCTCACGTGGAAAGCTTACAAGAAAAAGCCCAGGTAGCCCTCACAGAATATGTCCGAGCCCAGTATCCCTCTCAGCCCCAGCGCTTTGGGCGTCTCCTCTTACGACTGCCTGCACTTCGAGCTGTTCCTGCATCACTCATATCCCAGCTCTTCTTCATGAGGCTTGTCGGCAAGACCCCTATAGAAACACTTATTAGGGACATGCTTCTGTCTGGGAGTAGTTTTAATTGGCCATACTCATCTGGTCAATAG

atggccatggtgtctgggggctggggagaccccaacggagacactaatggtgtggggaag

M A M V S G G W G D P N G D T N G V G K

ggataccccaggaattctgaggaagaagaggcctcgcctcagggaggaatgagtgacccc

G Y P R N S E E E E A S P Q G G M S D P

gagcagggtgatgaagaacgcccagggatccaagtggactgtgtggtgtgtggagataaa

E Q G D E E R P G I Q V D C V V C G D K

tctagcggaaagcattatggggttttcacctgtgagggatgcaaaagtttctttaaaagg

S S G K H Y G V F T C E G C K S F F K R

agtgtccgcaggaatctcagttacacttgtag**g**tcaaaccgagattgtcagattgatcag

S V R R N L S Y T C **R** S N R D C Q I D Q

catcatcgaaaccagtgtcagtactgccgactgaagaagtgcttccgagttggcatgcgg

H H R N Q C Q Y C R L K K C F R V G M R

aaagaagctgtccagcgaggtagaatcccacctgcccactctagtgccagtcccacttct

K E A V Q R G R I P P A H S S A S P T S

gctccgggagcaggcgagtacttcaatggacagcccgtctctgaactgatctcccagctc

A P G A G E Y F N G Q P V S E L I S Q L

ctgagagctgagccatatccagcttcccgctatgggtcccagtacgctcaacagggtagt

L R A E P Y P A S R Y G S Q Y A Q Q G S

gtgatgggcattgataatatctgtgagttggcggcacggcttctctttagcactgttgaa

V M G I D N I C E L A A R L L F S T V E

tggtcaaggaacatcccctatttcccagaacttgctatggctgatcaggtctcattgctt

W S R N I P Y F P E L A M A D Q V S L L

cgactgagctggagtgaactctttgtacttagtgctgctcagtctgcccttccactccac

R L S W S E L F V L S A A Q S A L P L H

atggctccccttttggctgctgctggattccatgcttctccaatgtctgctgaccgagtc

M A P L L A A A G F H A S P M S A D R V

gtctcatttatggatcagattcgtctcttccaggaccaggttgagaagcttaaccgcctg

V S F M D Q I R L F Q D Q V E K L N R L

caggtggactcggctgaatatgcttgtttaaaagctattgcactcttcacttcag**a**tgcc

Q V D S A E Y A C L K A I A L F T S **D** A

tgtggcctcacagatccagctcacgtggaaagcttacaagaaaaagcccaggtagccctc

C G L T D P A H V E S L Q E K A Q V A L

acagaatatgtccgagcccagtatccctctcagccccagcgctttgggcgtctcctctta

T E Y V R A Q Y P S Q P Q R F G R L L L

cgactgcctgcacttcgagctgttcctgcatcactcatatcccagctcttcttcatgagg

R L P A L R A V P A S L I S Q L F F M R

cttgtcggcaagacccctatagaaacacttattagggacatgcttctgtctgggagtagt

L V G K T P I E T L I R D M L L S G S S

tttaattggccatactcatctggtcaatag

F N W P Y S S G Q -
